# Recovery of balance and walking in people with ataxia after acute cerebral stroke: study protocol for a prospective, monocentric, single-blinded, randomized controlled trial

**DOI:** 10.3389/fstro.2024.1388891

**Published:** 2024-08-05

**Authors:** Patricia Meier, Lukas Mayer-Suess, Stefan Kiechl, Ulrike Pachmann, Raphaela Greimann, Markus Kofler, Christian Brenneis, Astrid Grams, Ruth Steiger, Barbara Seebacher

**Affiliations:** ^1^Department of Neurology, Medical University of Innsbruck, Innsbruck, Austria; ^2^VASCage, Centre on Clinical Stroke Research, Innsbruck, Austria; ^3^Department of Neurology, Hochzirl Hospital, Hochzirl, Austria; ^4^Department of Neurology, Clinic for Rehabilitation Münster, Münster, Austria; ^5^Karl Landsteiner Institute for Interdisciplinary Rehabilitation Research, Münster, Austria; ^6^Department of Radiology, Neuro Imaging Research Core Facility, Medical University of Innsbruck, Innsbruck, Austria; ^7^Department of Rehabilitation Science, Clinic for Rehabilitation Münster, Münster, Austria

**Keywords:** ataxia, acute stroke, coordination exercises, postural control, balance, walking

## Abstract

**Introduction:**

Posterior circulation stroke can lead to ataxia, manifesting in a loss of coordination and balance. Patients experience difficulty in activities of daily living and an increased risk of falling, both profoundly affecting quality of life. In individuals with neurodegenerative diseases, coordination exercises have been shown to lead to a reduction in ataxic symptoms. There is, however, limited evidence on the effect of physical therapy, specifically coordination exercises in patients with stroke-related ataxia. We therefore present a study protocol for a prospective trial.

**Methods:**

The purpose of this trial is to investigate the effects of coordination exercises compared to standard physiotherapy on balance and walking in ataxic stroke patients. Therefore, a prospective, single-blinded randomized controlled trial is currently ongoing at the Clinical Department of Neurology, Medical University of Innsbruck, Austria, in collaboration with two local rehabilitation facilities in Austria, Hochzirl Hospital and the Clinic for Rehabilitation Münster. Balance is the primary outcome of the study as assessed using the Berg Balance Scale. Secondary outcomes are concerned with walking, risk and number of falls, independence in daily life, and quality of life, rated using appropriate scales and scores. Patients are allocated applying a 1:1 ratio and a stratified block randomization. In both groups recruited individuals undergo five 45-min treatment sessions per week, totaling 20 sessions of coordination exercises (IG) or standard physiotherapy (CG) over the course of 4 weeks. Data is collected at the baseline (T0), after the 4-week supervised practice (T1), and after another 8 weeks of independent home-based training (T2).

**Discussion:**

This is the first randomized controlled trial investigating the effects of coordination exercises on balance and walking in people with stroke-related ataxia. As stroke guidelines emphasize the limited evidence of treatment for ataxic symptoms, this study aims to contribute further knowledge regarding tailored interventions for these patients.

**Clinical Trial Registration:**

German Clinical Trials Registry (drks.de). Identifier: DRKS00020825.

## 1 Introduction

The worldwide yearly incidence of stroke is 16.9 million, with ataxia being one of the most common symptoms when the vertebrobasilar system is affected (Feigin et al., 2014). Most frequently, infarction of the superior cerebellar artery (SCA), the posterior inferior cerebellar artery (PICA), or the anterior inferior cerebellar artery (AICA) lead to deficits in coordination (Kim and Caplan, 2016). Ataxia can also manifest in a loss of balance, gait ataxia, dysmetria, dysarthria, and nystagmus. Gait ataxia is evidenced by a staggering gait pattern, irregular foot placement and stride length, and an increased stance width (Cabaraux et al., 2023). When balance impairment is severe, it significantly hampers an individual's ability to perform activities of daily living. The loss of balance and coordination increases the risk of falling, which is severely impacting quality of life (Belas Dos Santos et al., 2018).

Still, research on physical therapy approaches for patients with ataxia after stroke is scarce (Kruger et al., 2007; Marquer et al., 2014; Intercollegiate Stroke Working Party, 2023). Previous studies have investigated treatment approaches such as treadmill training (Bultmann et al., 2014), robot-assisted gait training (Kim et al., 2019), trunk exercises (Stoykov et al., 2005), or a combination of balance and walking exercises (Kruger et al., 2007). Unfortunately, these methods did not demonstrate a significant effect on coordination (Bultmann et al., 2014).

Most of the studies on rehabilitation of people with ataxic symptoms have been conducted in patients with degenerative diseases, like Friedreich's ataxia, multiple sclerosis, or degenerative cerebellar ataxia. In contrast to the studies in stroke-related ataxia mentioned above, most of these trials primarily involved coordination exercises and yielded promising results, reducing the progression of ataxic symptoms (Ilg et al., 2009, 2010, 2012; Miyai et al., 2012; Synofzik et al., 2013; Sartor-Glittenberg and Brickner, 2014). Another strength of coordination exercises was their easy applicability into clinical physical therapy practice (Brötz et al., 2007; Ilg et al., 2010; Marquer et al., 2014). Given that coordination exercises showed symptom reduction in patients with progressive diseases, it seems plausible to anticipate effects in patients with ataxia after stroke. The feasibility of incorporating coordination exercises into rehabilitation of acute stroke patients has already been shown in a pilot trial (Meier et al., 2021). These results underscore the need for a randomized controlled trial to explore their effectiveness with an appropriate number of participants.

## 2 Methods and analysis

### 2.1 Aims and design

A prospective, single-blinded, randomized controlled trial (RCT) is currently being conducted in a monocentric setting in Austria. The aim is to investigate the effects of coordination exercises compared to standard physiotherapy, on balance and walking in patients who suffer from stroke-related ataxia.

### 2.2 Study setting

The initial phase of the study is conducted on the stroke unit at the Clinical Department of Neurology, Medical University of Innsbruck, Austria, which includes a large, comprehensive stroke center. The second study phase is performed at the rehabilitation facility Hochzirl Hospital, Austria, or the Clinic for Rehabilitation Münster, Austria, as patients are usually transferred to a rehabilitation facility 2 weeks after a stroke. The choice of the rehabilitation facility and the timing of the transfer can differ based on individual requirements and the capacities of the rehabilitation facilities.

### 2.3 Participants

Patients are recruited at the Clinical Department of Neurology, Medical University of Innsbruck, Austria. All patients who are admitted due to an acute stroke are evaluated for eligibility. If the patient meets all inclusion criteria and none of the exclusion criteria, the investigator will seek an informative briefing with prospective subjects concerning the study details and a written informed consent is obtained prior to study enrolment.

#### 2.3.1 Inclusion criteria

People who are at least 18 years of age, who experienced an acute ischemic or hemorrhagic stroke (identified by MRI/CT), and who suffer from gait ataxia are eligible, if they score at least 1 point on the items gait, stance, trunk or heel-shin-slide of the Scale for the Assessment and Rating of Ataxia (SARA; Schmitz-Hübsch et al., 2006) and 47 points or less on the Berg Balance Scale (BBS; Stevenson, 2001).

#### 2.3.2 Exclusion criteria

Exclusion criteria are a modified Rankin Scale (mRS) score of 5 or more (Rankin, 1957), a previous stroke leading to persistent debilitating neurological deficits defined as an mRS score of 3 or more, a comorbidity that limits active study participation (e.g., life expectancy of <3 months, alcohol or drug abuse), physical or mental conditions that would not allow safe participation in the study or would influence the assessment of outcomes (e.g., dementia, cardiac insufficiency, severe aphasia, etc.), or pregnancy.

#### 2.3.3 Randomization, allocation, and blinding

Participants are randomly allocated into one of two groups. Group allocation uses a 1:1 ratio to either control group (CG) or intervention group (IG) utilizing stratified blocked randomization and hidden block sizes. The allocation sequence is generated using an online randomization software (Sealed Envelope Ltd., London, UK; Sealed Envelope Ltd., 2022). Stratification variables are age (≤ 70 years, >70 years) and gender (male, female).

Allocation concealment is conducted, as an independent researcher in charge for the randomization does not disclose patients' group allocation until after the baseline assessment (T0). To effectively conceal the randomization sequence, sequentially numbered, opaque sealed envelopes are used. After T0 assessment, department staff who are independent of assessment and intervention assign the patient to the appropriate group using the sealed envelopes. The staff then informs the therapist responsible for providing the assigned intervention.

Outcome assessors are blinded to patients' group assignment. Patients are informed that an examination of the effects of two distinct physiotherapy exercise programs is performed. However, they remain unaware of the precise details of the interventions of both groups and the study's rationale. Patients are encouraged not to talk to the assessor about the exercises. The planned time for regular unblinding is after study completion.

### 2.4 Intervention

Physical therapy sessions start after T0 assessment in both groups. Over the course of 4 weeks patients receive 20 supervised therapy sessions, five 45-min sessions per week, in addition to multidisciplinary rehabilitation treatment (Table 1).

**Table 1:** Schedule of enrolment, interventions, and assessments.

		**T0^a^**	**W1–W4**	**T1^b^**	**W5**	**W6**	**W7**	**W8**	**W9**	**W10**	**W11**	**W12**	**T2^c^**
Enrolment	Eligibility screen	X											
	Informed consent	X											
	Group allocation	X											
Data collection	Demographic data^d^	X											
	Stroke location, type, lesion side, and timepoint	X											
	Duration of rehabilitation stay												X
	mRS	X		X									X
	SARA	X		X									X
	BBS	X		X									X
	TCT	X		X									X
	FES-I	X		X									X
	Posturography: TYMO^®^	X		X									X
	FRT	X		X									X
	FAC	X		X									X
	TUG	X		X									X
	FGA			X									X
	SINGER			X									X
	EQ-5D-3L			X									X
	MRI^e^	X											X
	Adherence evaluation^f^					X		X		X			X
	Number of falls		X		X	X	X	X	X	X	X	X	
	Adverse events		X		X	X	X	X	X	X	X	X	
Intervention	Supervised intervention		X										
	Homebased intervention		X		X	X	X	X	X	X	X	X	

^a^Assessment at baseline.

^b^Assessment after 4-week intervention.

^c^Assessment after 8 weeks of home exercise.

^d^Demographic data include age and sex.

^e^Patients with cerebellar infarction (according to amendment).

^f^Phone calls in week 6, 8, and 10 and return of the checklist in week 13.

W, week; mRS, modified Rankin Scale; SARA, Scale for the Assessment and Rating of Ataxia; BBS, Berg Balance Scale; TCT, Trunk Control Test; FES-I, Falls Efficacy Scale International; FRT, Functional Reach Test; FAC, Functional Ambulation Categories; TUG, Timed Up and Go; FGA, Functional Gait Assessment; SINGER, Scores of Independence for Neurologic and Geriatric Rehabilitation; EQ-5D-3L, EuroQol five dimensions questionnaire; MRI, Magnetic resonance imaging.

Coordination exercises (intervention group, IG) are compared to standard physiotherapy (control group, CG) i.e., state of the art treatment according to clinical guidelines, that patients receive in a clinical routine.

Exercises in both groups are consolidated into an exercise program, which aids in selecting the appropriate exercises depending on the severity of the symptoms (see Sections 2.4.1 and 2.4.2). Typically, exercises for patients with more severe symptoms are listed at the beginning of the program with lower numbers, gradually advancing in difficulty as the numbers increase. Conversely, exercises for individuals with milder impairments are positioned further in the program with higher numbers, reflecting a progression toward more challenging tasks.

#### 2.4.1 Coordination exercises (intervention group, IG)

**Figure 1:**
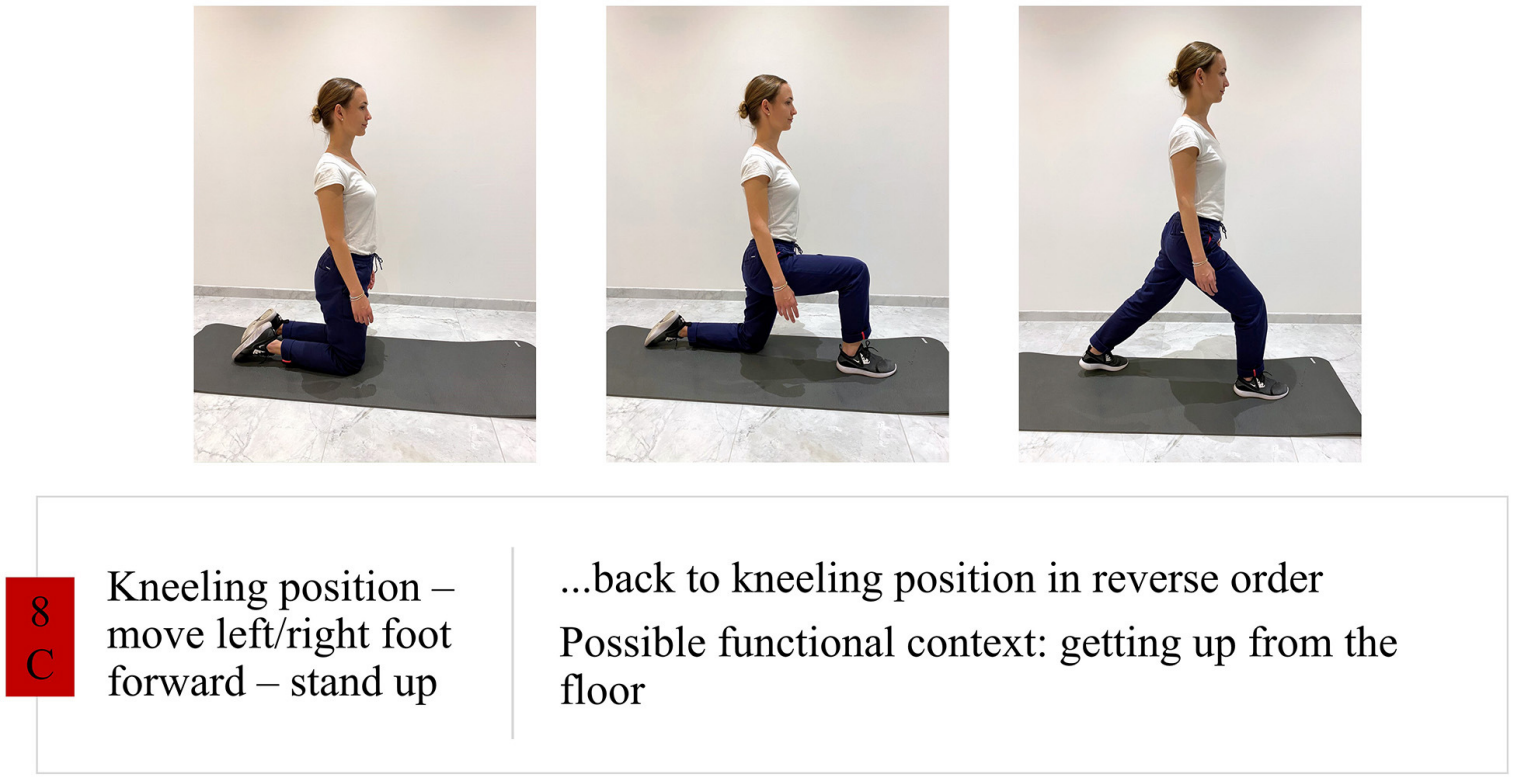
Intervention group exercise example.

Coordination exercises (IG) are part of ataxia-specific neurorehabilitation and focus on high repetition while allowing rather than suppressing body sway associated to ataxia (Brötz et al., 2007). Coordination exercises focus on repetitive trunk and limb movements, dynamic balance, and gait training, including training of protective movements and prevention of falling (Synofzik et al., 2013; Figure 1). The following briefly outlines the main aspects and principles of coordination exercises:

- Goals and exercises in therapy should be relevant to everyday life.
- Fixation mechanisms, e.g., elevated shoulders, should be identified and resolved so that sway becomes visible (Brötz et al., 2007).
- Patients are taught how to identify and resolve fixation mechanisms over the course of therapy.
- Free joint mobility, especially in the upper extremity and trunk, should be achieved.
- Movement sequences should be challenging for coordination, rich in variation, and at the limit of performance.
- The training is focusing on dynamic balance.
- Fall training and training of protective steps are part of every therapy session.
- Few different exercises are trained with a high number of repetitions.

Considering stroke rehabilitation in general, exercises follow the principles of motor learning, focusing on high repetition of exercises (Kwakkel et al., 2023). Bearing in mind cerebellar pathology, resulting in difficulties in motor learning (Hatakenaka et al., 2012), a high number of repetitions seems to be of special importance for patients with cerebellar lesions to compensate for difficulties in trial-and-error learning (Kelly and Shanley, 2016).

Regarding the number of repetitions and variety of exercises, a choice of 3 different exercises is recommended, each having at least 10 repetitions (per side), with a common functional goal (e.g., sit-to-stand) and their combination into one set. In a 45-min session, 3–4 sets can be performed.

Shaping is achieved by transitioning from rather static to dynamic balance exercises, from slow to fast movements (e.g., foot tapping exercises), and from single joint to complex multi-joint exercises (Ilg et al., 2009). The threshold for progressing exercises is determined by the patient's ability to resolve fixation mechanisms, with assistance from the therapist if needed. Resolving fixation mechanisms is addressed using a combination of hands-on facilitation paired with external feedback or solely through feedback, depending on the stage of motor learning. Feedback is provided by the therapist through verbal cues like “relax your shoulders” and as positive feedback on successful movements. Throughout the training, patients are guided to become aware of fixation mechanisms and learn to resolve them independently. By resolving fixation mechanisms free joint mobility is achieved. To work toward functional goals, for instance, if the objective is to ambulate from the bed to the toilette without any aid, the initial exercises may begin in a seated position and progress through activities such as sit-to-stand, standing, stepping, and eventually, walking exercises. Throughout this process, the therapist always ensures that the functional value and purpose of each exercise are clearly communicated. In order to keep the exercises challenging and at the individual performance limit until the end, i.e., when patients are already able to perform exercises on uneven ground, coordination exercises can be performed using trampolines (Giagazoglou et al., 2015). These exercises still follow the same principles.

Further details are reported according to the Template for Intervention Description and Replication (TIDieR; Hoffmann et al., 2014; Supplementary Data Sheet 1). Exercises of the IG are summarized in a handout, which is provided in English (Supplementary Data Sheet 2) and German language (Supplementary Data Sheet 3).

#### 2.4.2 Standard physiotherapy (control group, CG)

**Figure 2:**
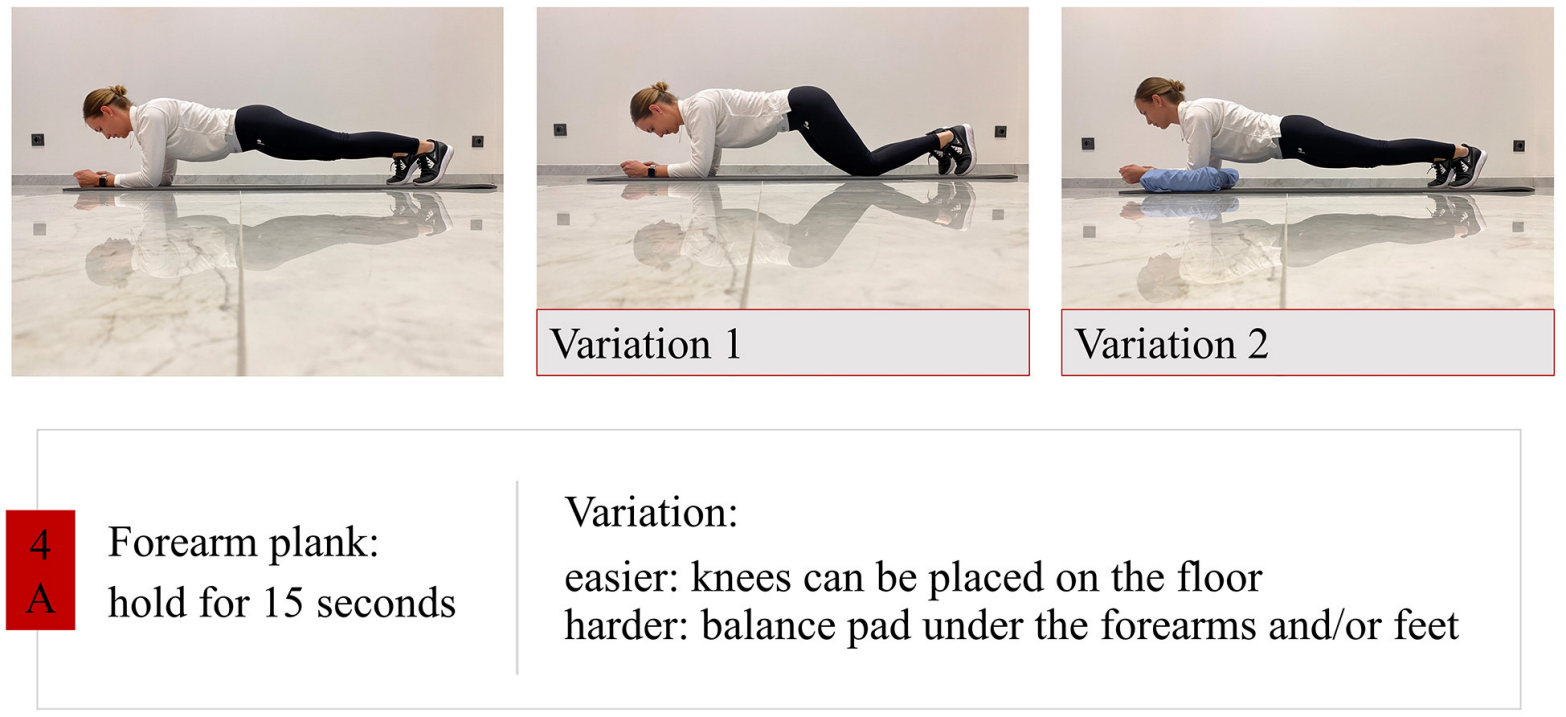
Control group exercise example.

Standard physiotherapy (CG) includes conventional post-stroke rehabilitation exercises. Exercise types were evaluated using a questionnaire for the pilot trial (Meier et al., 2021), confirmed for this RCT in the three study centers and corresponding with standard physiotherapy according to guidelines for stroke rehabilitation (Winstein et al., 2016; Intercollegiate Stroke Working Party, 2023). Exercises in CG can be categorized into four different groups:

- Trunk stability training: strengthening and segmental stabilization (Figure 2).
- Training activities of daily living (ADL): dressing, washing, changing body position (e.g., lie-to-sit, sit-to-stand) with and without aids.
- Walking training: walking with assistance or with an assistive device, variation of step length/step width/walking speed, stair climbing, walking on uneven ground.
- Balance training: using balance pads, exercises for shifting the body center of gravity, exercises with reduced support-surface.

The central principle of the CG is to keep goals and exercises in therapy relevant to everyday life. Therefore, the exercises are designed to target specific activities involving intricate movements such as transitioning from lying to standing, dressing oneself, or preparing a cup of coffee. While each repetition may take longer compared to the IG due to the complex sequence of activities involved, the exercises should still strive for a high number of repetitions in line with general stroke rehabilitation guidelines. The repetition count may vary depending on factors such as task duration, allowing for variability within the therapy session to cater to the patient's performance levels. This variability aims to maintain a level of challenge and interest for the patient. Additionally, considering the patient's condition is crucial; for instance, acute stroke patients may experience dizziness with repeated changes in body positions. Similar to the IG, exercise difficulty is adapted to the patient's limit of performance, but patients in the CG can receive support from the therapist or an assistive device to be able to perform the exercise. Another aspect in this group is to emphasize balance training, stability training and strengthening of the trunk.

Further information about the exercises in this group are reported using the TIDieR (Supplementary Data Sheet 4). The exercise handout for the CG is provided in English (Supplementary Data Sheet 5) and German language (Supplementary Data Sheet 6).

#### 2.4.3 Home exercise program

In addition to the supervised practice, patients in both groups are asked to practice independently 5 times per week, for 15 min throughout the 4-week intervention period (total 20x). The exercises for each day are provided during the supervised sessions according to their group assignment and level of performance.

Upon completing supervised treatments, both IG and CG patients are given a personalized home exercise program as a handout. This program includes a variety of exercises, tailored to each patient. Patients have the flexibility to choose from these exercises for their independent practice, which is recommended 5 times per week, each session lasting 15 min, over an 8-week period until the last visit (T2 assessment), totaling 40 sessions (Table 1). If IG patients have safely practiced with trampolines during supervised treatment, they are provided with a trampoline for continued exercise performance.

#### 2.4.4 Similarities and differences of the interventions

Both interventions follow the principles of motor learning; however, there are distinct differences between them. In the IG, exercises typically involve more repetitions but less variability in practice due to the exercise nature. Conversely, in the CG, there is more practice variability with fewer repetitions. The IG emphasizes allowing the body to sway and solving fixation mechanisms, which is not a central focus in the CG. Patients in the CG concentrate on enhancing stability through trunk exercises while in the IG, this aspect is not as prominent. The CG places a stronger emphasis on training for activities of daily living compared to the IG. Both interventions include walking training, but the approach differs. In the CG, walking training is scheduled regularly regardless of support levels, whereas in the IG, gait training is preferred at times when it can be executed without fixation mechanisms, therapist assistance, and with a focus on coordination.

#### 2.4.5 Strategies to improve adherence to interventions

To document independent practice sessions and facilitate adherence, patients are provided with a checklist. To enhance adherence further, patients undergo semi-structured phone call interviews every 2 weeks. These interviews encompass questions on the exercise frequency, experience and any issues encountered during the exercises, including aspects such as the exercise environment and motivation. Additionally, the interviews cover questions regarding completion of adherence and falling checklist (Table 2).

**Table 2:** Semi-structured telephone interview questions.

	**Question**	**Prompts**
1.	Please indicate how often you practice per week?	If not 5 times per week: What prevents you from practicing 5 times per week?
2.	Please indicate for how long you practice per day?	If not 15 minutes per day: What prevents you from practicing 15 min per day?
3.	Please describe how you get along with the exercises.	If there are problems: Could you provide explanations for this?
4.	Are you using the compliance checklist for documentation?	If not: Could you provide explanations for this?
5.	Are you using the fall protocol?	If not: Could you provide explanations for this?

#### 2.4.6 Relevant concomitant care

While a multi-disciplinary rehabilitation regimen may encompass speech therapy and occupational therapy, these are only included if deemed necessary for the patient. There are no additional one-on-one physiotherapy sessions beyond the trial intervention. Other rehabilitation modalities offered in the rehabilitation facilities may involve robotic interventions or group therapies with an exercise therapy focus (endurance training, strength training, Nordic walking, etc.) or an occupational therapy focus (crafting, gardening, writing training, etc.). The selection of additional therapies for study participants may vary based on individual patient needs, to deliver tailored care.

### 2.5 Data collection

Data is collected at the baseline (T0 assessment), after the 4-week phase of supervised practice (T1 assessment) and after 3 months (T2 assessment; Table 1). T0 and T2 assessments take place at the Clinical Department of Neurology, Medical University of Innsbruck, Austria. T1 assessment is conducted in the patients' rehabilitation facility. Demographic data (age, sex) and stroke-specific data (location, type, lesion side, and timepoint) are extracted from medical records. Outcomes of balance, walking, ataxia, and fear of falling are obtained from trained assessors.

Personal data is pseudonymized using a patient identification number (ID) and only pseudonymized data is transferred to statistical analyses. To ensure the plausibility and integrity of the data, random data checks (value range, field type, and logic checks) are performed, and the CRF data is entered into the database by two independent researchers.

#### 2.5.1 Primary outcome

The primary outcome is balance as assessed using the BBS (Berg et al., 1989). The BBS is considered the gold standard for evaluating balance in people after stroke (Blum and Korner-Bitensky, 2008). It comprises 14 items, and a Likert scale is used ranging from 0 to 4, depending on the patient's performance (Table 3).

**Table 3:** Outcomes, assessments, and timepoints.

**Outcome**	**Assessment**	**T0^a^**	**T1^b^**	**T2^c^**
**Primary outcome**		
Balance	Berg Balance Scale (BBS)	x	x	x
**Secondary outcomes**		
Severity of ataxic symptoms	Scale for the Assessment and Rating of Ataxia (SARA)	x	x	x
Balance	Posturography using the TYMO^®^	x	x	x
Balance (trunk control)	Trunk Control Test (TCT)	x	x	x
Balance	Functional Gait Assessment (FGA)		x	x
Risk of falling	Functional Reach Test (FRT)	x	x	x
Concerns about falling	Falls Efficacy Scale International (FES-I)	x	x	x
Number of falls	Structured log		x	x
Walking	Functional Ambulation Categories (FAC)	x	x	x
Walking	Timed Up and Go (TUG)	x	x	x
Quality of life	EuroQol five dimensions questionnaire (EQ-5D-3L)		x	x
Independence in daily life	Scores of Independence for Neurologic and Geriatric Rehabilitation (SINGER)		x	x

^a^Assessment at baseline.

^b^Assessment after 4-week intervention.

^c^Assessment after 8 weeks of home exercise.

#### 2.5.2 Secondary outcomes

Secondary outcomes are static balance, as assessed using posturography (Tymo Balance Plate, Tyromotion, Austria), dynamic balance during walking, measured using the Functional Gait Assessment (FGA; Thieme et al., 2009), and trunk control, as assessed using the Trunk Control Test (TCT; Collin and Wade, 1990). Additionally, concerns about falling are assessed through the Falls Efficacy Scale International (FES-I; Yardley et al., 2005; Kempen et al., 2007), and risk of falling is determined according to the Functional Reach Test (FRT; Duncan et al., 1990, 1992). The number of falls is documented as well.

Further secondary outcomes include walking ability, evaluated using the Functional Ambulation Categories (FAC; Collen et al., 1990), functional mobility, as measured by the Timed Up and Go Test (TUG; Podsiadlo and Richardson, 1991), health-related quality of life, assessed utilizing the EuroQol questionnaire EQ-5D-3L (Rabin and de Charro, 2001; Greiner et al., 2005), severity of ataxic symptoms, as determined by the Scale for the Assessment and Rating of Ataxia (SARA), and patients' independence in daily life, measured using the Scores of Independence for Neurologic and Geriatric Rehabilitation (SINGER; Funke et al., 2009; Gerdes et al., 2012; Table 3).

Due to the time-point of study recruitment being a susceptible one for diminished physical and mental resilience (i.e., in the acute phase after stroke), FGA, EQ-5D-3L, and SINGER are not performed at T0.

**Figure 3:**
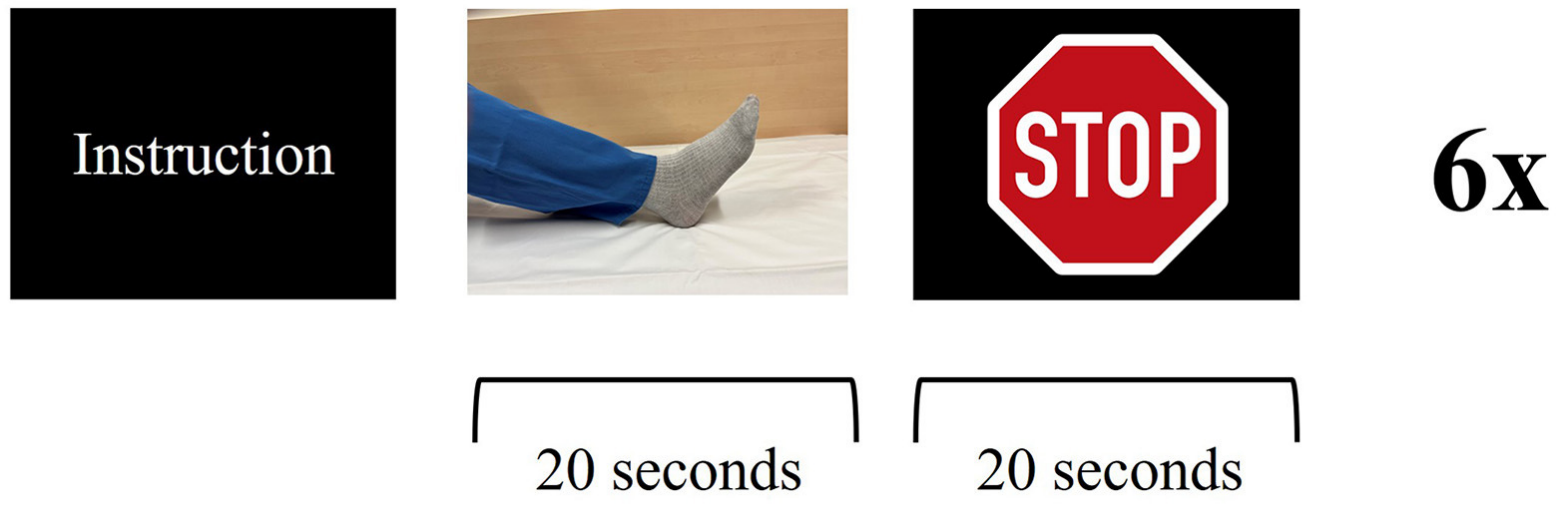
Functional magnetic resonance imaging paradigm—ankle dorsiflexion: the patient receives instruction about the upcoming task. The patient is asked to dorsiflex and plantarflex their foot in a constant, self-paced manner for 20 s, followed by a 20 s break. Visual stimulus presentation for stop (stop sign) and go (picture of a foot). Six cycles for each side.

**Figure 4:**
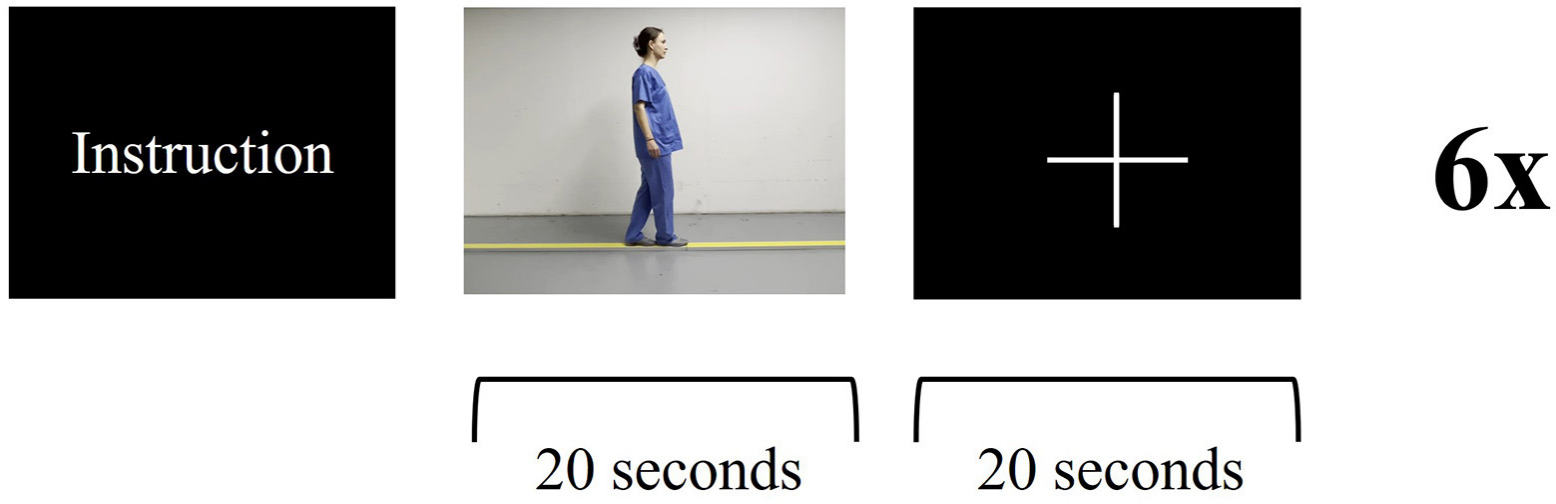
Functional magnetic resonance imaging paradigm—balance task: the patient receives instructions about the upcoming task. The patient is asked to imagine themself balancing on a straight line while watching a video of a person walking on a line for 20 s, followed by a 20s break. Visual stimulus presentation for stop (fixation cross) and go (video of balancing task). Six cycles.

Another secondary outcome for patients with cerebellar infarction is neuronal plasticity occurring over time, measured using magnetic resonance imaging (MRI) at T0 and T2. In addition to structural MRI, functional MRI is performed, including two motor paradigms: ankle dorsiflexion (Figure 3) and a balance task (Figure 4). As it is not possible to physically execute the balance task in the scanner, action observation plus motor imagery (AOMI) is performed while watching a video of a person doing the balance task. A combination of MI and video was chosen, as studies indicate a better activation of balance relevant brain areas than with MI or video alone (Taube et al., 2015; Ruffieux et al., 2018). MI capability is assessed using the Kinesthetic and Visual Imagery Questionnaire (KVIQ-10; Malouin et al., 2007). Mental chronometry, referring to the temporal coupling between real and simulated movement, is measured with a 6-Meter Walk Test (Lam et al., 2010). Assessments are validated to evaluate quality of MI (Schuster et al., 2012; Ma et al., 2022) and temporal consistency in stroke patients (Malouin et al., 2008; Malouin and Richards, 2010; Oostra et al., 2015; De Bartolo et al., 2020). After undergoing both assessments at T0, patients receive an MI training session before each MRI examination.

#### 2.5.3 Adverse event reporting and harms

Any adverse events (AE or serious AE) are recorded systematically, evaluated by authorized study personnel, and reported in accordance with responsible ethics committee requirements.

During the intervention phase (weeks 1–4), the occurrence of all adverse events is recorded by the responsible study center staff and assessed by a member of the medical team. The occurrence of falling during home exercises (weeks 5–12) is recorded by the patient as an adverse event of special interest, given that it serves as a secondary outcome within the study.

### 2.6 Sample size calculation

The sample size was estimated using the primary outcome data obtained from a pilot study (*n* = 7) conducted for this RCT (Meier et al., 2021), taking into account a correction for small samples. Based on a mean change in BBS score of 17.33 points (SD 2.08) in the IG and 13.67 points (SD 4.73) in the CG, the mean between-group difference and pooled SD were calculated and a correction for small samples was applied (pooled SD^*^1.442; Vickers, 2003). Using an independent samples *t*-test, power of 0.8, alpha of 0.05 and 1:1 allocation ratio, a sample size of 66 participants, 33 per group, was evaluated (HyLown Consulting LLC, 2022). Considering an attrition rate of 15%, the aim is to include 77 participants.

### 2.7 Statistical analysis

IBM SPSS software, release 29.0 (IBM Corporation, Armonk, New York, USA), R software version 4.3.2 (R Foundation for Statistical Computing, Vienna, Austria) and GraphPad Prism 10 (San Diego, California, USA) will be used for the data analyses. Descriptive statistics are used for the baseline demographic variables, primary and secondary outcomes. Continuous data is checked for significant outliers using studentized residuals and a normal distribution using the Shapiro Wilk Test, Q-Q plots, and histograms. Frequencies [number (N), %] are presented for counted (N falls, N adverse events if any, N of home exercises) and nominal data (gender, stroke etiology and location, and rehabilitation facility). Medians (interquartile range) are reported for ordinal data (mRS, BBS, FGA, TCT, FES-I, FRT, EQ-5D-3L, SARA, and SINGER), and mean (SD) is reported for continuous data (age, time after stroke, TUG, and posturography outcomes).

The significance level is defined by a two-tailed *p*-value of <0.05. Baseline (T0) between-group differences are evaluated using Fisher's exact test for nominal data, Mann Whitney-*U*-test for ordinal data and independent samples *t*-test for continuous data. If the distribution is non-normal in continuous data, data transformation or non-parametric statistics are performed, as appropriate. To evaluate within-group differences between T0, T1, and T2 measures, for ordinal data, Friedman test is utilized followed by Dunn's multiple comparisons test for pairwise comparisons. To examine between-group differences, using newly created variables (T1 or T2 minus T0 values), a Mann Whitney-*U*-test is performed. For continuous data, test assumptions for a repeated-measures analysis of variance (ANOVA) are checked (e.g., normality, sphericity) and dealt with as appropriate. If the requirements are met, repeated measures ANOVA are performed, with “time” (T0, T1, and T2 measurements) as the within-subject factor, “group” (IG, CG) as the between-subject factor, and adjusted for the stratification variables (age, sex). If statistical significance is present, a *post-hoc* analysis of pairwise comparisons between measurement time points is performed, followed by a *post-hoc* Bonferroni correction for multiple comparisons. An intention-to-treat analysis is performed analyzing all study participants in the groups which they have been assigned to.

## 3 Discussion

This is the first RCT to investigate the effects of coordination exercises in patients with post-stroke ataxic symptoms. As coordination exercises have been shown to reduce the progression of ataxic symptoms in patients with degenerative diseases (Marquer et al., 2014), and stroke guidelines highlight the limited evidence for targeted treatment of ataxic symptoms (Intercollegiate Stroke Working Party, 2023), this study aims to contribute further knowledge regarding tailored interventions in rehabilitation for stroke patients with ataxia. Building on the evidence for degenerative diseases, we hypothesize that stroke patients will experience faster recovery as well. As patients with cerebellar damage experience limitations in the procedural learning process (Molinari et al., 1997), especially trial-and-error learning, compensation must be sought to be able to learn motor tasks more efficiently. A high number of repetitions and intensive training, provided with coordination exercises for example, could be the simplest option (Kelly and Shanley, 2016). To assess the impact of the interventions on motor networks, the trial involves MRI examinations to investigate any potential changes.

However, there are also limitations to consider in this study. Firstly, the study is conducted in a rehabilitation setting where patients receive additional individualized therapies. These supplementary therapies could potentially influence the study results, making it challenging to exclusively attribute the observed effects to the specific intervention under investigation. Secondly, as the trial focuses on acute stroke patients who undergo rapid recovery, selecting a timely and appropriate balance assessment over the 3-month study period is challenging due to the potential ceiling effect. To address this concern, the researchers chose 47 points or fewer in BBS as an inclusion criterion to ensure the detection of significant changes from T0 to T1 (Stevenson, 2001) and included the FGA to demonstrate effects during the sub-acute stages (T1 to T2). This limitation is relatively minor since the primary objective of the study is to evaluate the impact of supervised therapy sessions from T0 to T1. Thirdly, the initial training of therapists conducting the interventions had to be performed online because of the COVID-19 pandemic. This adjustment in training methods may influence the delivery and consistency of the therapy sessions. However, therapists have the option to watch the recording of the training, whenever they feel it is necessary to refresh certain aspects.

## 4 Trial status

The initial version of the protocol was 1.0, dated on February 18th, 2020. An amendment (adding the MRI examination) resulted in version 1.1, dated November 11th, 2022. Participants will be recruited between July 2020 and March 2024, with study completion planned by the end of June 2024.

## Ethics statement

The present study was approved by the Ethics Committee of the Medical University of Innsbruck on July 2nd, 2020 (reference number 1050/2020). An agreement between the investigator and sponsor is provided, changes to the study protocol including reasons were made in writing and signed by all responsible parties. The approval of the responsible ethics committee was obtained on February 9th, 2023. The participants provide their written informed consent to participate in this study. Results from this clinical trial will be published and disseminated to the study participants. Written informed consent was obtained from the individual(s) for the publication of any potentially identifiable images or data included in this article.

## Author contributions

PM: Conceptualization, Data curation, Formal analysis, Funding acquisition, Investigation, Methodology, Project administration, Resources, Software, Supervision, Validation, Visualization, Writing – original draft, Writing – review & editing. LM-S: Investigation, Methodology, Supervision, Writing – review & editing. SK: Conceptualization, Funding acquisition, Methodology, Project administration, Resources, Supervision, Validation, Writing – review & editing. UP: Methodology, Project administration, Resources, Software, Writing – review & editing, Data curation. RG: Investigation, Methodology, Project administration, Resources, Writing – review & editing. MK: Investigation, Methodology, Resources, Supervision, Writing – review & editing. CB: Investigation, Methodology, Project administration, Resources, Supervision, Writing – review & editing. AG: Investigation, Methodology, Resources, Software, Visualization, Writing – review & editing. RS: Methodology, Supervision, Conceptualization, Project administration, Investigation, Resources, Visualization, Writing – review & editing. BS: Conceptualization, Investigation, Methodology, Project administration, Resources, Supervision, Writing – review & editing.
