# Supplementary material for: Recovery of balance and walking in people with ataxia after acute cerebral stroke: study protocol for a prospective, monocentric, single-blinded, randomized controlled trial
Source: Front Stroke. 2024 Aug 5;3:1388891. doi: 10.3389/fstro.2024.1388891 (PMC12802608; doi:10.3389/fstro.2024.1388891)
Supplement: Supplementary file 1 [file Data_Sheet_1.PDF]

TIDieR

Coordination exercises

Why:

Coordination exercises reduce the progression of ataxic symptoms in patients with degenerative diseases. It seems plausible that coordination exercises have significant effects on coordination in stroke patients with ataxic symptoms. The effect of coordination exercises has not yet been studied in stroke patients.

What (material):

For some exercises, floor markings, a trampoline, an exercise mat, juggling balls or everyday objects are needed.
Patients receive their individual home exercise program as a handout. Exercises are selected from the exercise program
(supplementary material). If the patients have already practiced safely with trampolines during the supervised treatment, they are provided with a trampoline so that they can continue to perform the exercises.

What (procedures):

Coordination training aims to restore the ability to act and move in everyday life, thus minimizing fear and occurrence of falls. Coordination exercises follow the principles of motor learning and therefore focus on high repetition while allowing rather than suppressing body sway associated with ataxia. Fixation mechanisms, e.g. elevated shoulder, should be identified and resolved so that sway becomes visible. The patient is taught how to identify and resolve fixation mechanisms over the course of therapy. Exercises should be challenging for coordination and focus on free joint mobility in the trunk and limbs, dynamic balance, protective steps, and prevention of falls. Attention is paid to adapt the exercise difficulty individually to each patient, which can be achieved by changing from single-joint to multi-joint exercises, from slow to fast, from easy to complex movements or vice versa. Coordination exercises can also be performed using trampolines. In this case, the exercises still follow the same principles.

Who provided:

The intervention is delivered by physical therapists of the participating study centers. Staff at each center has been trained in live and online sessions on the details of the intervention and general study requirements. The online sessions (recordings) are available on demand throughout the study. The intervention is carried out as part of their daily routine work.

How (mode of delivery; individual or group):

The intervention is conducted in a one-to-one setting in face-to-face contact. Supervision of the intervention during home exercises is done indirectly via telephone.

Where:

Patients are recruited at the Clinical Department of Neurology, Medical University of Innsbruck, Austria (Center 1), a large medical center with a Stroke Unit. Supervised interventions start in this acute care setting and are continued at the rehabilitation hospital Landeskrankenhaus Hochzirl-Natters, Austria (Center 2) or the Clinic for Rehabilitation Münster, Austria (Center 3), Patients are transferred during the supervised sessions, as a result of routine procedure in Tyrol, where patients receive intensive multidisciplinary rehabilitation in a rehabilitation hospital as early as possible.

When and how much:

Patients receive 4 weeks of supervised training. In addition, patients are asked to train independently for 15 minutes, 5 times per week (total 20x). The exercises for each day will be provided by the therapist conducting the supervised session and selected according to the patient´s level of performance.
After completion of the supervised treatments, patients are asked to practice independently for 5 times/ week (15 minutes each day) for 8 weeks i.e., until T2 assessment (total 40x). Patients practice according to [illegible] Form saved

program, which they receive from the therapist conducting the supervised sessions. Patients can choose out of these exercises to conduct their independent practice.

Tailoring: Adjusting the difficulty and intensity of the exercises to the patient's level of performance is a basic principle of therapeutic treatment and will be performed individually for the patient whenever necessary.

How well (planned): To record the supervised session, therapists are provided with an intervention documentation sheet. To record the independent practice sessions, patients receive a compliance checklist. To record falls during unsupervised practice, patients are provided with a falls protocol. For improving adherence, patients receive semi-structured phone call interviews every 2 weeks. The interview covers questions regarding exercise frequency, experience of any problems related to the exercises, including the exercise environment and motivation, and regarding completion of compliance and falls protocol.

Form saved
