# Supplementary material for: Recovery of balance and walking in people with ataxia after acute cerebral stroke: study protocol for a prospective, monocentric, single-blinded, randomized controlled trial
Source: Front Stroke. 2024 Aug 5;3:1388891. doi: 10.3389/fstro.2024.1388891 (PMC12802608; doi:10.3389/fstro.2024.1388891)
Supplement: Supplementary file 2 [file Data_Sheet_2.PDF]

GLAAS II
Coordination exercises for ataxia[1]

Patricia Meier

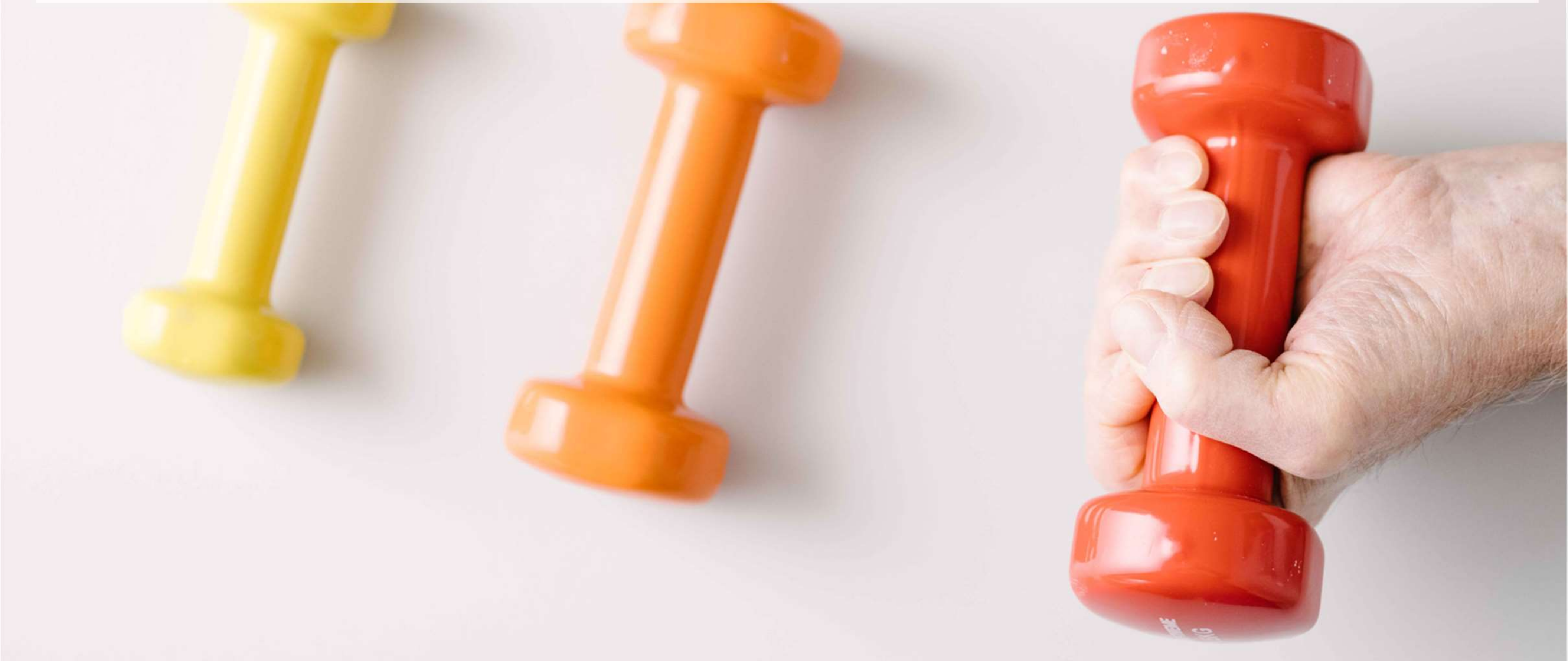

©Rene Böhmer

[1] Exercises based on current literature (Ilg et al. 2009/2010/2012; Brötz et al. 2007) and questionnaires in the study centers of Tyrol

Principles of training (1)

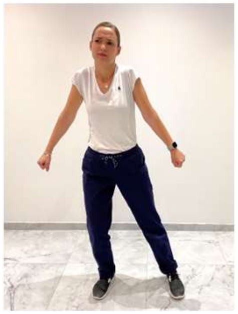

- Fixation mechanisms, e.g., elevated shoulders, should be identified and resolved so that sway becomes visible.
- Patients are taught how to identify and resolve fixation mechanisms over the course of therapy.
- Free joint mobility, especially in the upper extremity and trunk, should be achieved.
- Few different exercises are trained with a high number of repetitions:
  - One exercise should be repeated at least 10 times (if applicable:) per side
  - It is best to combine three exercises (as a set) and then repeat this set three times

Principles of training (2)

- Movement sequences should be challenging for coordination, rich in variation, and at the limit of performance.
- The training of dynamic balance is focused on.
- The goals and exercises in therapy should be relevant to everyday life and the functional context (FC) should always be clear to the patient.
- Fall training and the training of protective steps are part of every therapy session.
- 20 supervised sessions: 5 per week for 45 minutes & 60 sessions of independent training: 5 per week for 15 minutes.

Variation

- Speed fast/slow
- Rhythm (e.g., through auditory cueing)
- Increasing/decreasing the support surface (e.g., stance or track width)
- Change in visual control (e.g., with/without head movements/gaze fixation, open/closed eyes)
- Inclusion of one or more objects relevant to everyday life

Increasing difficulty (1)

You can tell whether the difficulty can be increased by whether the patient can still resolve the fixation mechanisms (the therapist's advice, e.g., to relax the shoulders, is permitted and desired).

- From rather static to dynamic balance exercises
- From slow to fast movements (e.g., foot tapping exercises)
- From single-joint to complex multi-joint movements
- From exercises with one limb to complex movements (involving the whole body)

Increasing difficulty (2)

- From closed to open kinematic chains
- From simple to difficult starting positions (lying, sitting, standing, etc.)
- From simple to complex rhythms
- From undivided to divided attention
- None, one, several objects (for exercises for the upper limb)
- None, one, several obstacles (for exercises for the lower limb)

The difficulty can be increased by one or more factors, but these should be added one after the other.
E.g: sitting: foot tapping slow - sitting: foot tapping fast - standing: foot tapping slow - standing: foot tapping fast

Motor Learning

Principles of Motor Learning:

Repetitive training, Variable practice, shaping, action-oriented and task-specific training, goal-oriented training, frequent training (dosage, duration)

Factors affecting the Motor Learning process:

- Intrinsic/ extrinsic motivation: adequate exercise design and goal setting
- Positive reinforcement: positive feedback on a "successful" movement
- Manual guidance: „hands-off"
- External focus of attention: visual cues, etc.

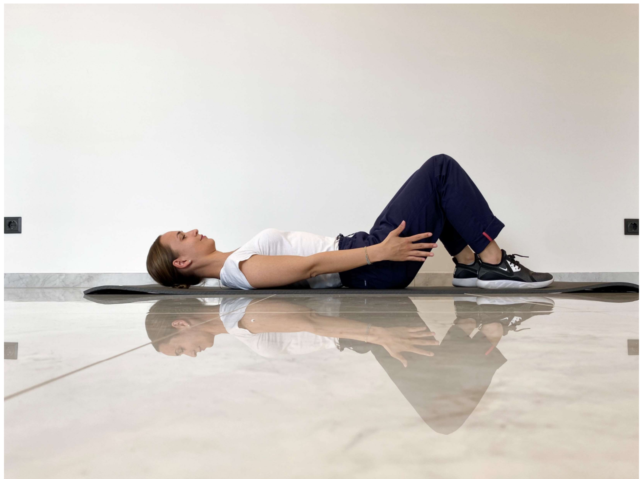

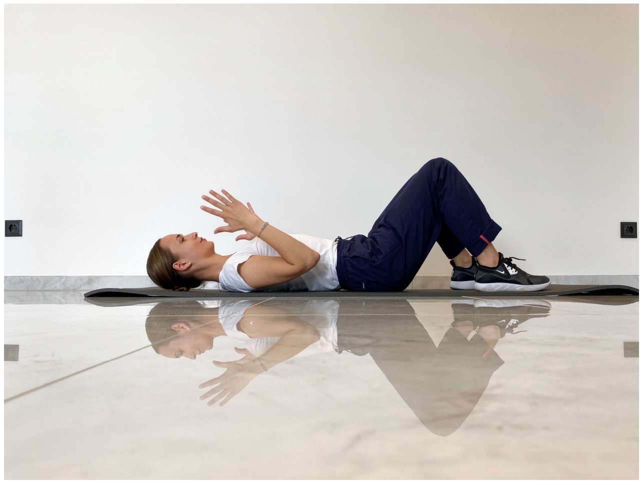

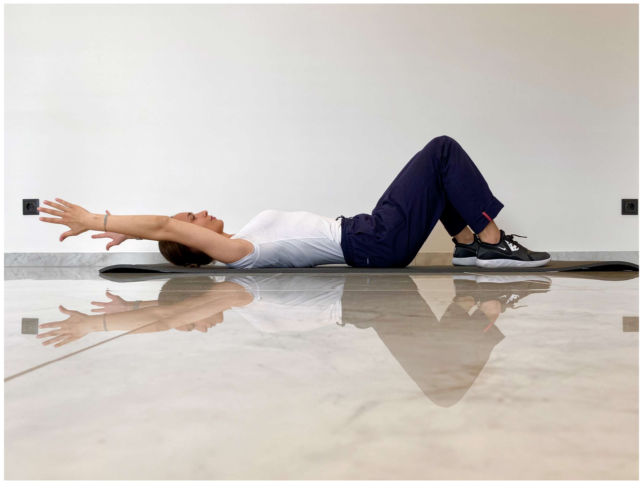

1
A

Arm movements in supine position

- Possible FC (functional context): preliminary exercise for: putting clothes in the wardrobe, making the bed, ...

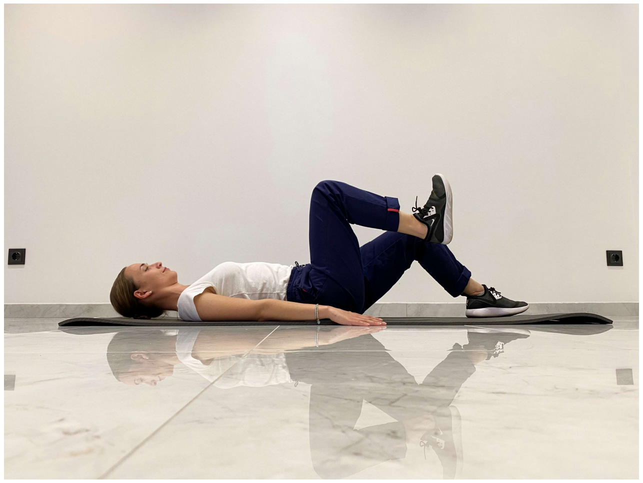
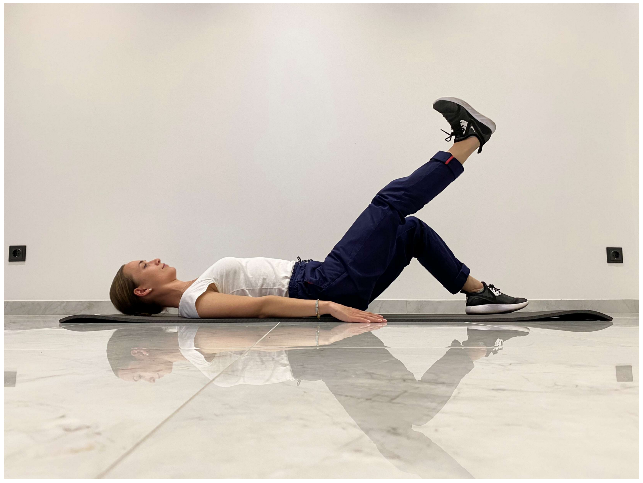
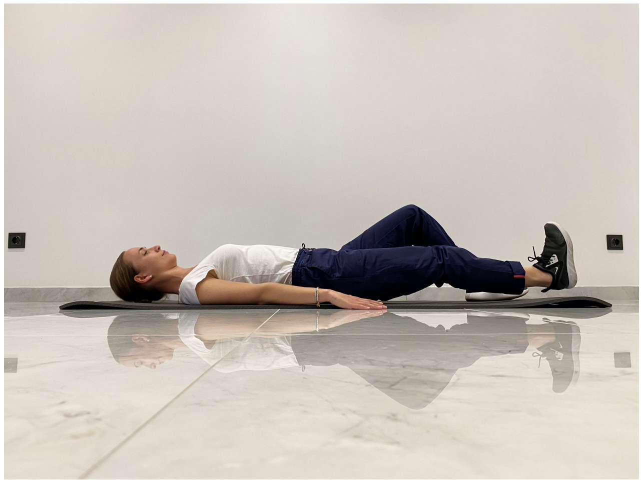

1 B

Leg movements in supine position with left/right leg

- Possible FC: preliminary exercise for: walking, climbing stairs, cycling, ...

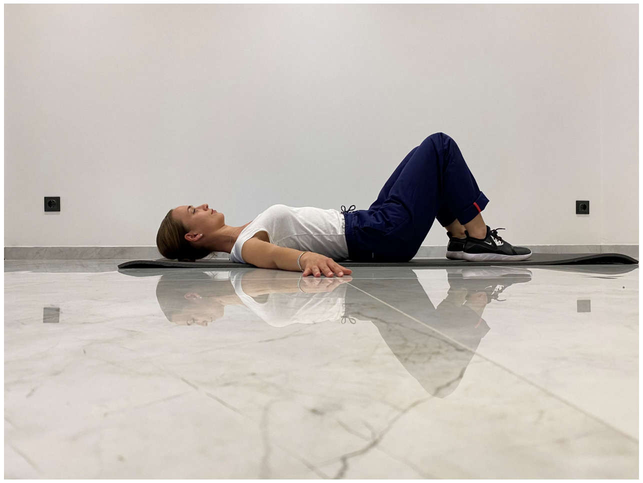 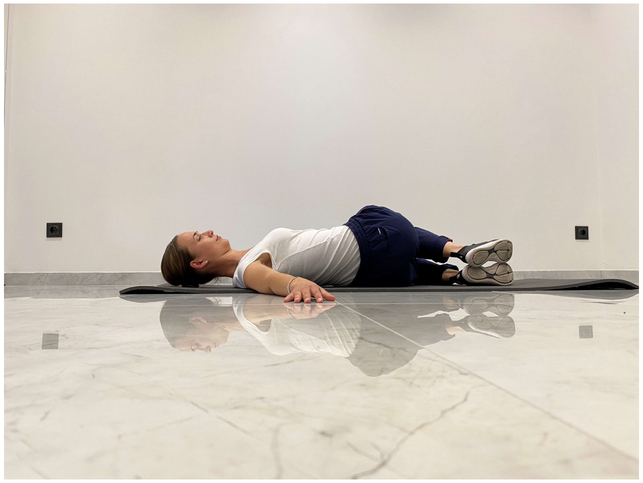 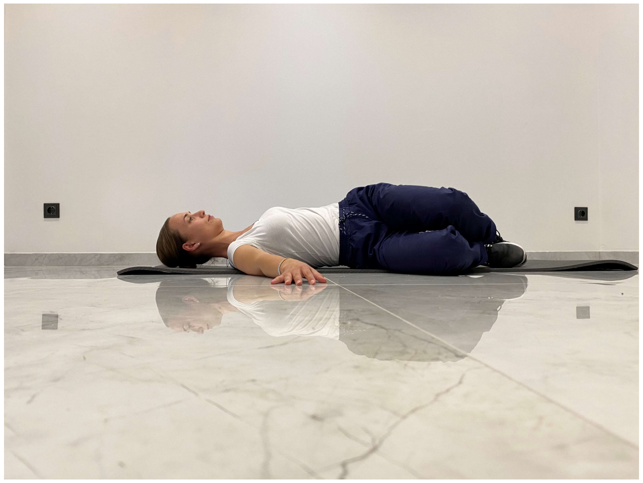

1 C

Rotation of the spine in supine position

- Possible FC: turn over in bed, ...

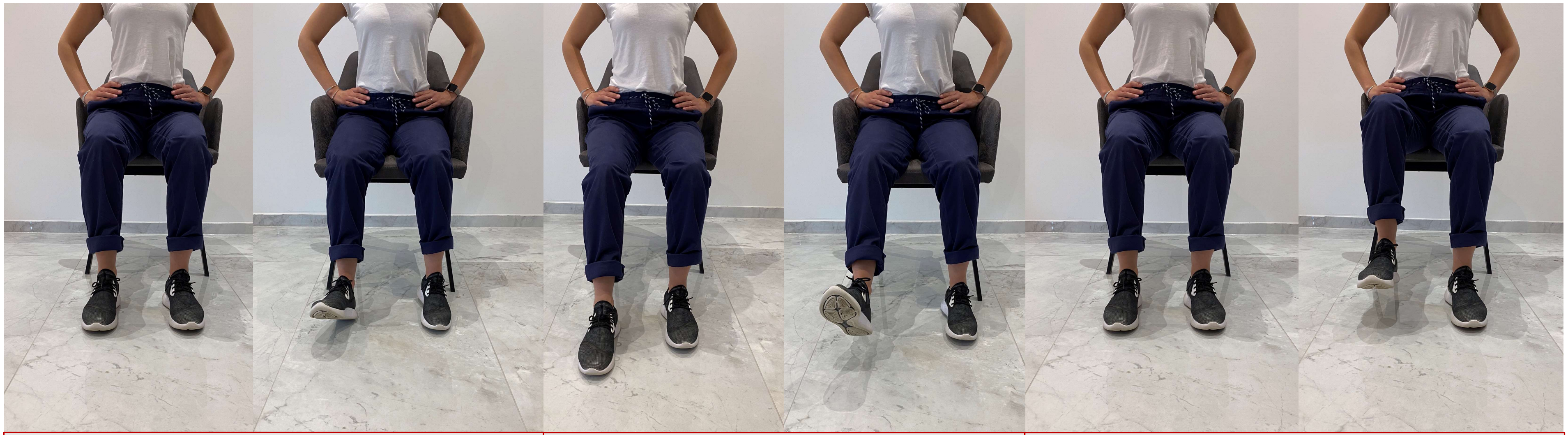

Foot tapping	Tapping with knee movement	Stomping

2 A

Sitting: foot tapping

This exercise serves as an introduction to coordination training and can always be used as a preliminary exercise for new rhythms or speeds.
Tap foot using the entire range of motion, high number of repetitions, tap as fast as possible while keeping the rhythm.

- Variation: tap with movement of the knee, stomp (movement in hip joint), tap softer/louder, tap faster/slower, use various rhythms (e.g., 2x left/1x right).
- Possible FC: preliminary exercise for walking or dancing

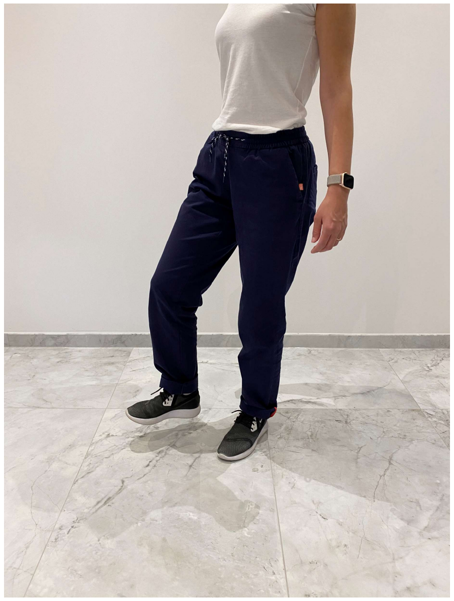 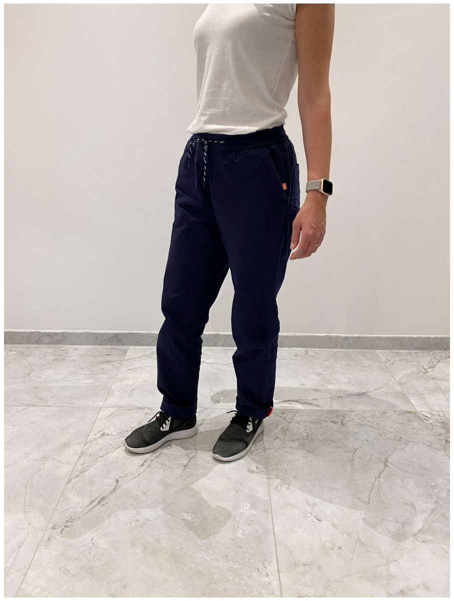

2 B

Standing: foot tapping

This exercise also serves as an introductory or preliminary exercise.

- Variations: stomp (movement in hip joint) tap softer/louder, tap faster/slower, use various rhythms (e.g. 2 times left/1 time right).
- Possible FC: preliminary exercise for walking and protective steps or for dancing

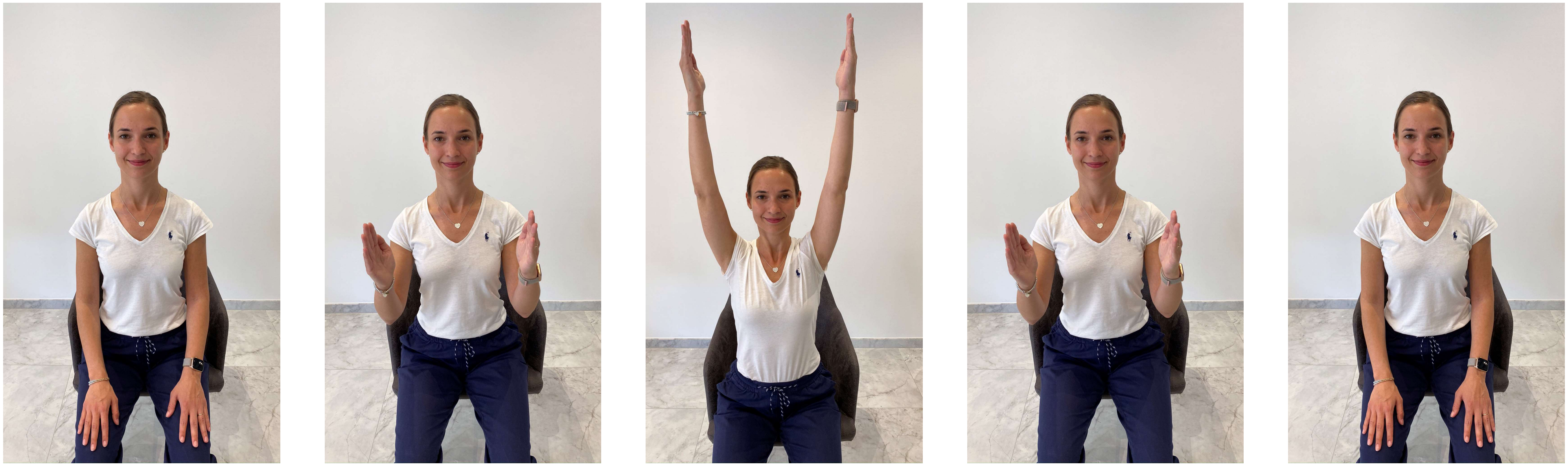

3 A

Sitting: raise arms overhead

- Variation: various Exercises with juggling balls/cloths
- Possible FC: putting clothes in the wardrobe, making the bed, ...

3B = exercise 3A plus...

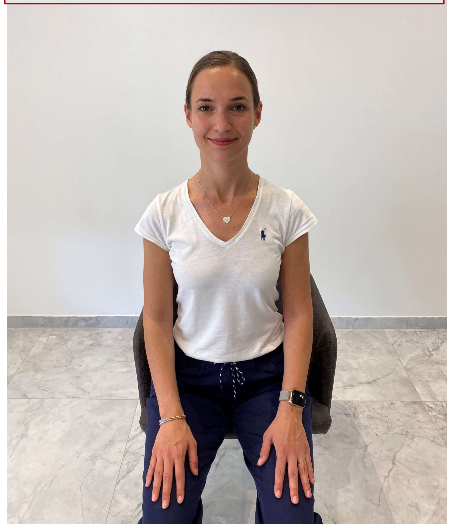

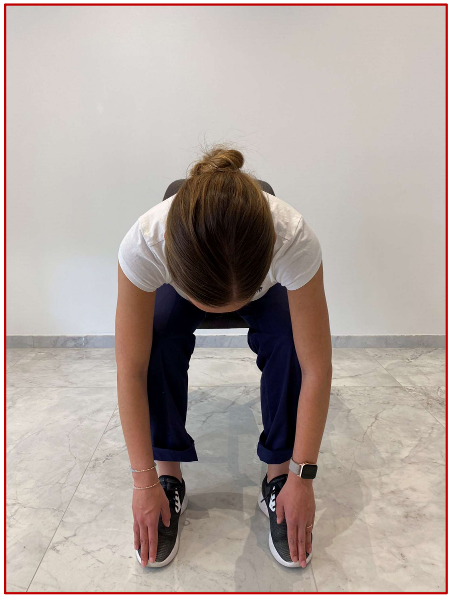

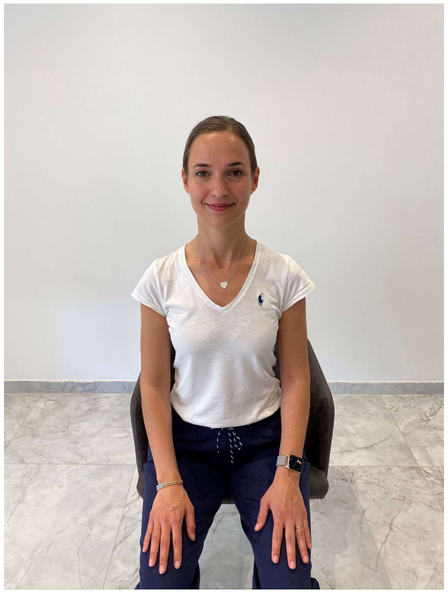

3 B

Sitting: raise arms overhead and then down to the tip of the toes

- Possible FC: put on shoes/socks/pants, ...

3C = exercise 3B plus...

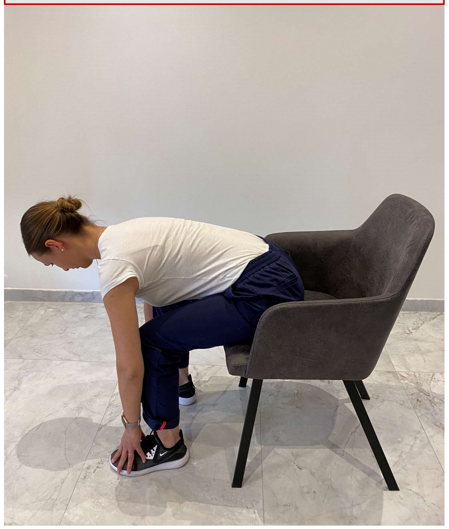

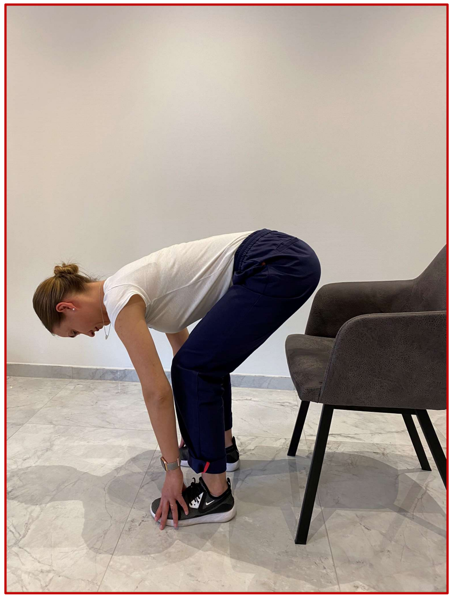

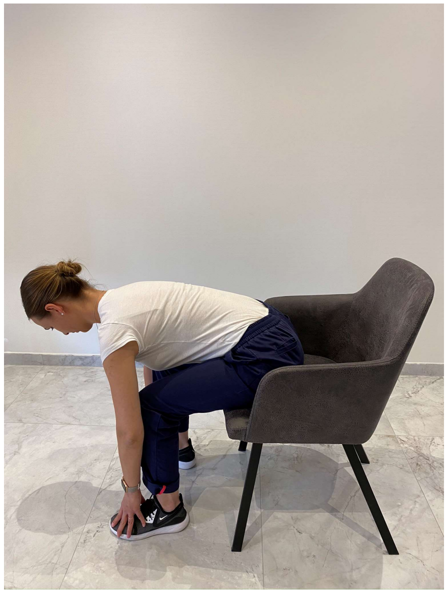

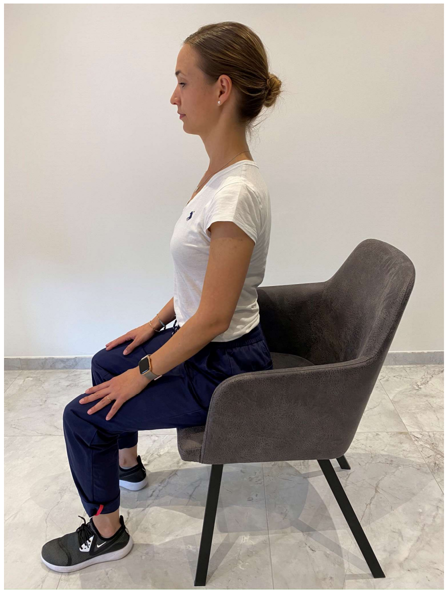

3 C

Sitting: raise arms overhead, then down to the tip of the toes and lift buttocks

...go back in reverse order.

- Possible FC: put on pants, getting up from a low couch, ...

3D = exercise 3C plus...

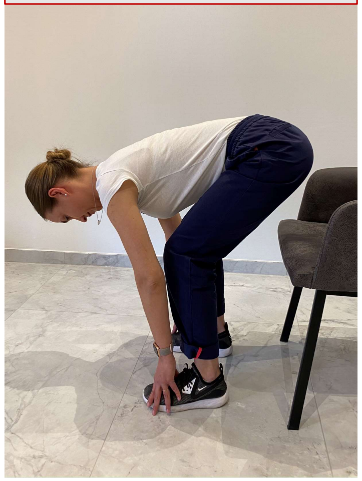

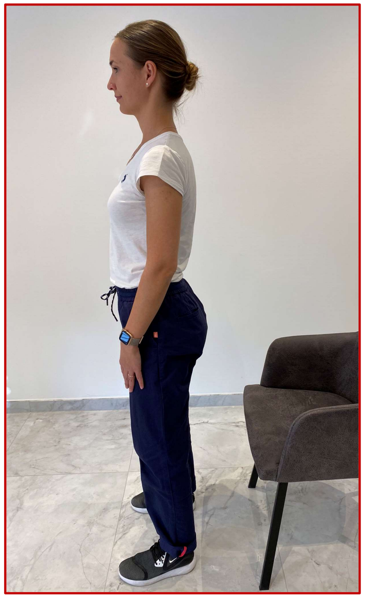

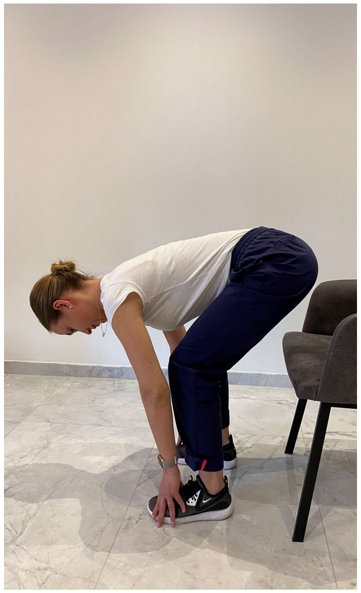

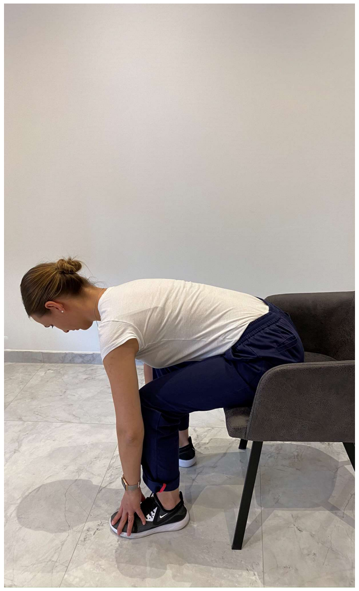

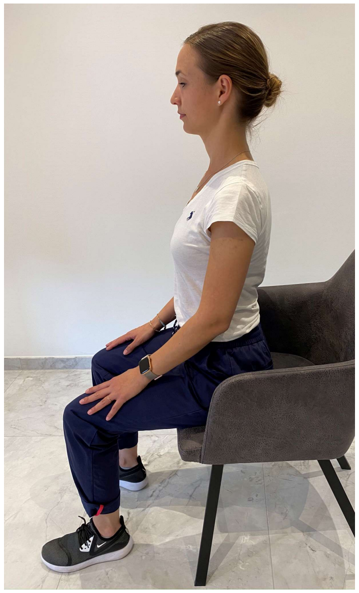

3 D

Sitting: raise arms overhead, then down to the tip of the toes, lift buttocks & stand up

...go back in reverse order.

- Possible FC: put on pants, getting up from a low couch, ...

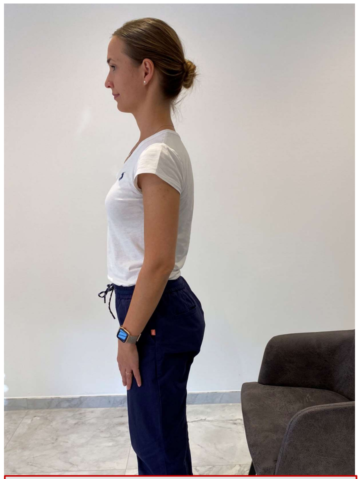

3E = exercise 3D plus...

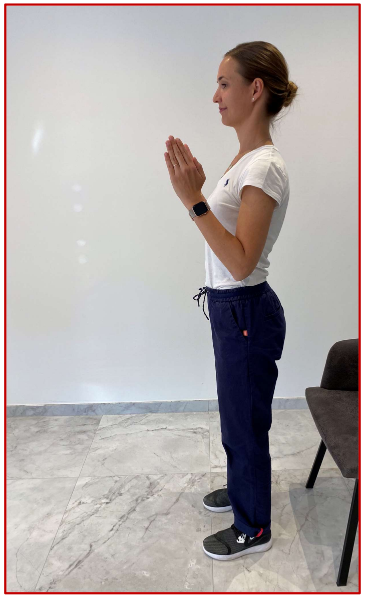

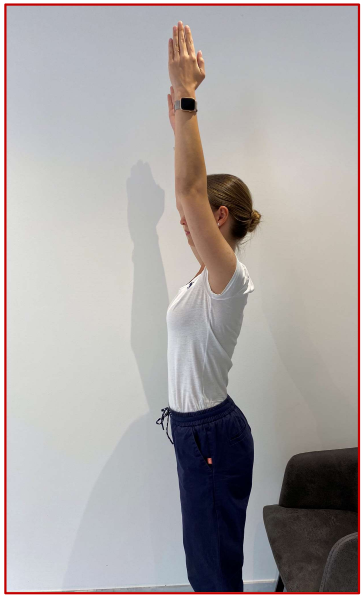

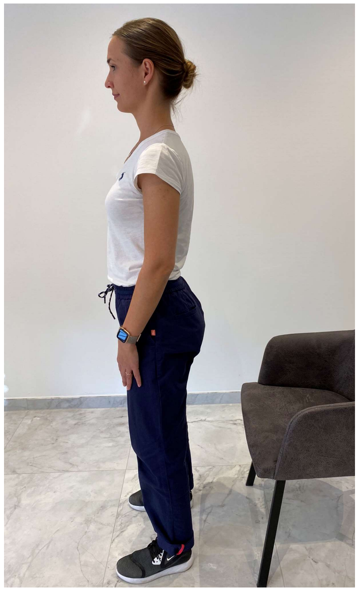

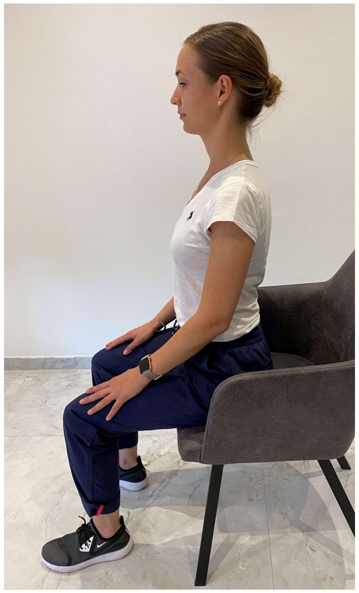

3 E

Sitting: raise arms overhead, then down to the tip of the toes, lift buttocks, stand up & raise arms overhead

...go back in reverse order.

- Possible FC: putting on shoes and taking a coat, getting out of bed in the morning and stretching, ...

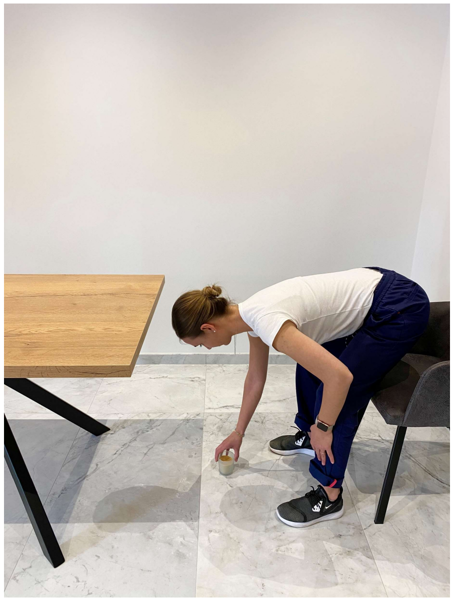

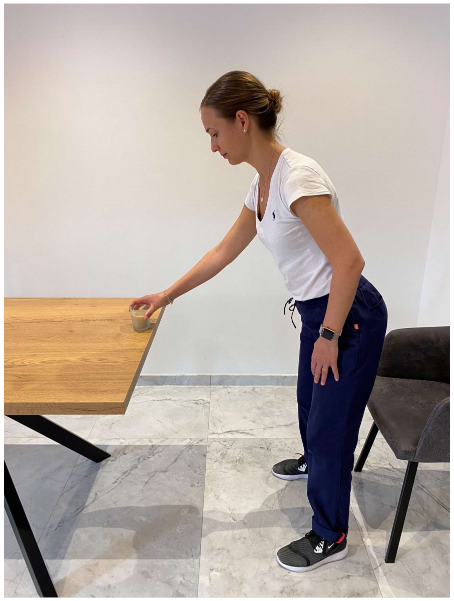

3 F

Standing (hip-width apart), pick up an object from the floor and place it in front of you at the table

- Variation:
  Starting position: narrow/ wide stance, tandem stance
  Object: place at different heights (e.g., on a shelf)

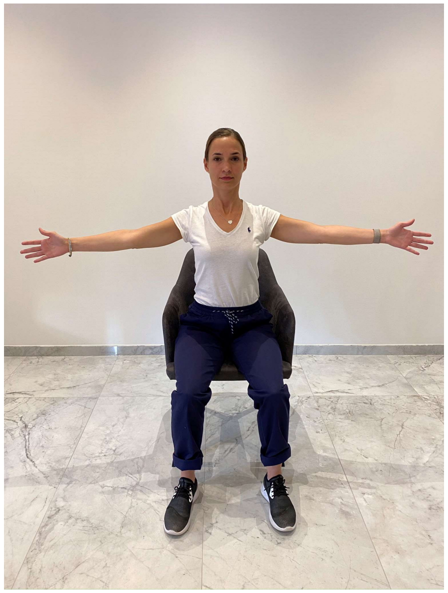

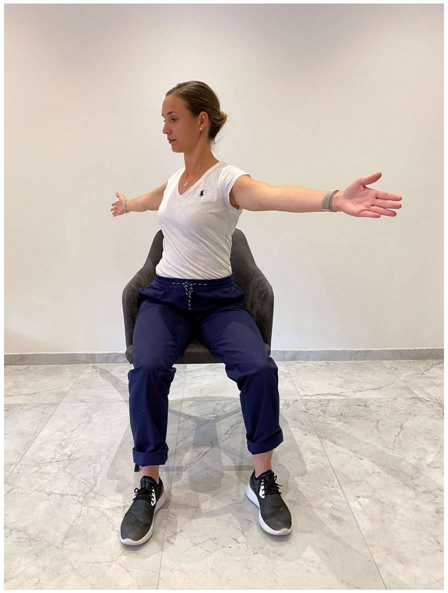

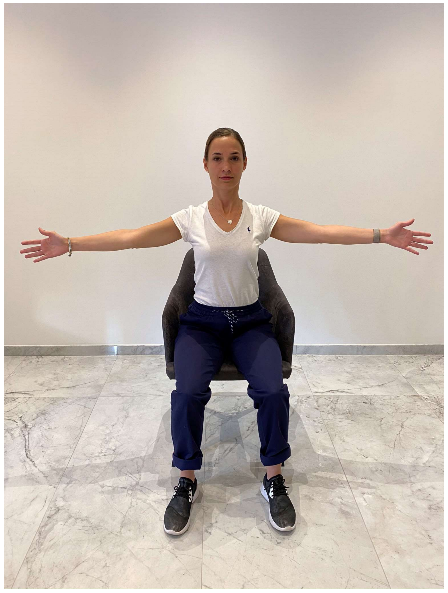

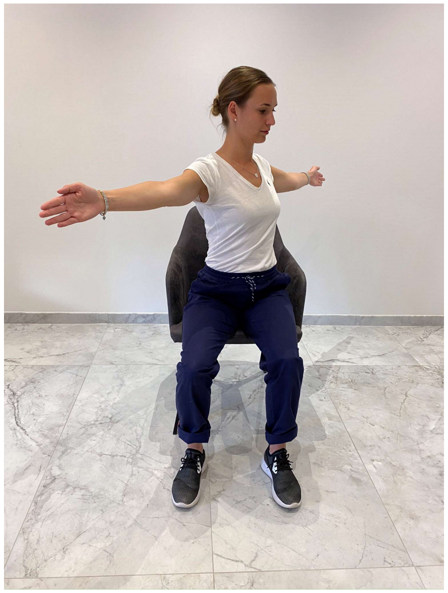

4 A

Sitting: rotate your torso to the right/left with your arms stretched out to the side

...and back to the center

- Variation: sitting without the feet touching the floor
- Possible FC: look back over the left/right shoulder, grab an object behind you, ...

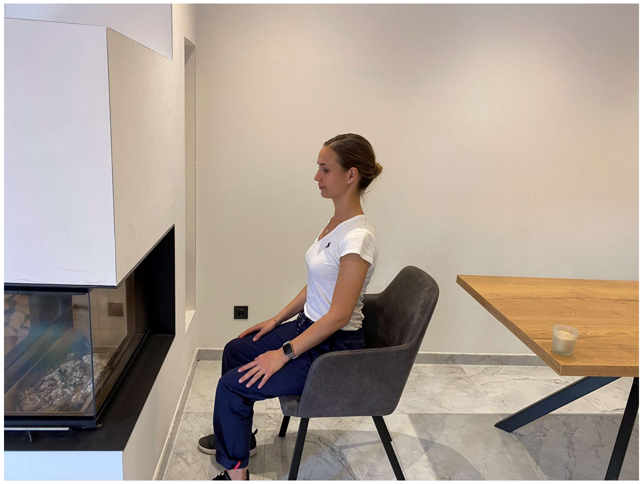 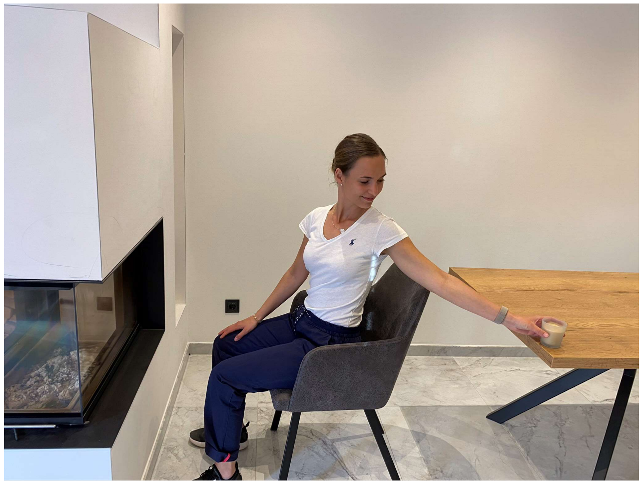 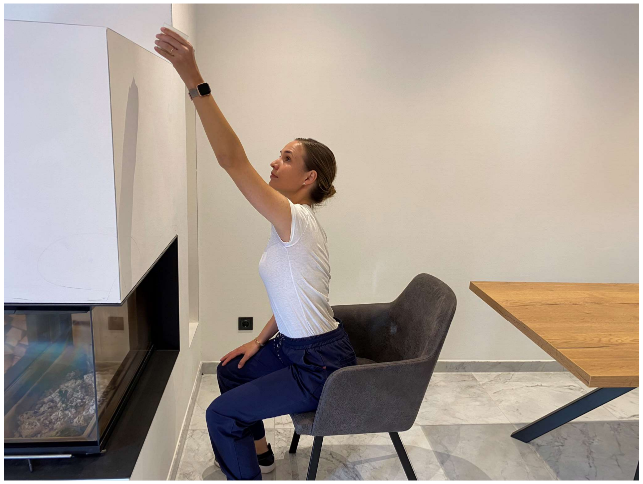

4
B

Sitting: rotate your torso to grasp an object behind you

...and place the item in front of you at the top of the shelf; repeat several items.

- Variation: sitting without the feet touching the floor, different heights: e.g. placing an object in front of you or on the floor

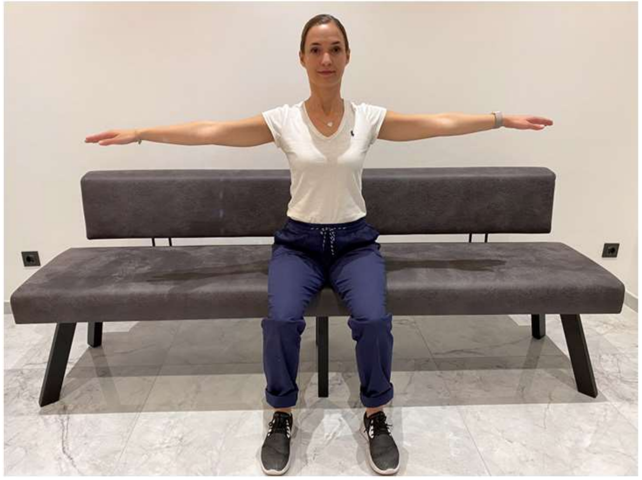 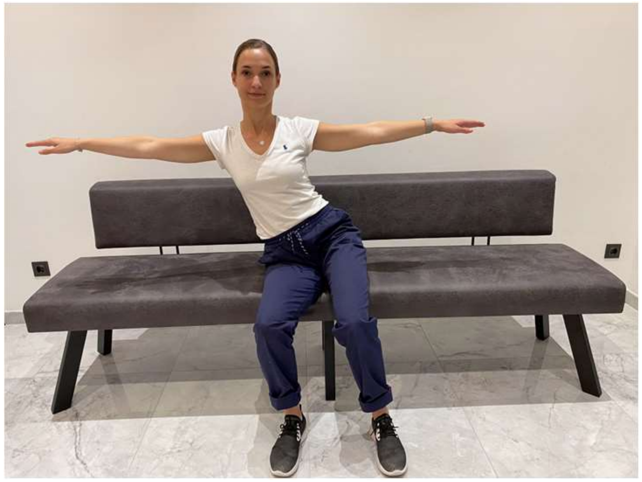 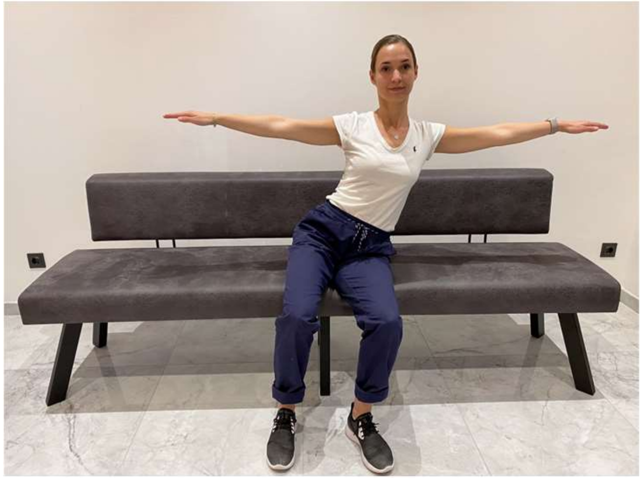

5
A

Sitting: lean as far as possible to the right/left side with arms stretched out

...and back to the center

- Variation: sitting without the feet touching the floor
- Possible FC: grab an object, ...

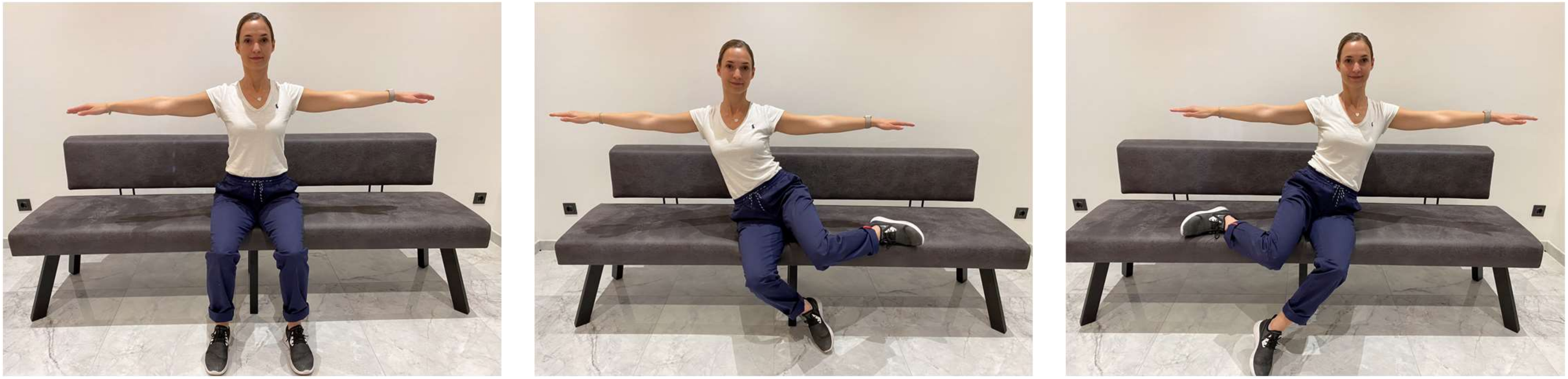

5 B

Sitting: lean as far as possible to the right/left side with arms stretched out & lift 1 leg (onto the bed)

...and back to the center

- Variation: sitting without the feet touching the floor
- Possible FC: lie down in bed, sit comfortably on the couch, ...

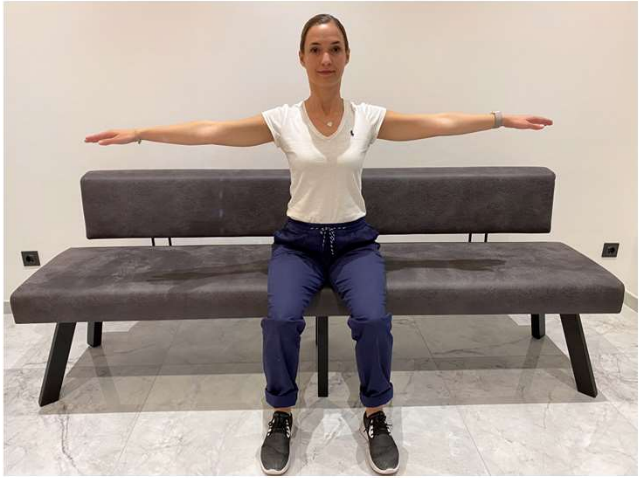
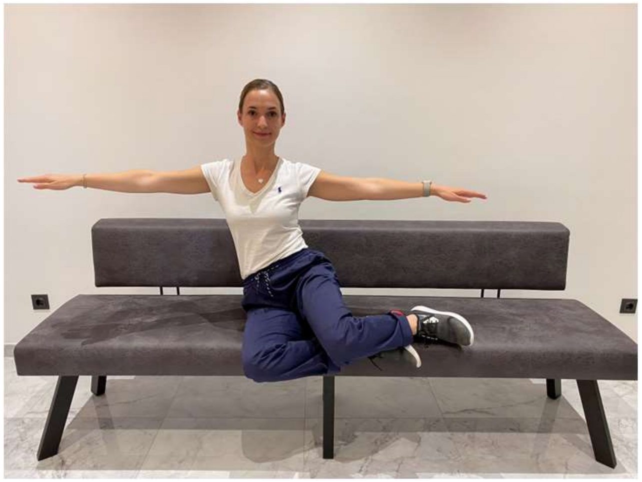
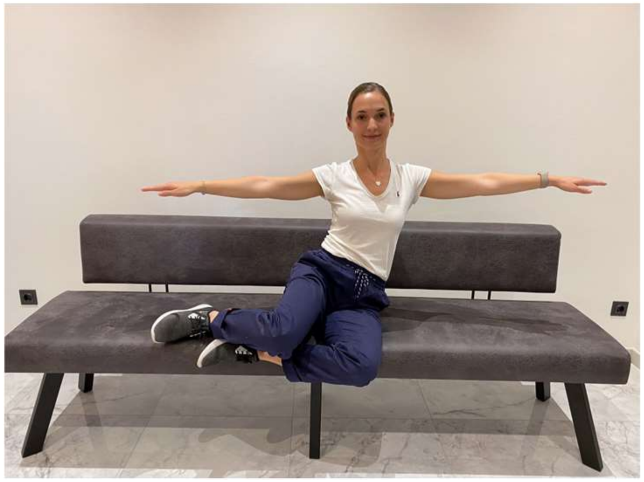

5
C

Sitting: lean as far as possible to the right/ left side with arms stretched out & lift both legs (onto the bed)

...and back to the center

- Variation: sitting without the feet touching the floor
- Possible FC: lie down in bed, sit comfortably on the couch, ...

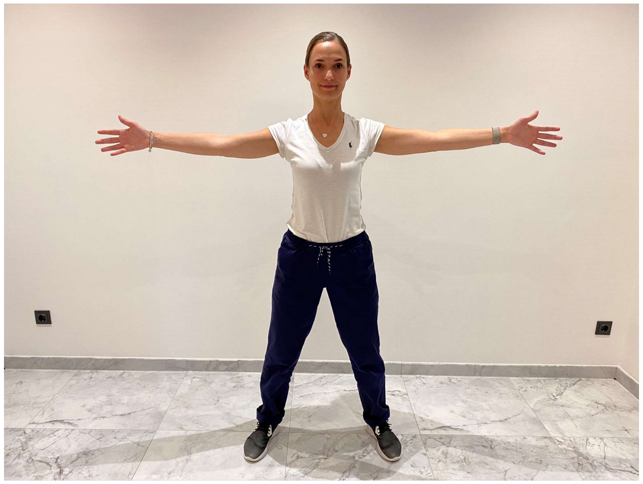

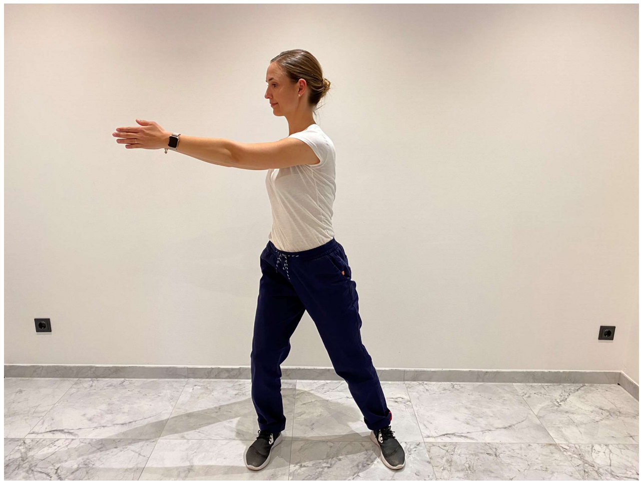

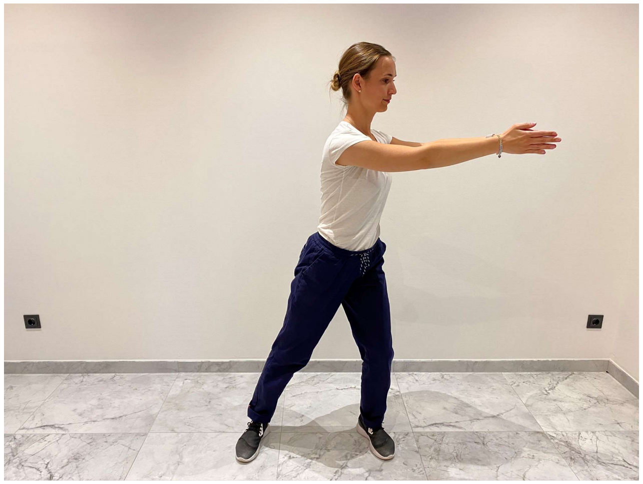

6 A

Wide stance: shift your weight all the way to the right/left and clap your hands

...and back to the center, rapid alternations

- Possible FC: hanging laundry on the washing line, taking items from a shelf

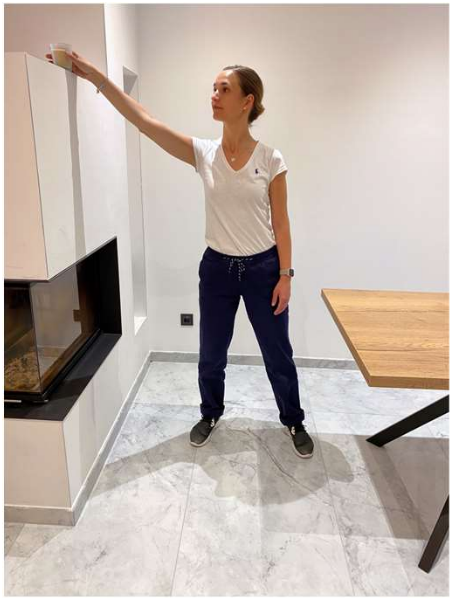

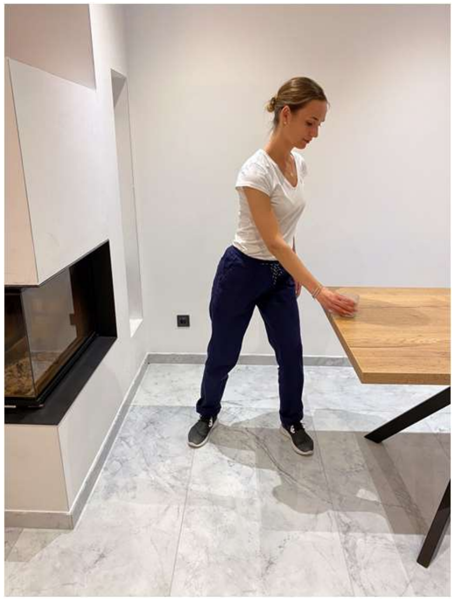

6 B

Wide stance: take objects from the right/left side

...and place them front of you on the table

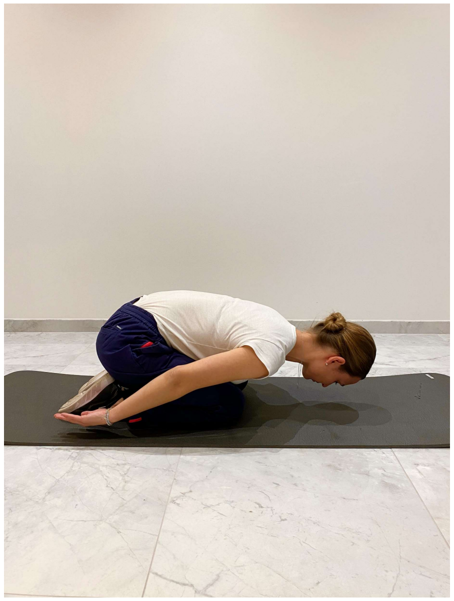

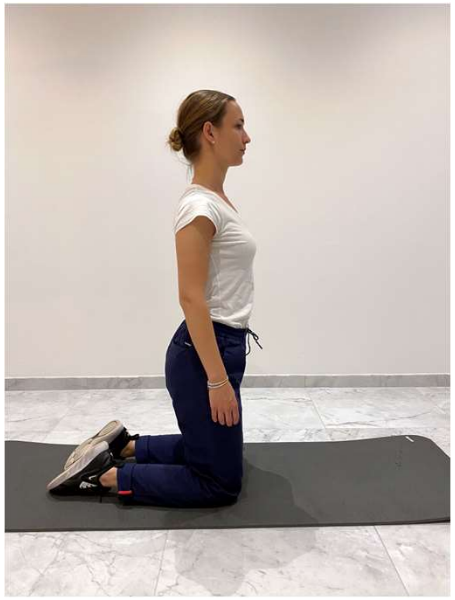

7 A

Heel sit – kneeling position

...and back to the heel sit.

- Possible FC: sit-to-stand

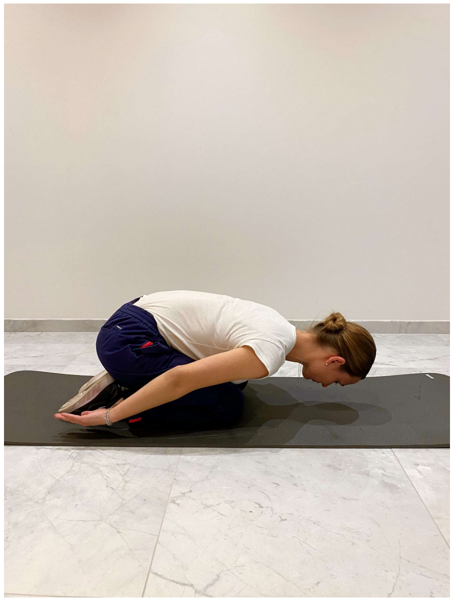

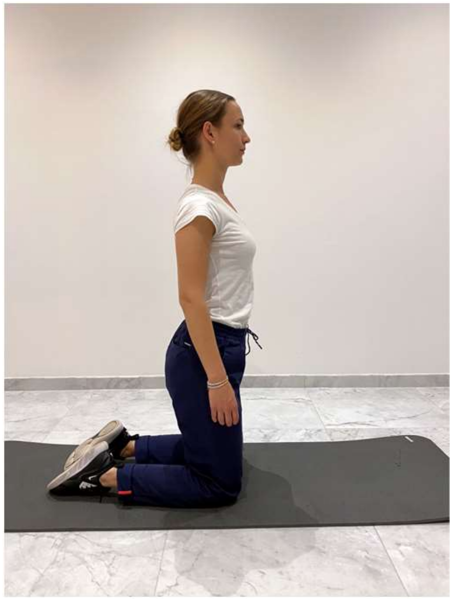

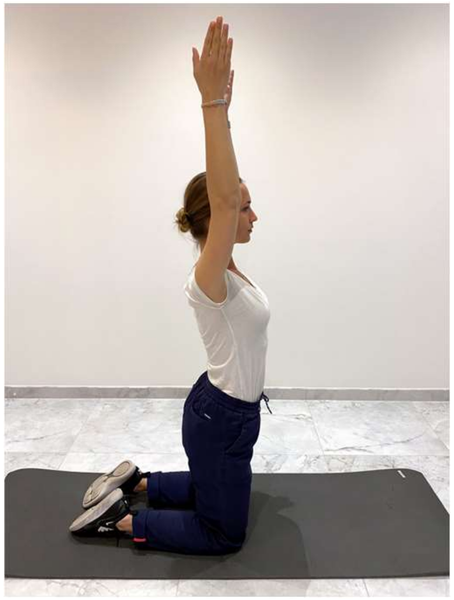

7 B

Heel sit – kneeling position & arms up

...and back to the heel sit.

- Possible FC: sit-to-stand

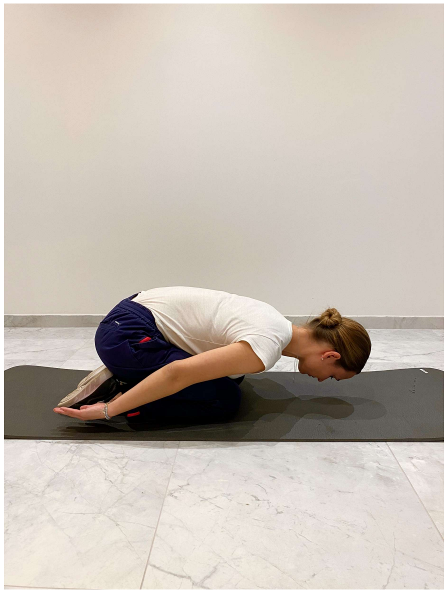
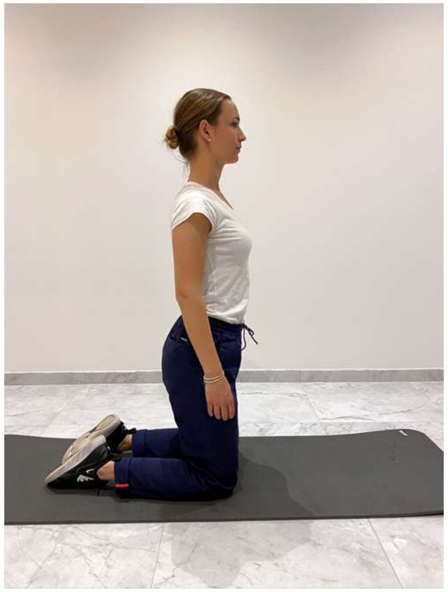
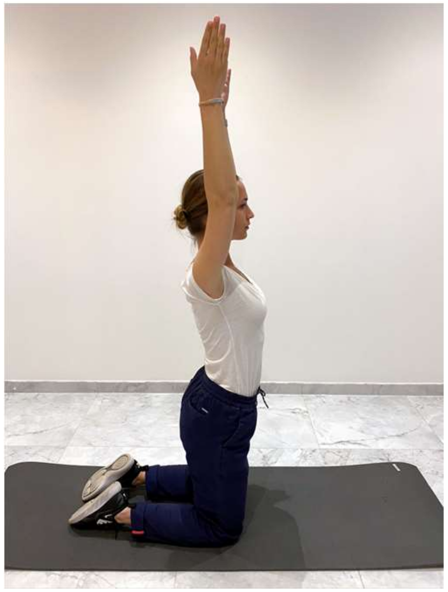
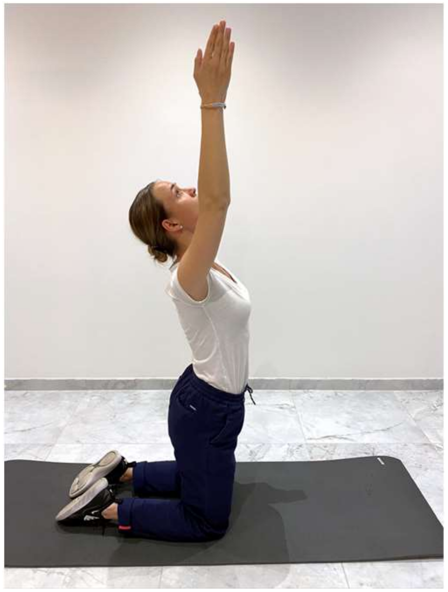

7 C

Heel sit – kneeling position, arms up & look at your hands

...and back to the heel sit in reverse order.

- Possible FC: sit-to-stand

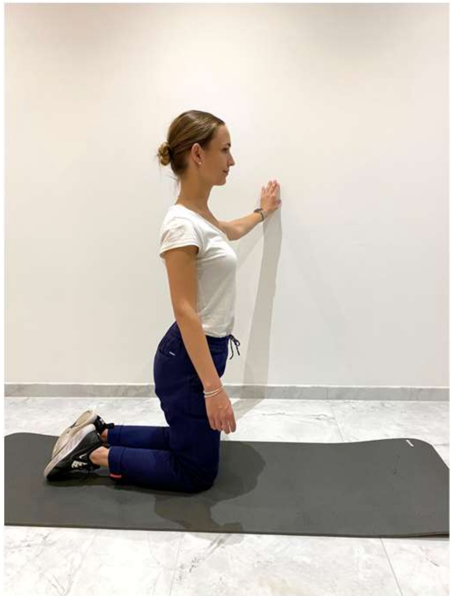

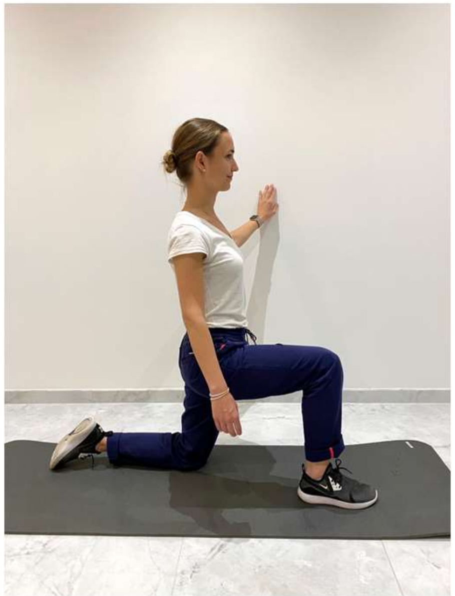

8 A

Holding on to the wall: kneeling position – move left/right leg forward

...and move leg backwards to kneeling position.

- Possible FC: getting up from the floor

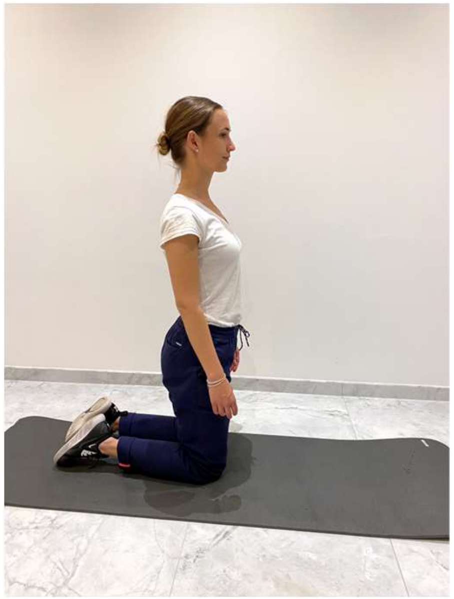

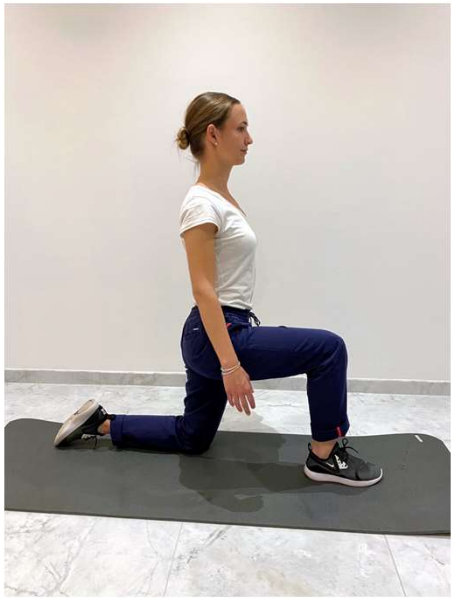

8 B

Without holding on: kneeling position – move left/right leg forward

...and move leg backwards to kneeling position

- Possible FC: getting up from the floor

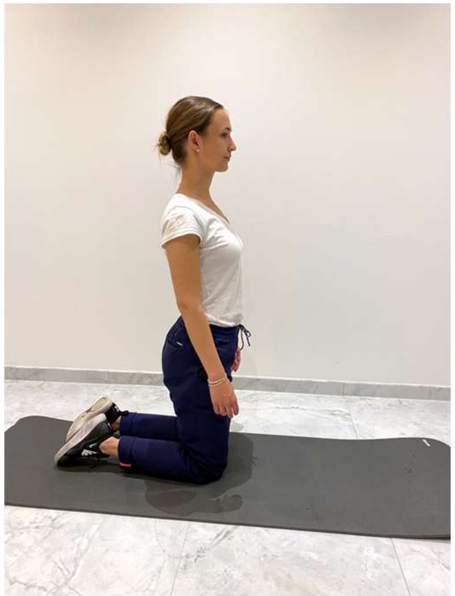

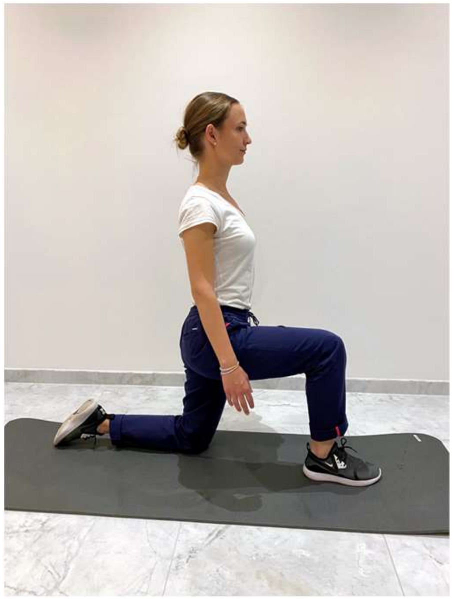

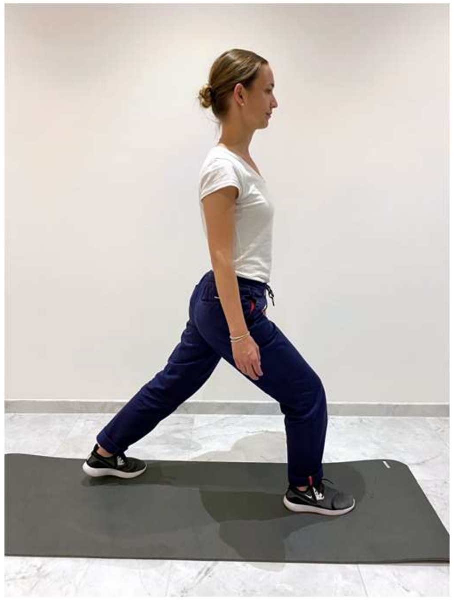

8 C

Kneeling position – move left/right leg forward – stand up

...back to the kneeling position in reverse order

- Possible FC: getting up from the floor

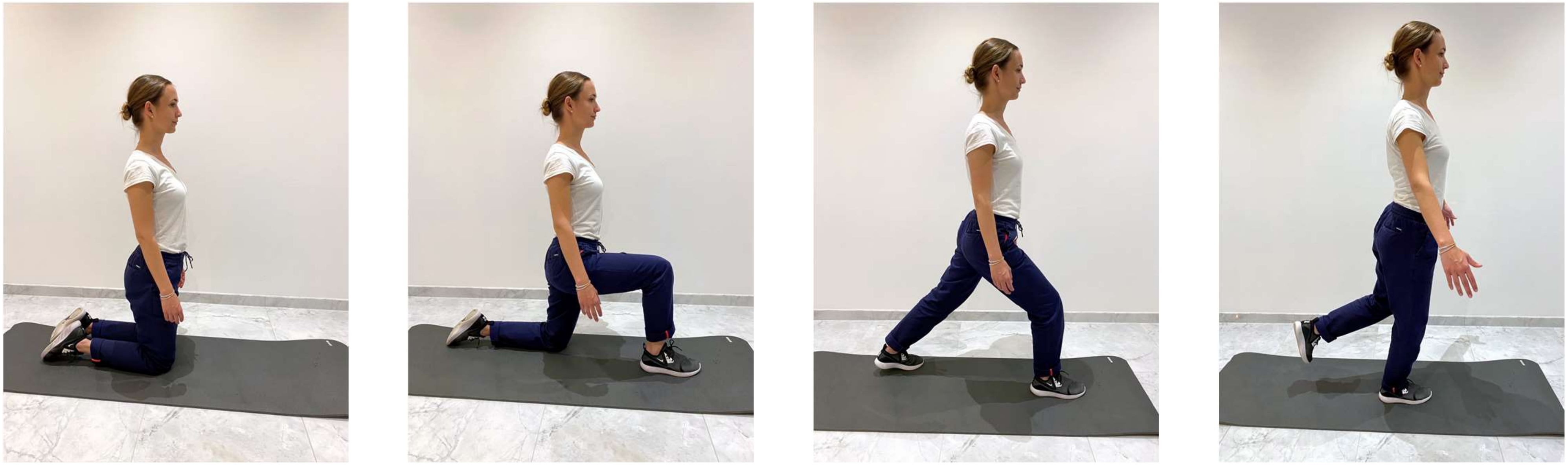

8
D

Kneeling position – move left/right leg forward – stand up – lift your foot

...back to the kneeling position in reverse order

- Possible FC: getting up from the floor

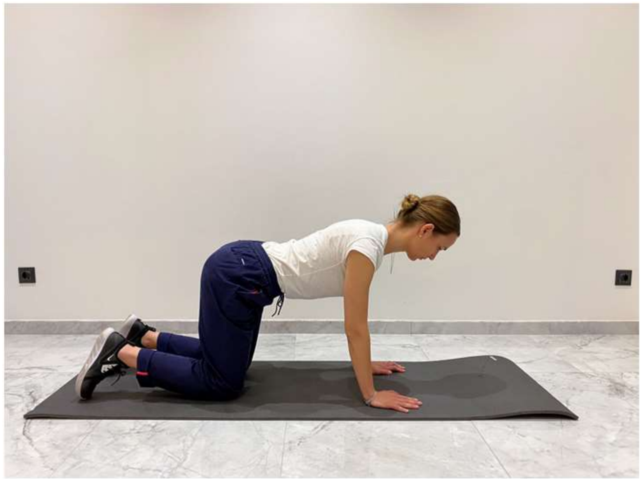

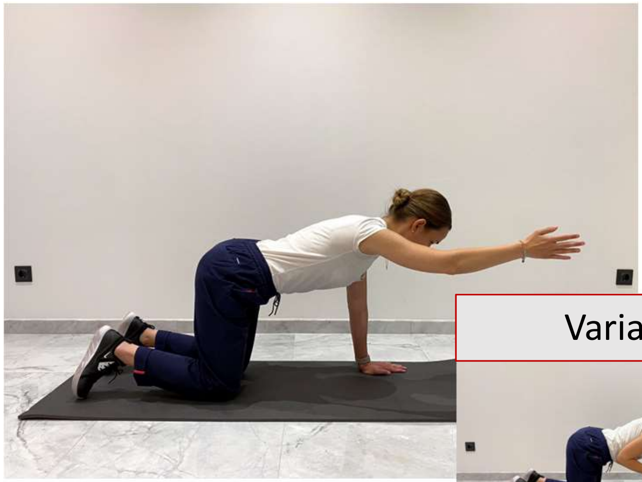

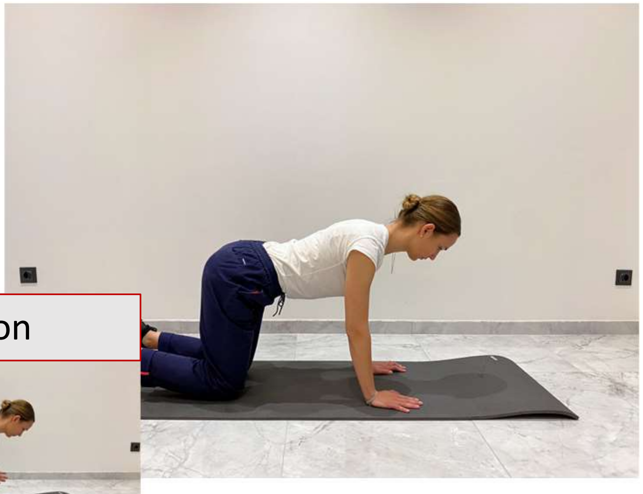

Variation

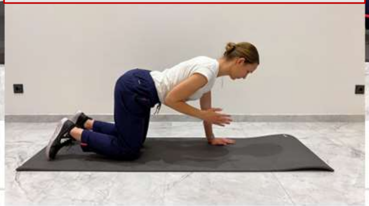

9 A

Quadruped position: alternately extend the left and right leg

- Variation: pull your elbows towards your stomach, before putting them down again
- Possible FC: switch off the alarm clock while lying in bed; preliminary exercise for getting up from the floor, climbing stairs and walking

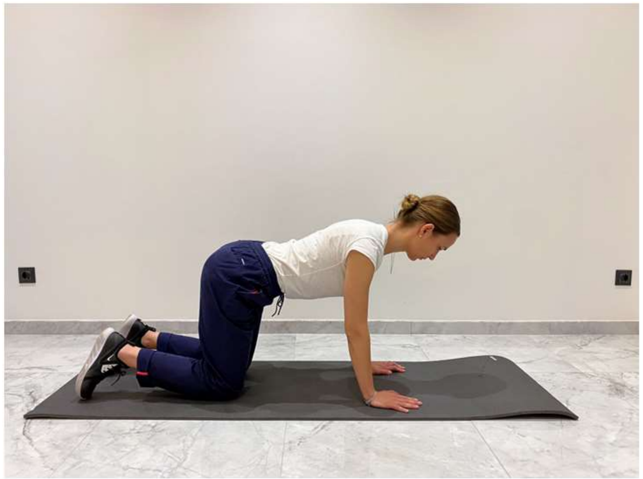

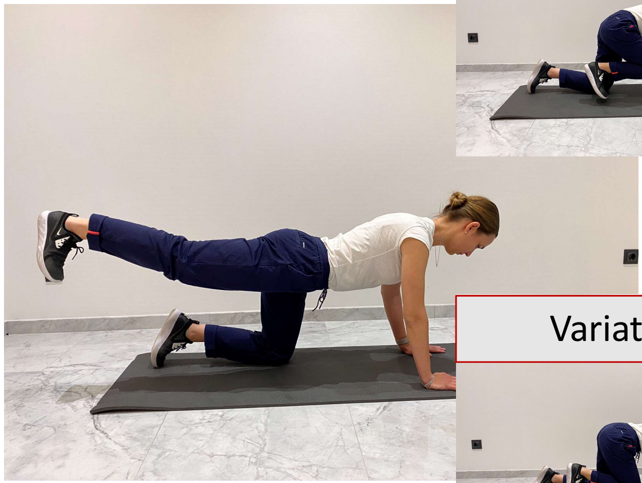

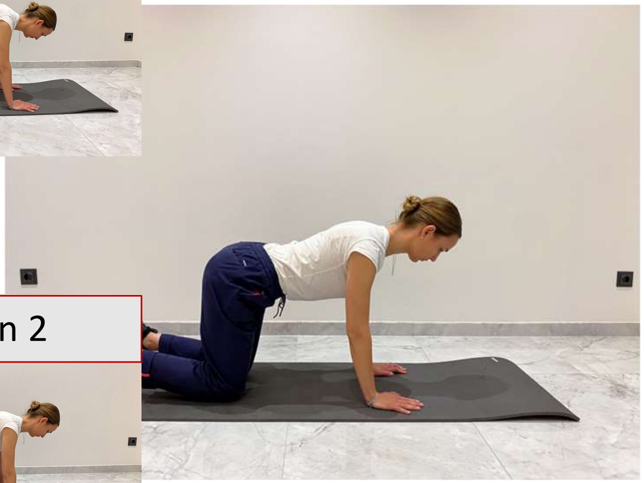

Variation 1

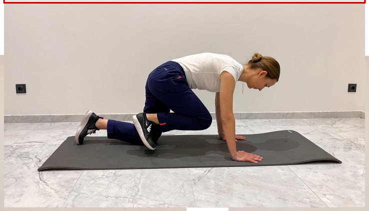

Variation 2

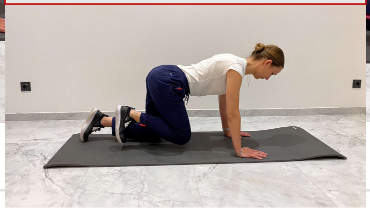

9 B

Quadruped position: alternately extend the left and right leg

- Variation: pull your knees towards your stomach before putting them down again, pull your knees towards your stomach & bend your spine before putting them down again
- Possible FC: getting out of bed lying in a prone position; preliminary exercise for getting up from the floor, climbing stairs and walking

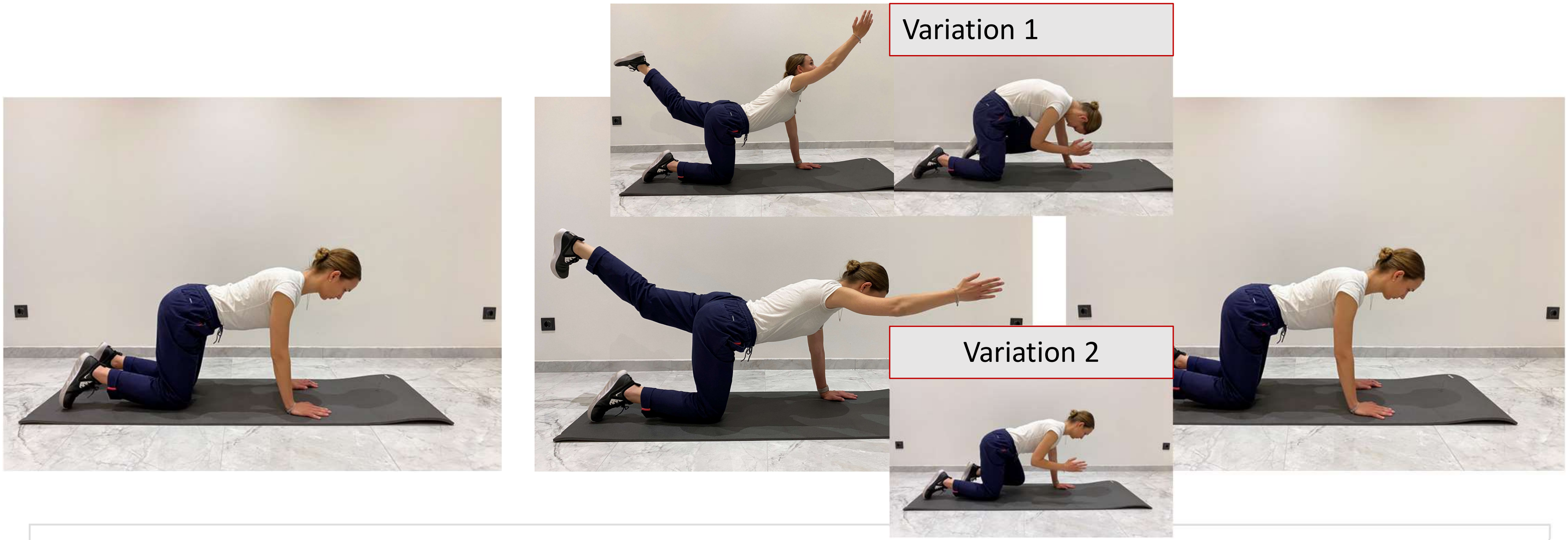

9 C

Quadruped position: lifting left arm and right leg or right arm and left leg at the same time

- Variation:
  - Pull your elbows/knees towards your stomach before putting them down again.
  - When stretching out, stretch your spine (cow position), when pulling in, bend the spine (cat position).
- Possible FC: preliminary exercise for getting up from the floor, climbing stairs and walking.

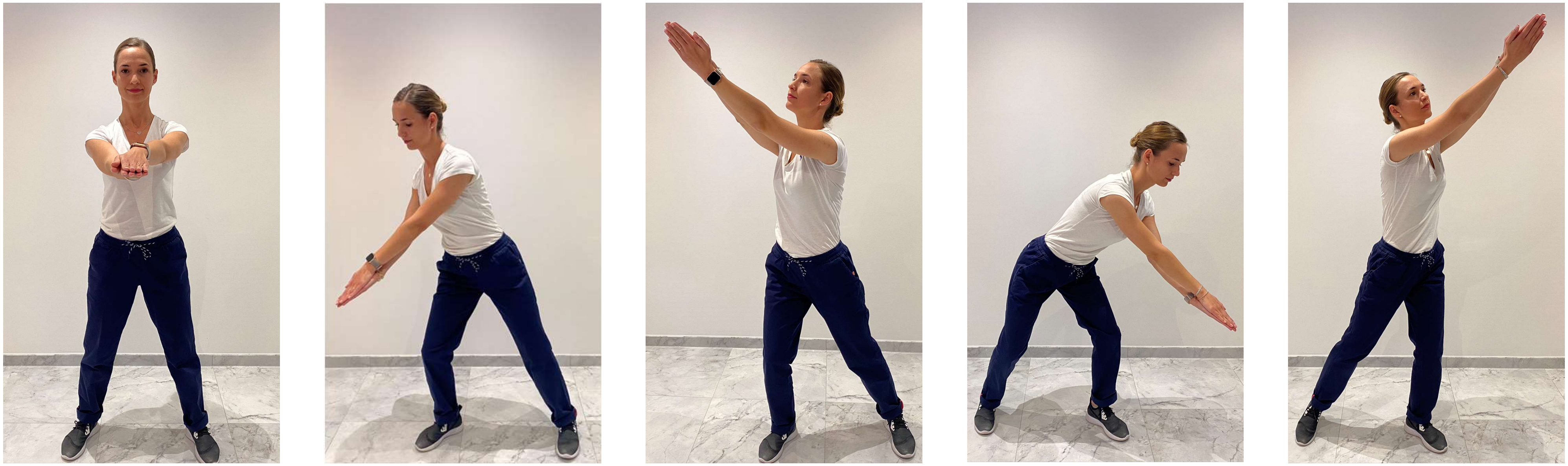

10 A

Standing: describe a large horizontal figure 8 with both arms, shifting your weight to the right/left

- Variation: starting positions (e.g., wide/narrow stance, etc.)
- Possible FC: cleaning windows, ...

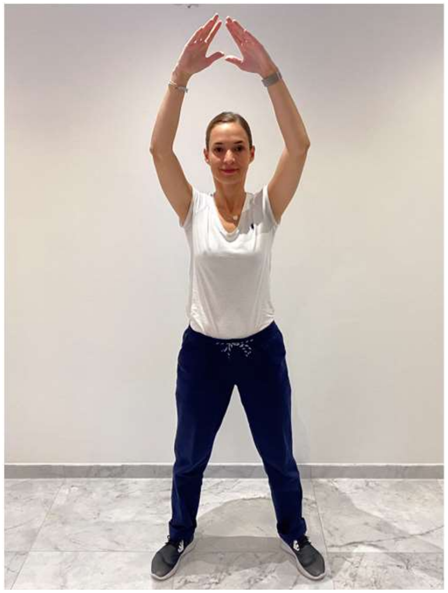
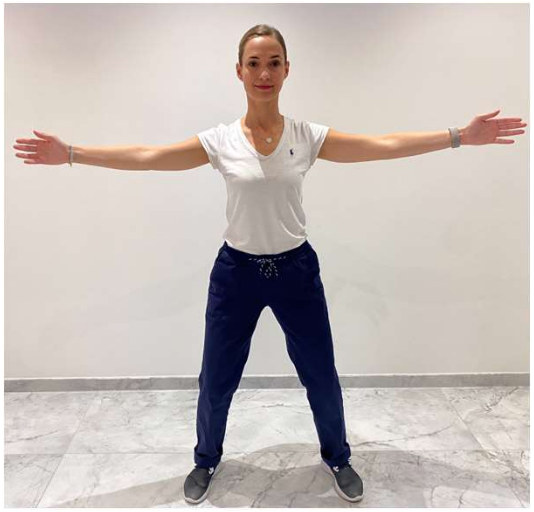
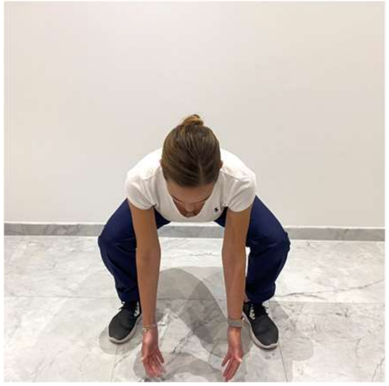
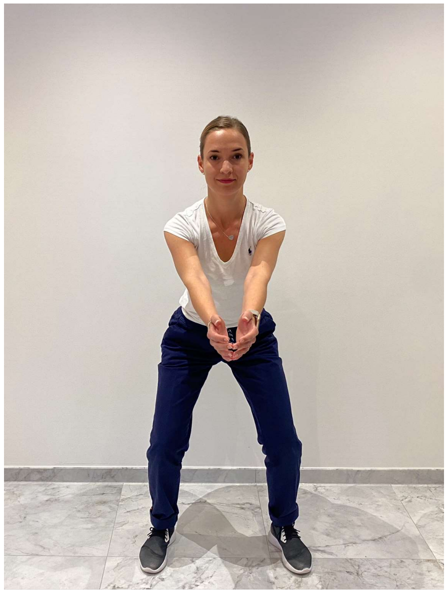

10 B

Standing: describe a large circle with both arms, stretch your fingertips down to the floor and all the way up

- Variation: starting positions (e.g. wide/narrow stance, etc.)
- Possible FC: pick up objects (e.g. laundry) from the floor, loading your car, ...

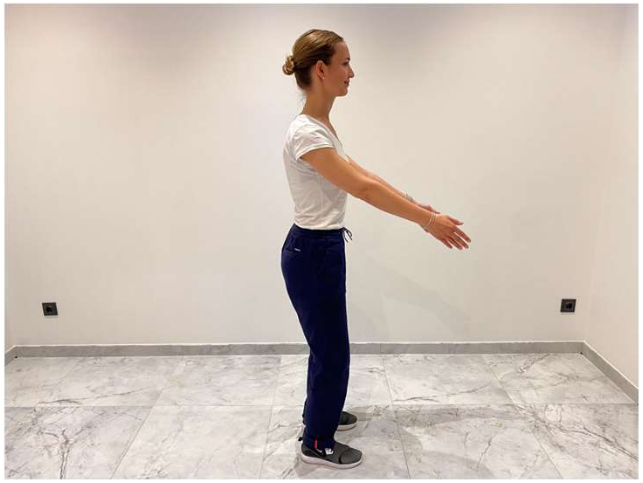

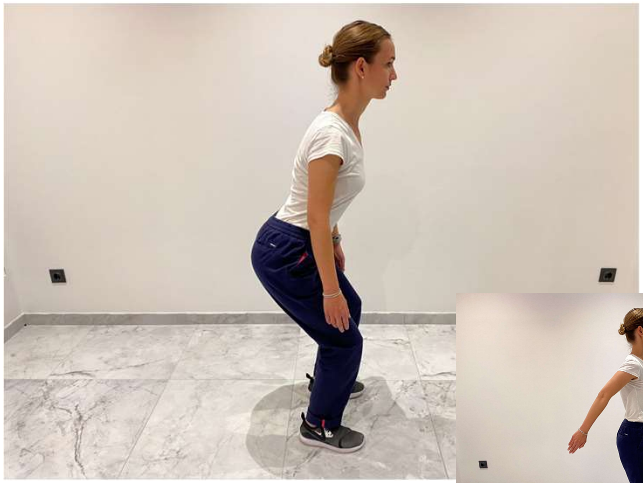

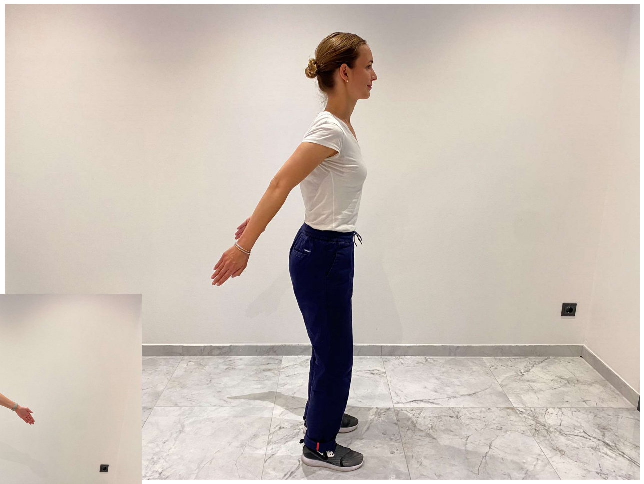

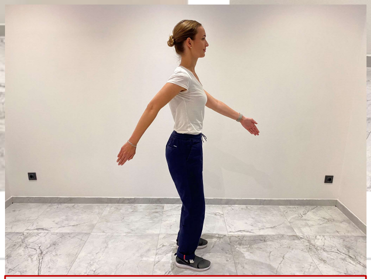

Variation

11 A

Wide stance: bounce (bend your knees) while swinging your arms forward & backwards

- Variation: alternating arm swing
- Possible FC: this exercise serves as a preliminary exercise for walking, dancing, cross-country skiing, etc.

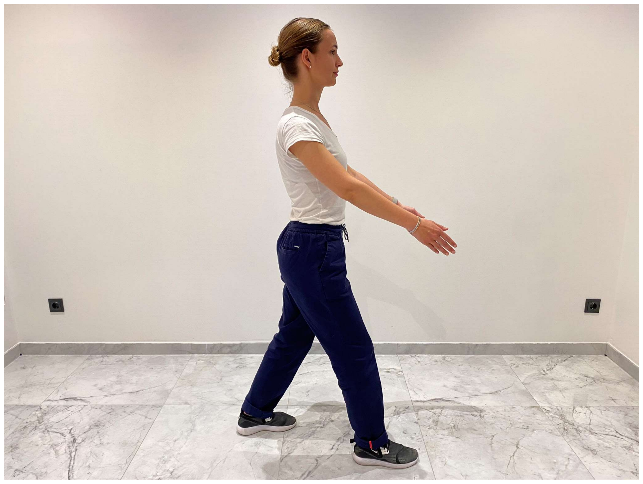

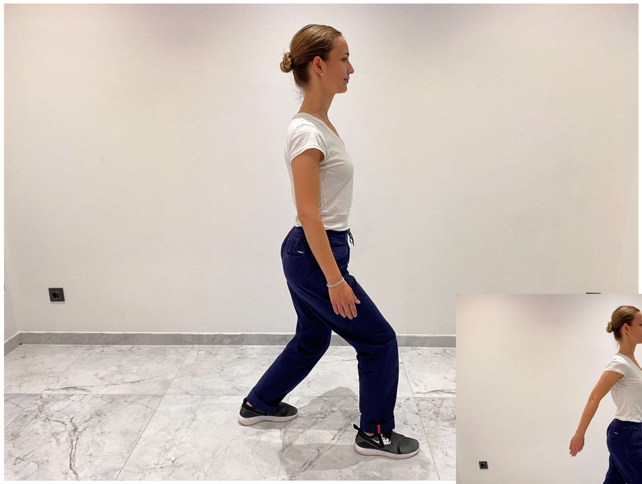

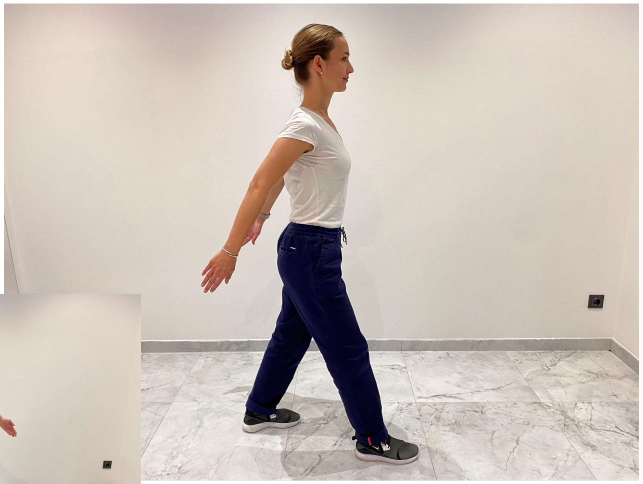

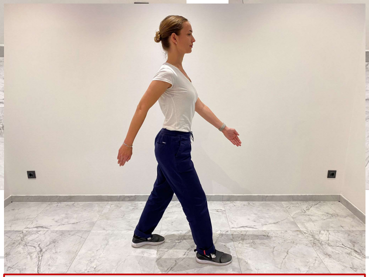

Variation

11
B

Staggered stance: bounce (bend your knees) while swinging your arms forward & backwards

- Variation: alternating arm swing
- Possible FC: this exercise serves as a preliminary exercise for walking, dancing, cross-country skiing, etc.

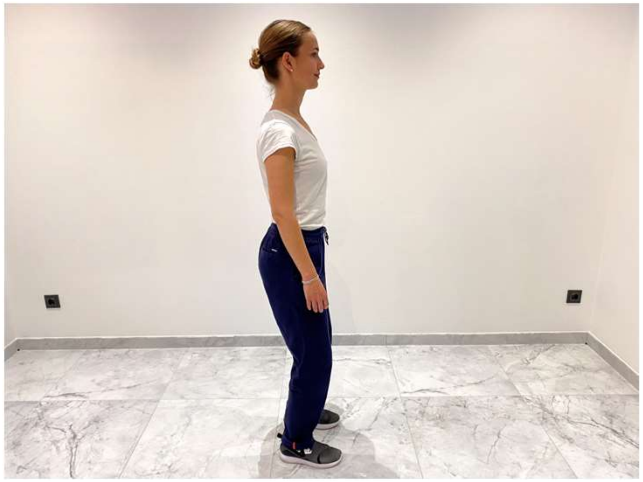

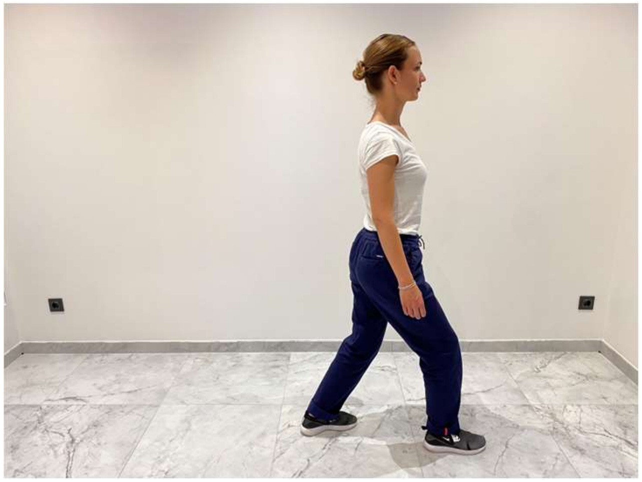

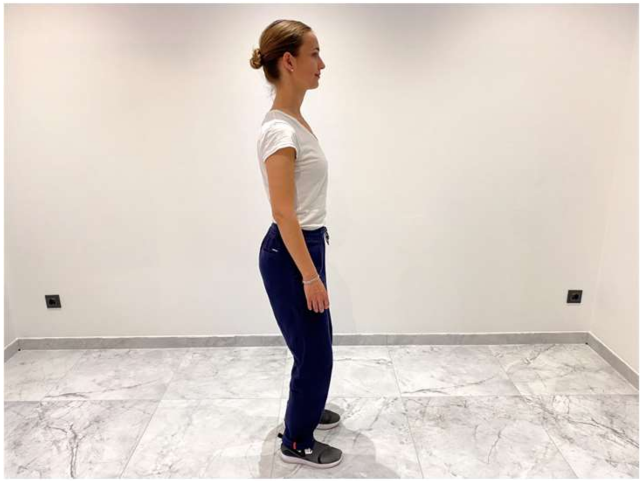

12 A

Steps: forward (with left/right foot)

Stand with your feet hip width apart – step forward – step back again (to hip width stance)

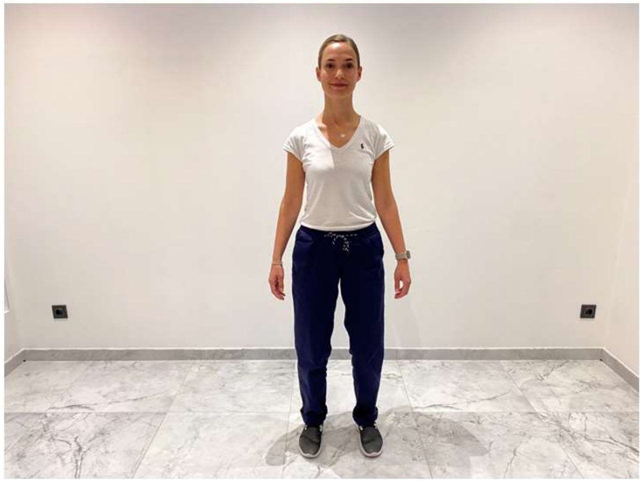
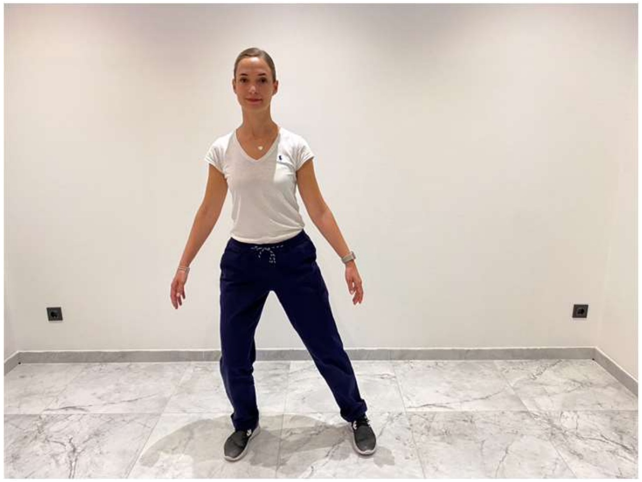
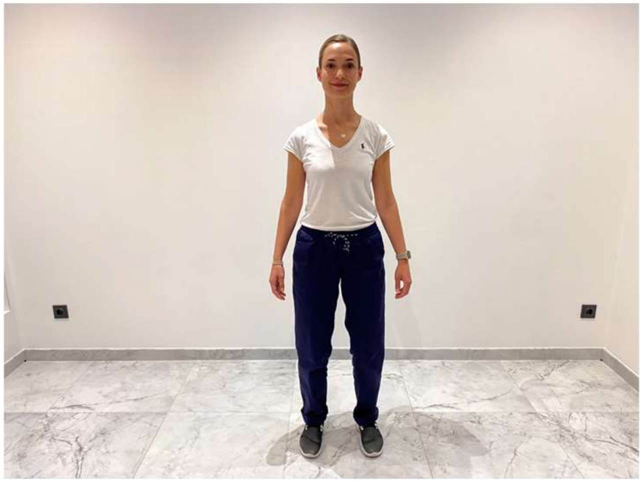

12 B

Side steps (with left/right foot)

Stand with your feet hip width apart – step

...with left foot to the left side or

...with right foot to the right side

...and back to the center (hip width stance)

- Possible FC: preliminary exercise for walking and protective steps, dancing, etc.

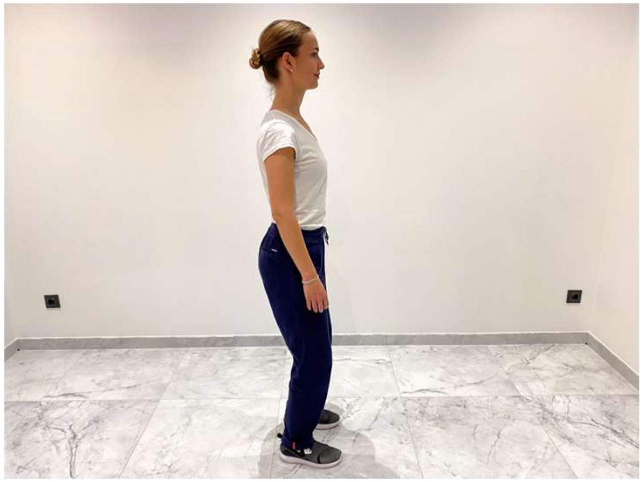

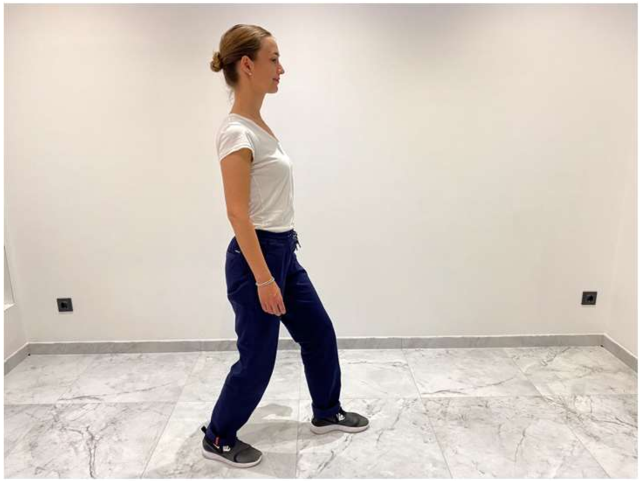

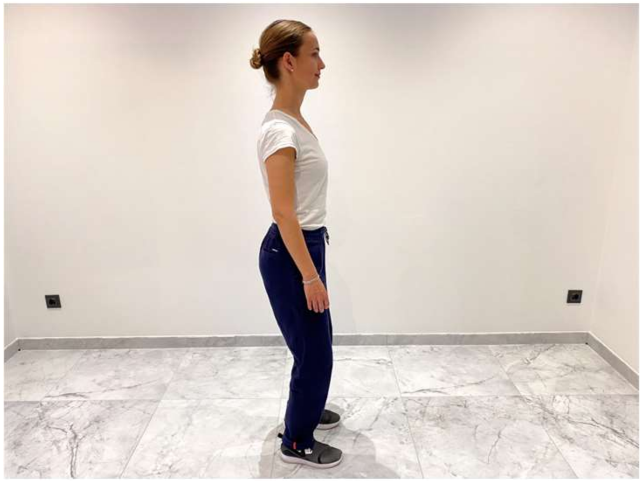

12 C

Steps: backwards (with left/right foot)

Stand with your feet hip width apart – step backwards – step back to starting position (hip width stance)

- Possible FC: preliminary exercise for walking and protective steps, dancing, etc.

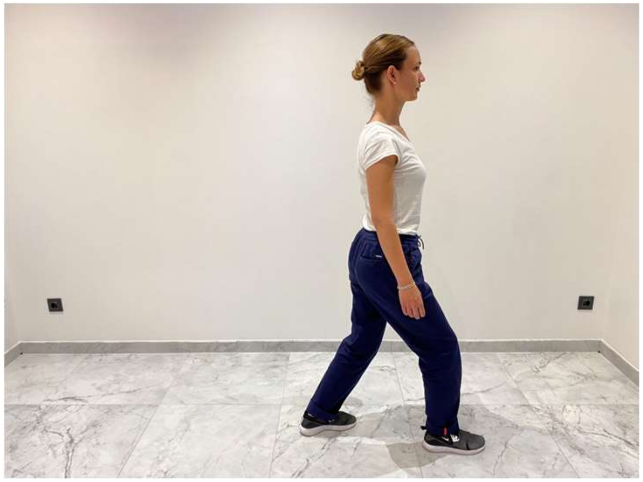 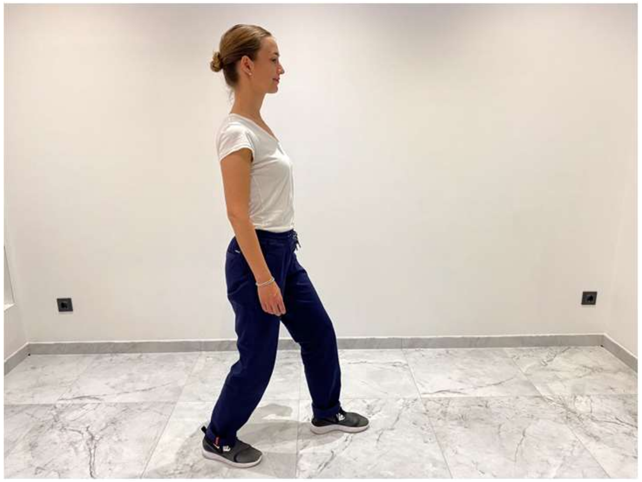 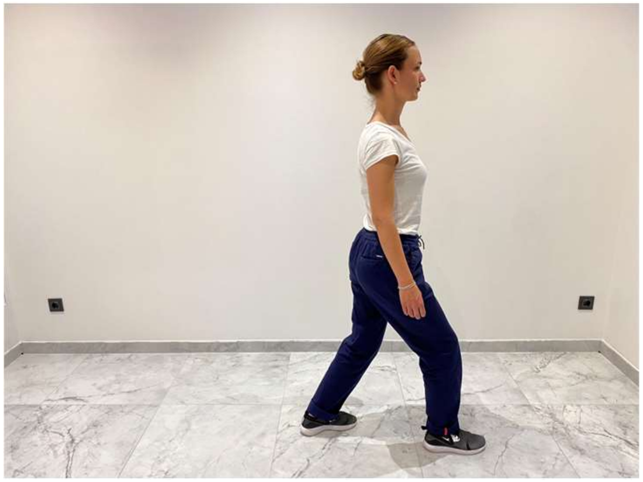

12 D

Step-combination: from front to back and back to front (with left/right foot)

- Possible FC: preliminary exercise for walking and protective steps, dancing, etc.
- Variation: other combinations of step exercises 12 A–C, e.g. step backwards – side step right – step forward – side step left, etc.

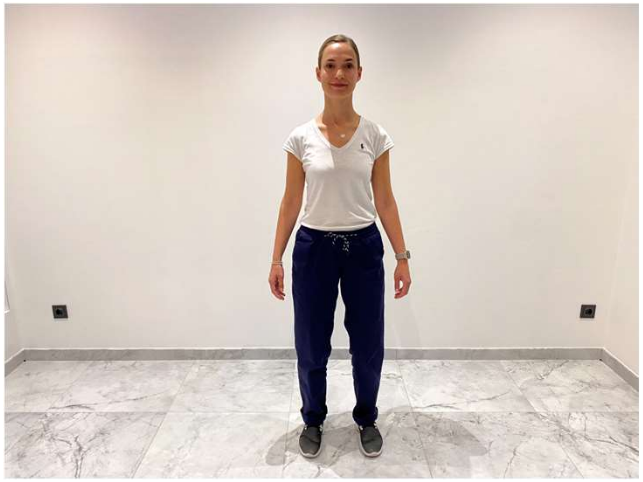

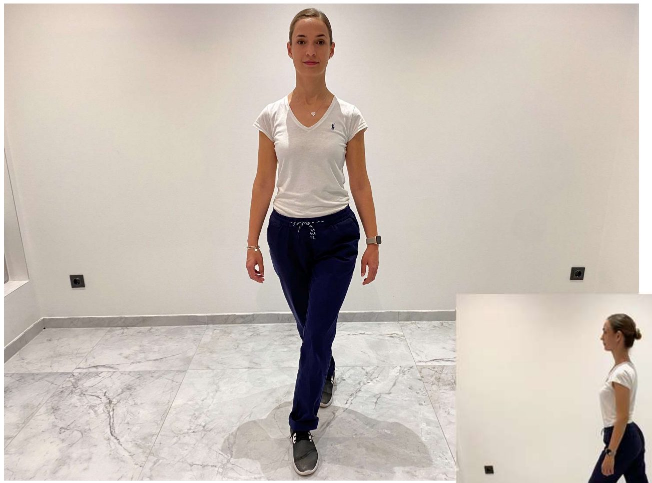

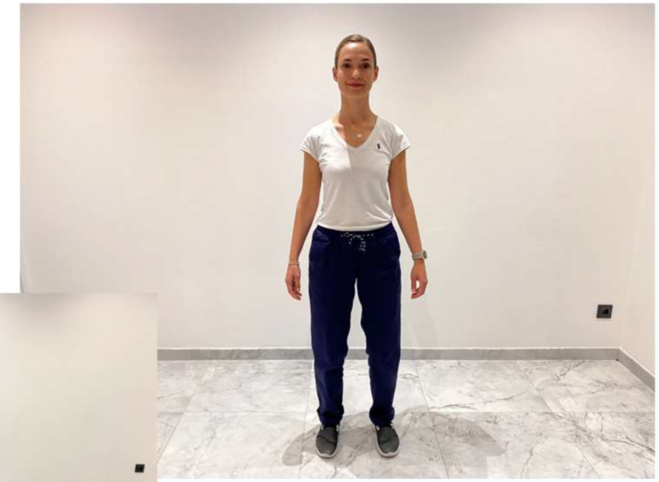

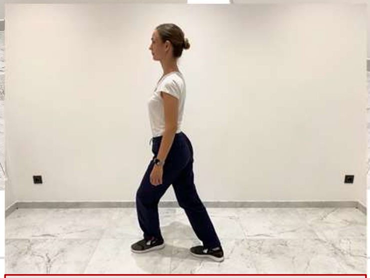

Lateral view

13 A

Cross steps: forward (with left/right foot)

...and back to starting position (hip width stance)

- Possible FC: preliminary exercise for walking and protective steps, dancing, etc.

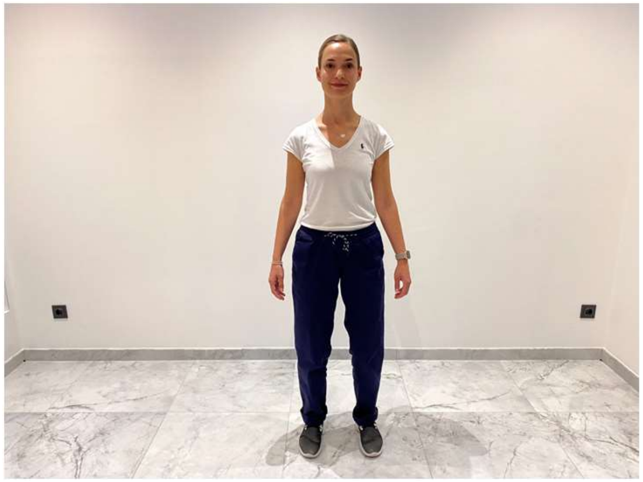

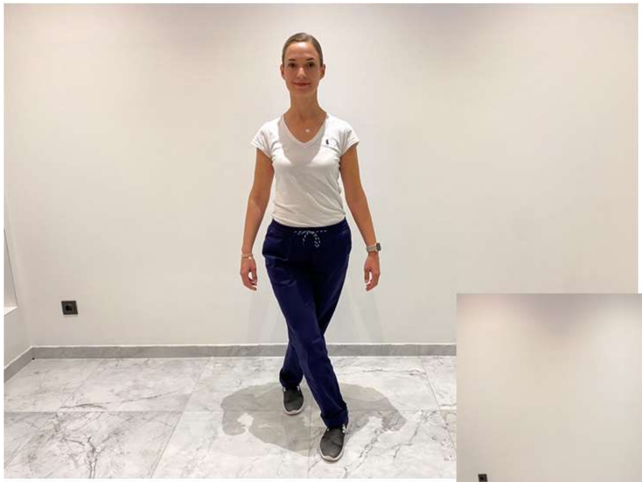

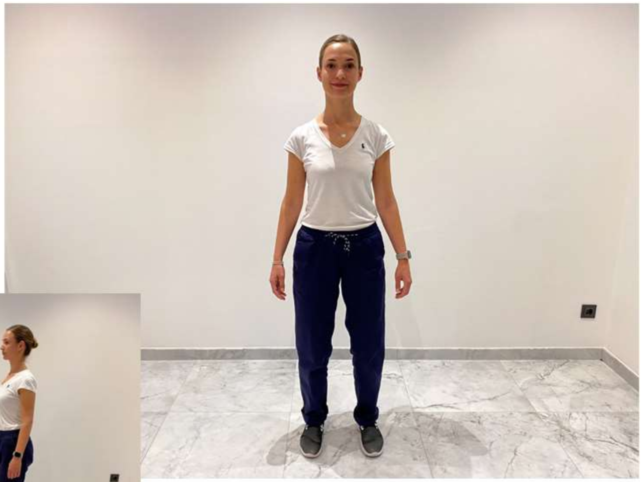

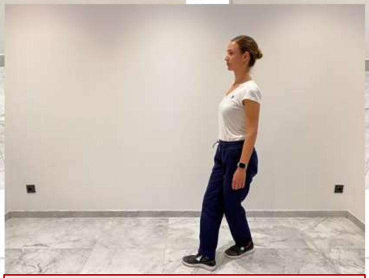

Lateral view

13 B

Cross steps: backwards (with left/right foot)

...and back to starting position (hip width stance)

- Possible FC: preliminary exercise for walking and protective steps, dancing, etc.

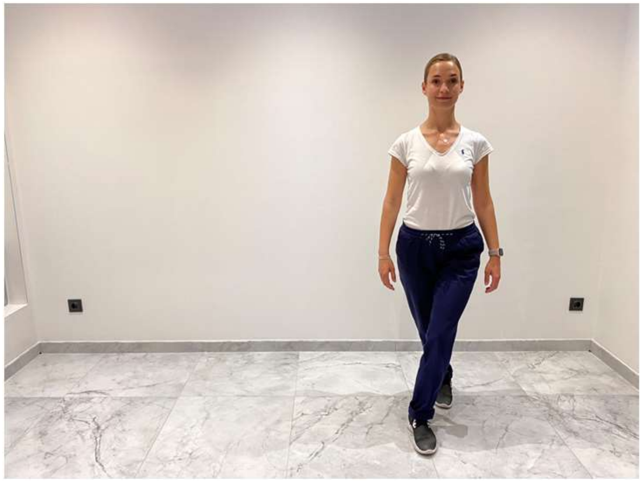

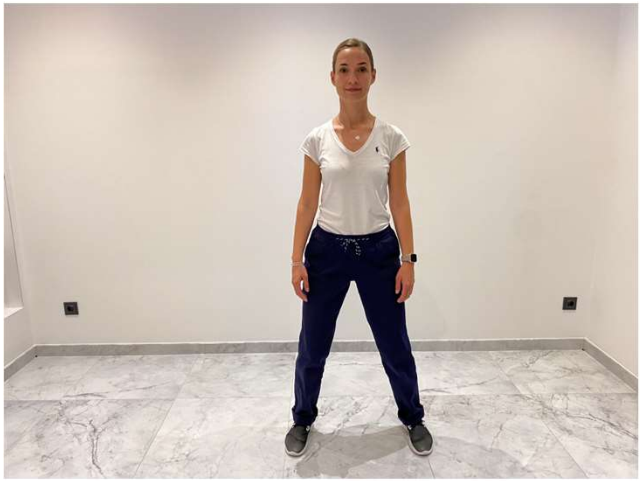

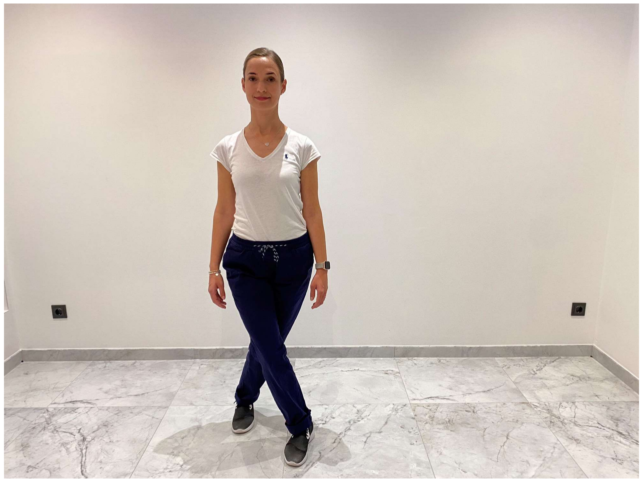

13 C

Step-combination: cross step front - side step - cross step back - repeat

... make several repetitions of step-combination: e. g. walk 10 meter

...to the left side or

...to the right side

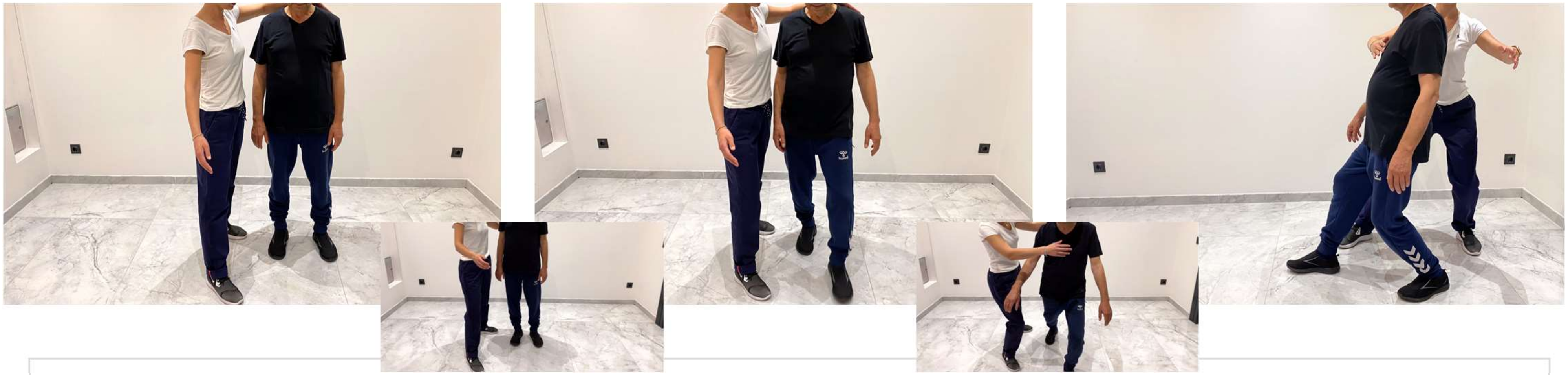

14
A

Supervised setting only: protective steps forward/backwards

Therapist pushes patient off balance

Patient makes reactive step forward/backwards

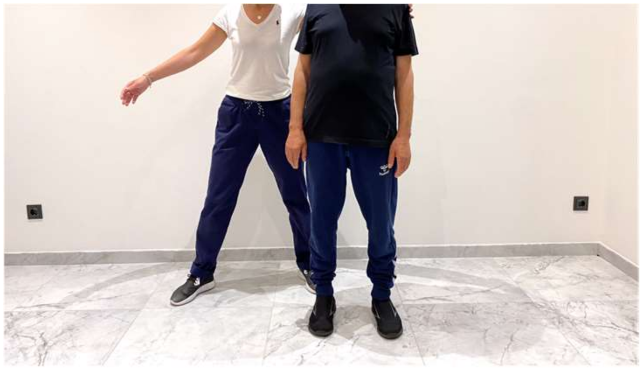 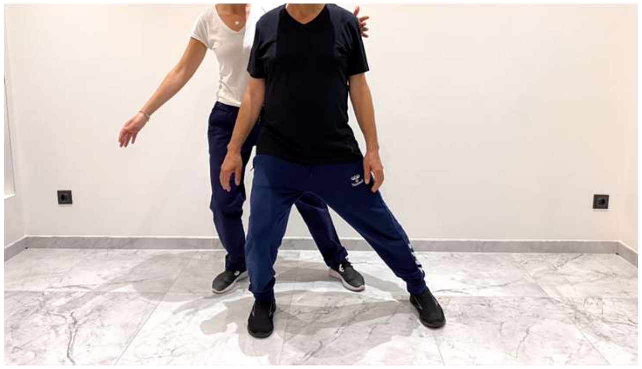

14 B

Supervised setting only: protective steps to the left/right side

Therapist pushes patient off balance

Patient makes reactive step to the left/right side

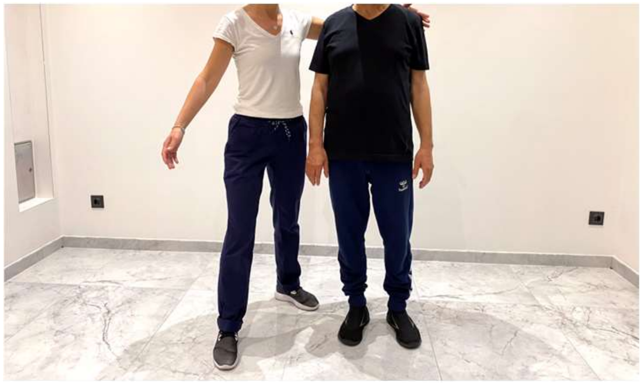

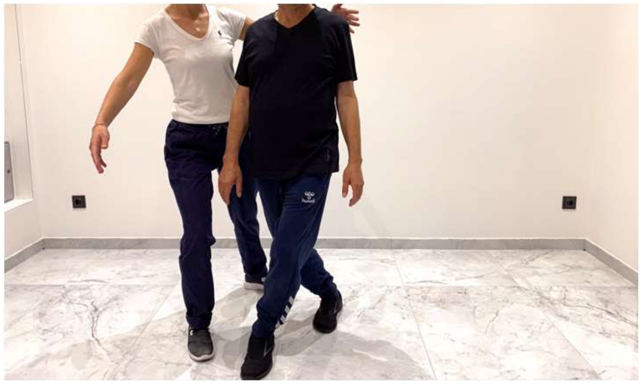

14 C

Supervised setting only: protective cross steps

Therapist pushes patient off balance

Patient makes reactive cross-step

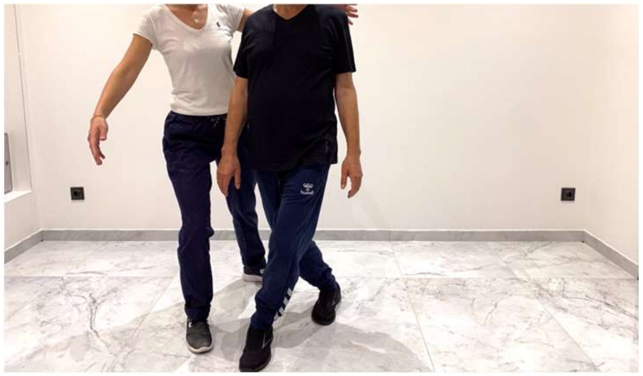

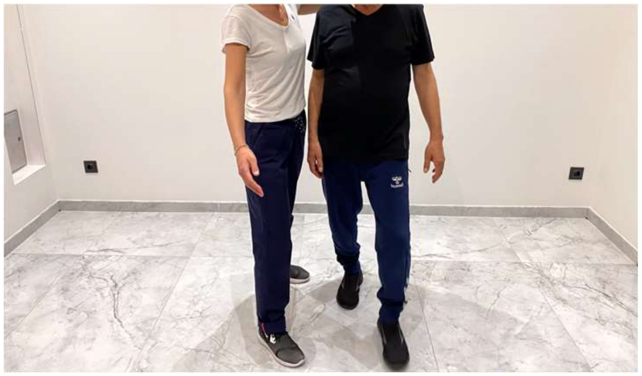

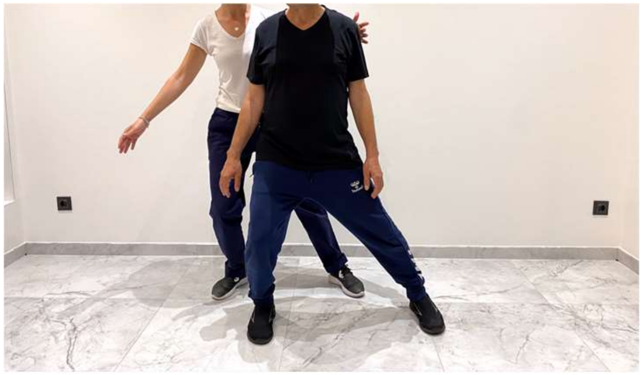

14 D

Supervised setting only: protective steps in all directions

Therapist pushes patient off balance

Patient makes reactive steps forward/backwards, left/right or cross-step

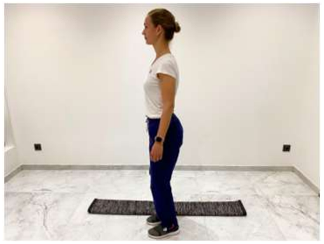
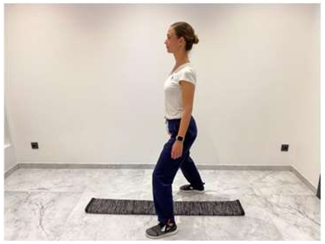
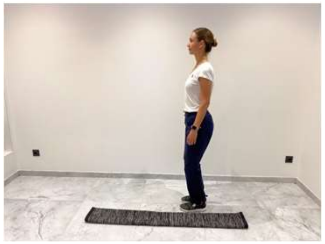
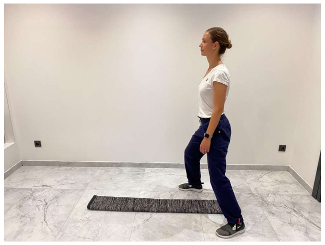
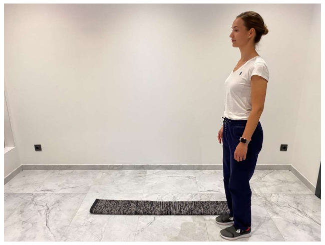
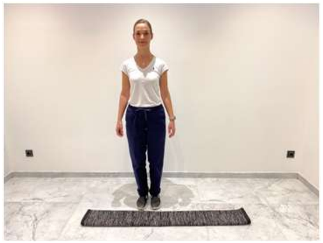
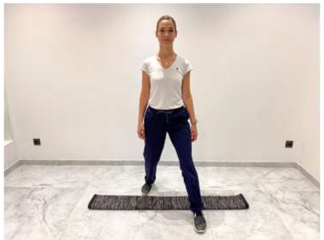
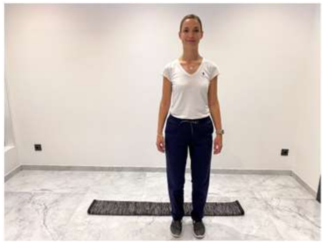
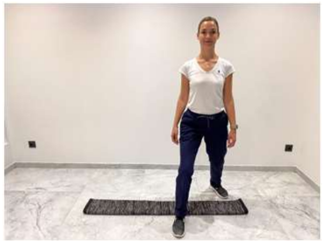
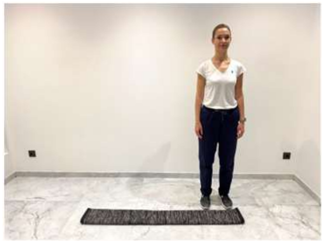

15 A

Side steps or steps forward/backwards with visual cue (e.g., line marking, speed ladder)

- Variation: diagonal steps (side steps with forward movement, steps forward/backwards with lateral movement) along the line
- Variation: different width of the line (to vary step width), different speed (slow/fast), variety of rhythms.

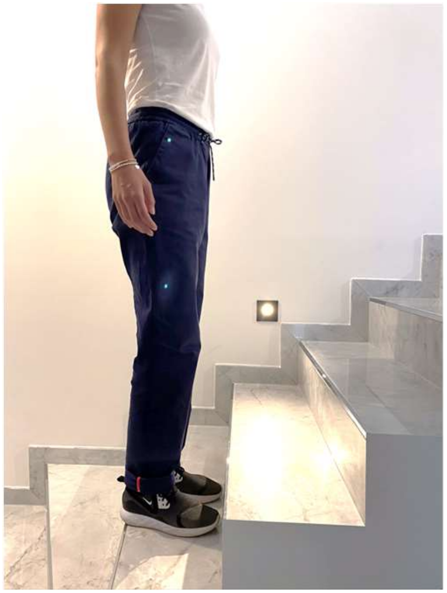
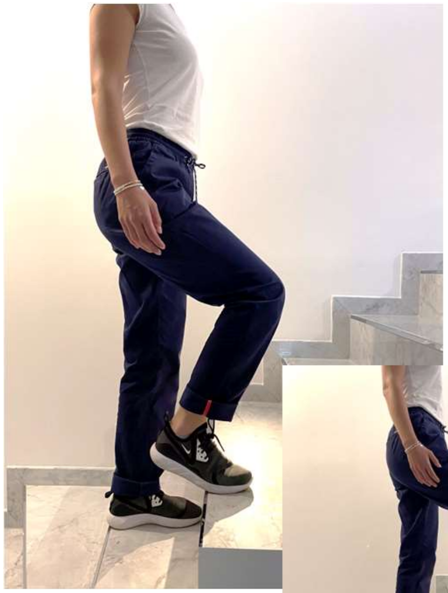
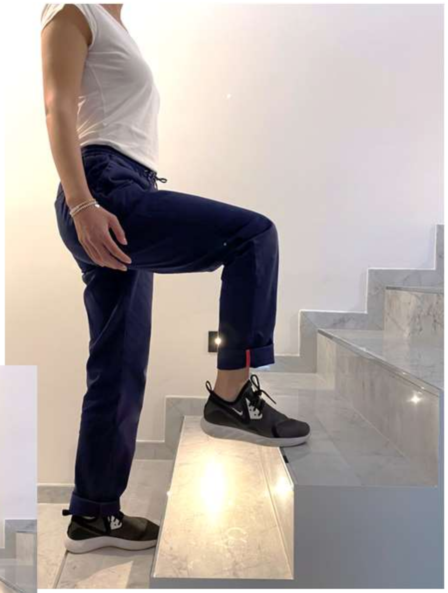
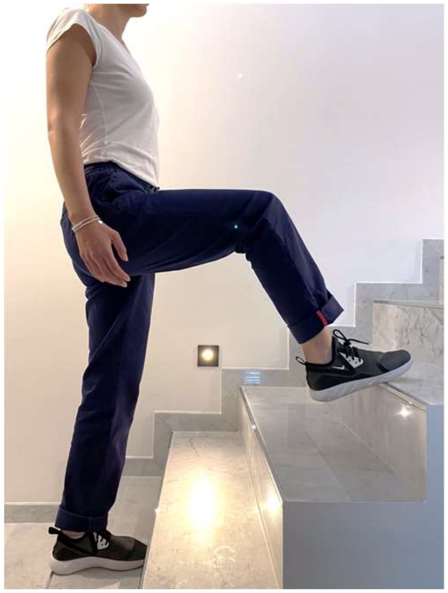
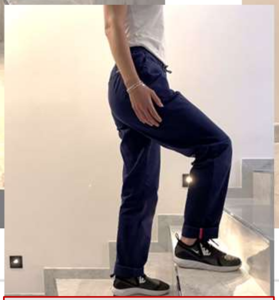

Variation

15 B

Stairs: step taps: put right/left foot on the first step and down again

- Variations: different staircases (rise of step), tap on the 2nd or 3rd step, combination of different steps (e.g., tap 1st/2nd/3rd/2nd/1st step), vary tapping speed, etc.

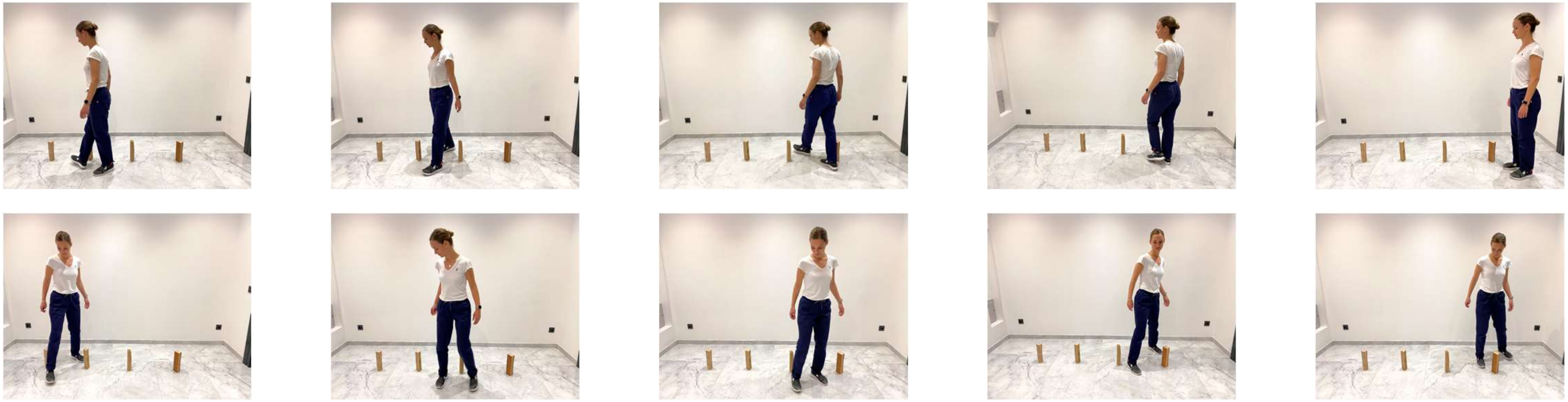

15
C

Slalom walking:
walk forward or sideways around the obstacles

- Variation: large/small distance between the cones/slalom poles

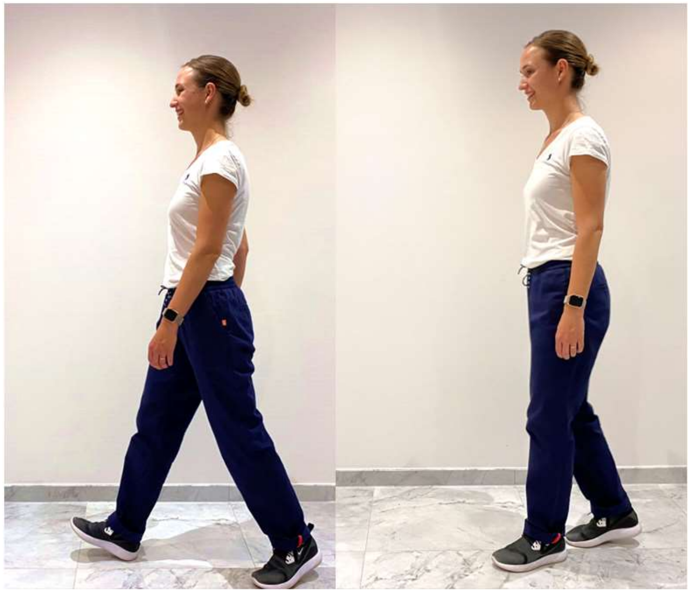

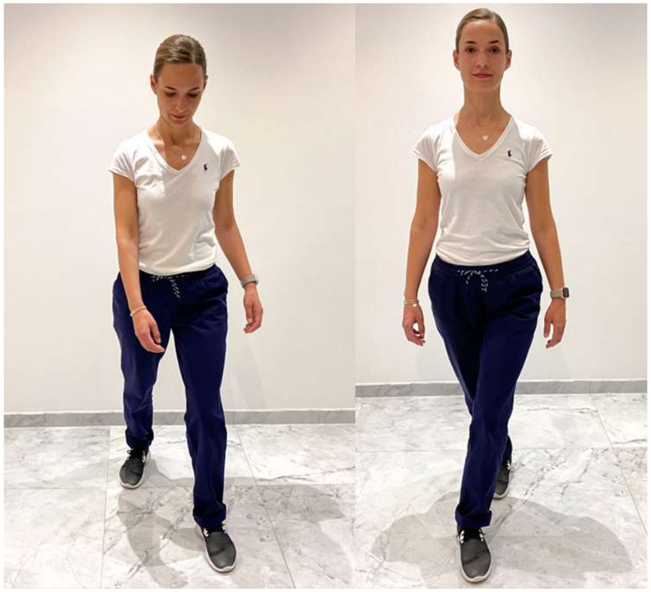

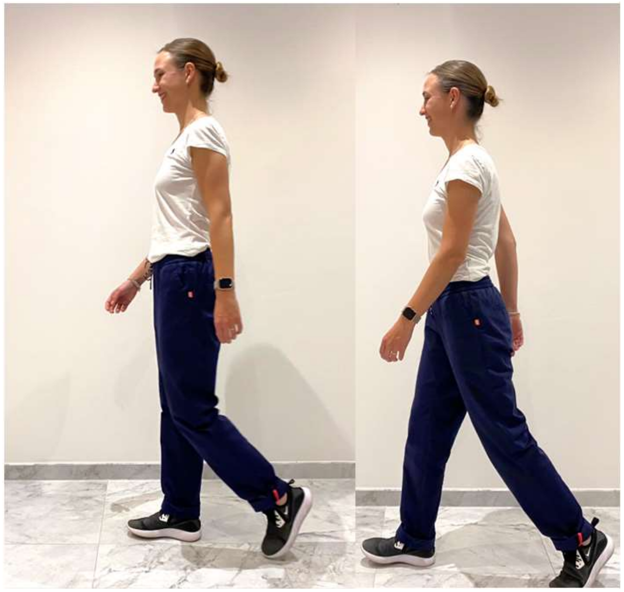

15 D

Walking: variation of gait parameters

- Step length: alternating small and large steps (change every 3rd, 10th, etc. step)
- Stride width: alternating narrow and wide steps (change every 3rd, 10th, etc. step)
- Speed: alternating slow and fast steps (change every 3rd, 10th, etc. step)

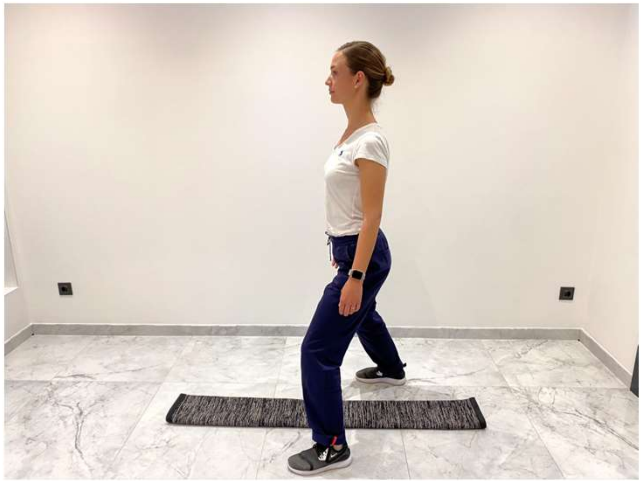

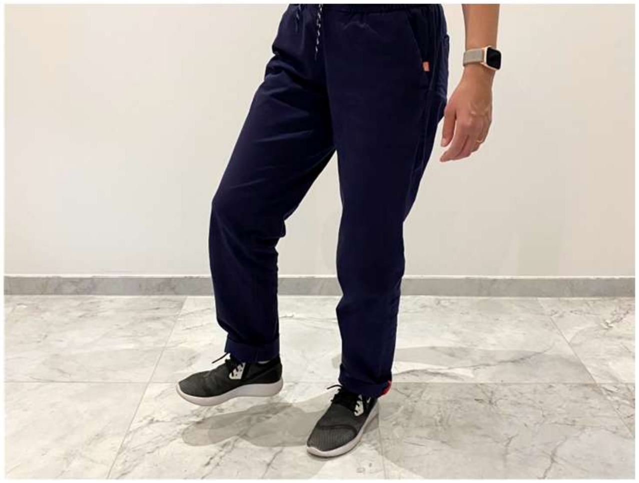

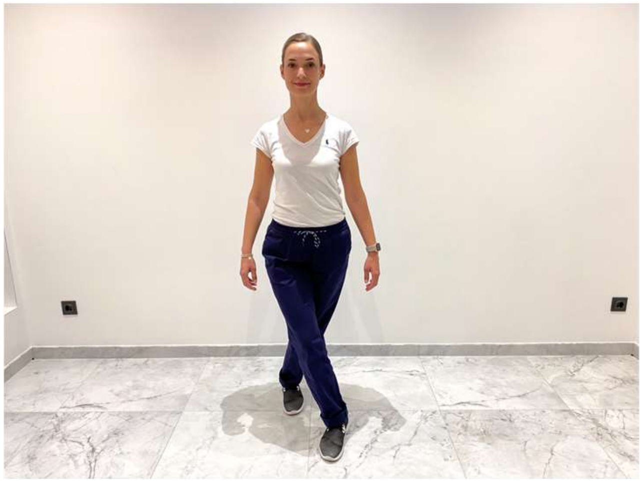

TIPP

Combine exercises 2, 12, 13, 14, 15 with each other for more challenging coordination options

e.g.: tap 4x right, tap 2x left, cross step forward with left foot, cross step backwards with left foot, side step left

VASC-age

Trampoline

Warm Up			
**Number**		**Exercise**	**Description**
**T1**	**a**	Stand hip width apart with eyes closed	Try to bounce a little while keeping your eyes closed.
	**b**	Stand hip width apart: do hip circles	Circle to the left/right side, eyes are open. Increase difficulty by decreasing stance width.
	**c**	Stand hip width apart: do calf raises	If necessary, the handlebar of the trampoline can be used.
**T2**	**a**	Stand hip width apart: bouncing (use handlebar)	Knees are bend, stance width can be varied, no jumping.
	**b**	Stand hip width apart: bouncing (without using the handlebar)	Knees are bend, stance width can be varied, no jumping.
**T3**	**a**	Tandem stance: bouncing (use handlebar)	One foot in front of the other, knees are bend, step length can be varied, no jumping.
	**b**	Tandem stance: bouncing (without using the handlebar)	One foot in front of the other, knees are bend, step length can be varied, no jumping.

VASC••age

Trampoline

Training			
**Number**		**Exercise**	**Description**
**T4**		Walk-in-place	Alternating lifting left and right leg.
**T5**	**a**	Walk-in-motion	Walk forward and backwards on the trampoline.
	**b**	Walk-in-motion	Walk sideways (left/right) on the trampoline.
	**c**	Walk and avoid obstacles (preferable a tennis ball)	Place a ball on the trampoline. As you walk the ball will move – try not to step on the ball!
**T6**	**a**	Stand hip width apart: bouncing and jumping slightly (use handlebar)	Try that both legs are simultaneously in the air.
	**b**	Stand hip width apart: bouncing and jumping slightly (without using the handlebar)	Try that both legs are simultaneously in the air.
**T7**	**a**	Wide to narrow jumps	Jump and land with your feet wide apart – jump and land as narrow as possible – repeat.
	**b**	Jump sideways	Jump alternatingly to the right and left side of the trampoline.
	**c**	Jump forward/backwards	Jump alternatingly forward and backwards.
	**d**	Jump while turning 360°	Turn to the left/right side while jumping. Use several jumps to complete the 360° turn.

Trampoline

Training			
**Number**		**Exercise**	**Description**
**T8**	**a**	Staggered stance: jump forward/backwards	Place right foot in front of the left foot. Knees are bend. Jump forward and land on your right foot and backwards to land on your left foot. Switch sides.
	**b**	Stand hip width apart: jump sideways	Knees are bend. Jump and land on your right foot, jump again to land on your left foot.
**T9**	**a**	Single leg stance	Stand on one leg, knee is bend, try to hold the position (=preliminary exercise for T9b+c).
	**b**	Single leg stance & bouncing	Stand on one leg, knee is bend, try to bounce while keeping the balance.
	**c**	Single leg stance & jumping	Stand on one leg, knee is bend, try to jump and land on the same leg.

VASCage

The COMET-Centre VASCage is funded within the
COMET Programme - Competence Centres for Excellent Technologies
by

- Austrian Ministry for Climate Action, Environment, Energy, Mobility, Innovation and Technology
- Austrian Ministry of Labour and Economy

and the federal states

- Tyrol
- Salzburg
- Vienna

The COMET Programme is conducted by the Austrian Research Promotion Agency (FFG).

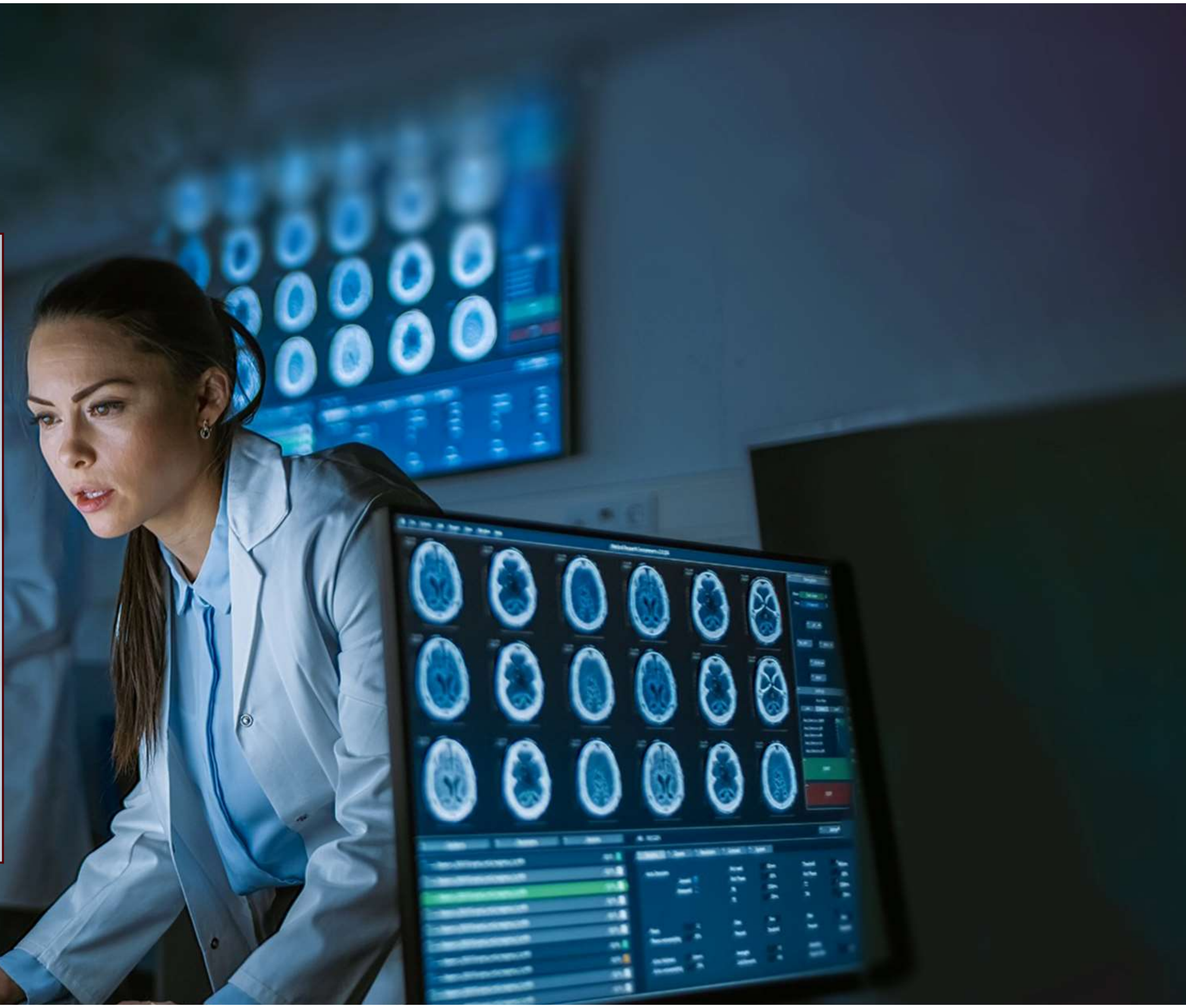

Federal Ministry
Republic of Austria
Labour and Economy

wirtschafts agentur wien

Tirol STANDORTAGENTUR TIROL

FFG Promoting Innovation.

LAND SALZBURG

Federal Ministry
Republic of Austria
Climate Action, Environment, Energy, Mobility, Innovation and Technology
