# Supplementary material for: Recovery of balance and walking in people with ataxia after acute cerebral stroke: study protocol for a prospective, monocentric, single-blinded, randomized controlled trial
Source: Front Stroke. 2024 Aug 5;3:1388891. doi: 10.3389/fstro.2024.1388891 (PMC12802608; doi:10.3389/fstro.2024.1388891)
Supplement: Supplementary file 3 [file Data_Sheet_3.PDF]

VASCage

GLAAS II
Koordinatives Training für Ataxie-Patient*innen[1]

Patricia Meier

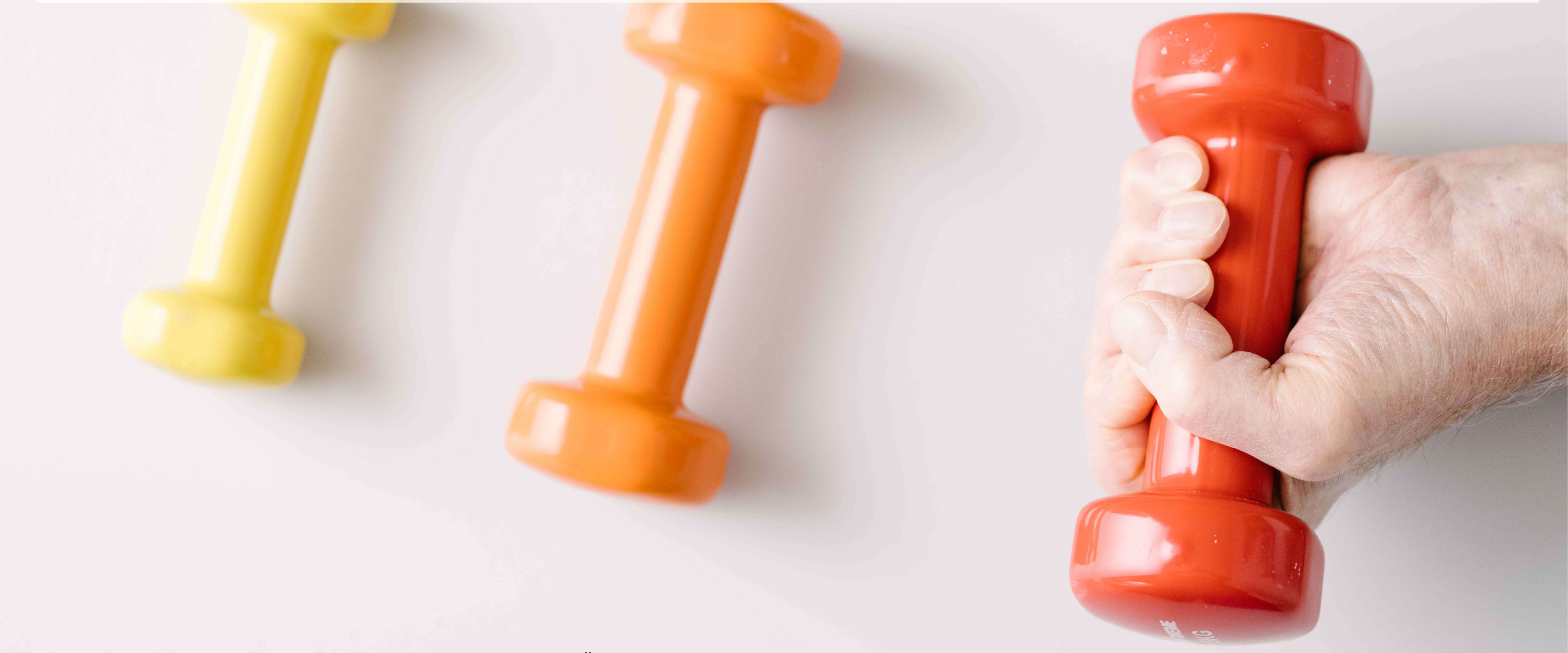

©Rene Böhmer

[1] Übungsprogramm erstellt auf Basis aktueller Studien (Ilg et al. 2009/2010/2012; Brötz et al. 2007) und reevaluiert durch Fragebögen in den teilnehmenden Studienzentren in Tirol.

Zentrale Aspekte (1)

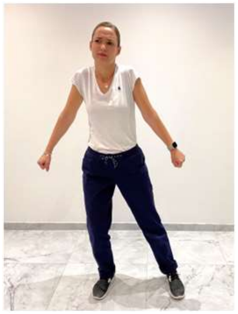

- Fixationsmechanismen sollen erkannt und aufgelöst werden, sodass das Wackeln sichtbar wird. Fixationen sollten im Laufe der Therapie vom Patienten* eigenständig identifiziert und behoben werden.
- Die freie Gelenksbeweglichkeit, insbesondere der Schultergelenke und Wirbelsäule, soll erzielt werden.
- Es sollen wenige, verschiedene Übungen häufig wiederholt werden:
  - eine Übung in einer Variante mind. 10 Wiederholungen (falls zutreffend:) pro Seite
  - Am besten kombinieren Sie drei Übungen bzw. Übungsvariation zu einem Satz und wiederholen diesen Satz dann 3-mal.
- Die Bewegungsabläufe sollen koordinativ herausfordernd sein.

*In dieser Einschulung wird generell – aus Gründen der einfacheren Lesbarkeit – die männliche Form verwendet. Selbstverständlich sind jedoch männliche und weibliche Personen gleichermaßen gemeint.

Zentrale Aspekte (2)

- Übungen sollen im Sinne des „Shapings“ an die Leistungsgrenze des Patienten angepasst sein.
- Die Bewegungen sollen variationsreich sein: die Variation soll durch kleine Veränderungen und systematisch erfolgen.
- Das Training des dynamischen und reaktiven Gleichgewichts soll im Vordergrund stehen.
- Die Ziele und die Übungen in der Therapie sollen alltagsrelevant sein, der funktionelle Kontext (FK) soll für den Patienten immer klar sein.
- Falltraining und Training von Schutzschritten sollen fixe Bestandteile der Therapie sein.
- 20 Therapieeinheiten (5 pro Woche) zu je 45min. & 60 Einheiten eigenständiges Training (5 pro Woche) zu je 15min.

Variationen

- Geschwindigkeit schnell/ langsam
- Rhythmus z.B. durch auditives Cueing
- Vergrößerung/ Verkleinerung der Unterstützungsfläche
  z.B. Stand- oder Spurbreite
- Veränderung der visuellen Kontrolle
  - mit/ohne Kopfbewegungen/ Blickfixation
  - offene/geschlossene Augen
- Einbeziehung eines oder mehrerer alltagsrelevanter Gegenstände

Schwierigkeit steigern (1)

Ob die Schwierigkeit gesteigert werden kann, erkennt man daran, ob der Patient die Fixationsmechanismen noch auflösen und das Wackeln zulassen kann (Hinweis des Therapeuten, die Schultern locker zu lassen, etc. durchaus erlaubt und erwünscht)

- Vom eher Statischen zum Dynamischen
- Von langsamer zu schneller Bewegung
  (CAVE! manchmal ist die schnelle Bewegung auch leichter – z.B. beim Gehen – Vorwärtsvektor!)
- Von eingelenkigen zu mehrgelenkigen Bewegungen
- Vom isolierten Üben mit einer Extremität bis hin zu komplexen Bewegungen

Schwierigkeit steigern (2)

- Von geschlossenen zu offenen Ketten
- Von einfachen zu schwierigen Ausgangsstellungen
- Von einfachen zu komplexen Rhythmen
- Von ungeteilter zu geteilter Aufmerksamkeit
- Keine, ein, mehrere Gegenstände
- Keine, ein, mehrere Hindernisse

Die Schwierigkeit kann durch eine oder auch mehrere Faktoren gesteigert werden, diese sollte aber nacheinander dazu genommen werden.
z.B.: Tapping langsam im Sitzen – Tapping schnell im Sitzen – Tapping langsam im Stehen – Tapping schnell im Stehen

Motorisches Lernen

Prinzipien:

Repetition, Variation, Shaping, Alltagsnähe sowie Handlungs- und Zielorientierung, Regelmäßiges Training

Beeinflussende Faktoren:

- Intrinsische/ extrinsische Motivation: durch adäquate Übungen/ Zielformulierung
- Positive Verstärkung: durch pos. Feedback gelungener Bewegung
- Manuelle Unterstützung: „hands-off"
- externer Fokus: arbeiten mit bildlichen Vorstellungen (über einen Ast steigen, Langlaufen, Scheibenwischer, etc.)

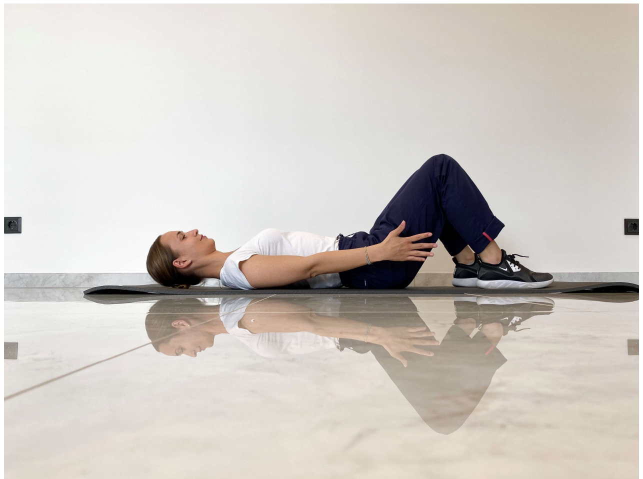 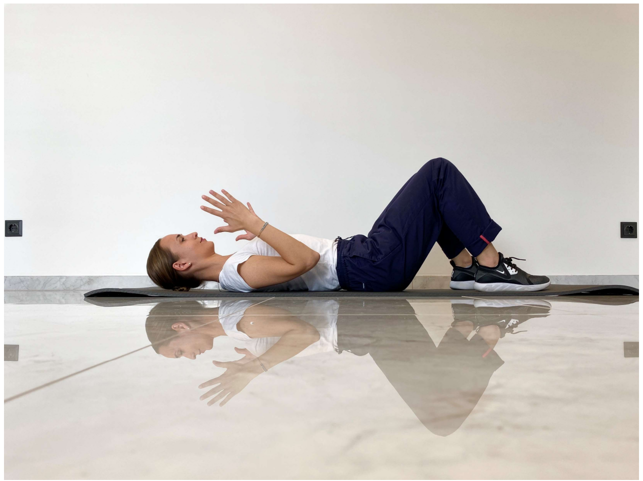 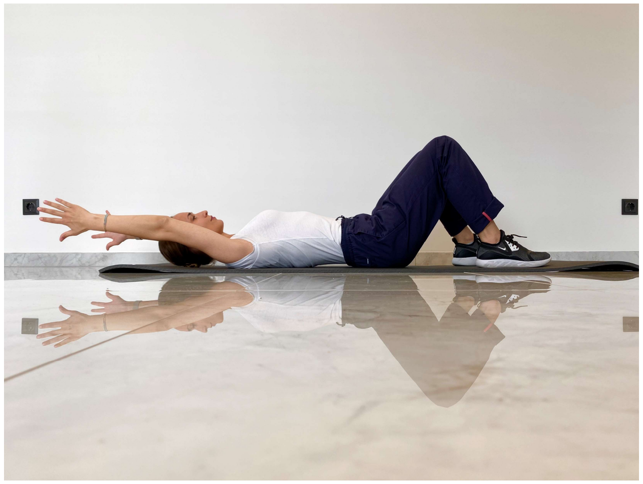

1 A

Armbewegungen in Rückenlage

- Möglicher FK (funktioneller Kontext): Vorübung für: Kleidung in den Schrank einräumen, Bettwäsche aufschütteln, …

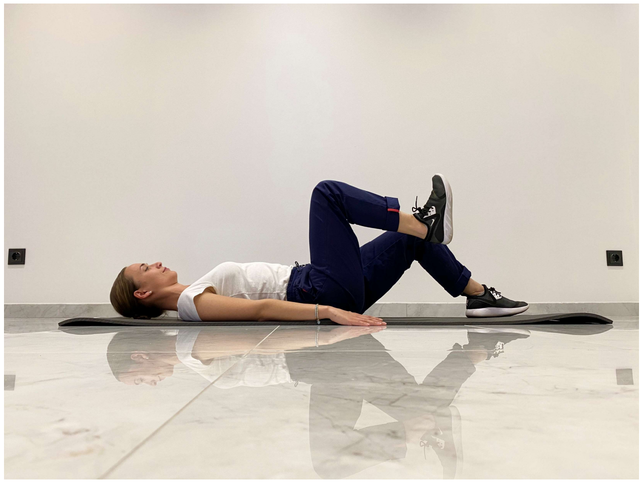

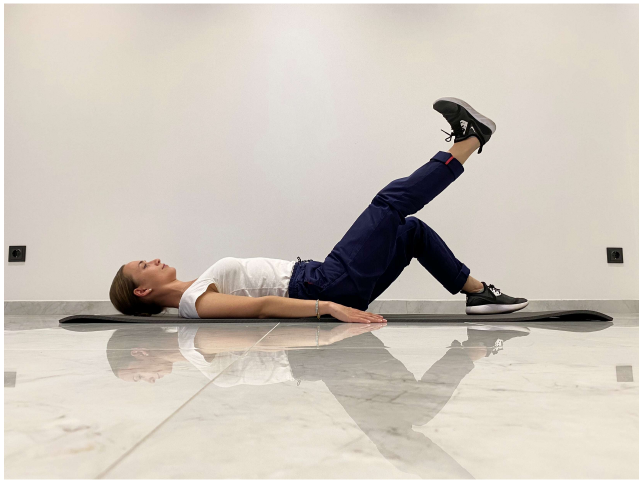

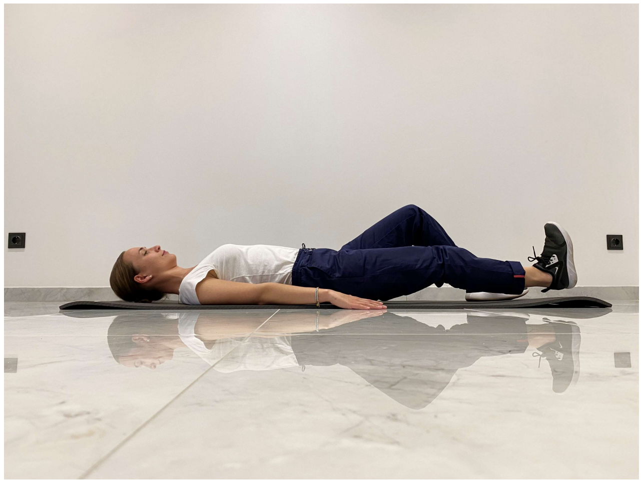

1
B

Beinbewegungen in Rückenlage mit linkem/rechtem Bein

- Möglicher FK: Vorübung für: Gehen, Stiegen steigen, Fahrrad fahren, …

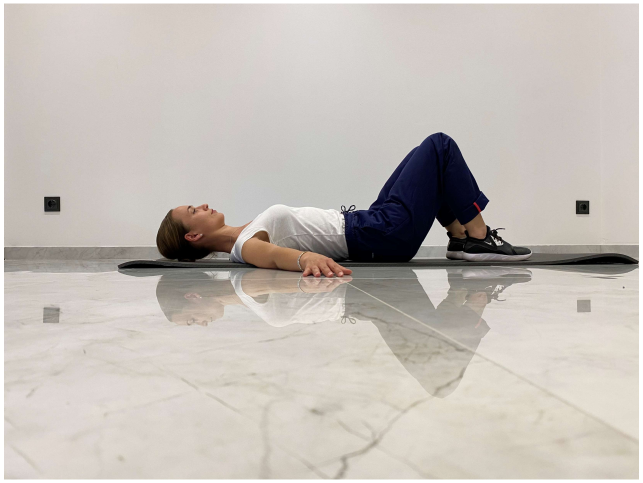

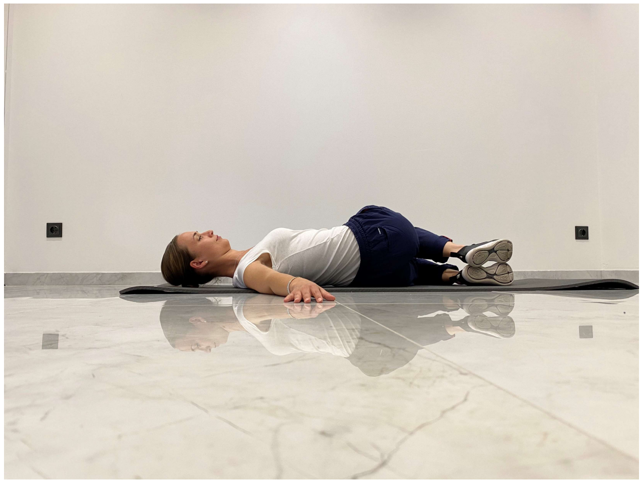

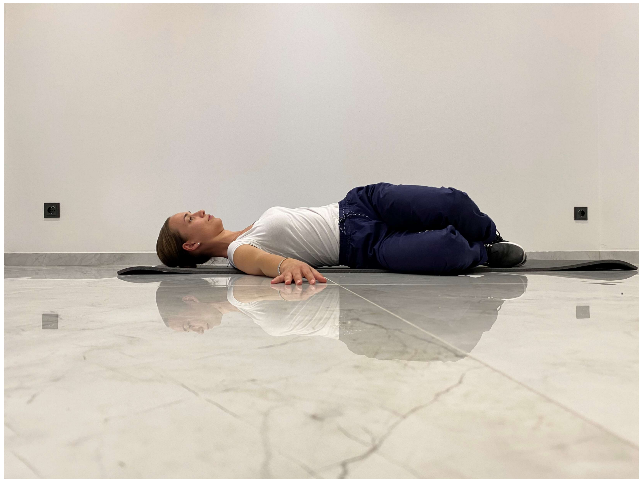

1 C

Drehung der Wirbelsäule in Rückenlage

- Möglicher FK: umdrehen im Bett, …

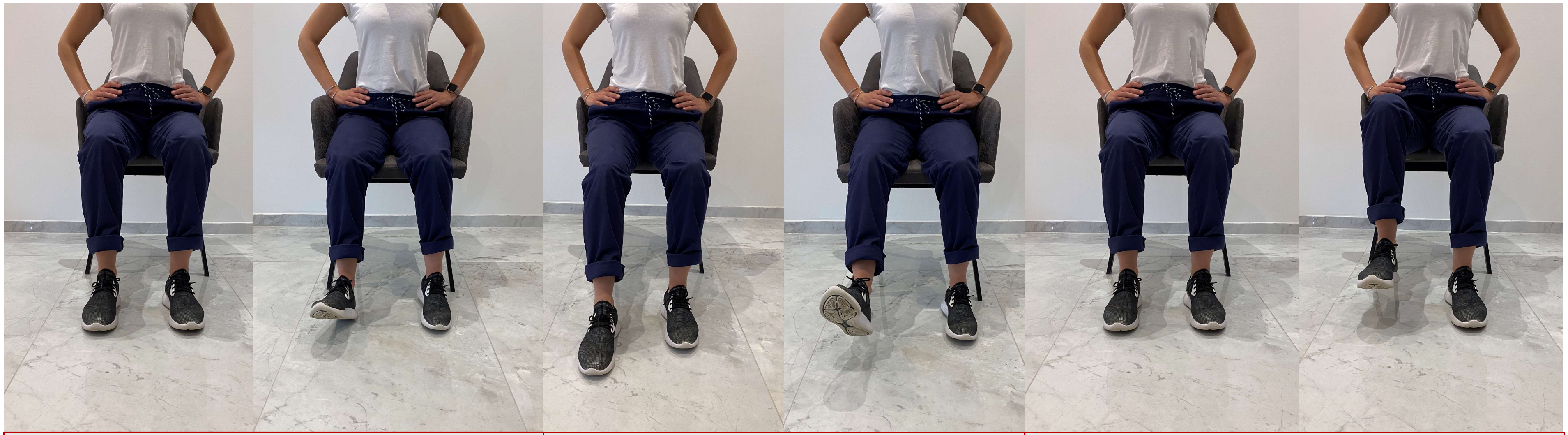

Oberes Sprunggelenk	Kniegelenk	Hüftgelenk

2 A

Im Sitz: in rascher Folge mit Füßen auf Boden „tappen“

Diese Übung dient dem Einstieg in das koordinative Training (hohe Anzahl an WH in schneller Folge über das ganze Bewegungsausmaß, ggf. auch alternierend links/rechts) und kann immer wieder als Vorübung für neue Rhythmen oder Geschwindigkeiten eingesetzt werden.

- Variationen: Bewegung im OSG, KG, HüG;
  leiser/lauter; schneller/langsamer; versch. Rhythmen (z.B. 2x li/1x re)
- Möglicher FK: Vorübung für das Gehen oder auch Tanzen

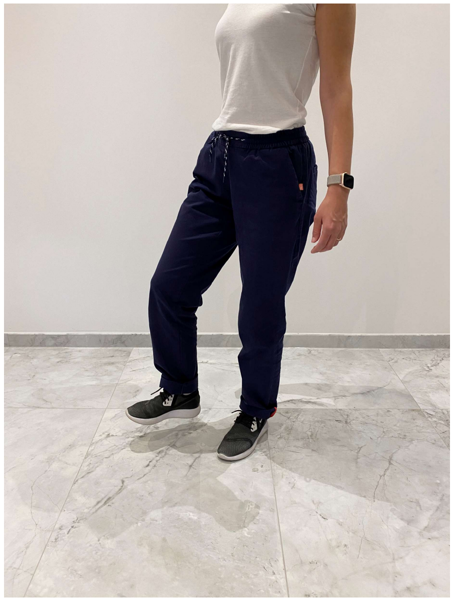

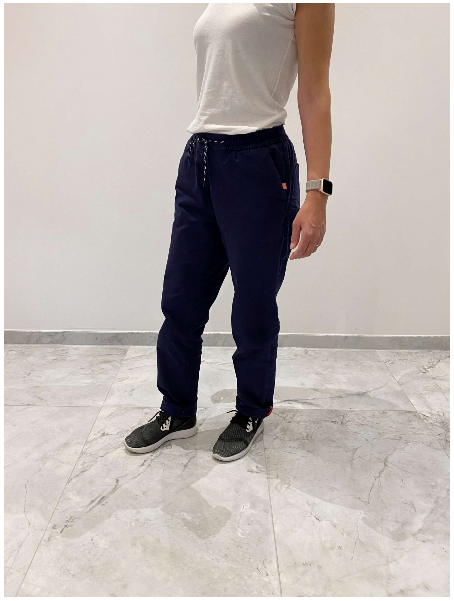

2 B

Im Stand: in rascher Folge mit Füßen auf Boden „tappen“

Dient ebenfalls als Einstiegs- oder Vorübung.

- Variationen: Beine in versch. Höhen anheben lassen; leiser/lauter „tappen“; schneller/langsamer „tappen“; versch. Rhythmen (z.B. 2x li/1x re)
- Möglicher FK: Vorübung für das Gehen sowie für Schutzschritte oder auch für das Tanzen

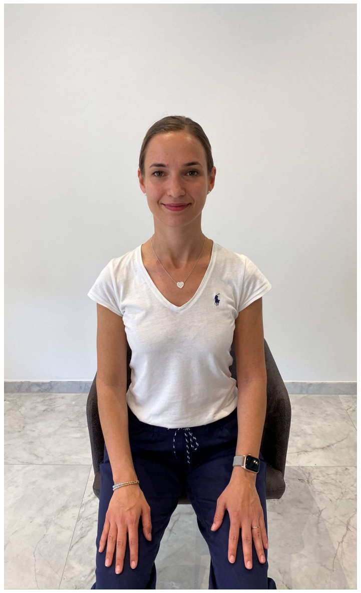 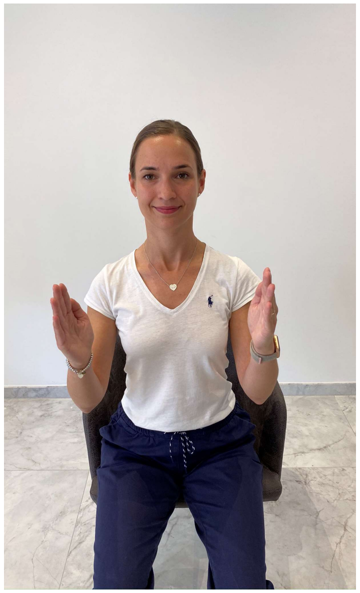 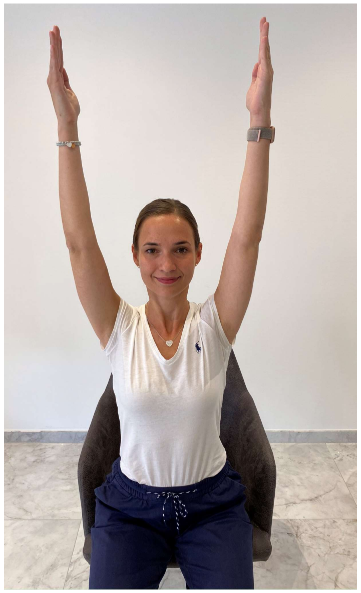 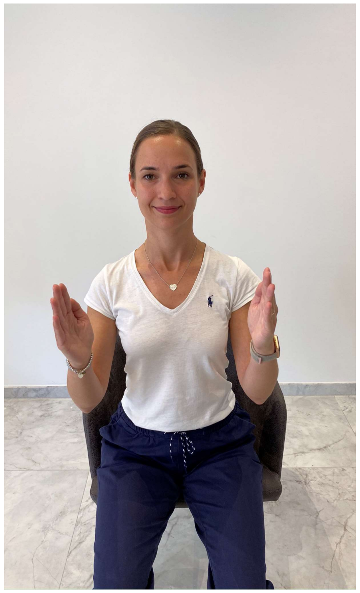 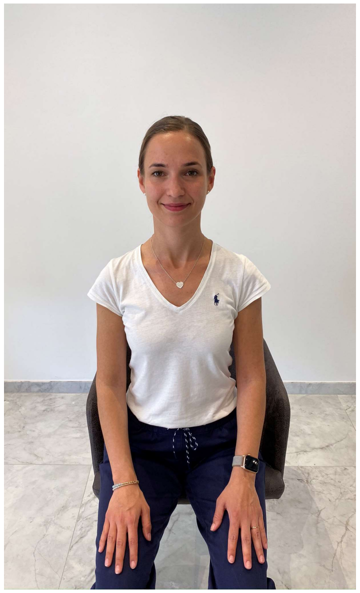

3 A

Armbewegungen im Sitz

- Variation: verschiedene Übungen mit Jonglierbällen/-tüchern
- Möglicher FK: Kleidung in den Schrank einräumen, Bettwäsche aufschütteln, …

3B = Übung 3A plus...

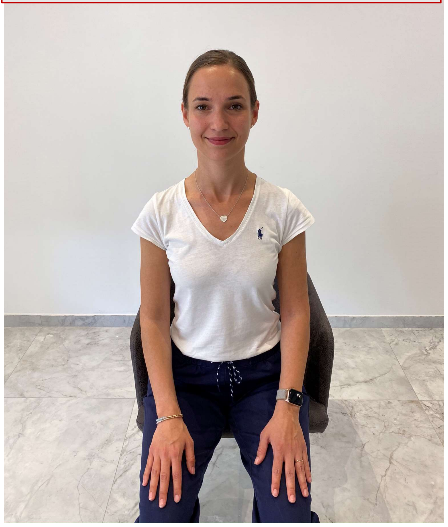

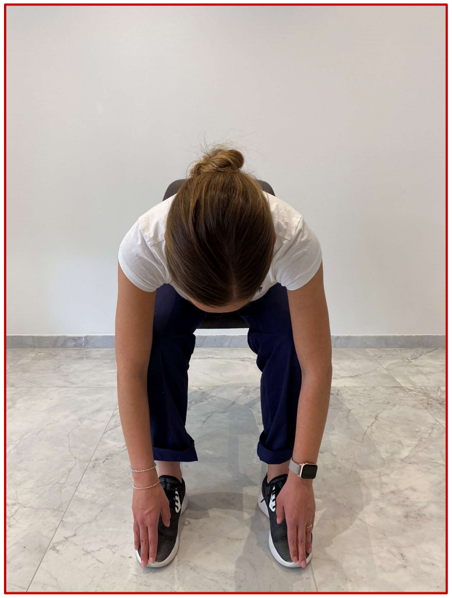

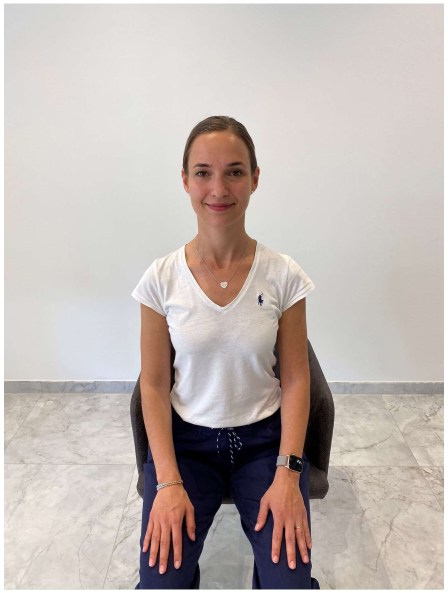

3
B

Armbewegungen im Sitz bis zu den Zehenspitzen

- Möglicher FK: Schuhe/ Socken/ eine Hose anziehen, ...

3C = Übung 3B plus…

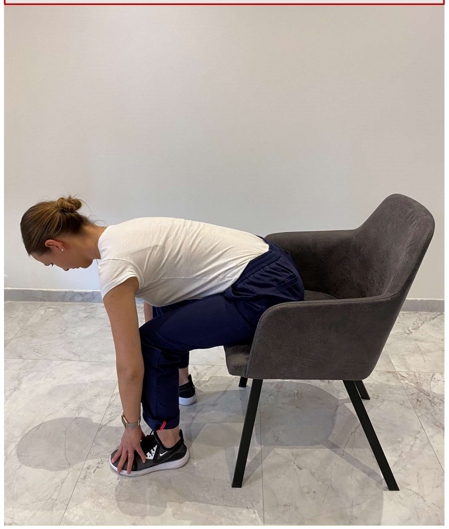

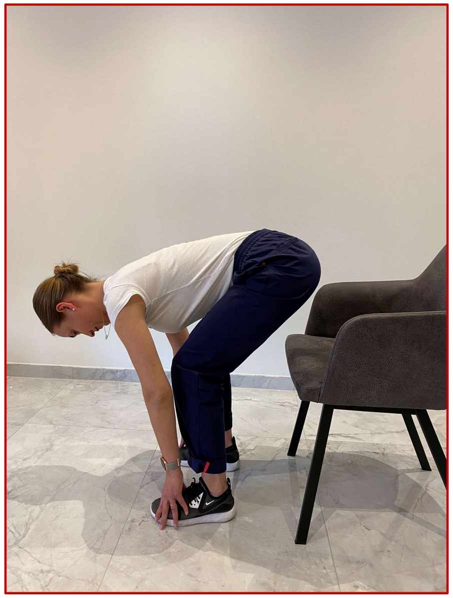

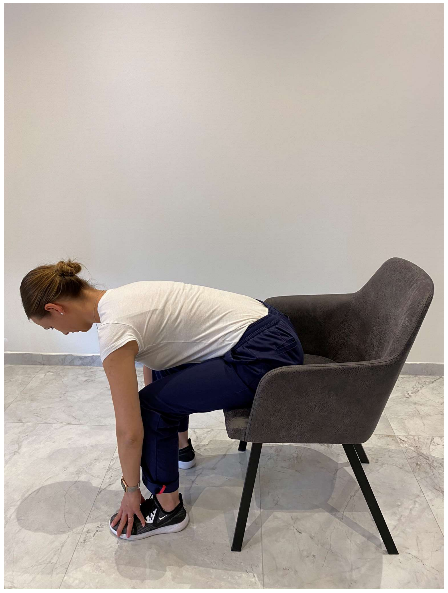

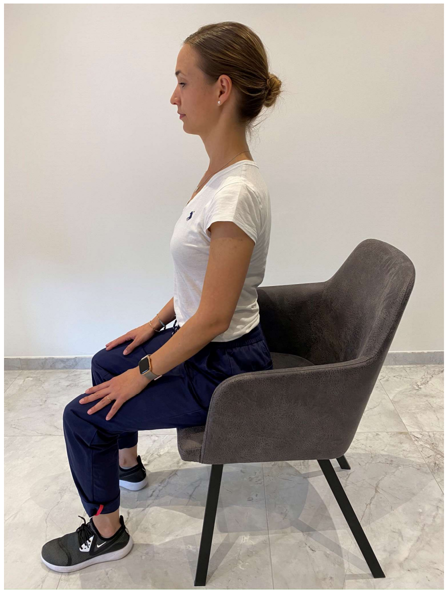

3
C

Armbewegungen im Sitz bis zu den Zehenspitzen und Gesäß anheben

…in umgekehrter Reihenfolge wieder zurück zum Sitz

- Möglicher FK: eine Hose anziehen, aus der Tiefen Hocke aufstehen, …

3D = Übung 3C plus…

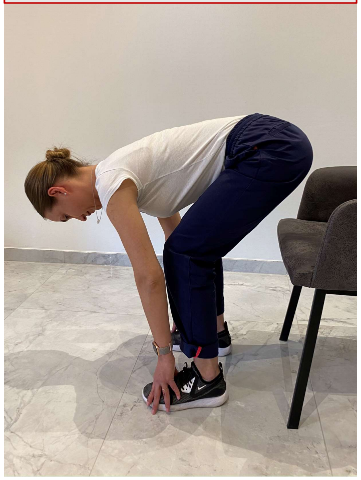
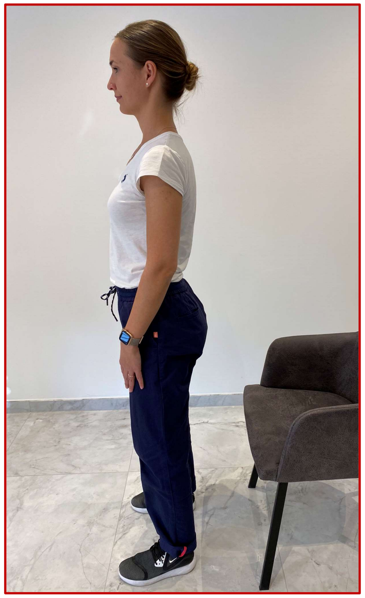
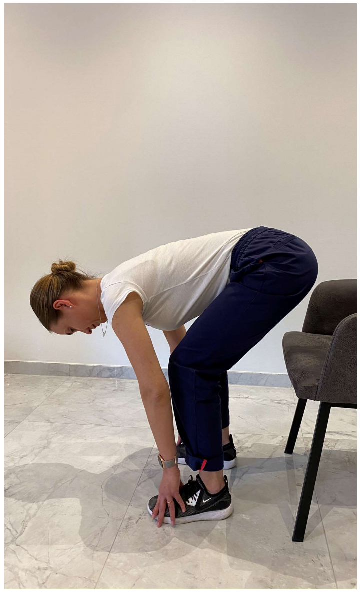
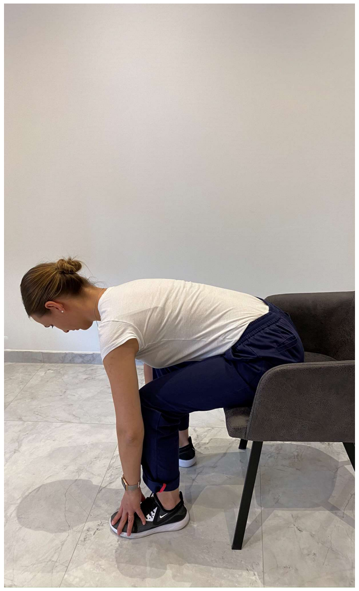
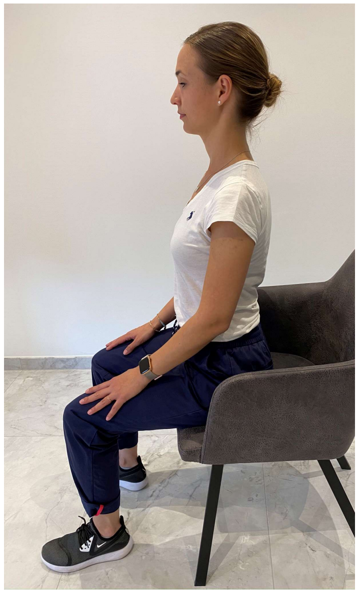

3
D

Armbewegungen im Sitz bis zu den Zehenspitzen & Gesäß anheben & aufstehen

…in umgekehrter Reihenfolge wieder zurück zum Sitz

- Möglicher FK: eine Hose anziehen, aus der Tiefen Hocke aufstehen, …

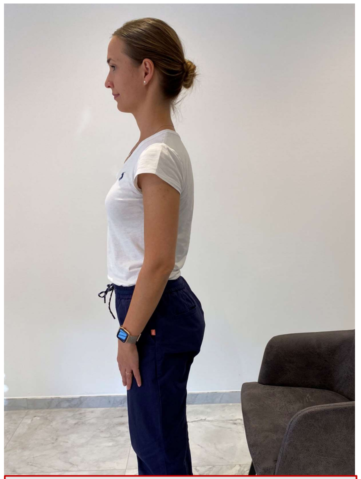

3E = Übung 3D plus…

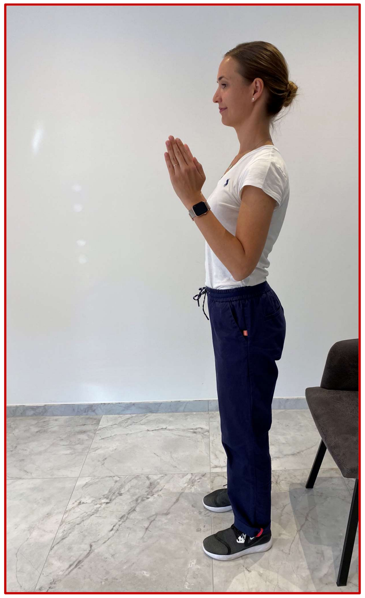

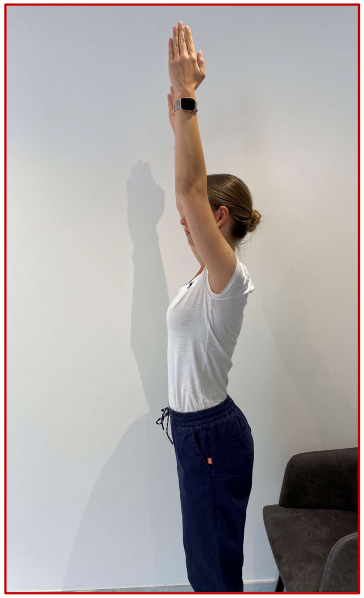

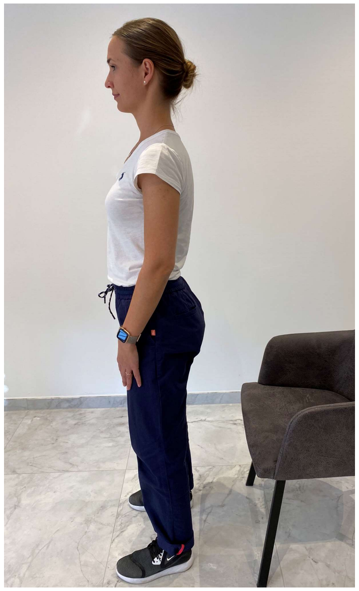

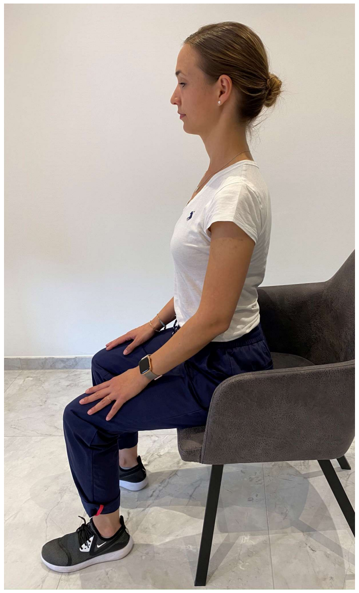

3 E

Armbewegungen… Zehenspitzen & Gesäß anheben & aufstehen & Arme Über-Kopf heben

…in umgekehrter Reihenfolge wieder zurück zum Sitz

- Möglicher FK: Schuhe anziehen und Mantel nehmen, morgens aus dem Bett aufstehen und sich strecken, …

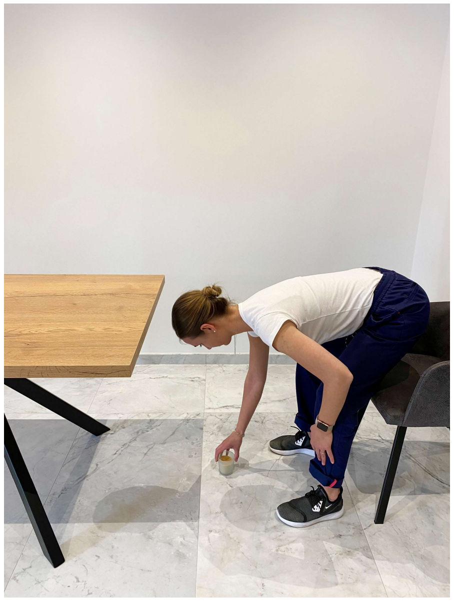

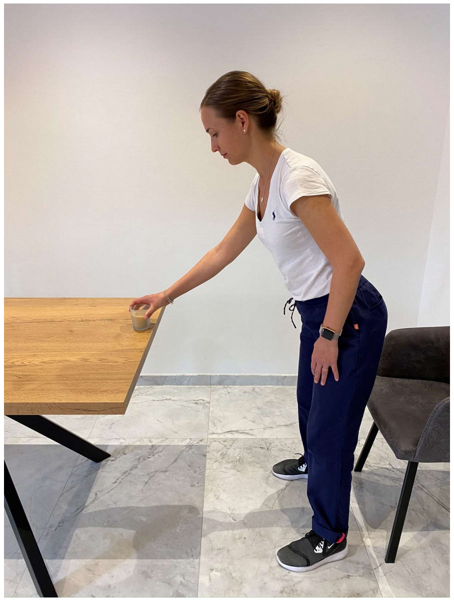

3 F

Im Stand (hüftbreit) Gegenstand vom Boden aufheben und vor sich am Tisch platzieren

- Variationen: Ausgangsstellung: schmaler Stand, Schrittstellung, Tandemstand; Gegenstand: auf verschiedenen Höhen platzieren (z.B. im Regal)

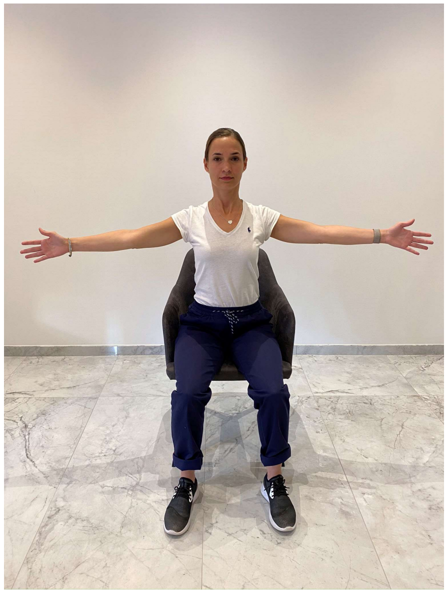
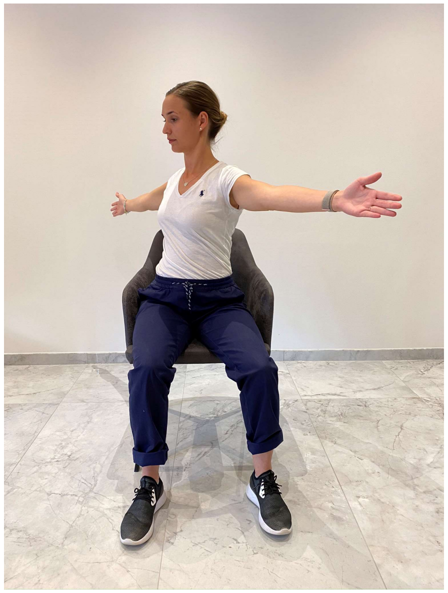
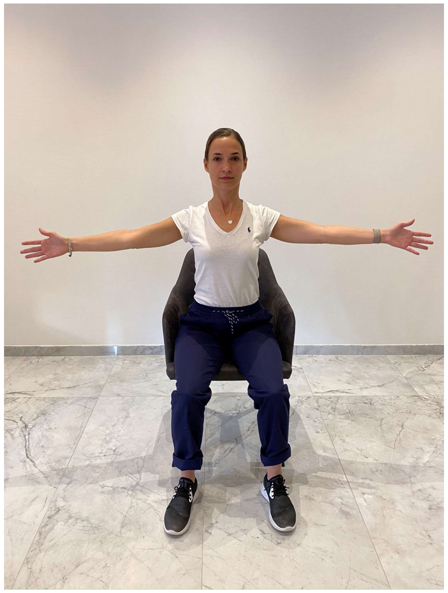
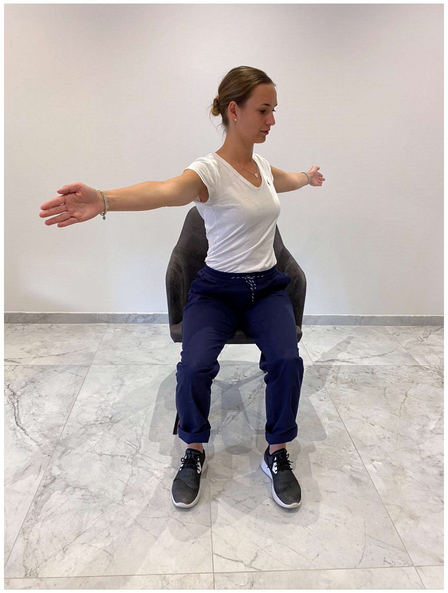

4 A

Im Sitz den Rumpf nach rechts/links rotieren, Arme sind dabei seitlich ausgestreckt

…und wieder zurück in die Mitte

- Variationen: Sitz ohne Bodenkontakt der Füße, versch. Ausgangsstellungen (z.B. Stand breit/schmal, etc.)
- Möglicher FK: über die linke/rechte Schulter nach hinten schauen, einen Gegenstand hinter sich ergreifen, …

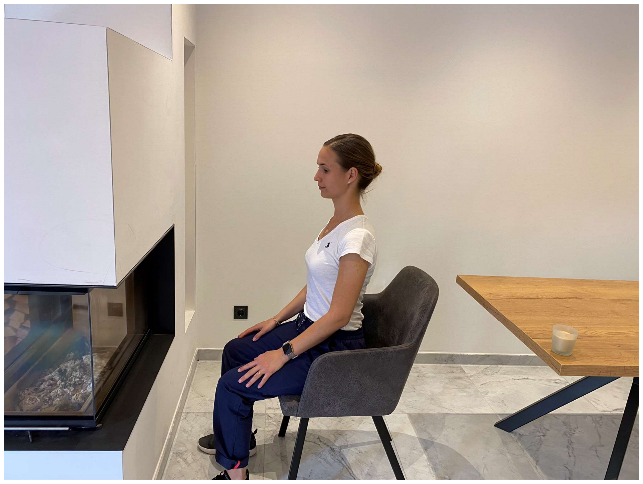

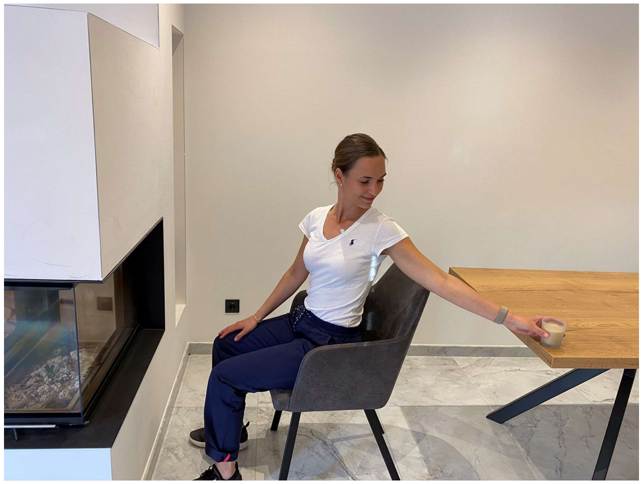

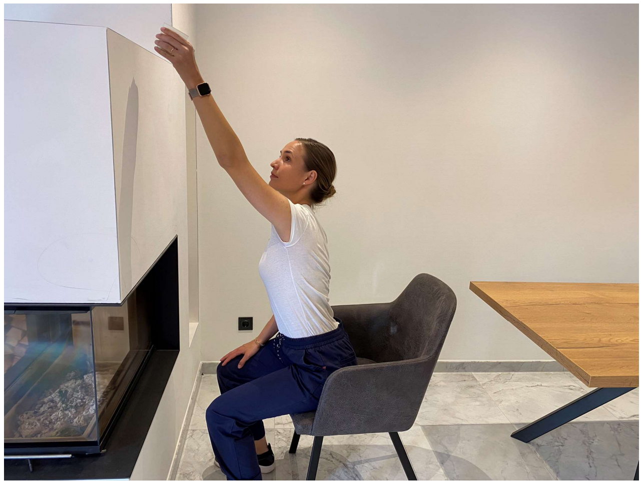

4
B

Im Sitz den Rumpf rotieren, um mit einer Hand einen Gegenstand zu ergreifen

…und den Gegenstand vor sich oben im Regal platzieren; mit allen Gegenständen wiederholen

- Variation: Sitz ohne Bodenkontakt der Füße, versch. Ausgangsstellungen (z.B. Stand breit/schmal, etc.), verschiedene Höhen: z.B. Gegenstand vor sich am Boden platzieren

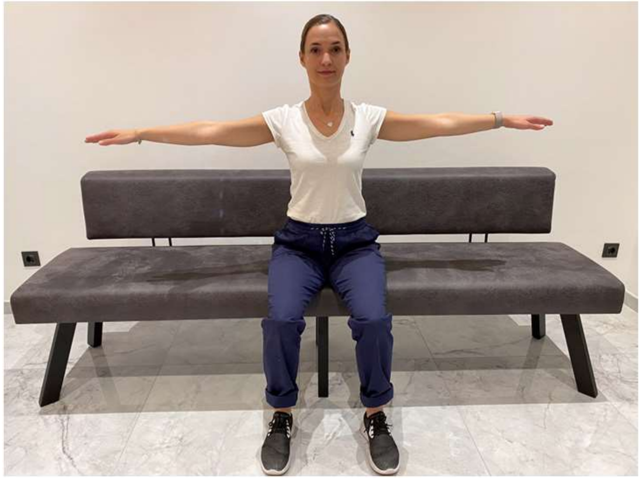

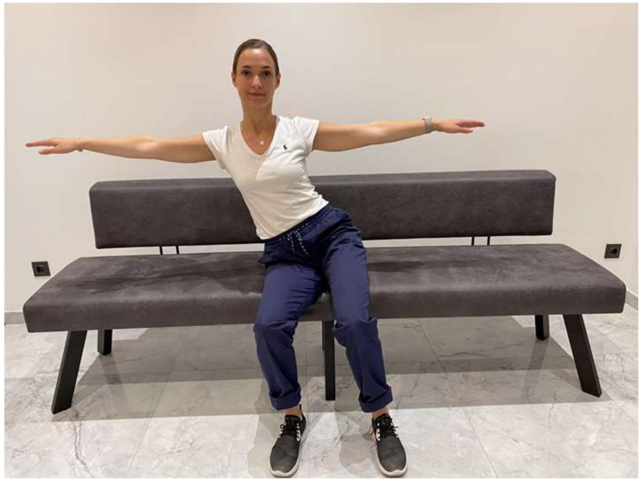

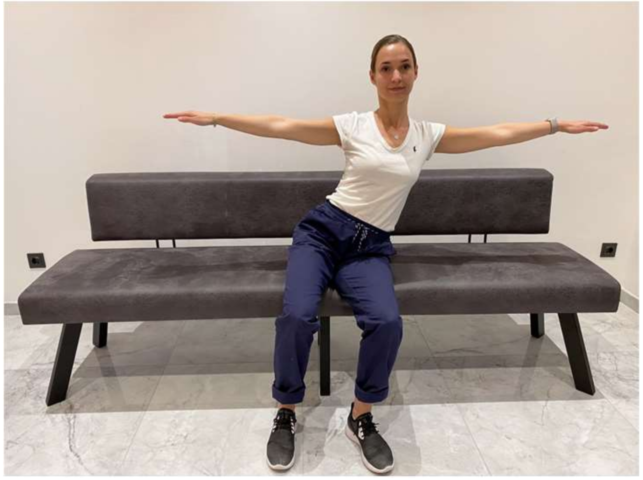

5 A

Gewichtsverlagerung nach rechts/links im Sitz

..und wieder zurück in die Mitte

- Variation: Sitz ohne Bodenkontakt der Füße
- Möglicher FK: einen Gegenstand ergreifen, …

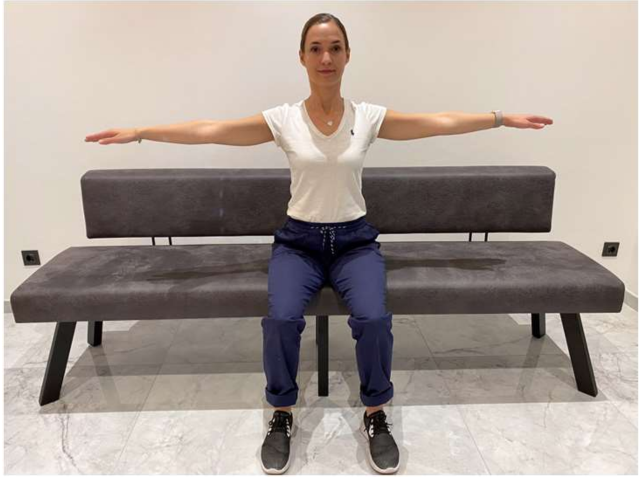

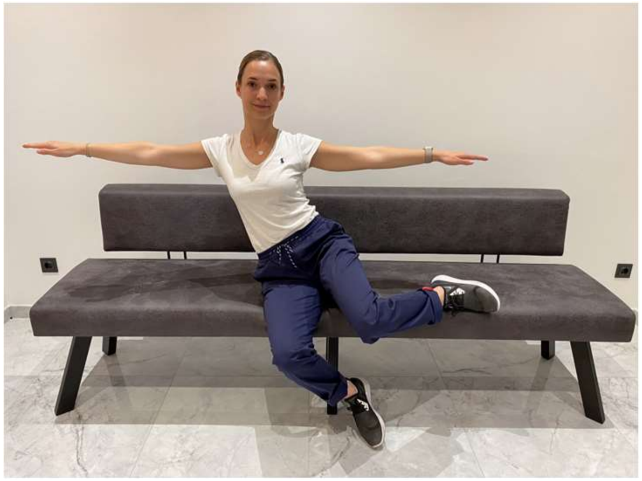

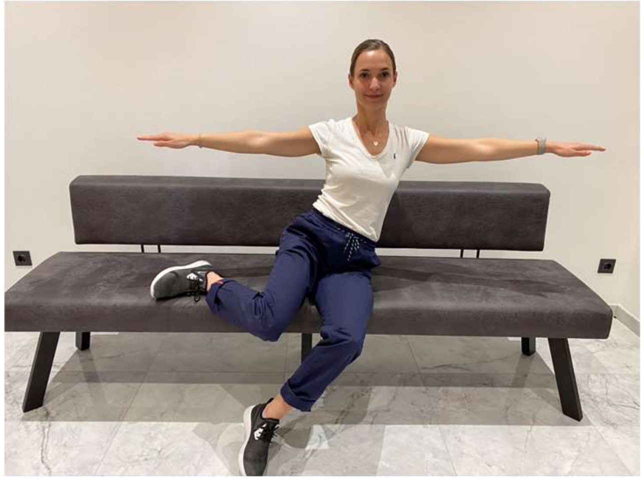

5
B

Gewichtsverlagerung nach rechts/links im Sitz & 1 Bein anheben (auf das Bett)

…in umgekehrter Reihenfolge wieder zurück in die Mitte

- Variation: Sitz ohne Bodenkontakt der Füße
- Möglicher FK: sich ins Bett legen, gemütlich auf die Couch setzen, …

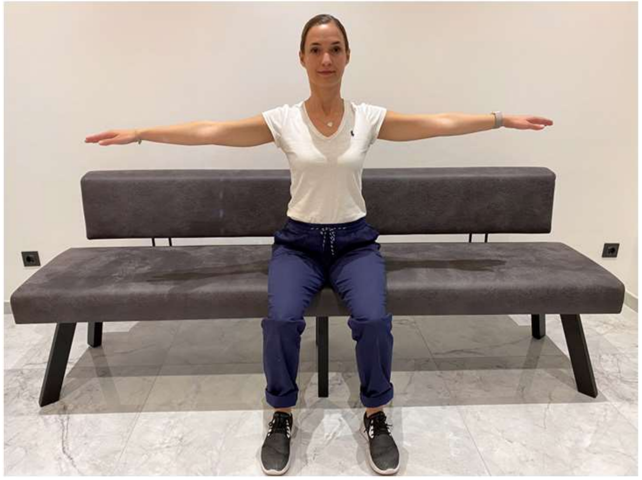

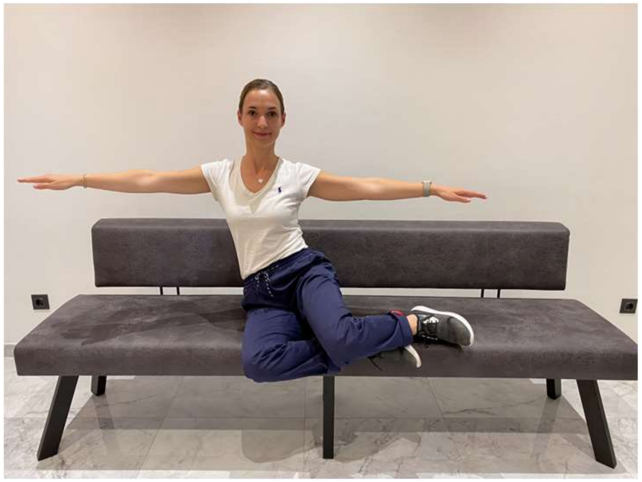

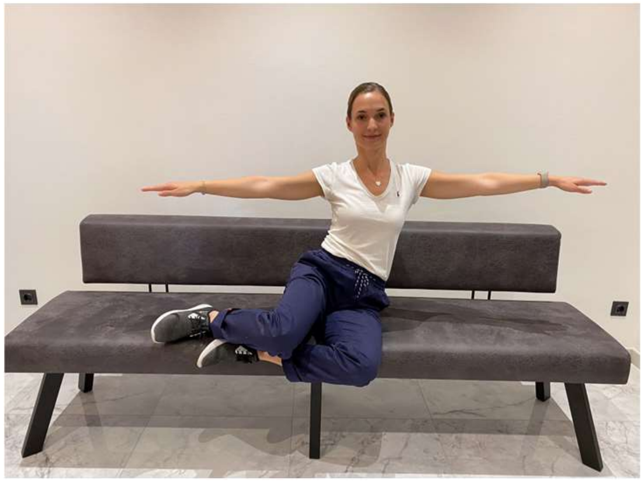

5 C

Gewichtsverlagerung nach rechts/links im Sitz & beide Beine anheben (auf das Bett)

…in umgekehrter Reihenfolge wieder zurück in die Mitte

- Variation: Sitz ohne Bodenkontakt der Füße
- Möglicher FK: sich ins Bett legen, gemütlich auf die Couch setzen, …

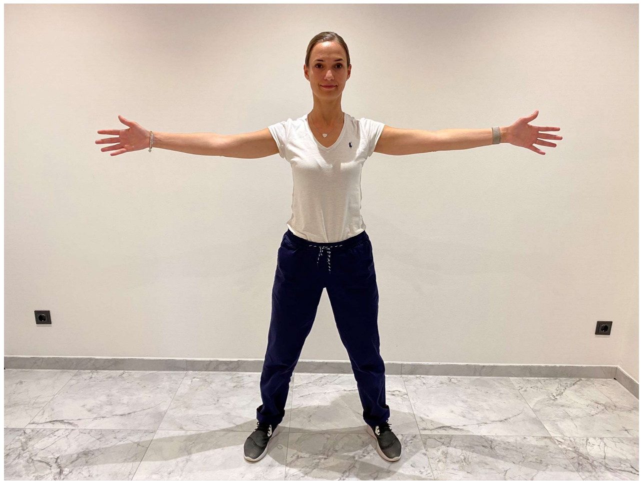

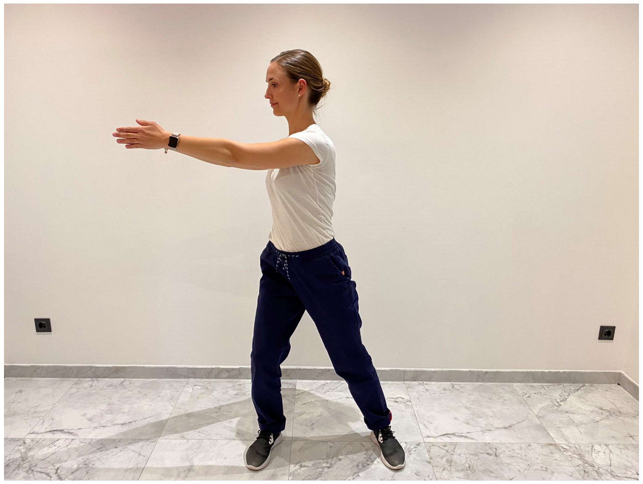

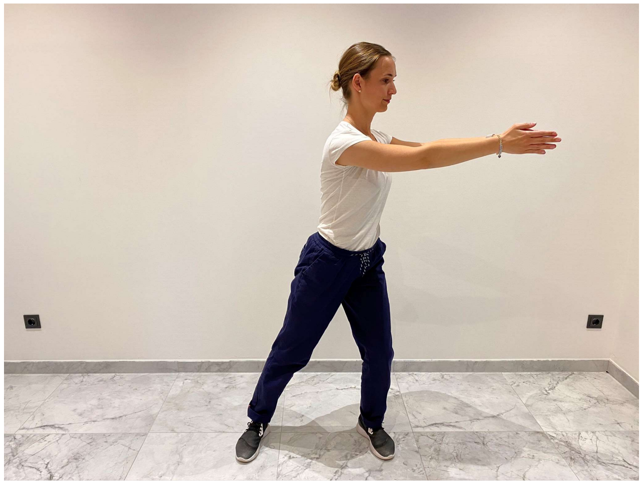

6
A

Breiter Stand: in raschem Wechsel das Gewicht ganz nach re/li verlagern & in die Hände klatschen

…und wieder zurück in die Mitte

- Möglicher FK: Wäsche an der Wäscheleine auf- oder abhängen, Gegenstände aus einem Regal holen oder reinlegen

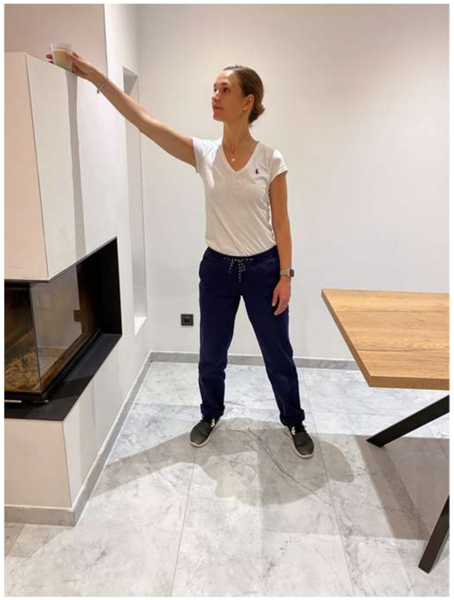

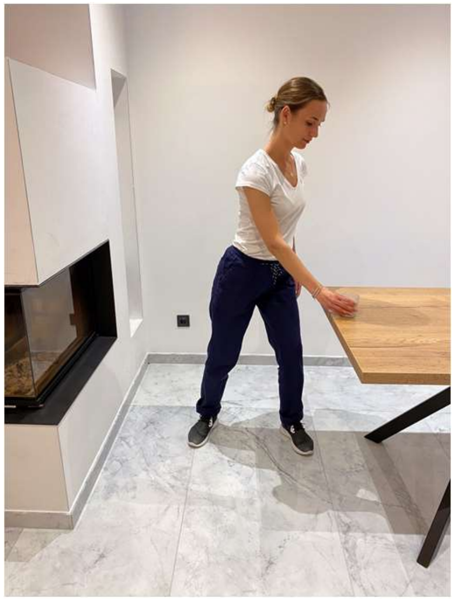

6
B

Breiter Stand: in raschem Wechsel Gegenstände von rechts & links holen

…und vor sich auf dem Tisch platzieren

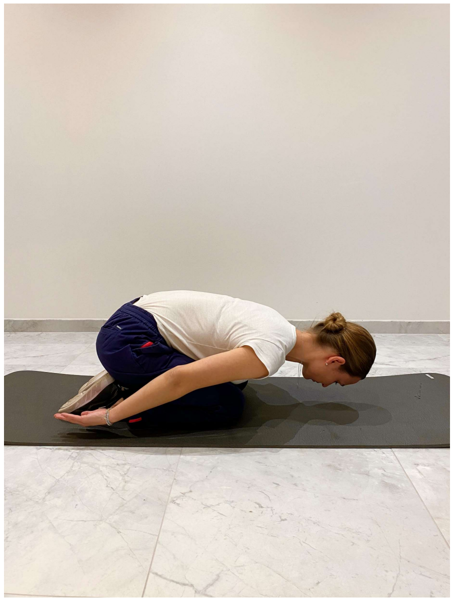

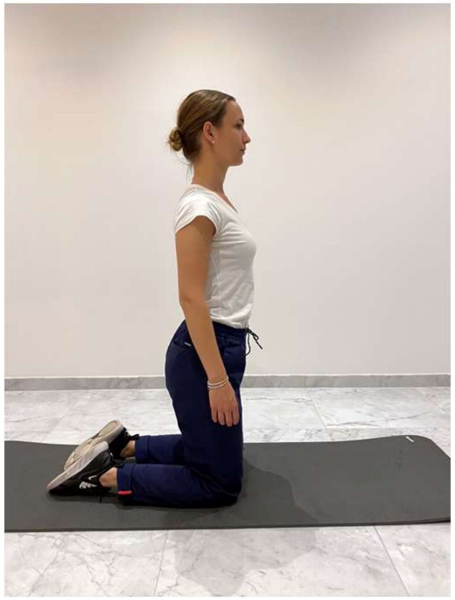

7
A

Hocke - Kniestand

…in umgekehrter Reihenfolge wieder zurück in die Hocke

- Möglicher FK: aufstehen

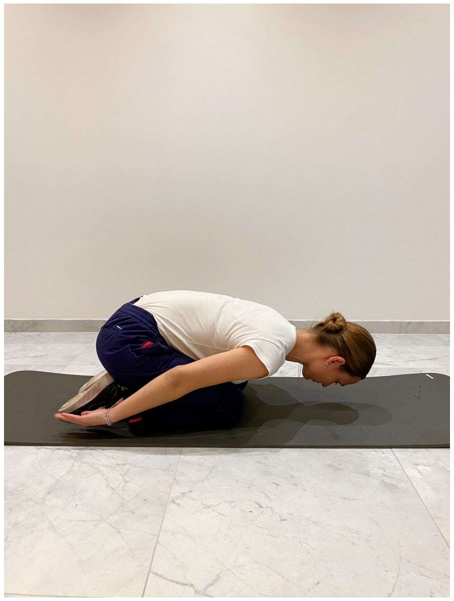
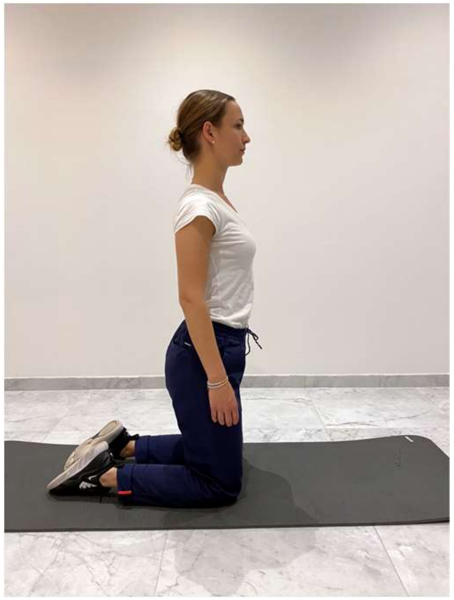
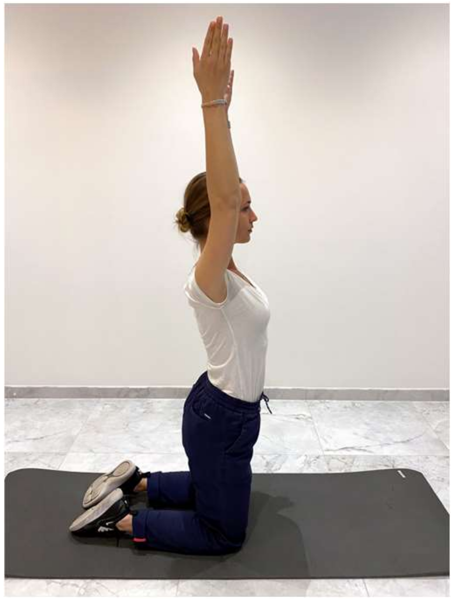

7 B

Hocke – Kniestand & Arme hoch

…in umgekehrter Reihenfolge wieder zurück in die Hocke

- Möglicher FK: aufstehen

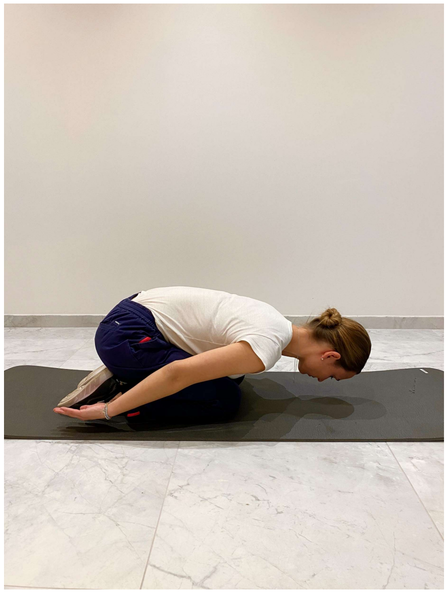 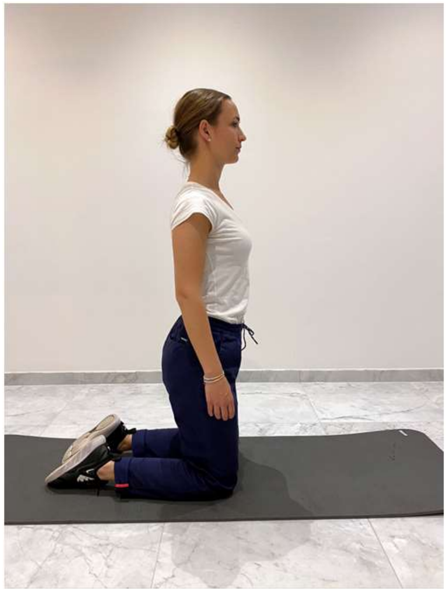 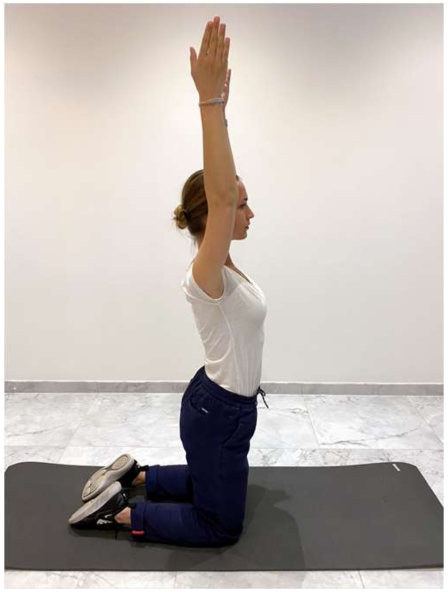 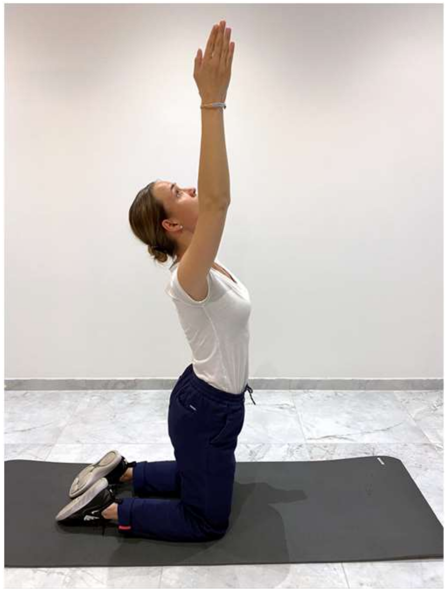

7 C

Hocke – Kniestand & Arme hoch & Händen nachschauen

…in umgekehrter Reihenfolge wieder zurück in die Hocke

- Möglicher FK: aufstehen

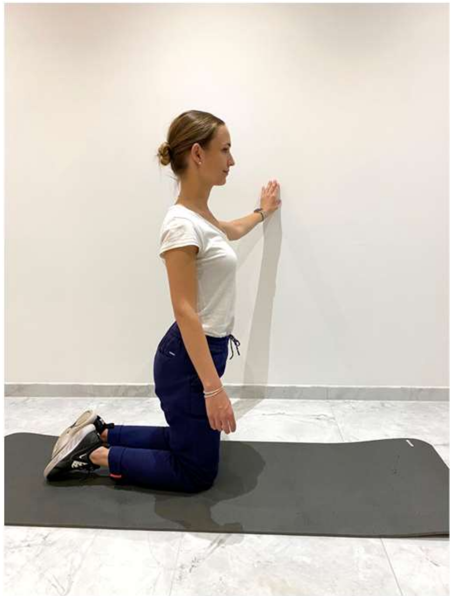

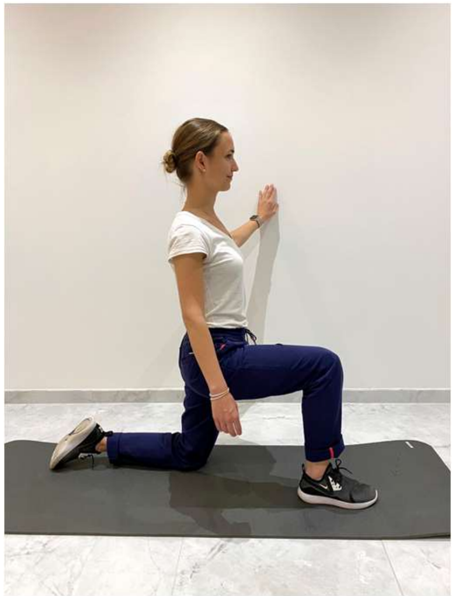

8 A

Mit festhalten: Kniestand – Halbkniestand mit linkem/rechtem Bein

…und wieder zurück in den Kniestand

- Möglicher FK: aufstehen vom Boden

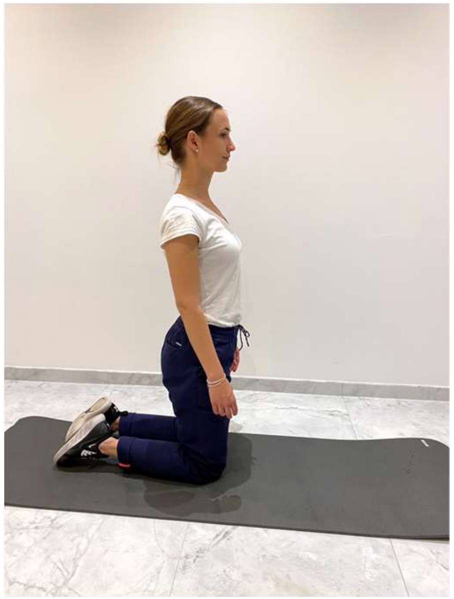

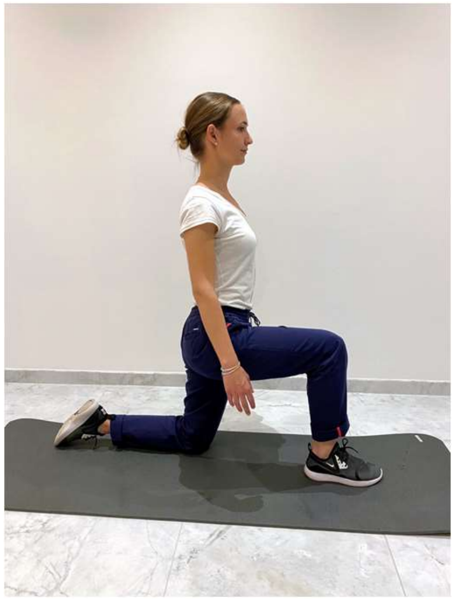

8
B

Ohne festhalten: Kniestand – Halbkniestand mit linkem/rechtem Bein

…und wieder zurück in den Kniestand

- Möglicher FK: aufstehen vom Boden

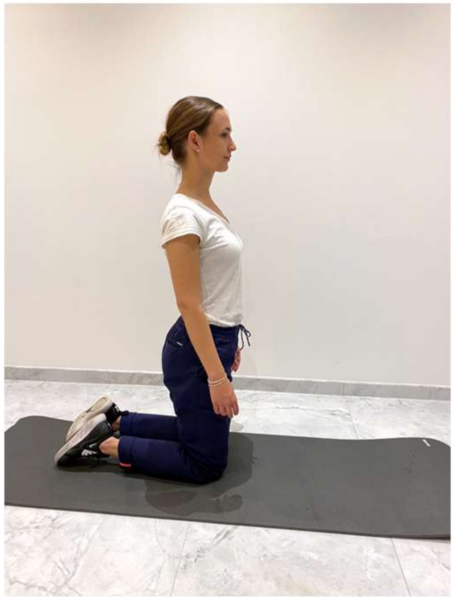

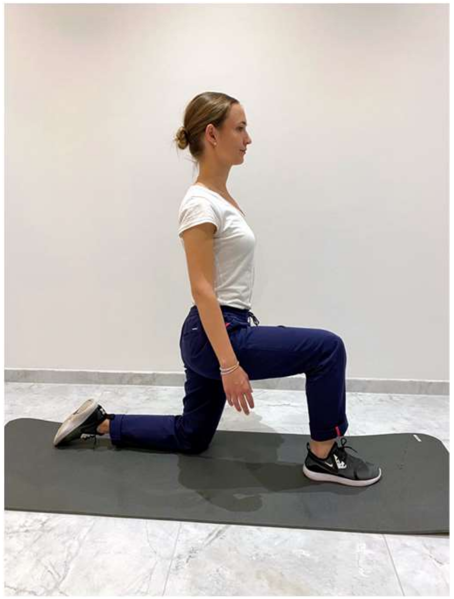

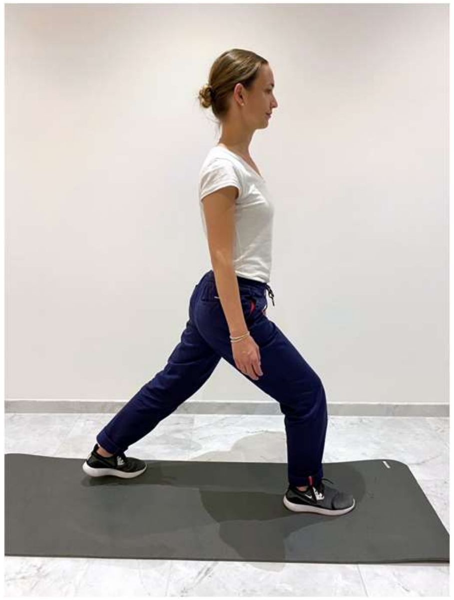

8 C

Kniestand – Halbkniestand – Schrittstellung mit linkem/rechtem Bein

…in umgekehrter Reihenfolge wieder zurück in den Kniestand

- Möglicher FK: aufstehen vom Boden

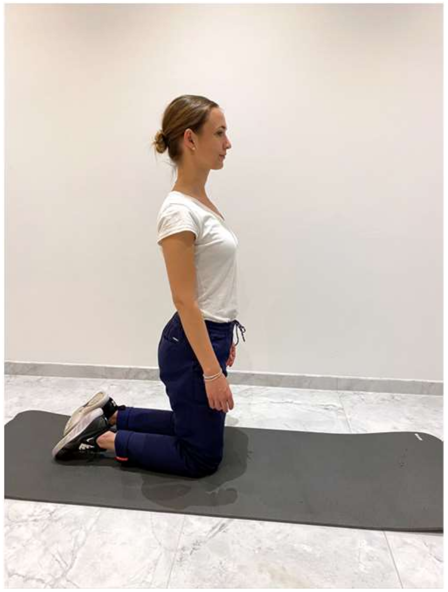
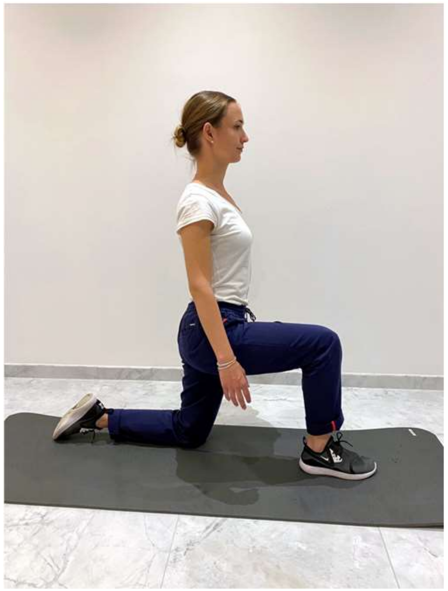
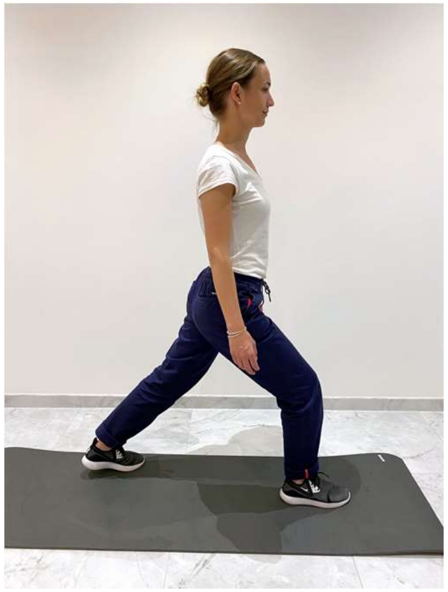
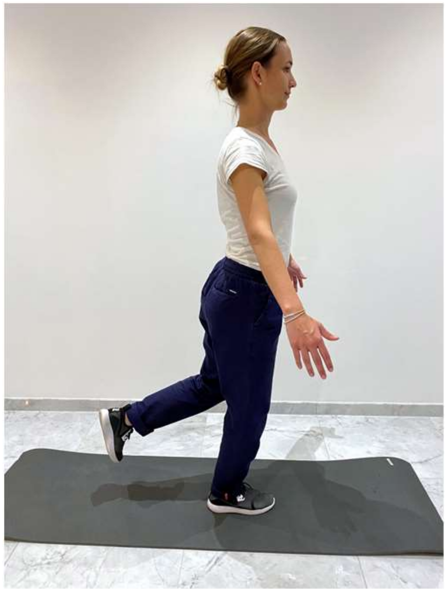

8
D

Kniestand – Halbkniestand – Schrittstellung – Einbeinstand mit linkem/rechtem Bein

…in umgekehrter Reihenfolge wieder zurück in den Kniestand

- Möglicher FK: aufstehen vom Boden

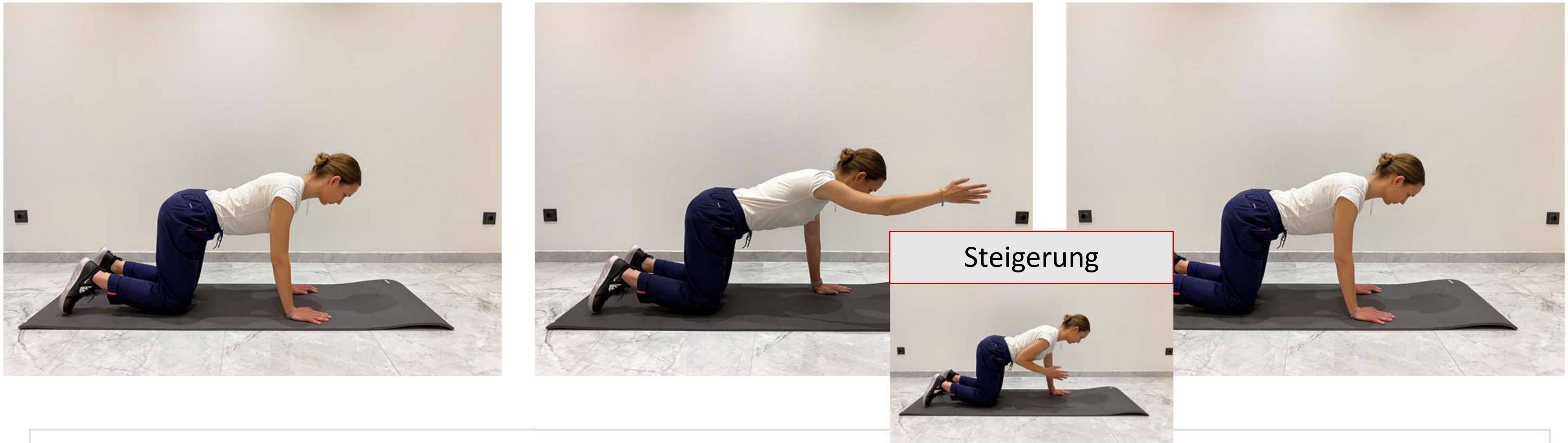

9
A

Vierfüßler-Stand abwechselnd den linken & rechten Arm nach vorne strecken

- Steigerung: & Ellbogen Richtung Bauch ziehen, dann wieder abstellen
- Möglicher FK: im Bett liegend den Wecker ausschalten; Vorübung für aufstehen vom Boden, Stiegen steigen und Gehen

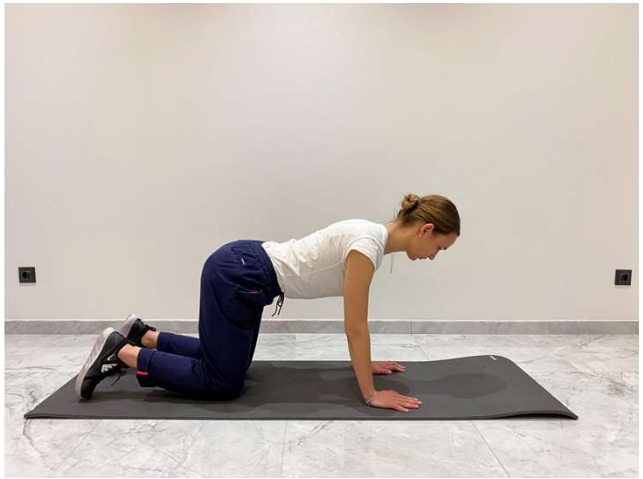

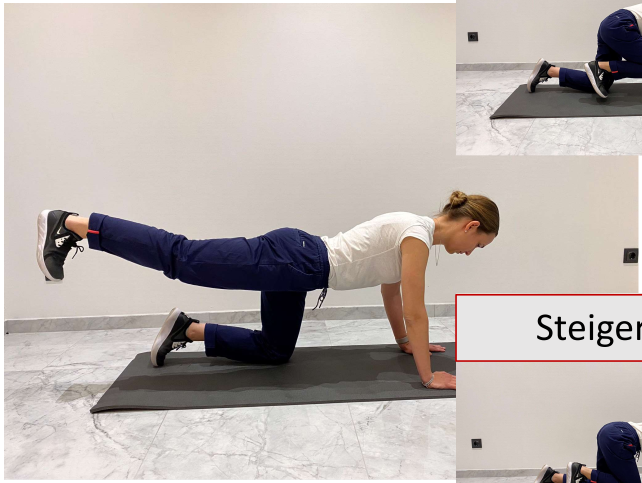

Steigerung 1

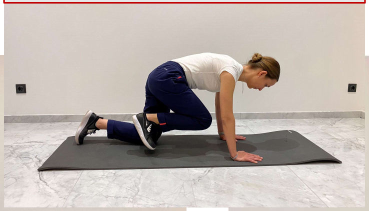

Steigerung 2

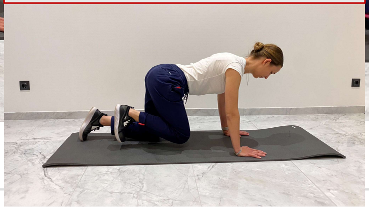

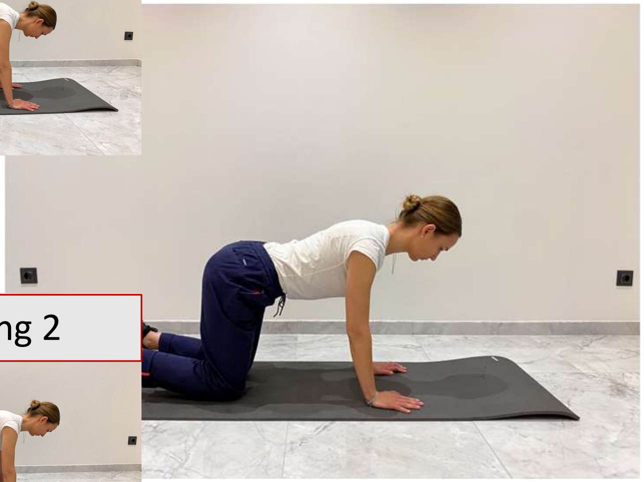

9 B

Vierfüßler-Stand abwechselnd das linke & rechte Bein nach hinten strecken

- Steigerung: & Knie Richtung Bauch ziehen, dann wieder abstellen;
  & Knie Richtung Bauch ziehen & WS beugen, dann wieder abstellen
- Möglicher FK: am Bauch liegend aus dem Bett aufstehen; Vorübung für aufstehen vom Boden, Stiegen steigen und Gehen

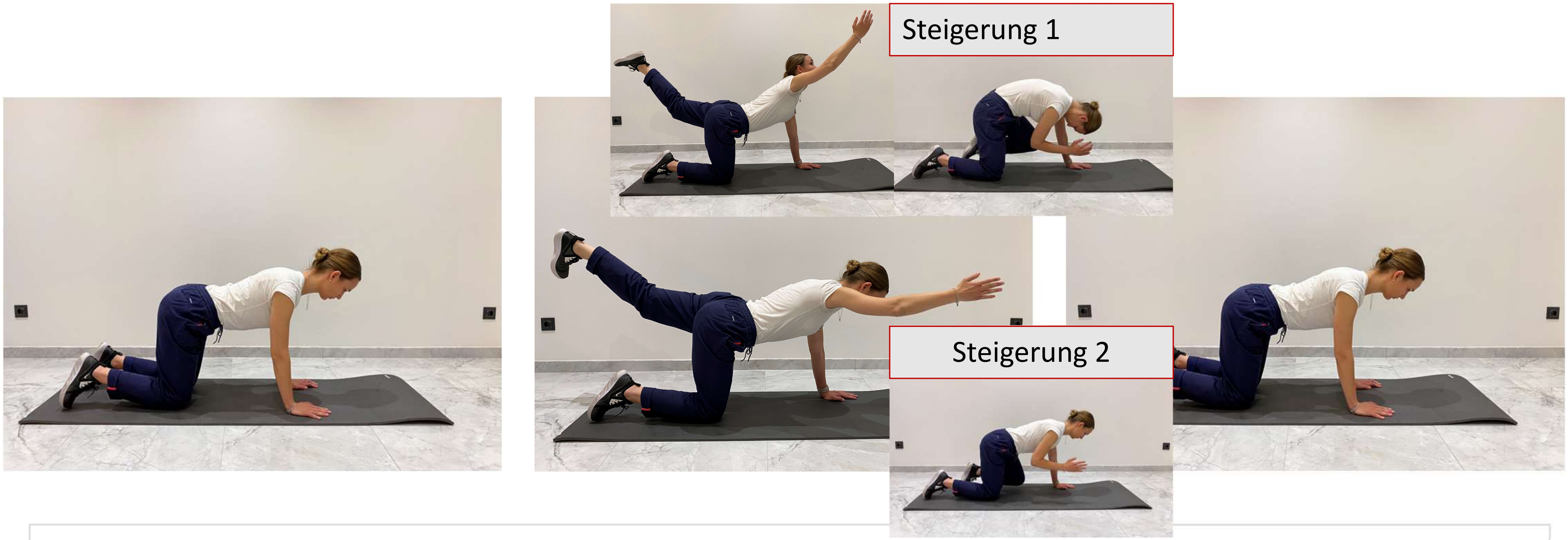

9
C

Vierfüßler-Stand den linken Arm und das rechte Bein ausstrecken bzw. re Arm und li Bein

- Steigerung:
  - & Ellbogen/Knie Richtung Bauch ziehen, dann wieder abstellen
  - Beim Ausstrecken die Wirbelsäule strecken, Beim Heranziehen die Wirbelsäule beugen und jeweils der Hand nachschauen.
- Möglicher FK: Vorübung für aufstehen vom Boden, Stiegen steigen, Gehen und komplexere Handlungen

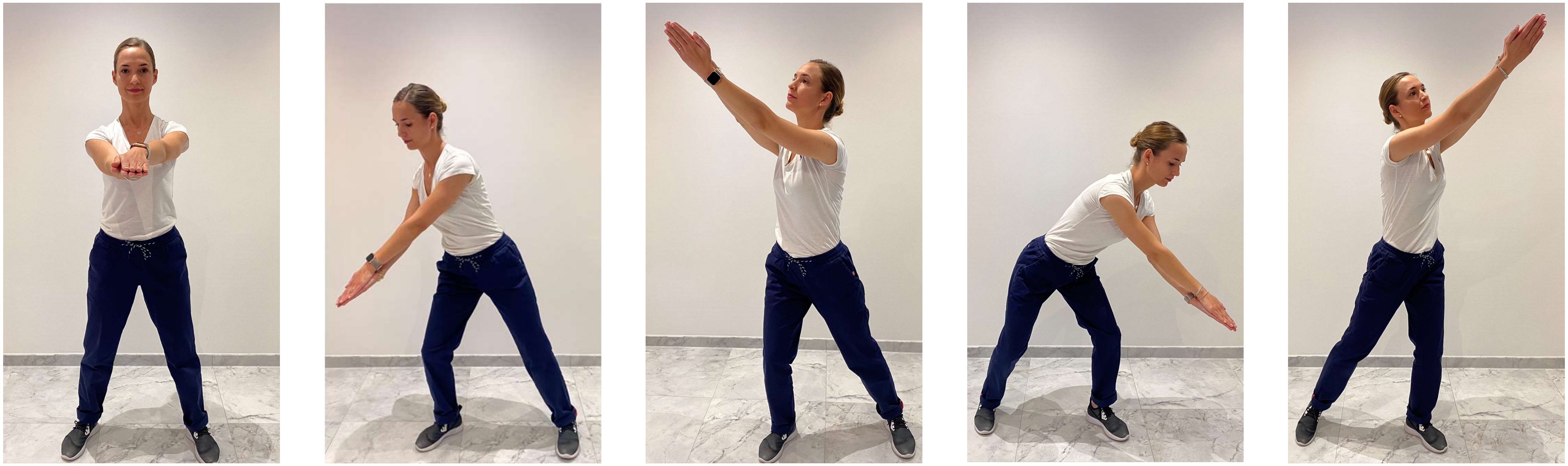

10 A

Im Stand: mit beiden Armen einen großen liegenden 8-er beschreiben, dabei Gewicht nach rechts/ links verlagern

- Variation: versch. Ausgangsstellungen (z.B. Stand breit/ schmal, Schrittstellung, etc.)
- Möglicher FK: Fenster putzen, große Gegenstände im Auto verladen, …

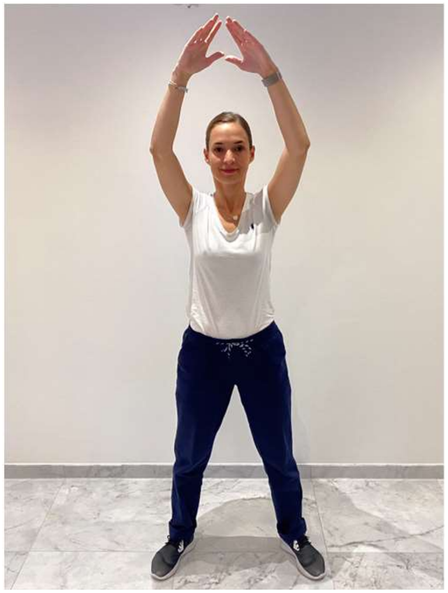
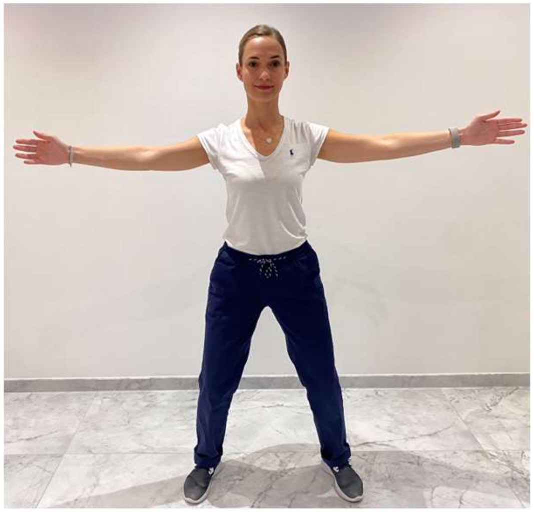
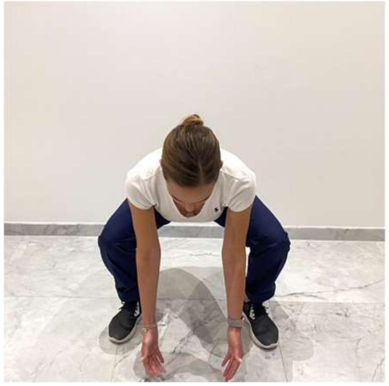
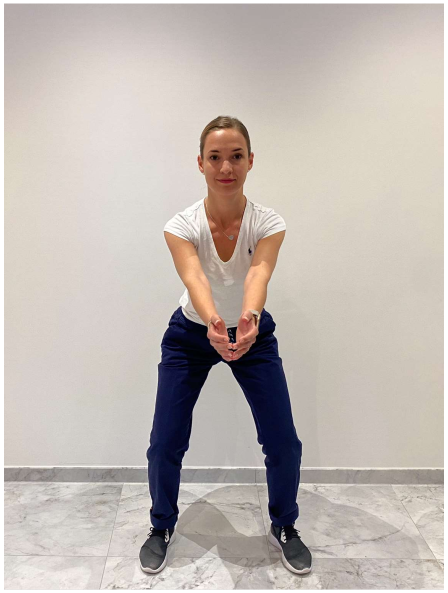

10 B

Im Stand: mit beiden Armen einen großen Kreis beschreiben, Fingerspitzen bis zum Boden und ganz nach oben strecken

- Variation: versch. Ausgangsstellungen (z.B. Stand breit/schmal, Schrittstellung, etc.)
- Möglicher FK: Gegenstände (z.B. Wäsche) vom Boden aufheben, …

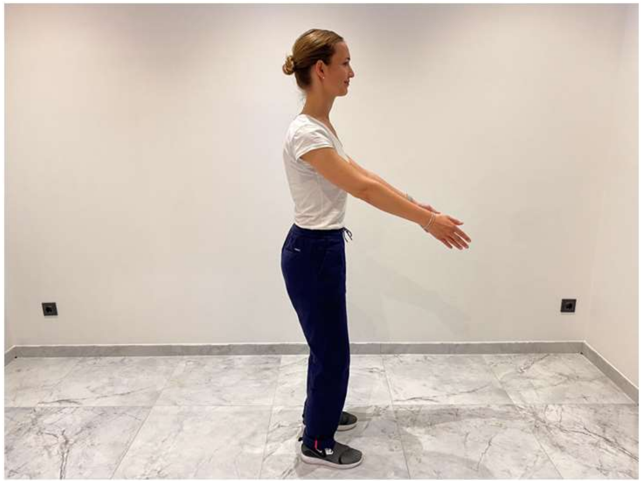

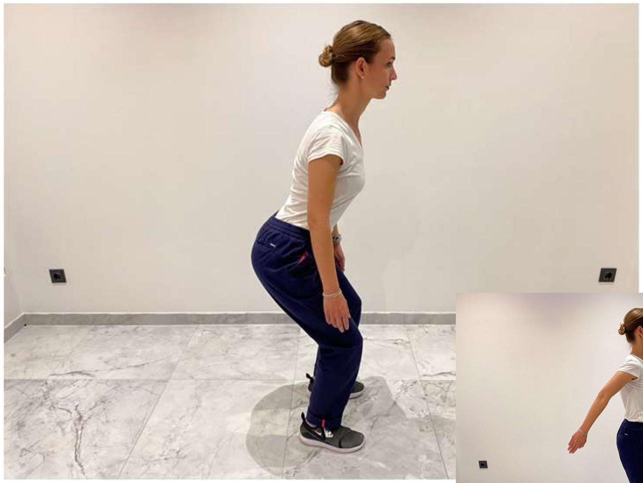

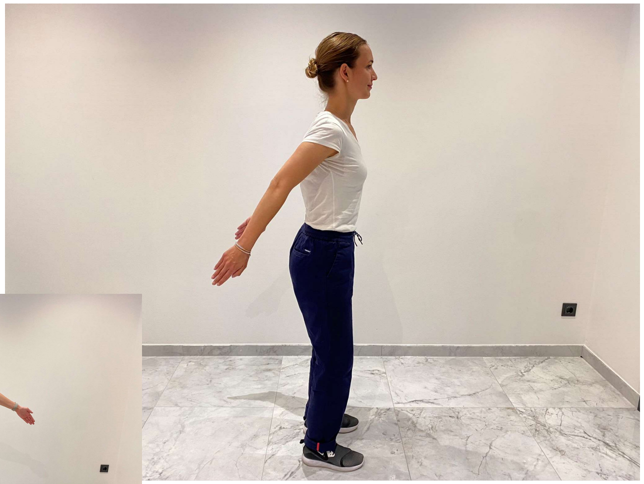

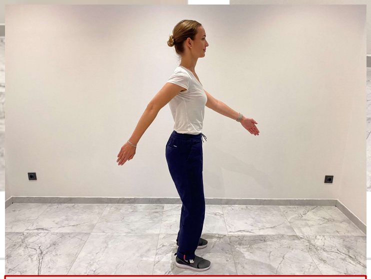

Variation

11 A

Breiter Stand: Knie wippen und Arme gleichzeitig schwingen

- Variation: Arme gegengleich schwingen
- Möglicher FK: diese Übung dient als Vorübung für das Gehen oder auch für das Tanzen, Langlaufen, etc.

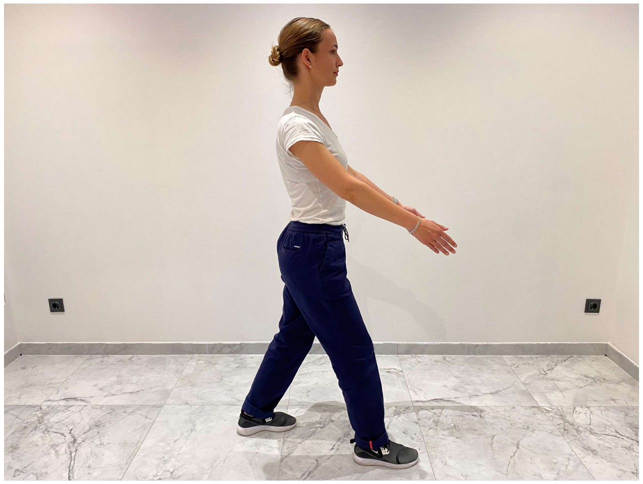
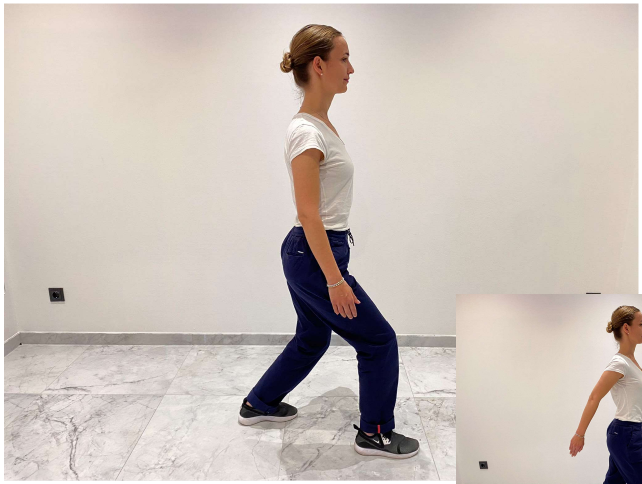
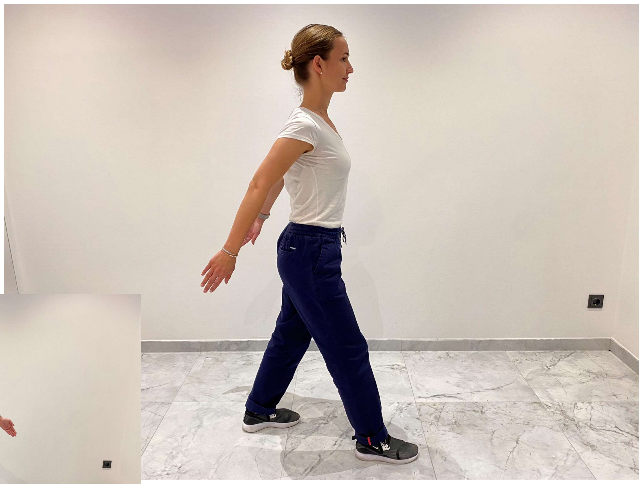
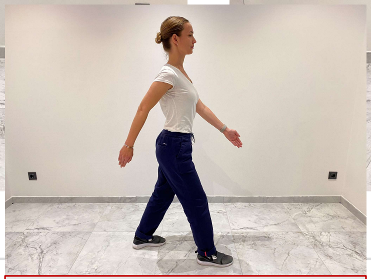

Variation

11 B

Schrittstellung: Knie wippen und Arme gleichzeitig schwingen

- Variation: Arme gegengleich schwingen
- Möglicher FK: diese Übung dient als Vorübung für das Gehen oder auch für das Tanzen, Langlaufen, etc.

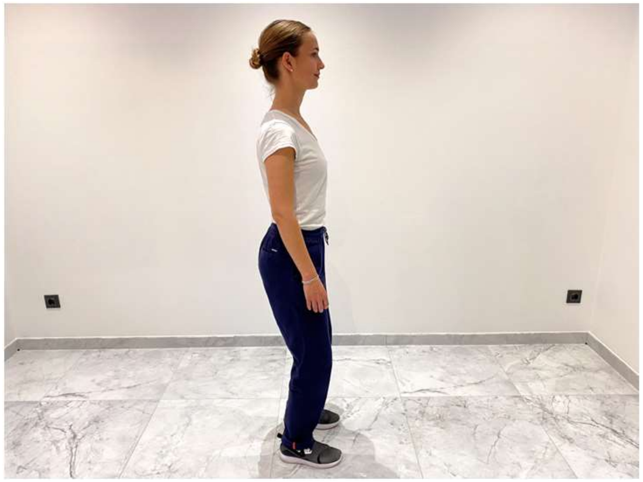

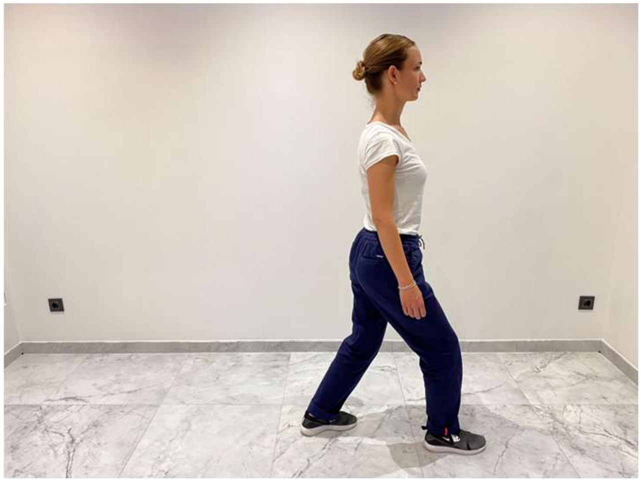

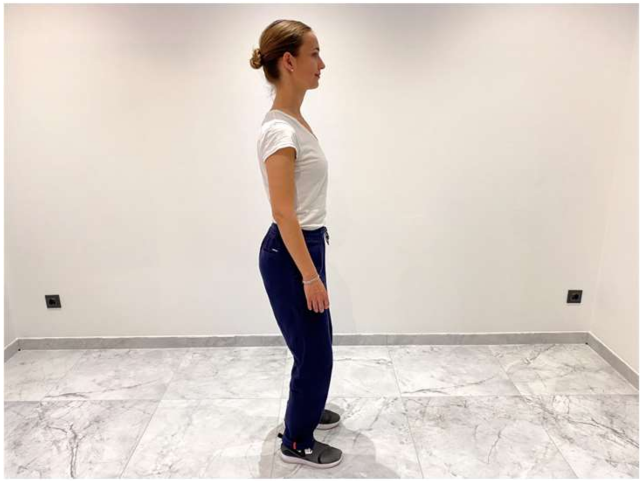

12
A

Ausfallschritte: nach vorne (mit linkem/ rechtem Fuß)

...und wieder zurück/zusammen (Füße nebeneinander)

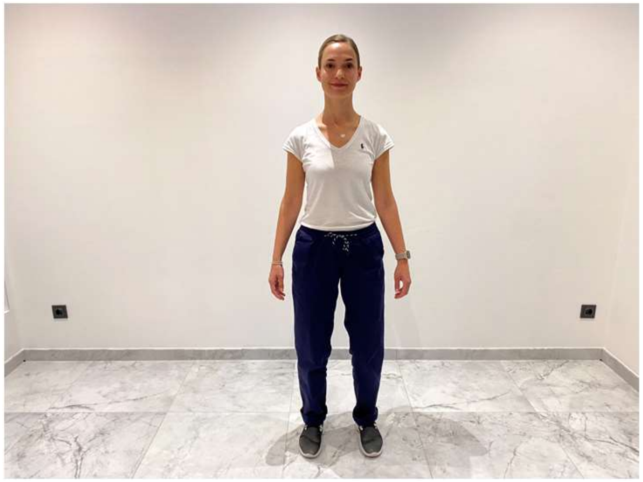
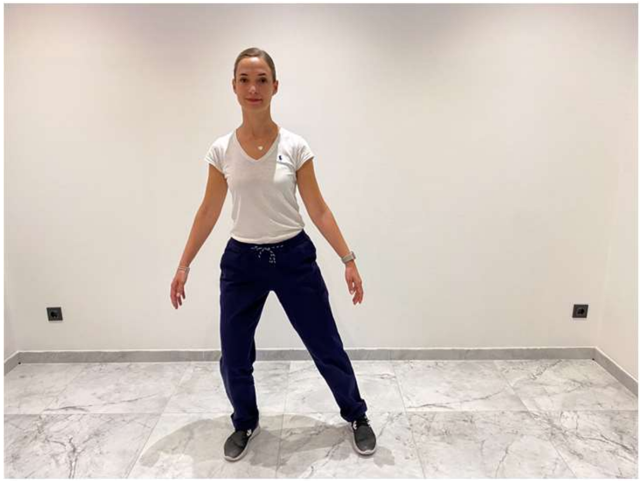
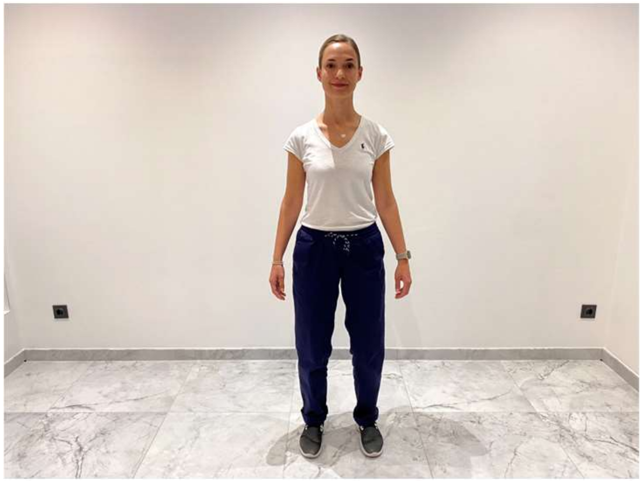

12
B

Ausfallschritte: zur Seite (mit linkem/ rechtem Fuß)

…mit linkem Fuß zur linken Seite

…mit rechtem Fuß zur rechten Seite

…und wieder zur Mitte/zusammen (Füße nebeneinander)

- Möglicher FK: Vorübung für Gehen und Schutzschritte; Tanzen

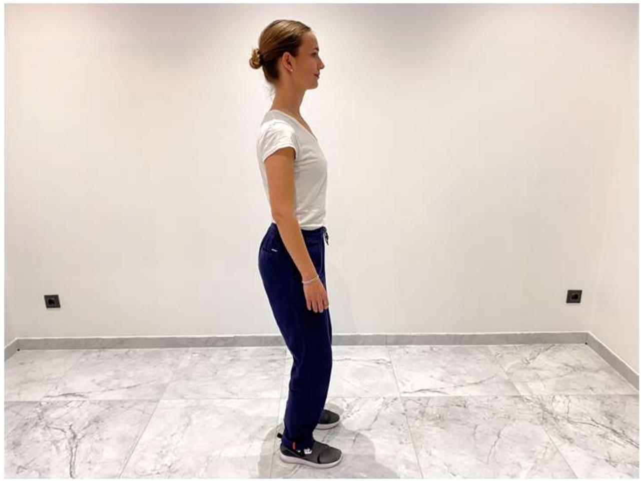

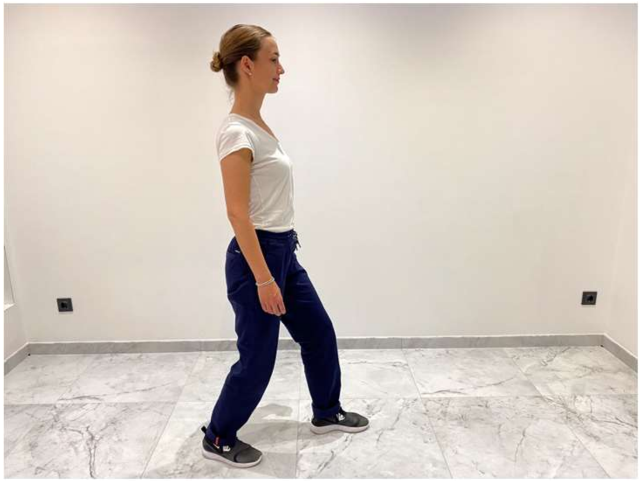

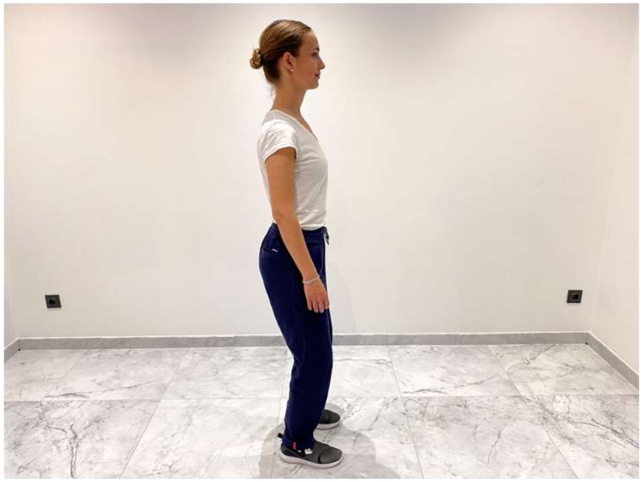

12
C

Ausfallschritte: nach hinten (mit linkem/ rechtem Fuß)

…und wieder nach vorne/zusammen (Füße nebeneinander)

- Möglicher FK: Vorübung für Gehen und Schutzschritte; Tanzen

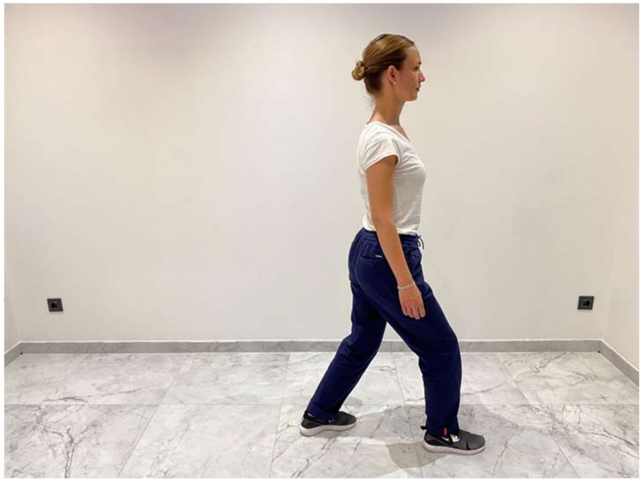

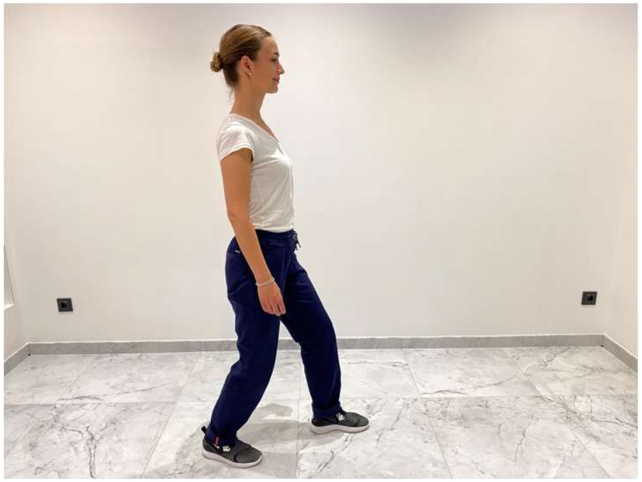

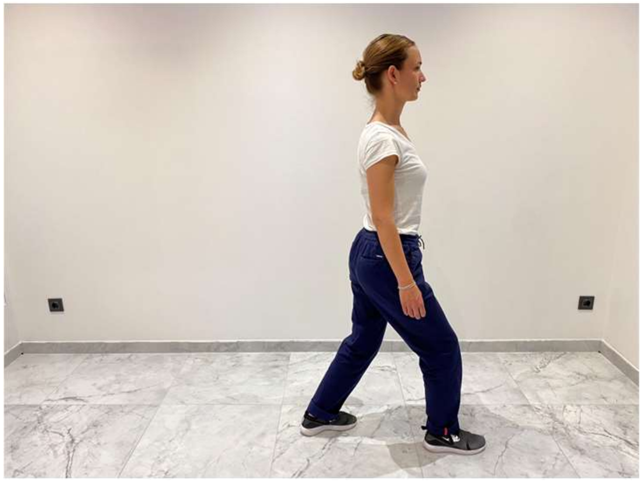

12
D

Ausfallschritte: von vorne nach hinten und wieder nach vorne (mit linkem/ rechtem Fuß)

- Möglicher FK: Vorübung für Gehen und Schutzschritte; Tanzen
- Variation/ Tipp: es können auch andere Ausfallschritte miteinander kombiniert werden: so ist z.B. zurück-seitlich-schließen und vor-seitlich-schließen der Grundschritt des Walzers

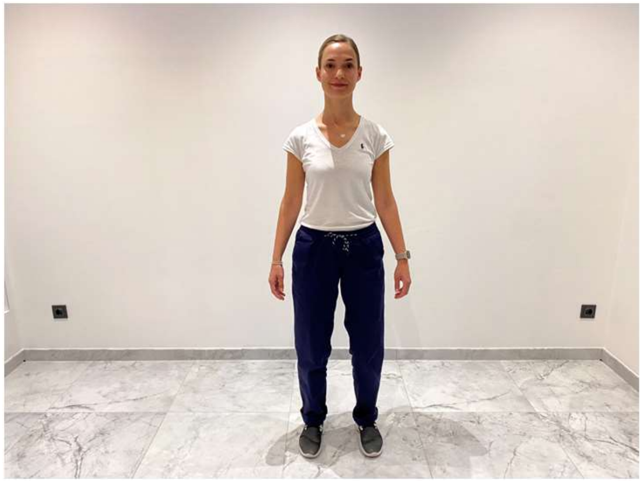

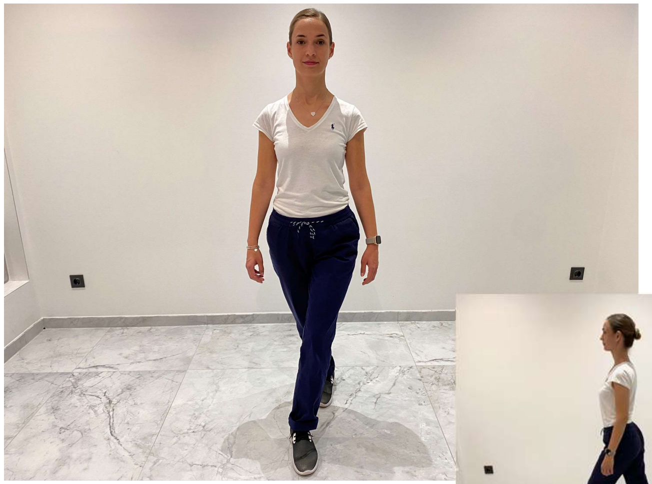

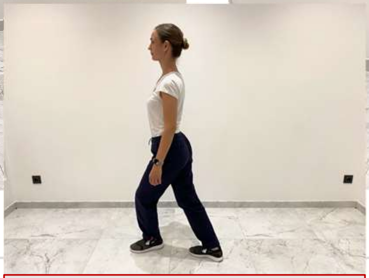

Seitliche Ansicht

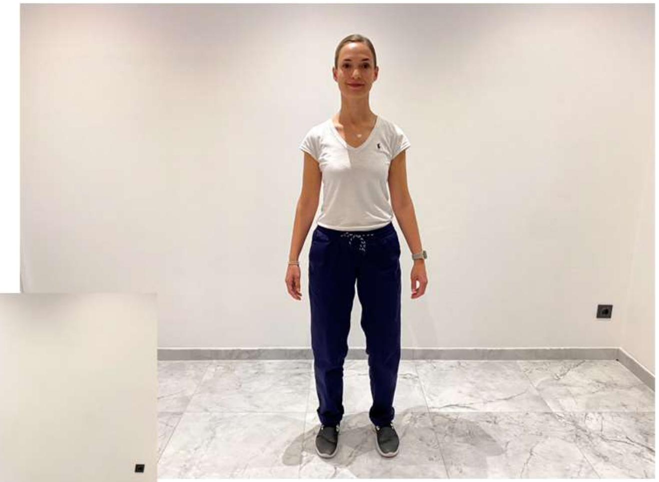

13 A

Kreuzschritte: nach vorne (mit linkem/rechtem Fuß)

…und wieder zurück/zusammen (Füße nebeneinander)

- Möglicher FK: Vorübung für Gehen und Schutzschritte; Tanzen

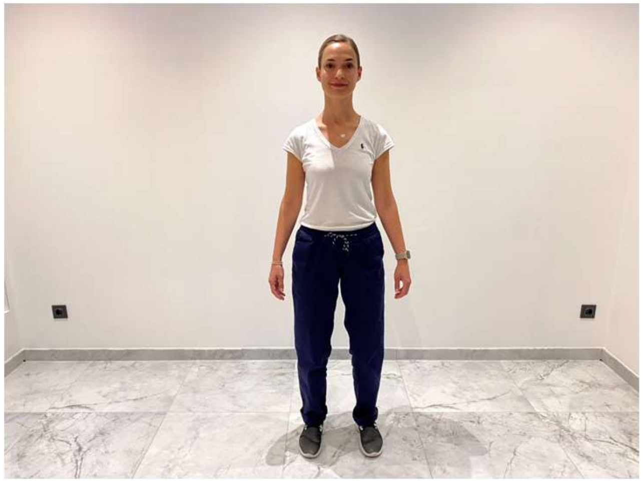

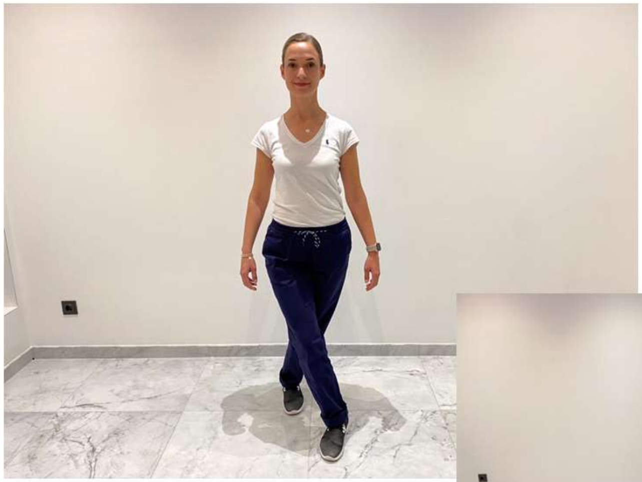

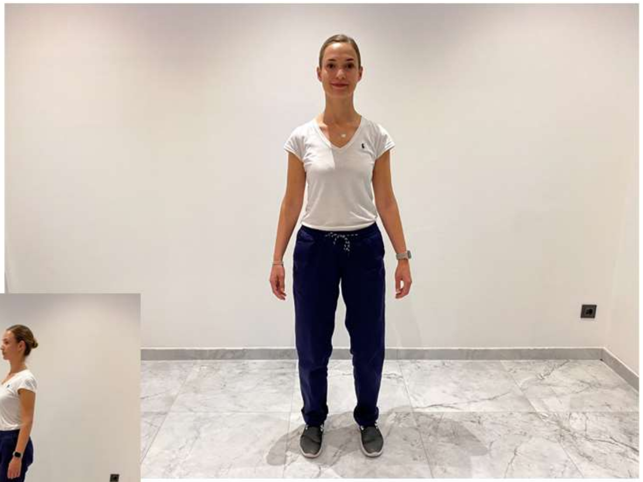

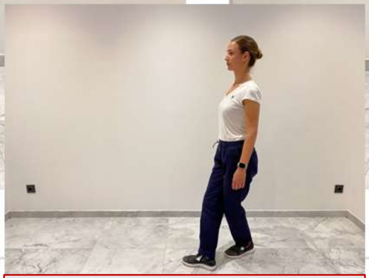

Seitliche Ansicht

13 B

Kreuzschritte: nach hinten (mit linkem/rechtem Fuß)

…und wieder nach vorne/zusammen (Füße nebeneinander)

- Möglicher FK: Vorübung für Gehen und Schutzschritte; Tanzen

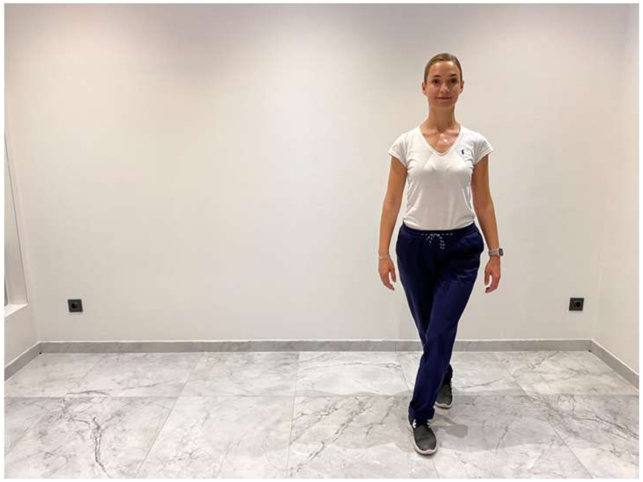

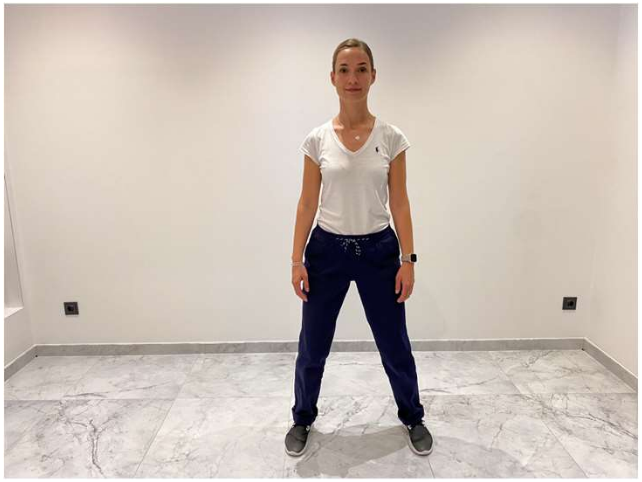

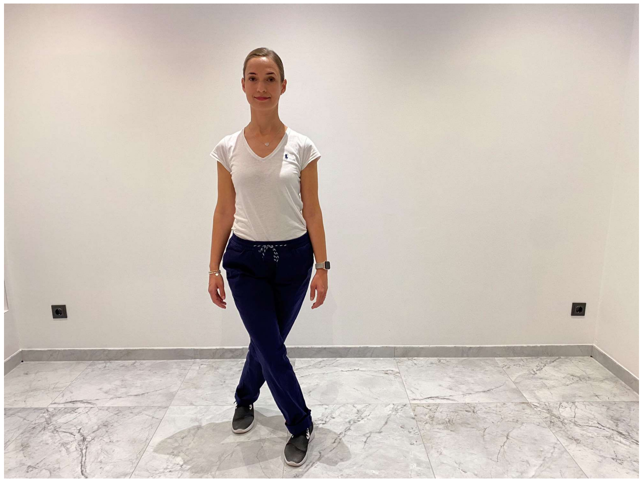

13
C

Kreuzschritte:
Kreuzschritt vorne –
Seitschritt –
Kreuzschritt hinten
(mit li/ re Fuß)

…mehrere WH, also eine ganze Länge mit den Kreuzschritten gehen

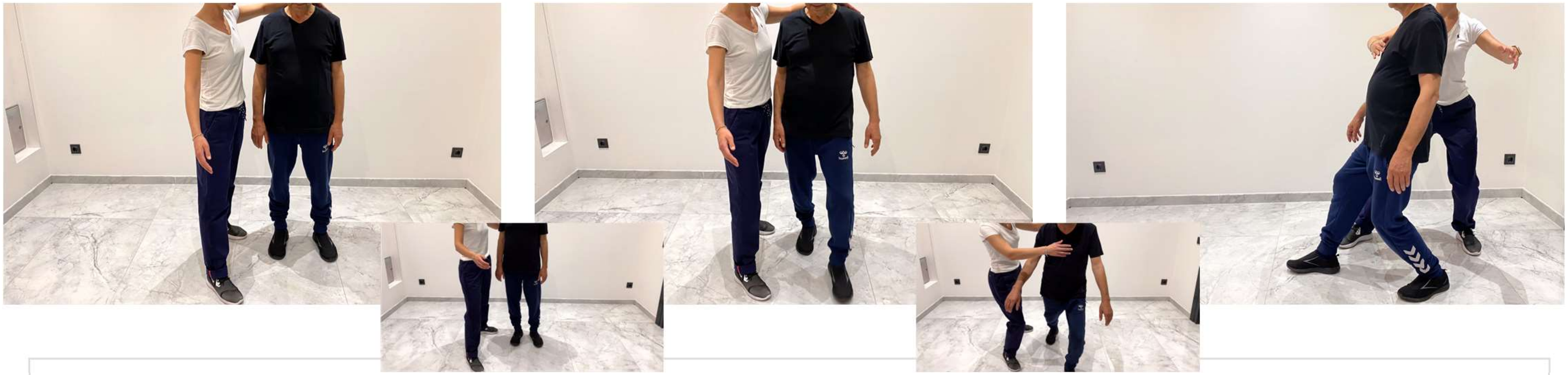

14 A

Nur mit Therapeut: Schutzschritte nach vorne/hinten

Therapeut schubst Patienten aus der Balance

Patient macht reaktiven Ausfallschritt nach vorne/hinten

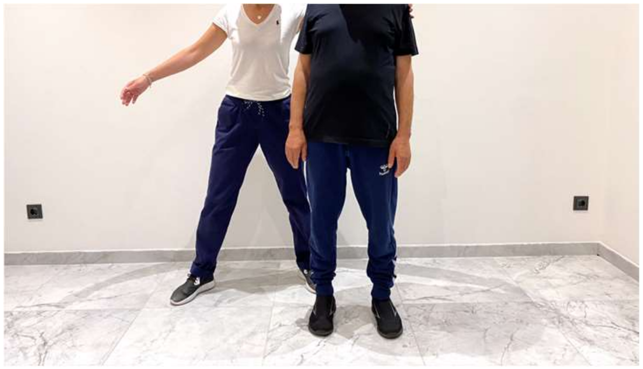

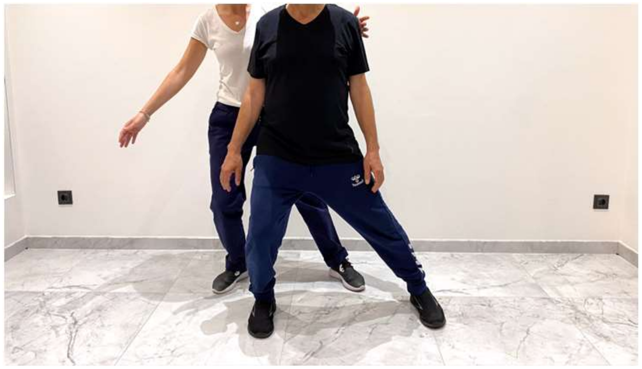

14
B

Nur mit Therapeut: Schutzschritte nach links/rechts

Therapeut schubst Patienten aus der Balance

Patient macht reaktiven Ausfallschritt nach links/rechts

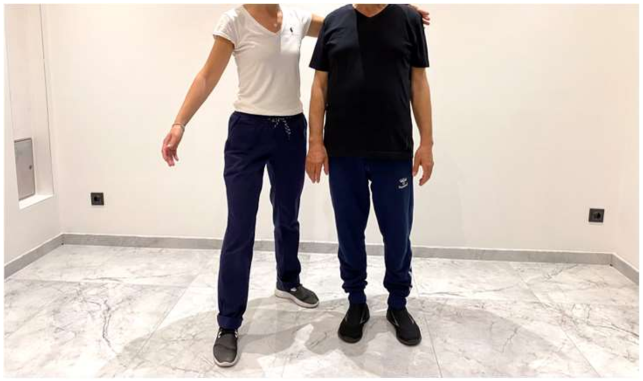 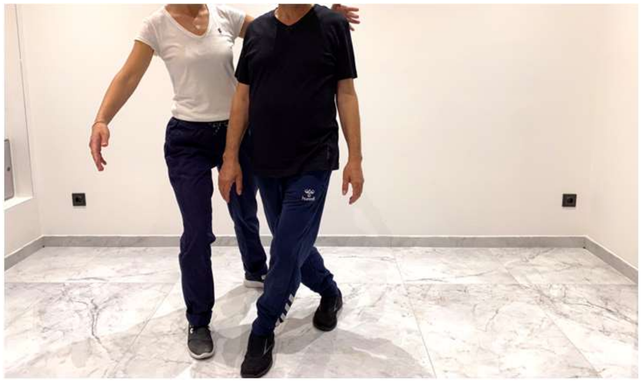

14 C

Nur mit Therapeut: Schutzschritte diagonal

Therapeut schubst Patienten aus der Balance

Patient macht reaktiven Kreuzschritt

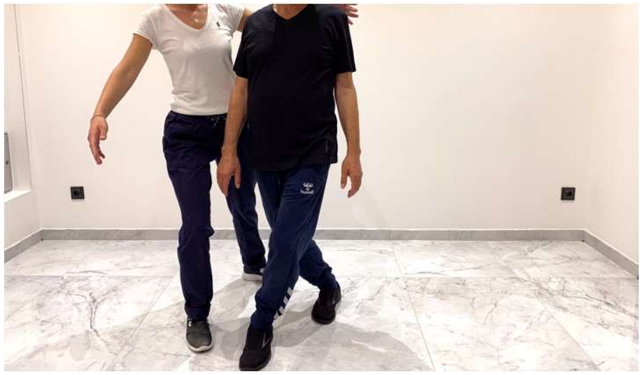
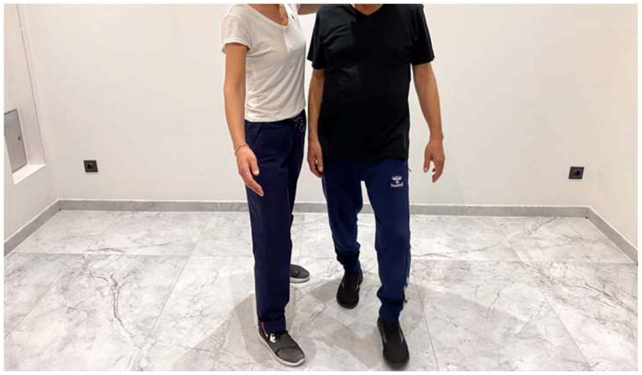
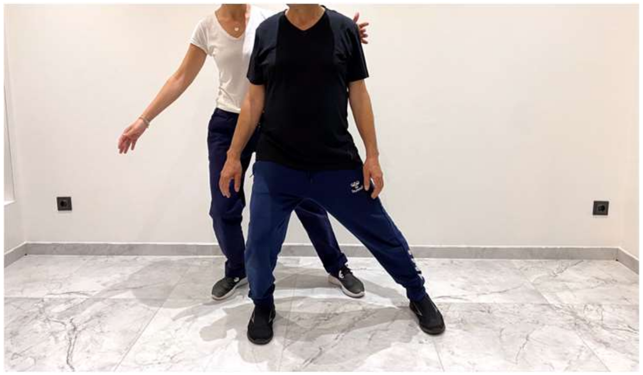

14
D

Nur mit Therapeut: Schutzschritte in alle Richtungen

Therapeut schubst Patienten aus der Balance

Patient macht reaktiven Ausfall- oder Kreuzschritt

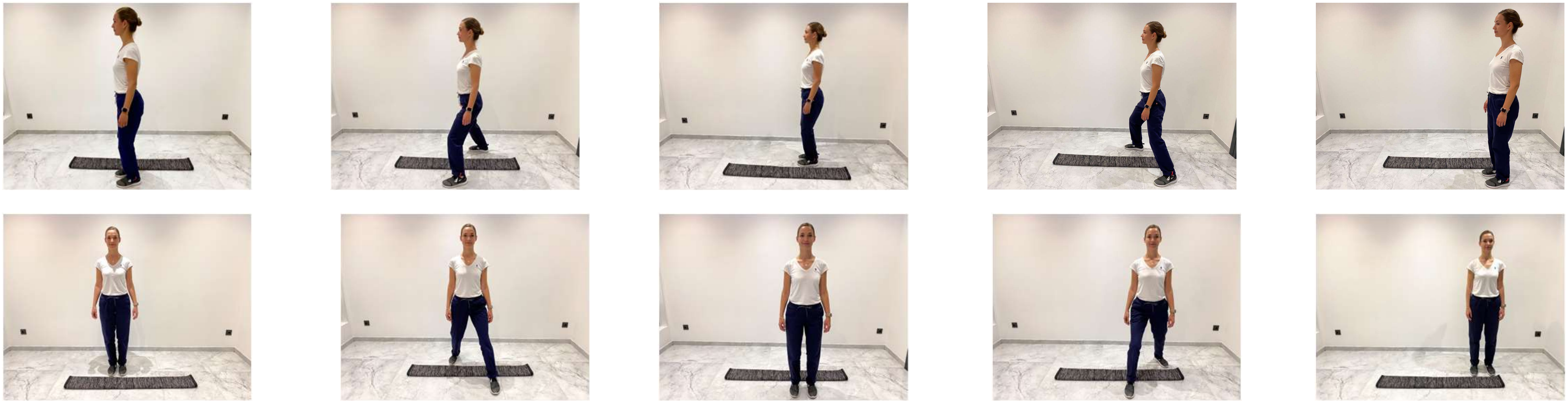

15
A

Linie übersteigen: seitlich oder vor/zurück

- Steigerung: Schritte im Wechsel mit Vorwärtsbewegung bis zum Ende der Linie
- Variationen: versch. breite Linien; langsam, schnell oder mit versch. Rhythmen (z.B. langsam/schnell/schnell)

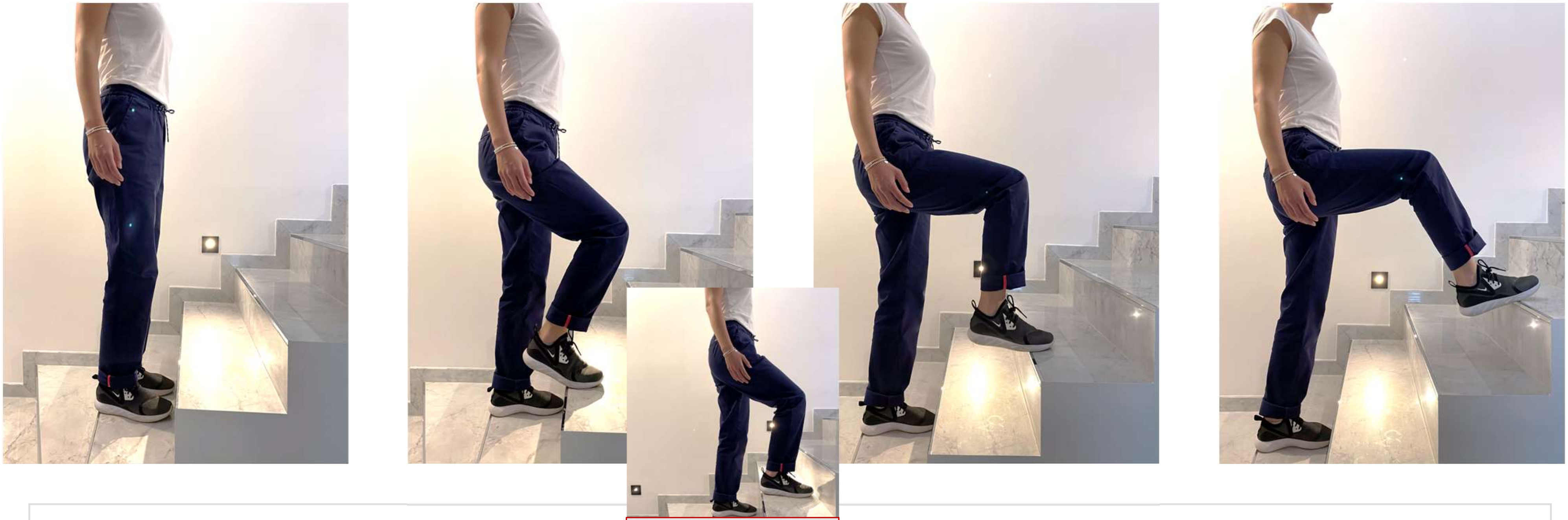

Variation

15 B

Stufe: rechten/ linken Fuß rauf und runter stellen

- Variationen: Stufen in versch. Höhen, an der Treppe: Fuß auf verschiedene Treppen stellen (z.B. 1./2./3./2./1. Stufe)

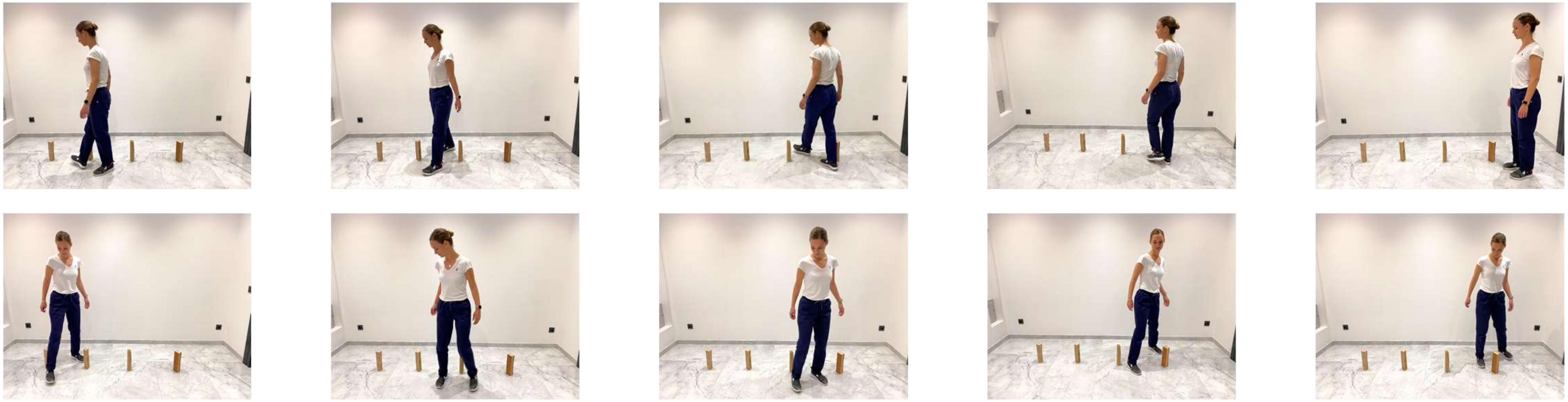

15 C

Slalom: gerade oder seitlich durchgehen

- Variation: großer/kleiner Abstand zwischen den Hütchen/Slalomstangen

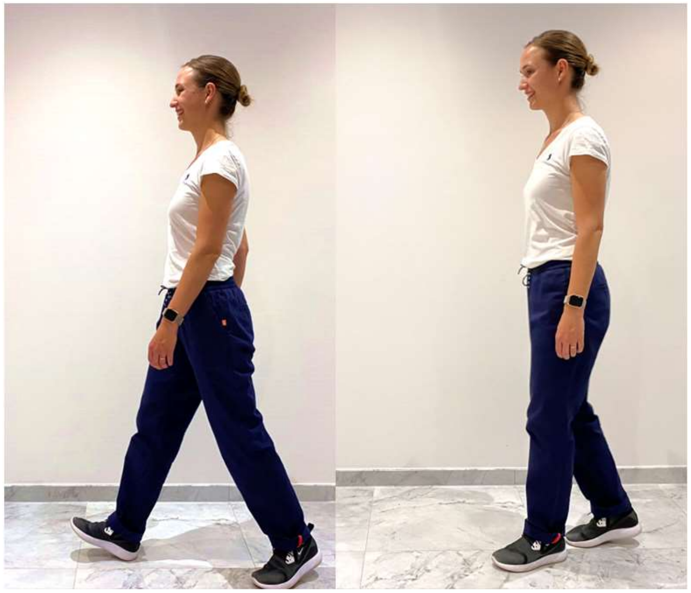
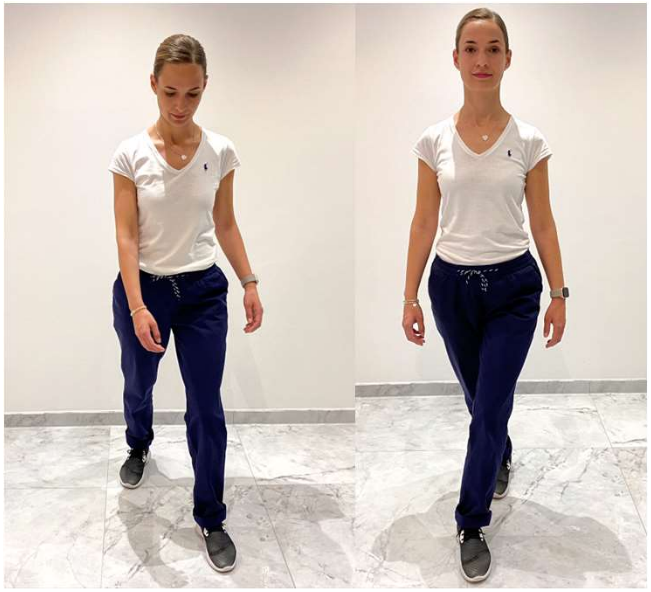
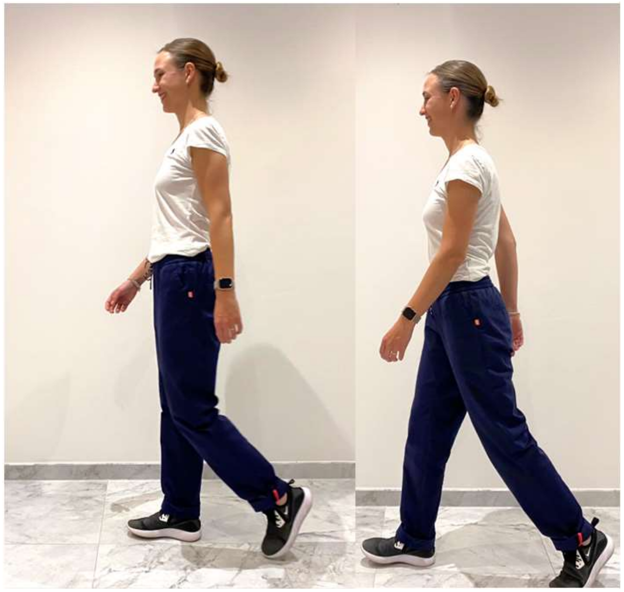

15
D

Gangvariationen

- Schrittlänge: kleine und große Schritte im Wechsel (Wechsel nach einem, drei, zehn, etc. Schritten)
- Spurbreite: schmale und breite Schritte im Wechsel (Wechsel nach einem, drei, zehn, etc. Schritten)
- Geschwindigkeit: langsame und schnelle Schritte im Wechsel (Wechsel nach einem, drei, zehn, etc. Schritten)

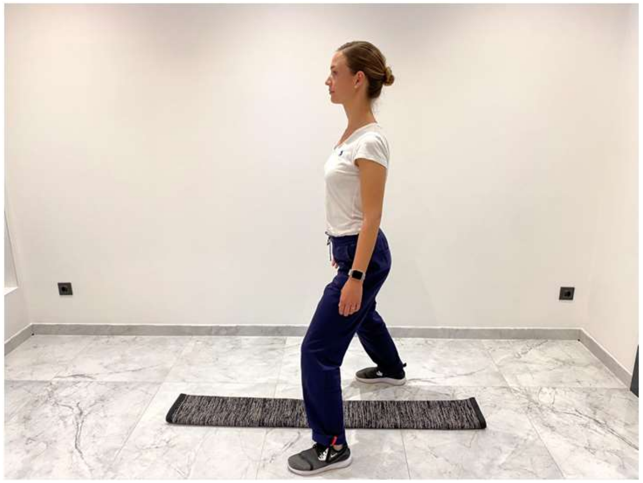

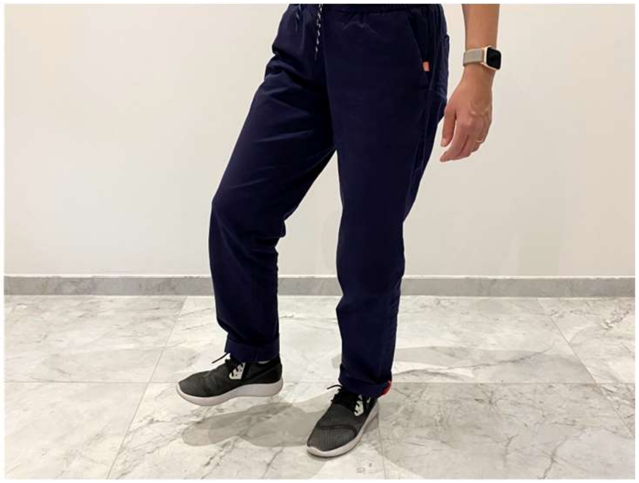

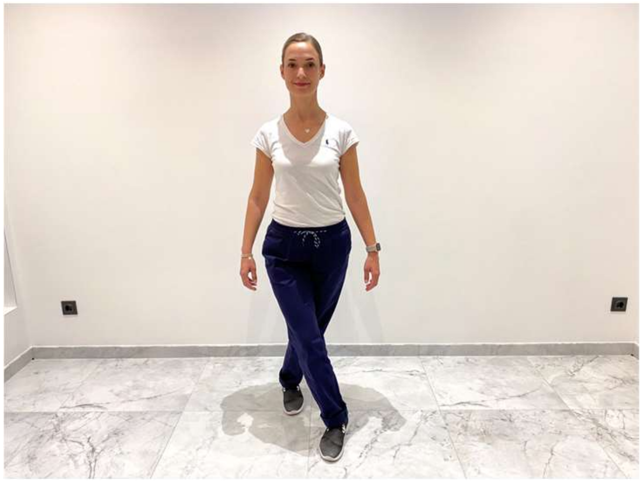

TIPP

Kombinieren Sie die Übungen 2, 12, 13, 14, 15 miteinander für mehr koordinative herausfordernde Möglichkeiten!

z.B.: 4x rechts tappen, 2x links tappen, Kreuzschritt rechts vorne, Kreuzschritt rechts hinten, Ausfallschritt links

VASC••age

Trampolin

Aufwärmen			
**Nr.**		**Übung**	**Anmerkung**
**T1**	**a**	Zweibeinstand (Augen geschlossen)	Übung mit leichtem Wippen, die Augen sind dabei geschlossen
	**b**	Zweibeinstand & Hüfte kreisen	Übung wird mit offenen Augen absolviert; mögliche Steigerung: schmaler Stand
	**c**	Zweibeinstand und auf Zehenspitzen gehen	Bei der Übung darf sich festgehalten werden. Der Patient geht auf die Zehenspitzen rauf und wieder runter.
**T2**	**a**	Zweibeinstand und Wippen (mit Halten)	Knie sind dabei leicht gebeugt. Nur federn, kein Abheben der Füße vom Trampolinnetz und mit Zuhilfenahme der Haltevorrichtung. Spurbreite kann variiert werden.
	**b**	Zweibeinstand und Wippen (ohne Halten)	Knie sind dabei leicht gebeugt. Nur federn, kein Abheben vom Netz und ohne Zuhilfenahme der Haltevorrichtung. Spurbreite kann variiert werden.
**T3**	**a**	Tandemstand und Wippen (mit Halten)	1 Fuß steht vorne einer hinten, Knie sind leicht gebeugt. Nur federn, kein Abheben vom Netz, mit Zuhilfenahme der Haltevorrichtung. Schrittlänge kann variiert werden.
	**b**	Tandemstand und Wippen (ohne Halten)	1 Fuß steht vorne einer hinten, Knie sind leicht gebeugt. Nur federn, kein Abheben vom Netz, ohne Zuhilfenahme der Haltevorrichtung. Schrittlänge kann variiert werden.

VASC••age

Trampolin

Training			
**Nr.**		**Übung**	**Anmerkung**
**T4**		Gehen auf der Stelle	Die Füße abwechselnd heben, wie beim Marschieren.
**T5**	**a**	Gehen vor/zurück	Am Trampolin Schritte vor und zurück gehen
	**b**	Gehen links/rechts	Am Trampolin Seitschritte nach links und rechts machen
	**c**	Gehen und Ball ausweichen	Ein Ball (vorzugsweise Tennisball) auf das Trampolin rollen, der Ball bewegt sich durch das Trampolin und der Patient muss dem Ball ausweichen, um nicht drauf zu steigen.
**T6**	**a**	Wippen und leicht Springen (mit Halten)	leichtes Abheben vom Netz/Springen mit Zuhilfenahme der Haltevorrichtung.
	**b**	Wippen und leicht Springen (ohne Halten)	leichtes Abheben vom Netz/ Springen (auf der Stelle) ohne Zuhilfenahme der Haltevorrichtung
**T7**	**a**	Wippen und leicht Springen (auseinander und zusammen)	leichtes Abheben vom Netz/ Springen ohne Zuhilfenahme der Haltevorrichtung, wobei schrittweise weiter auseinandergesprungen wird (so weit wie möglich) und dann wieder schrittweise zusammen.
	**b**	Wippen und leicht Springen (links/ rechts)	leichtes Abheben vom Netz/ Springen ohne Zuhilfenahme der Haltevorrichtung, wobei der Patient beidbeinig soweit wie möglich (Trampolingröße) nach rechts springt und dann wieder nach links.

VASCage

Trampolin

	**c**	Wippen und leicht Springen (vor/zurück)	leichtes Abheben vom Boden/ Springen ohne Zuhilfenahme der Haltevorrichtung wobei der Patient beidbeinig soweit wie möglich (Trampolingröße) nach vorne springt und dann wieder nach hinten.
	**d**	Wippen und leicht Springen (im Kreis)	leichtes Abheben vom Boden/ Springen ohne Zuhilfenahme der Haltevorrichtung, wobei der Patient beidbeinig springt und sich dabei schrittweise einmal im Kreis dreht.
**T8**	**a**	Tandemstand: Sprünge vom vorderen auf den Hinteren Fuß	1 Fuß steht vorne einer hinten, Knie sind leicht gebeugt. Leichtes Abheben vom Boden/ Springen auf den vorderen und hinteren Fuß ohne Zuhilfenahme der Haltevorrichtung. Schrittlänge kann variiert werden.
	**b**	Zweibeinstand: Sprünge vom linken auf den rechten Fuß	Knie sind leicht gebeugt. Leichtes Abheben vom Boden/ Springen auf den linken und rechten Fuß ohne Zuhilfenahme der Haltevorrichtung. Spurbreite kann variiert werden.
**T9**	**a**	1-Bein-Stand	Stehen auf einem Bein, Knie ist dabei leicht gebeugt, probieren das Gleichgewicht zu halten (=Vorübung für T9 b+c)
	**b**	1-Bein-Stand und wippen	Stehen auf einem Bein, Knie ist dabei leicht gebeugt, und in das Trampolin hineinfedern
	**c**	1-Bein-Stand und springen	Stehen auf einem Bein, Knie ist dabei leicht gebeugt, und leicht vom Trampolin abspringen. Bestenfalls am Gleichen Fuß wieder landen und mehrmals wiederholen.

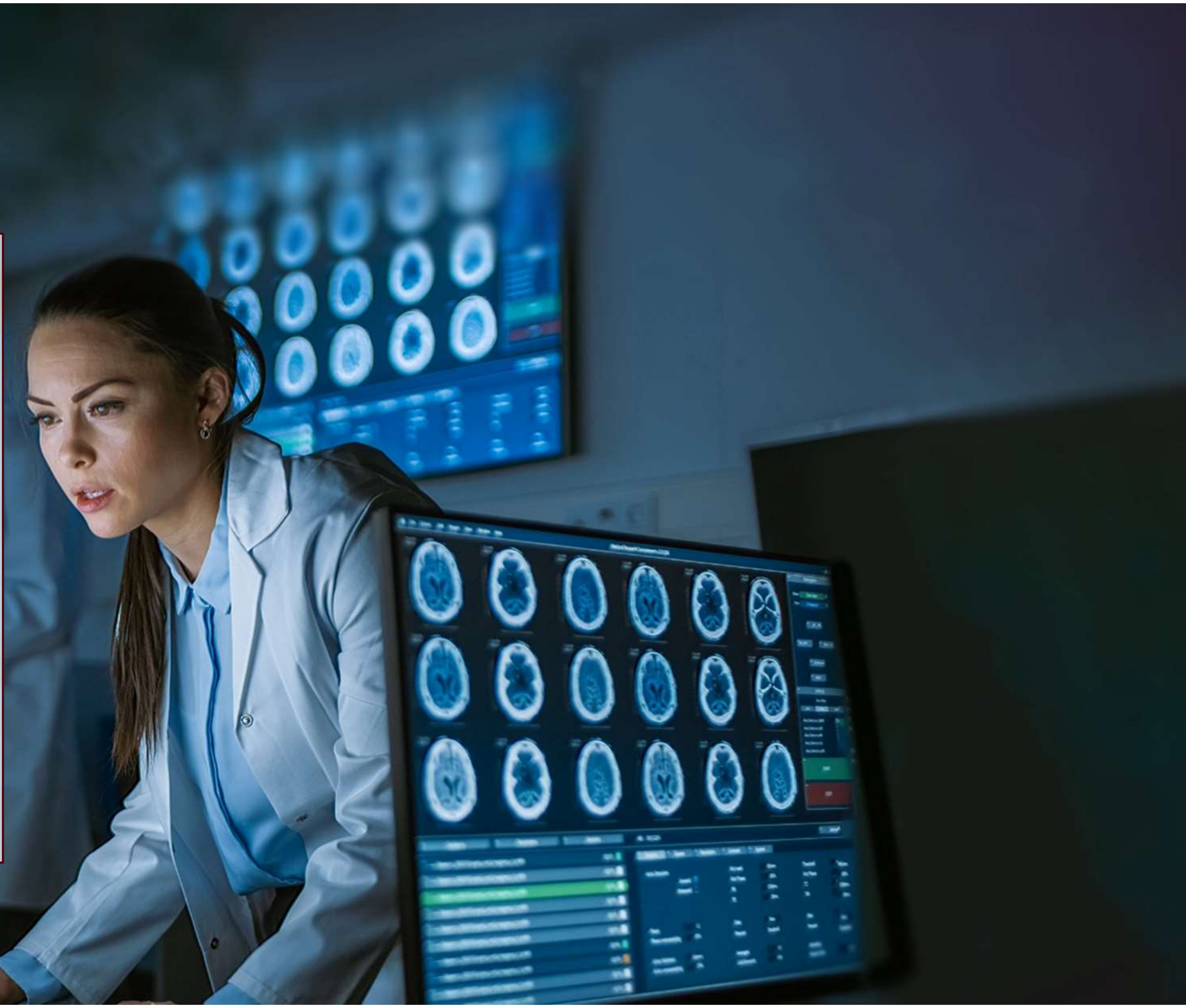

VASCage

The COMET-Centre VASCage is funded within the

COMET Programme - Competence Centres for Excellent Technologies

by

- Austrian Ministry for Climate Action, Environment, Energy, Mobility, Innovation and Technology
- Austrian Ministry of Labour and Economy

and the federal states

- Tyrol
- Salzburg
- Vienna

The COMET Programme is conducted by the Austrian Research Promotion Agency (FFG).

Federal Ministry Republic of Austria Labour and Economy

wirtschafts agentur wien

Tirol STANDORTAGENTUR TIROL

FFG Promoting Innovation.

LAND SALZBURG

Federal Ministry Republic of Austria Climate Action, Environment, Energy, Mobility, Innovation and Technology
