# Supplementary material for: Recovery of balance and walking in people with ataxia after acute cerebral stroke: study protocol for a prospective, monocentric, single-blinded, randomized controlled trial
Source: Front Stroke. 2024 Aug 5;3:1388891. doi: 10.3389/fstro.2024.1388891 (PMC12802608; doi:10.3389/fstro.2024.1388891)
Supplement: Supplementary file 4 [file Data_Sheet_4.PDF]

TIDieR

Standard physiotherapy

Details: Standard physiotherapy incorporates standard exercises of stroke rehabilitation according to national guidelines. Categories of exercises were assessed for the pilot trial and confirmed for this RCT by using a questionnaire in the participating study centers.

Why: Standard physiotherapy was defined as control group to see if coordination exercises have the same, a better, or worse effect than usual care.

What (material): For some exercises, an exercise mat, a balance pad, assistive devices, or everyday objects are needed.
Patients receive their individual home exercise program as a handout. Exercises are selected from the exercise program (supplementary material).

What (procedures): Exercises in this group should be relevant to everyday life and therefore focus on training of Activities of Daily Living. Even tough complex movements and activities are trained, exercises should be repeated an appropriate number of times. Exercises are adapted to the patient's limit of performance. However, if necessary, the patient can be supported by the therapist or an assistive device. Besides the training of Activities of Daily Living, balance training, stability training and strengthening of the trunk is of importance in this group.

Exercises in this group can be classified into four groups:

1. Training of trunk stability, including core strengthening and segmental stabilization.
2. Training of Activities of Daily Living (ADL), especially concerning mobility (e.g., lie-to-sit, sit-to-stand) and self-care (e.g. dressing, washing, etc.). Activities can be performed with or without aids.
3. Walking training, involving the variation of step length/step width/ walking speed, stair climbing, and walking on uneven ground. Training can be performed with an assistant or assistive device.
4. Balance training, incorporating exercises with balance pads, exercises for shifting the body center of gravity and exercises with reduced support-surface.

Who provided: The intervention is delivered by physical therapists of the participating study centers. Staff at each center has been trained in live and online sessions on the details of the intervention and general study requirements. The online sessions (recordings) are available on demand throughout the study. The intervention is carried out as part of their daily routine work.

How (mode of delivery; individual or group): The intervention is conducted in a one-to-one setting in face-to-face contact. Supervision of the intervention during home exercises is done indirectly via telephone.

Where: Patients are recruited at the Clinical Department of Neurology, Medical University of Innsbruck, Austria (Center 1), a large medical center with a Stroke Unit. Supervised interventions start in this acute care setting and are continued at the rehabilitation hospital Landeskrankenhaus Hochzirl-Natters, Austria (Center 2) or the Clinic for Rehabilitation Münster, Austria (Center 3), Patients are transferred during the supervised sessions, because of routine procedure in Tyrol, where patients receive intensive multidisciplinary rehabilitation in a rehabilitation hospital as early as possible.

When and how much: Patients receive 4 weeks of supervised training. In addition, patients are asked to train independently for 15 minutes, 5 times per week (total 20x). The exercises for each day will be provided by the therapist conducting the supervised session and selected according to the patient´s level of performance.

Form saved

After completion of the supervised treatments, patients are asked to practice independently for 5 times/ week (15 minutes each day) for 8 weeks i.e., until T2 assessment (total 40x). Patients practice according to an individualized home exercise program, which they receive from the therapist conducting the supervised sessions. Patients can choose out of these exercises to conduct their independent practice.

Tailoring:

Adjusting the difficulty and intensity of the exercises to the patient's level of performance is a basic principle of therapeutic treatment and will be performed individually for the patient whenever necessary.

How well (planned):

To record the supervised session, therapists are provided with an intervention documentation sheet. To record the independent practice sessions, patients receive a compliance checklist. To record falls during unsupervised practice, patients are provided with a falls protocol. For improving adherence, patients receive semi-structured phone call interviews every 2 weeks. The interview covers questions regarding exercise frequency, experience of any problems related to the exercises, including the exercise environment and motivation, and regarding completion of compliance and falls protocol.

Form saved
