# Supplementary material for: Recovery of balance and walking in people with ataxia after acute cerebral stroke: study protocol for a prospective, monocentric, single-blinded, randomized controlled trial
Source: Front Stroke. 2024 Aug 5;3:1388891. doi: 10.3389/fstro.2024.1388891 (PMC12802608; doi:10.3389/fstro.2024.1388891)
Supplement: Supplementary file 5 [file Data_Sheet_5.PDF]

VASC • • age

GLAAS II
Standard physiotherapy for ataxia[1]

Patricia Meier

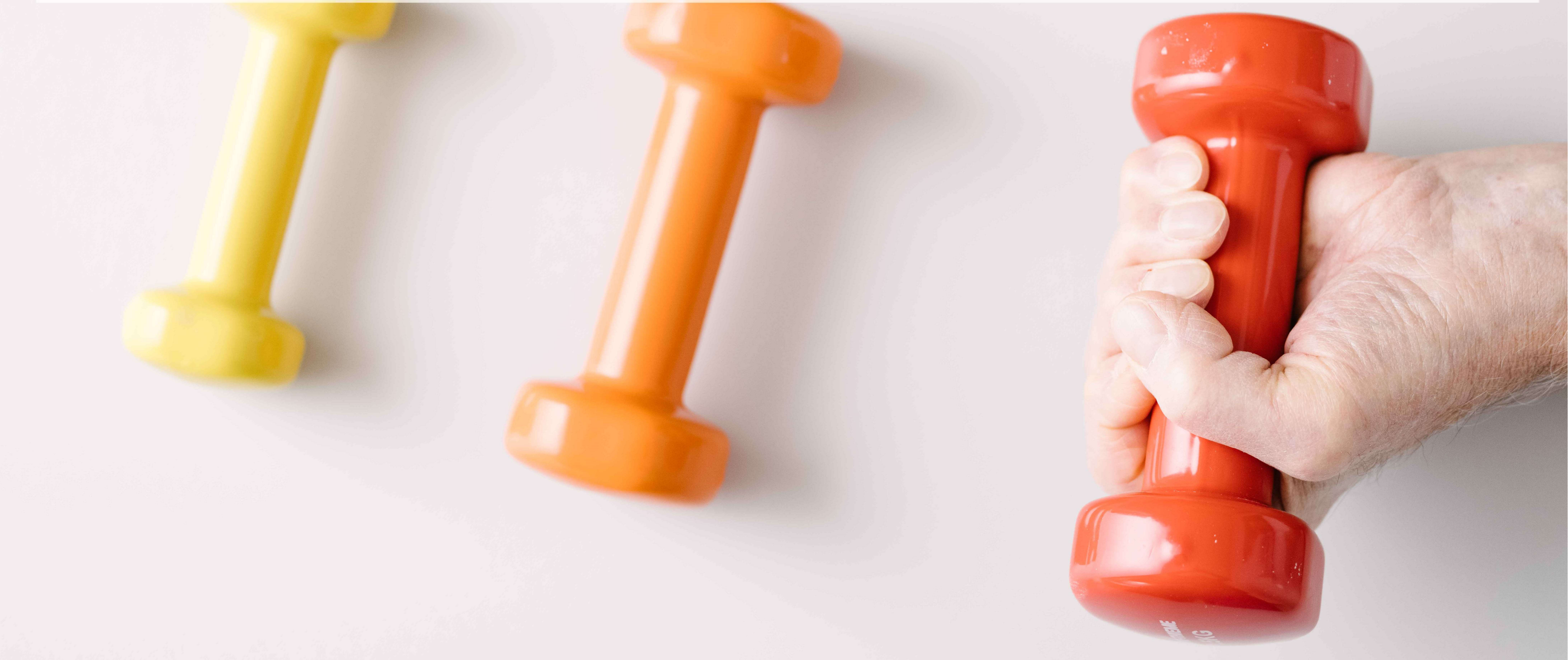

©Rene Böhmer

[1] Exercises based on national stroke guidelines of the USA and UK and questionnaires in the study centers of Tyrol

Principles of training

- Goals and exercises in therapy should be relevant to everyday life.
- Exercises focus on an activity level and involve complex movements such as transitioning from a lying position to standing.
- Exercises should nevertheless be repeated an appropriate number of times.
- Exercise difficulty should be adapted to the patient's limit of performance.
- Patients can be supported by the therapist or an assistive device to be able to do the exercise.
- Emphasize trunk stability and strengthening of the trunk.
- 20 supervised sessions: 5 per week for 45 minutes & 60 sessions of independent training: 5 per week for 15 minutes.

Exercise categories

- **Trunk stability training:**
  strengthening, segmental stabilization.
- **Training of activities of daily living (ADL):**
  training everyday activities for mobility and self-care, like dressing, washing, changing body position (e.g., lie-to-sit, sit-to-stand) with aids if necessary.
- **Walking training:**
  walking with an assistant or assistive device, variation of step length/ step width/ walking speed, stair climbing, walking on uneven ground.
- **Balance training:**
  using balance pads, exercises for shifting the body center of gravity, exercises with reduced support-surface.

Increasing difficulty (1)

- Trunk stability training:
  Start with exercises for segmental stabilization of the torso while lying, then:
  - While sitting, standing, walking
  - Include movement of arms or legs while keeping the trunk stable
  - Only increase if the patient can remain stable in the trunk!
- Training for "Activities of daily living":
  - Changing body positions starting from a lying position (turning to left/ right side), lie-to-sit, sit-to-stand, walking - with aids if necessary
  - ADL´s in lying position, seated, standing (e.g., brushing teeth)
  - From simple to complex activities

Increasing difficulty (2)

- Balance training:
  - Exercises for shifting the body center of gravity in lying position, seated, standing
  - Using balance pads
  - Reduced support-surface
  - The focus is on static balance!
- Gait training:
  - With assistant, assistive device, independently
  - With variation of step length/ step width/ walking speed
  - Obstacle course, stair climbing, walking on uneven ground

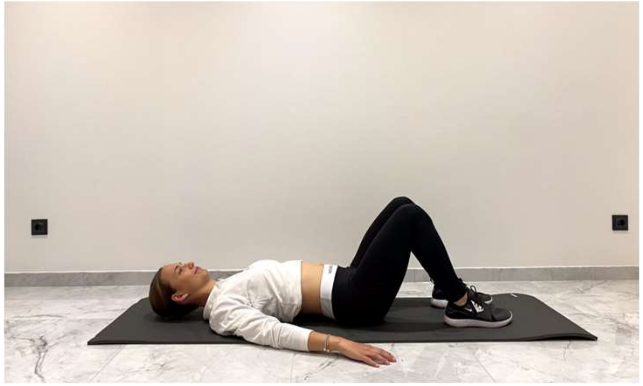

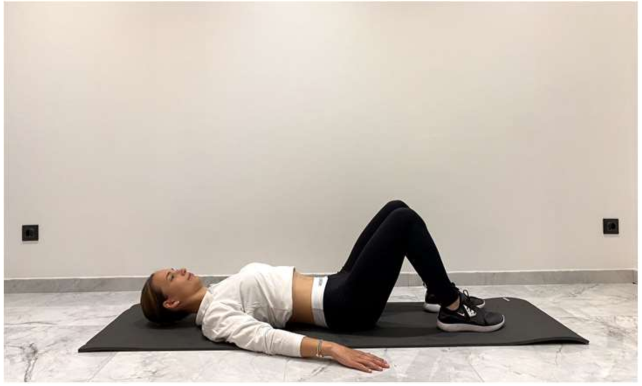

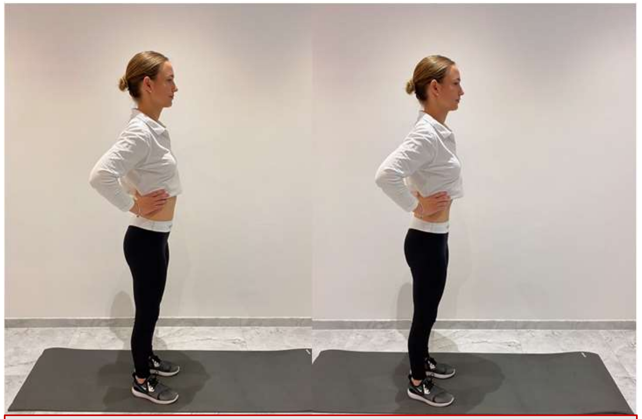

Variation

1
A

Supine position:
Draw belly button towards spine

- If necessary, check the activity using the "pressure biofeedback unit".
- In supine position, lateral position, sitting, standing: move one/both arms/ legs and keep the trunk stable.
- While walking.

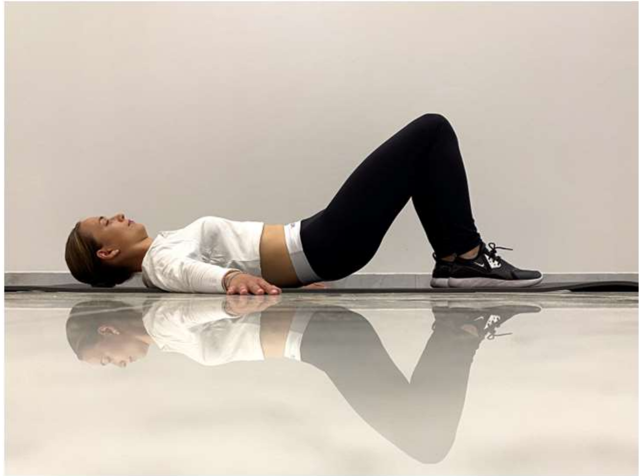

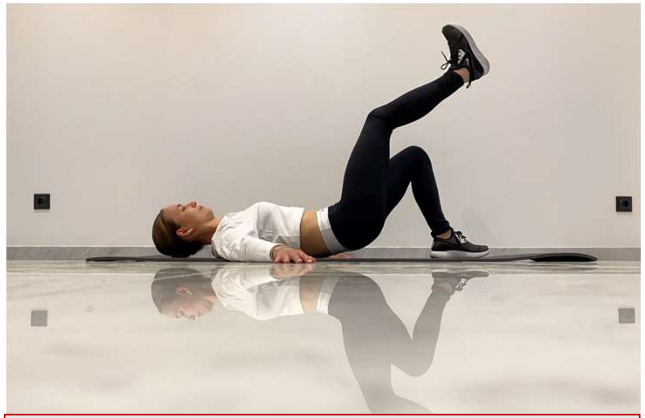

Variation 1

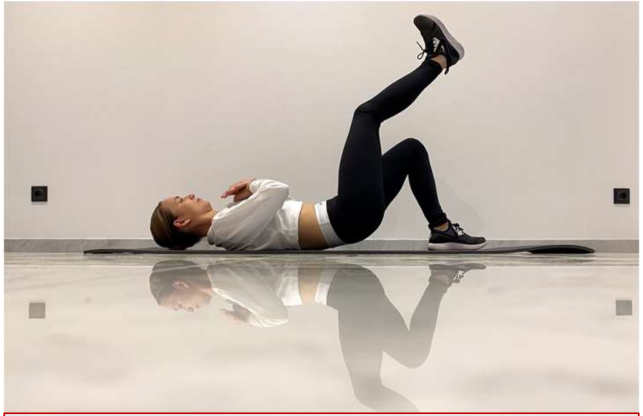

Variation 2

1 B

Supine position, knees bend: navel to spine & lift buttocks slightly

- A stable torso must be ensured during execution.
- Hold the position briefly at first, then increase the duration.
- Variation: left or right leg raised, without support of the arms, etc.

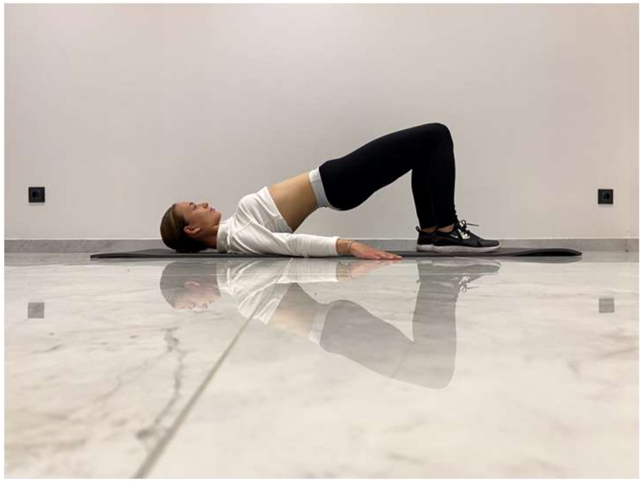

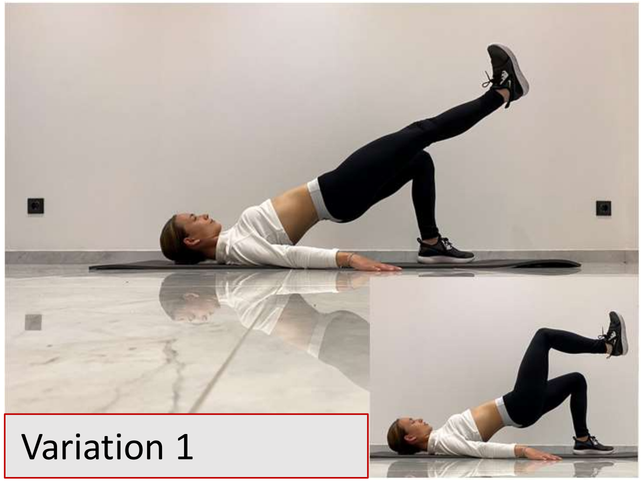

Variation 1

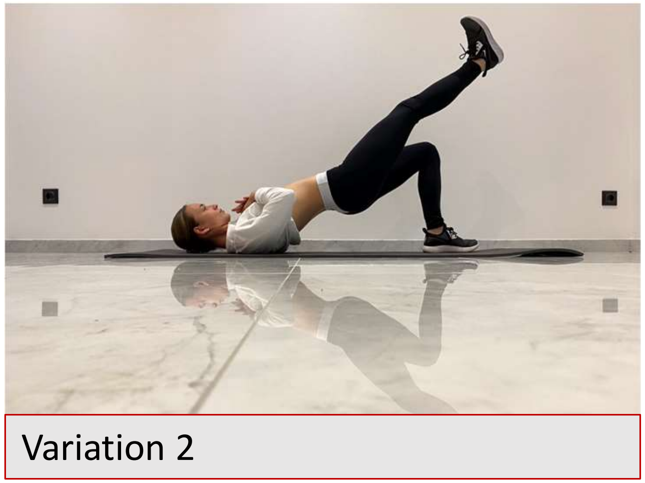

Variation 2

1 C	Supine position, knees bend: navel to spine & lift buttocks as far as possible (bridge position)

- Hold your position and make sure your torso is stable.
- Variation: lift your left or right leg, without support of the arms, etc.

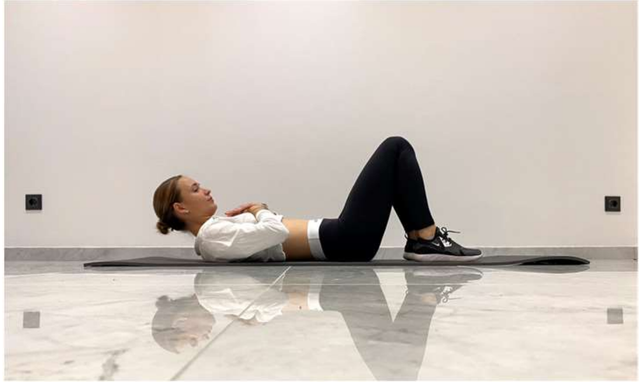

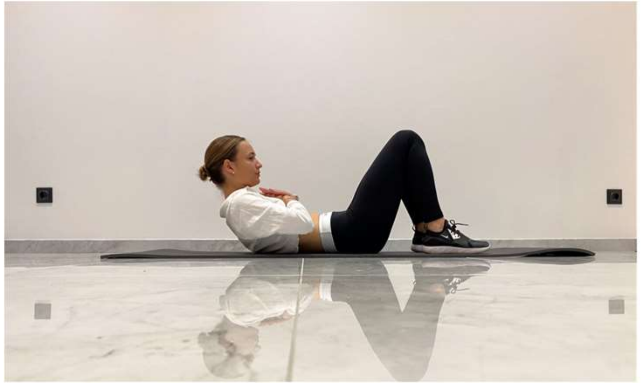

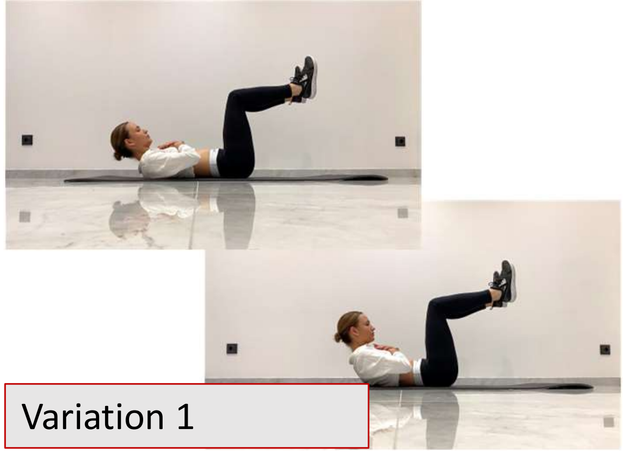

2
A

Supine position, knees bend: sit-ups

- Variation: both legs are raised in a 90° angle

Variations 2-5

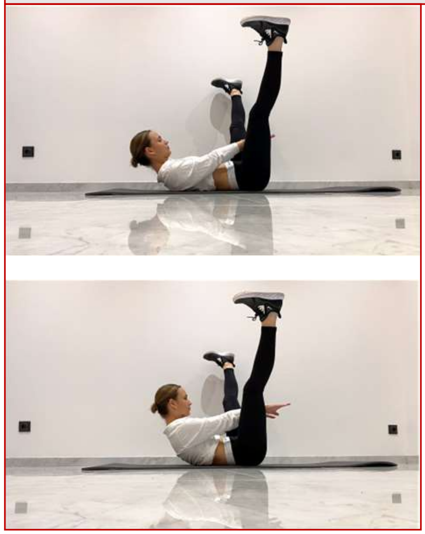

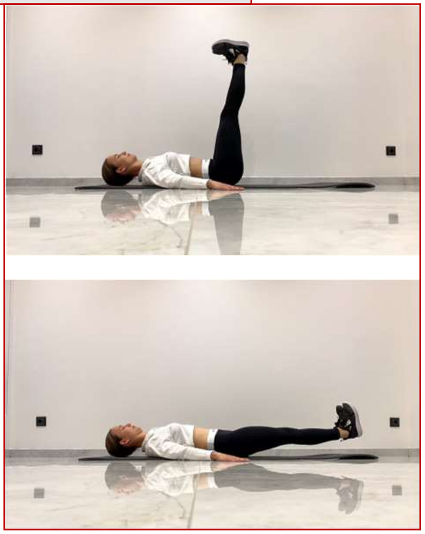

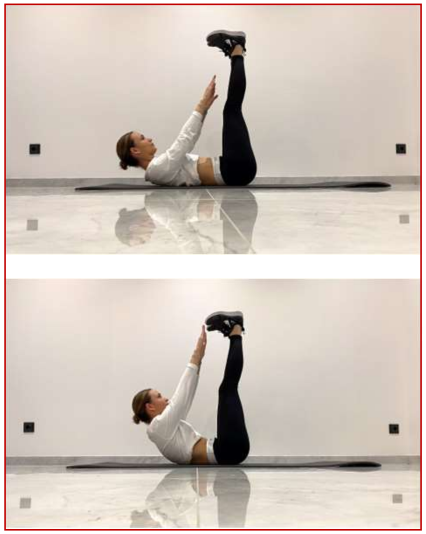

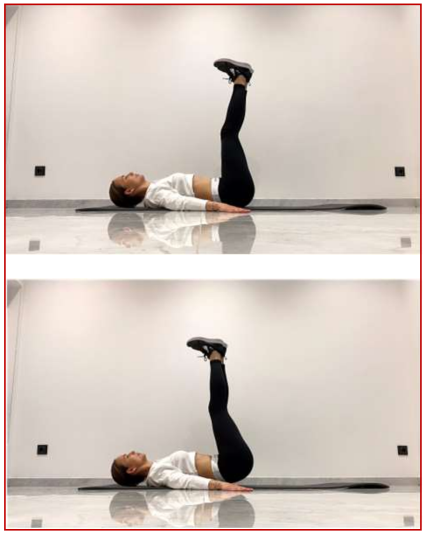

2
A

- Further variations:

legs stretched out wide towards the ceiling
legs stretched out towards the ceiling & slowly lower legs & lift them up again (leg raise)
legs stretched out towards the ceiling & try to touch the toes (bug)
legs stretched out towards the ceiling and lifting the pelvis

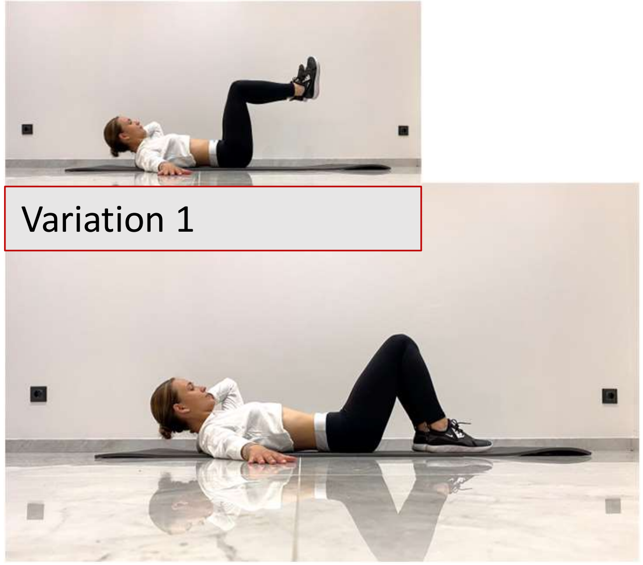

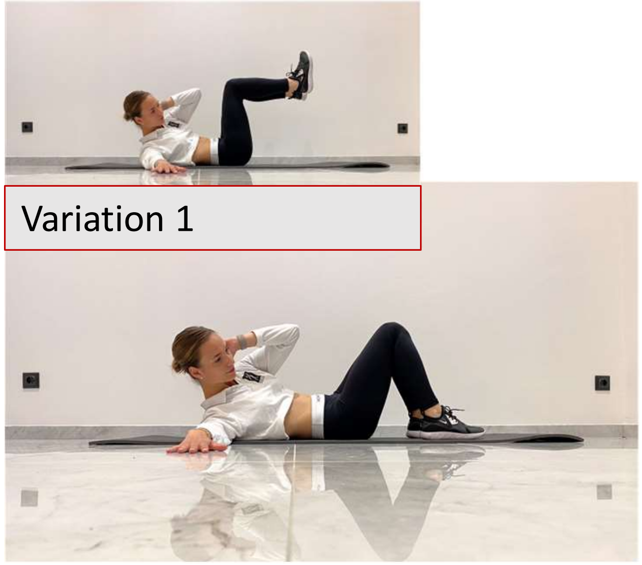

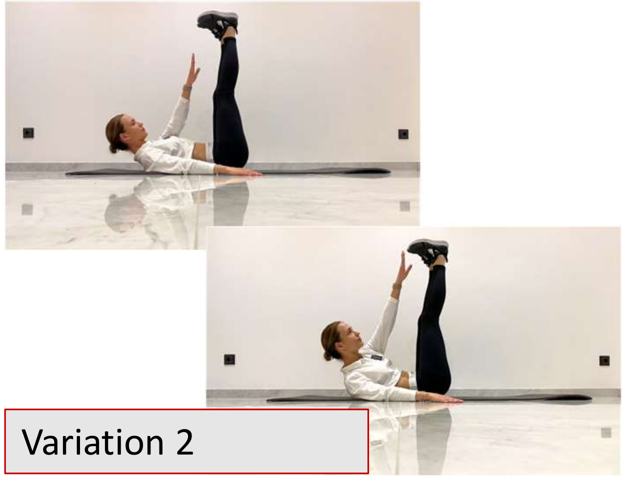

2 B Supine position: cross crunch

The right elbow touches the left knee, the left elbow touches the right knee.

- Variation: both legs are raised in a 90° angle, legs stretched out towards the ceiling (right hand touches left foot, left hand touches right foot)

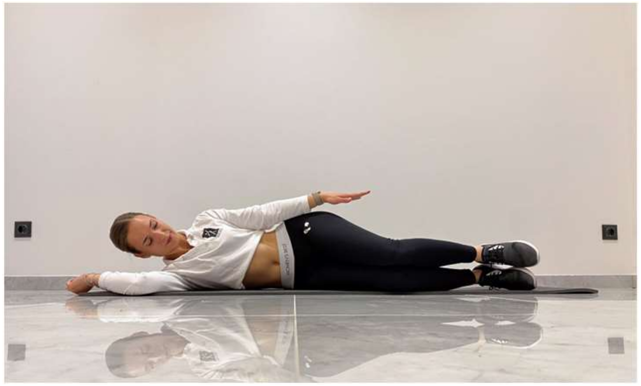

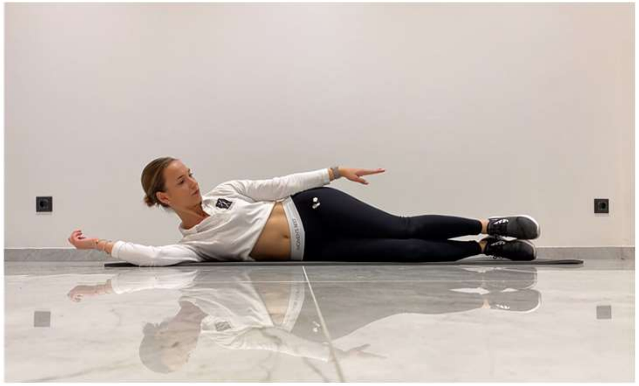

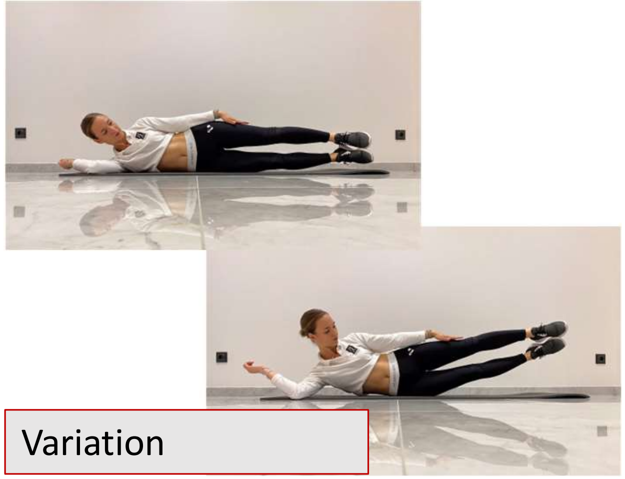

2
C

Supine position : side crunch

- Variation: lift legs at the same time

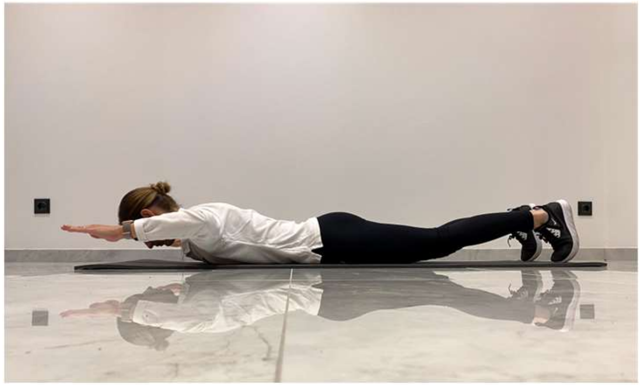

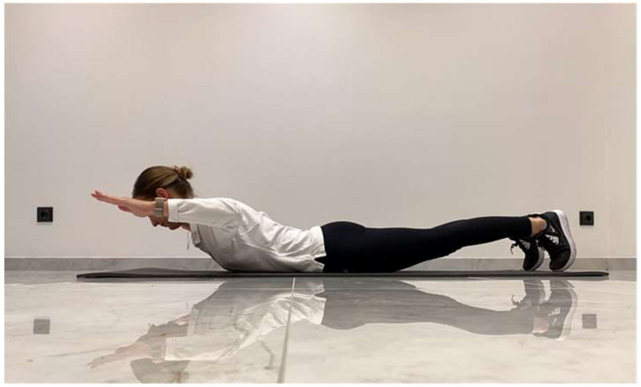

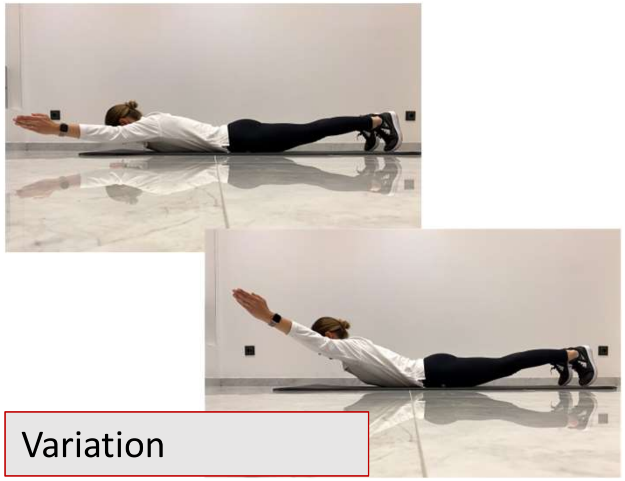

3 A

Prone position: lift upper body

- Variation: Hold position, different positions of the arms (T, W, I, V)

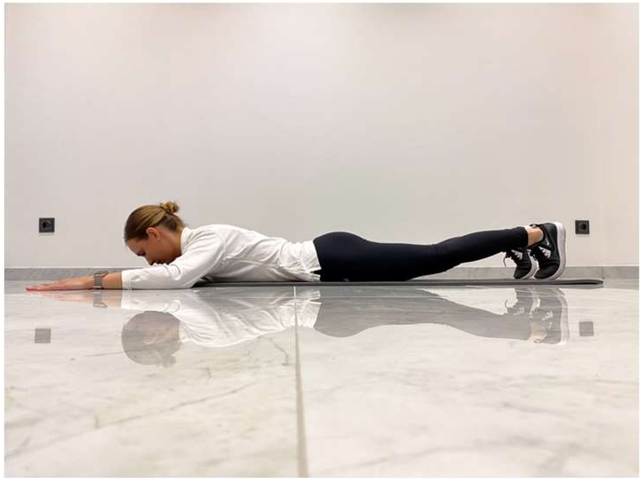

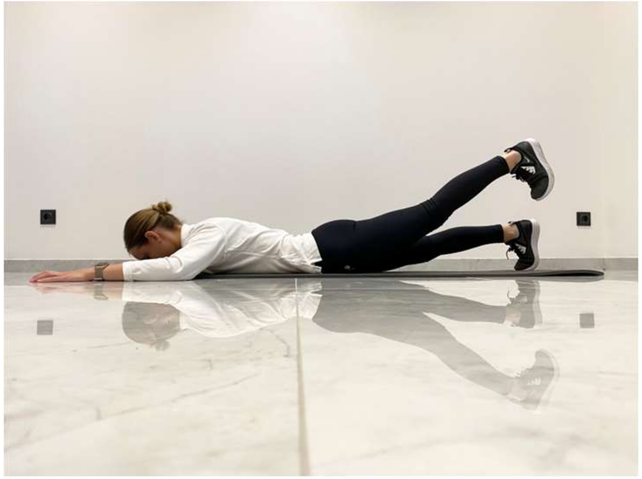

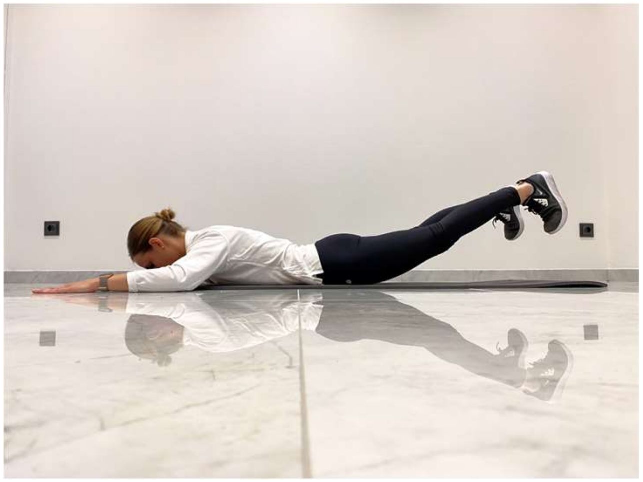

3 B

Prone position: lift one or both legs

- The leg should be raised as far as possible (preferably with the knee extended), while the pelvis should remain stable on the floor.

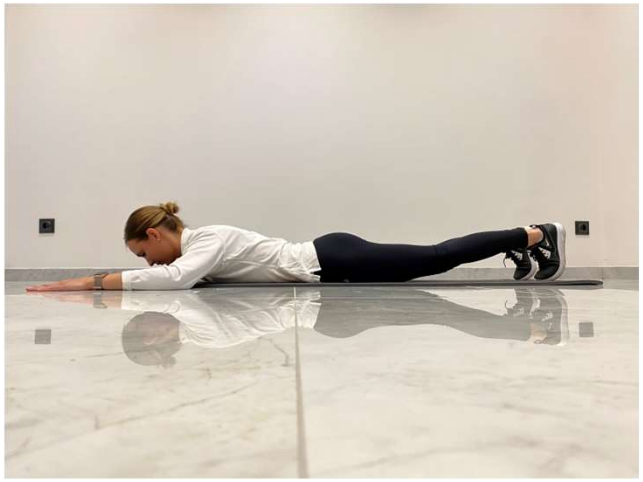

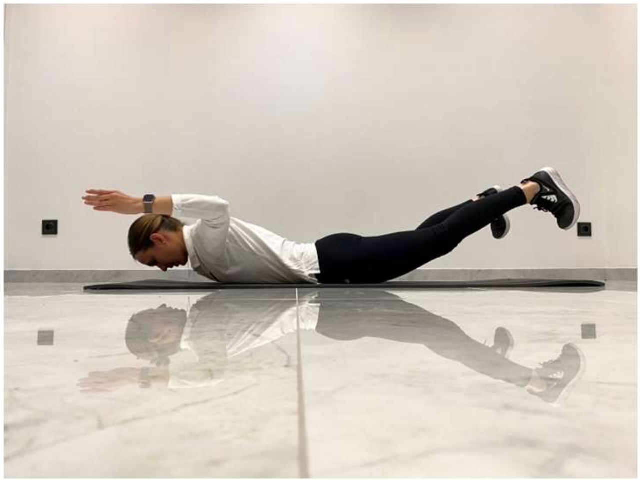

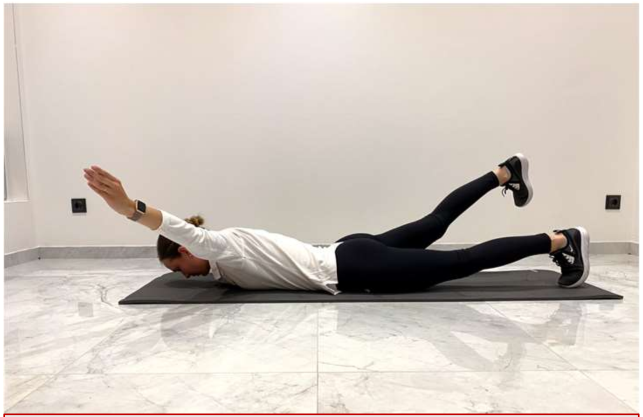

Variation

3
C

Prone position: lift arms and legs

- The pelvis always remains stable on the floor during this exercise.
- Variation: opposite (lift left arm and right leg), alternating (first arms, then legs), lift the entire upper body in addition to the arms and legs

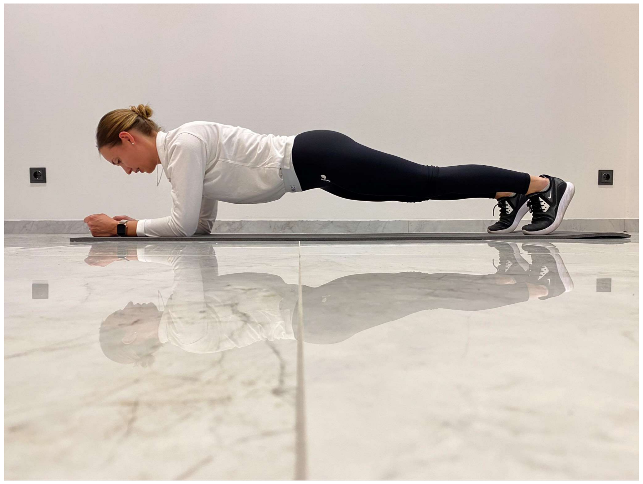

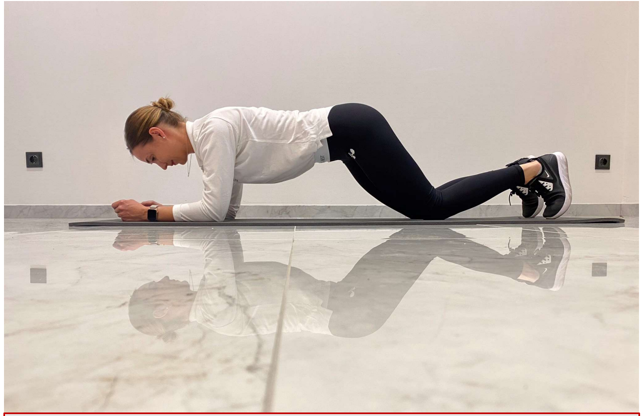

Variation 1

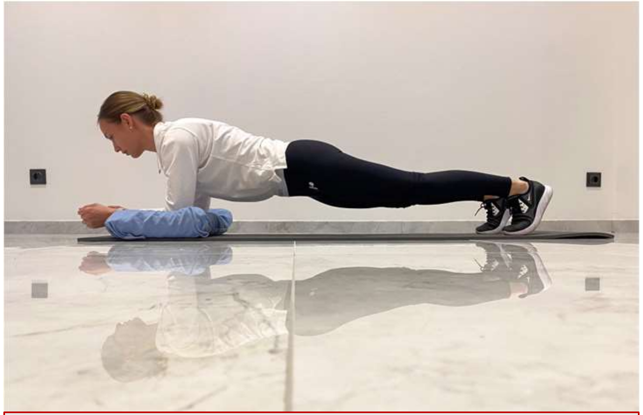

Variation 2

4 A

Forearm plank: hold for 15 seconds

- Variation: easier: knees can be placed on the floor; harder: balance pad under the forearms and/or feet

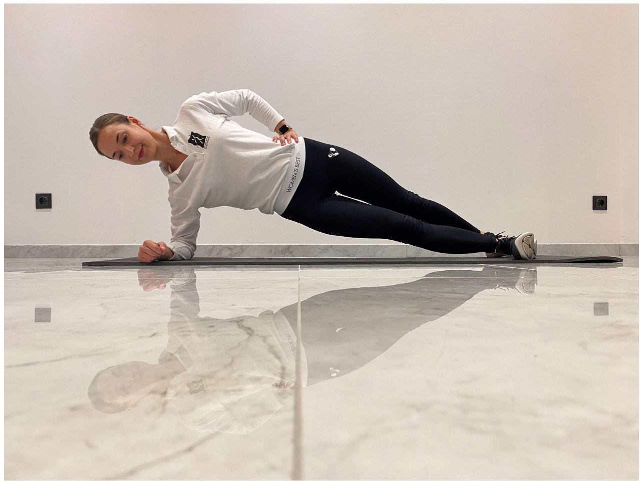

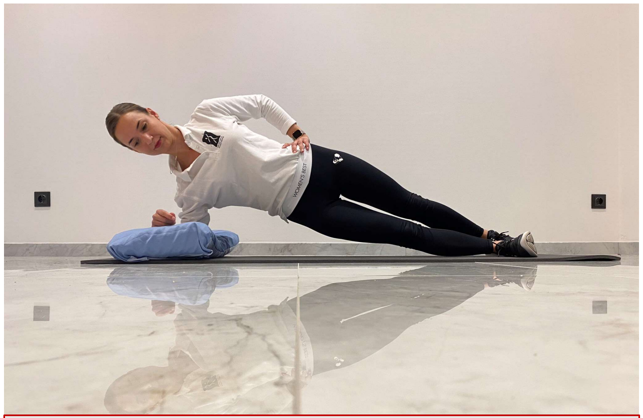

Variation 1

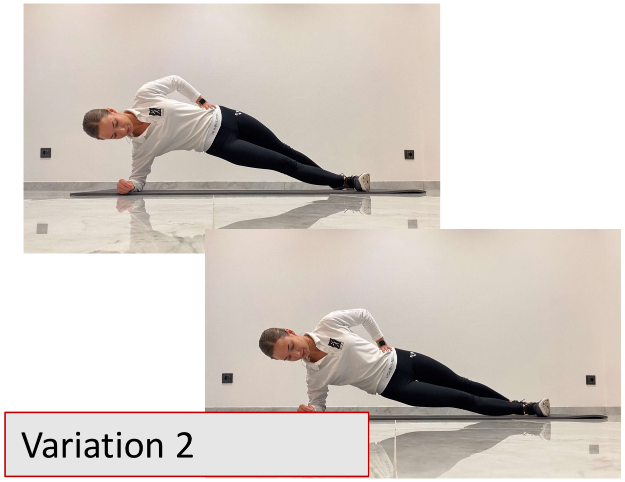

Variation 2

4
B

Forearm side plank: hold for 15 seconds

- Variation: slight lifting and lowering of the pelvis, balance pad under the forearms and/or feet

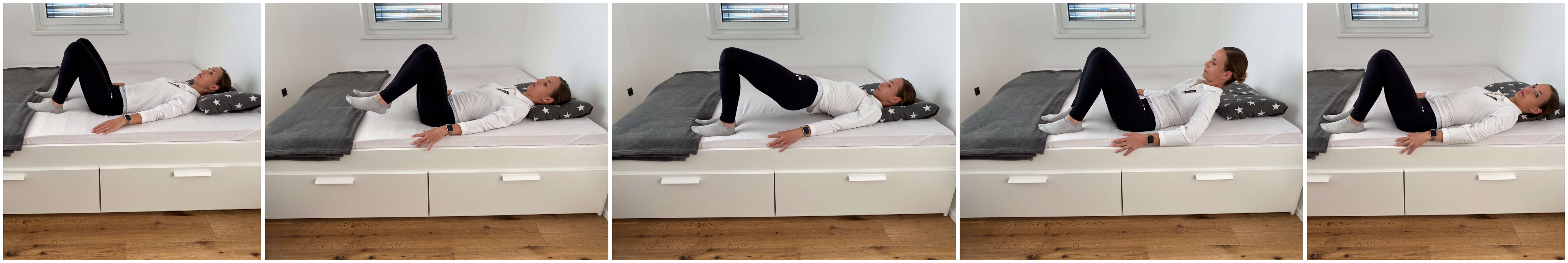

5
A

Lying in bed: move to the left/ right edge of the bed

- If necessary, support can be provided by aids or the therapist.

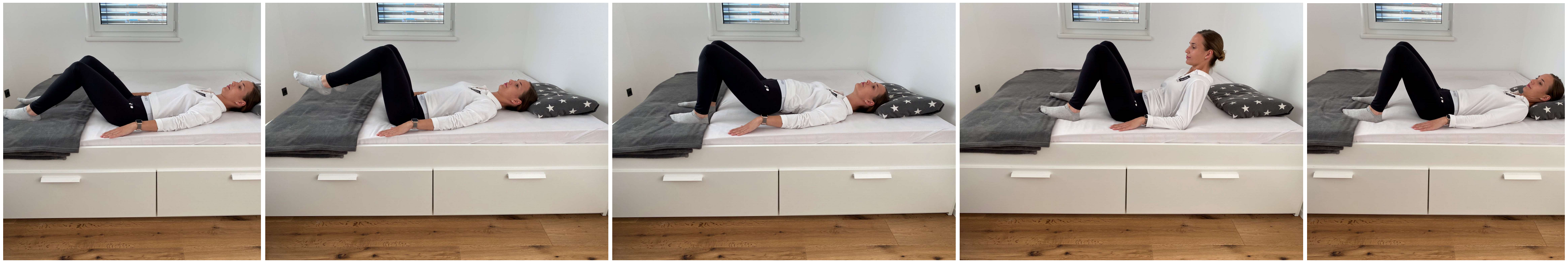

5
B

Lying in bed: move to the upper/lower edge of the bed

- If necessary, support can be provided by aids or the therapist.

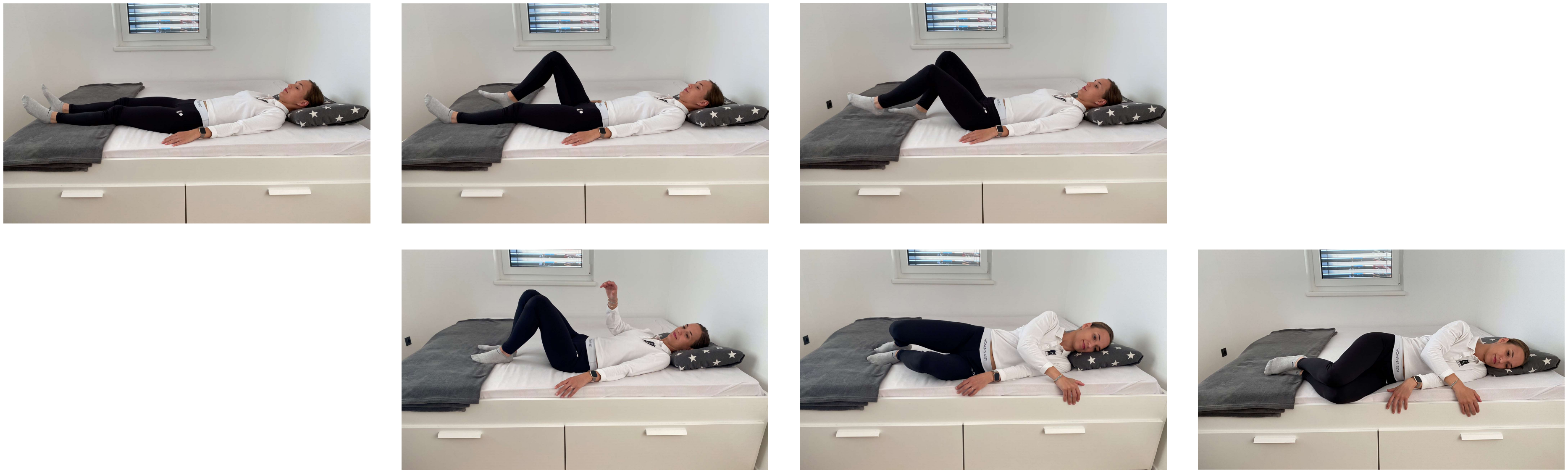

5 C

Lying in bed: turn to the left and right side

- If necessary, support can be provided by aids or the therapist.

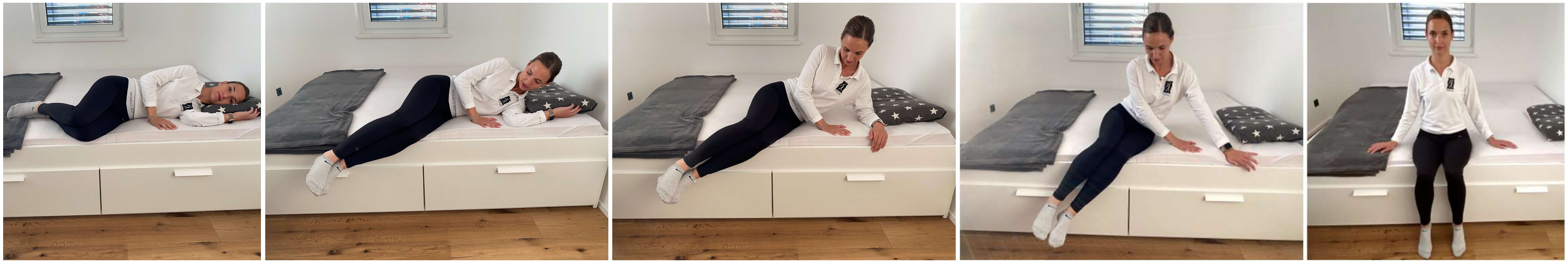

6 A

Sit up from side-lying position & lie down again

- If necessary, support can be provided by aids (e.g., bed gallow) or the therapist.
- Variation: sit up from supine position, sit up from prone position, sit up in bed then turn and put feet on the ground

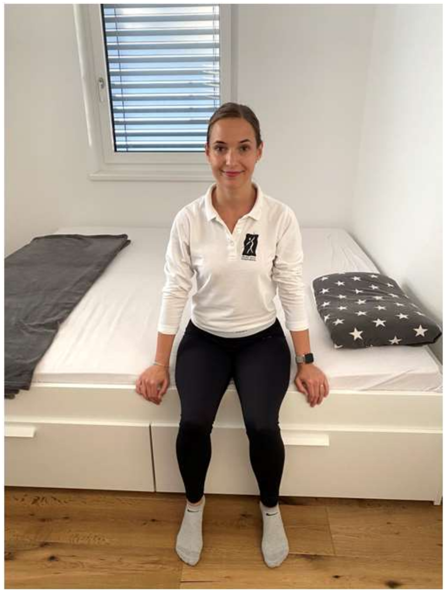

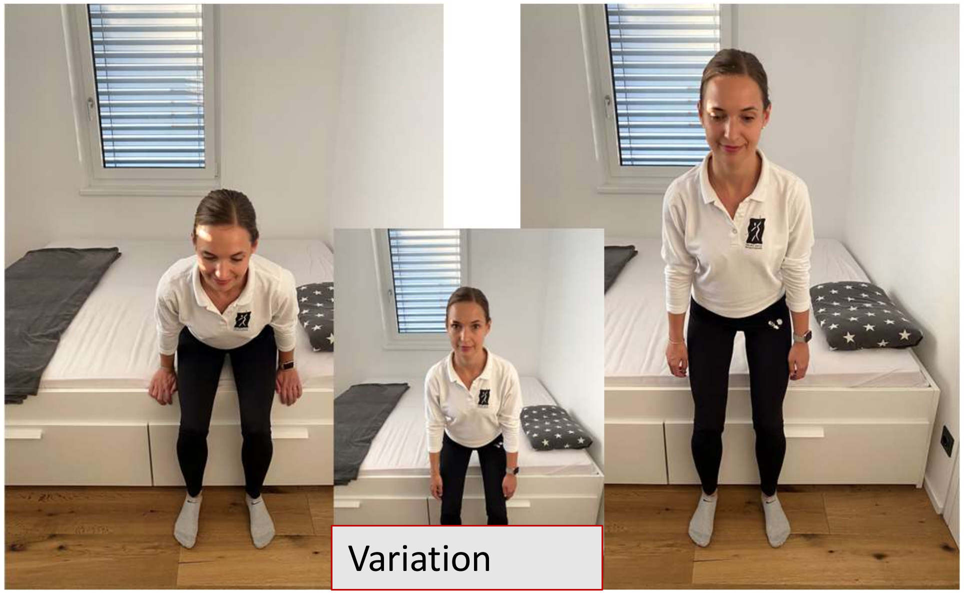

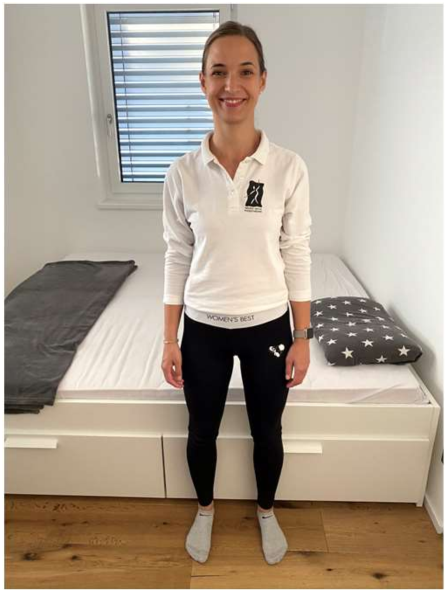

6 B

Sit-to-stand, stand-to-sit

- If necessary, support can be provided by aids or the therapist.
- Variation: various starting positions (low/raised seat, different stance widths), with or without support of the arms

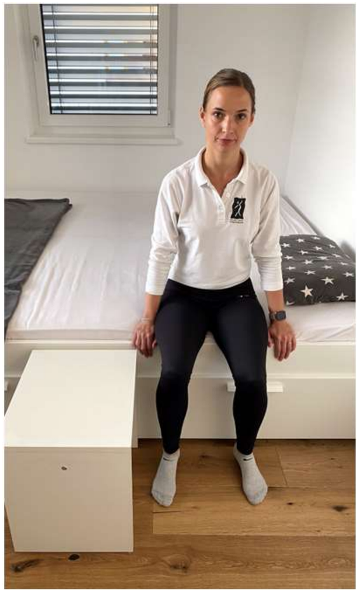 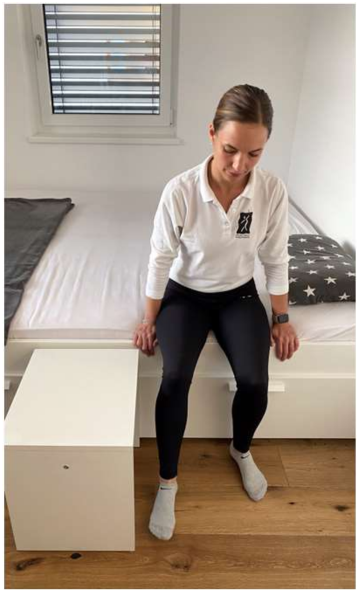 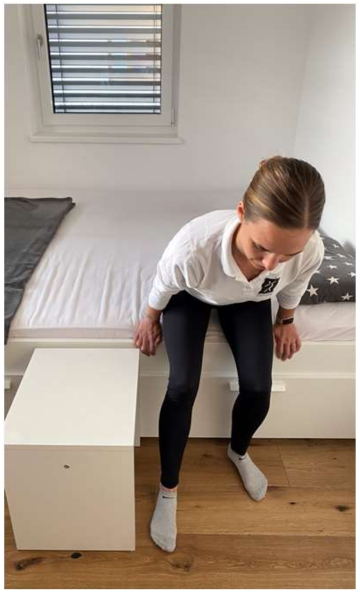 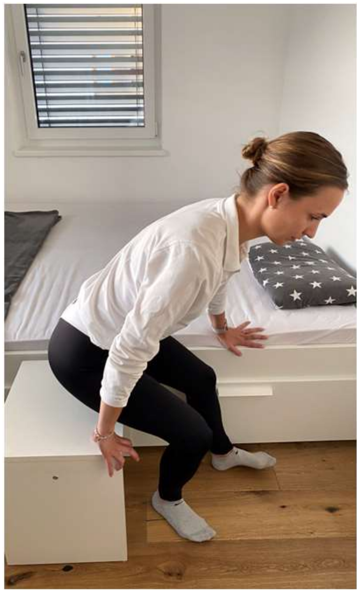 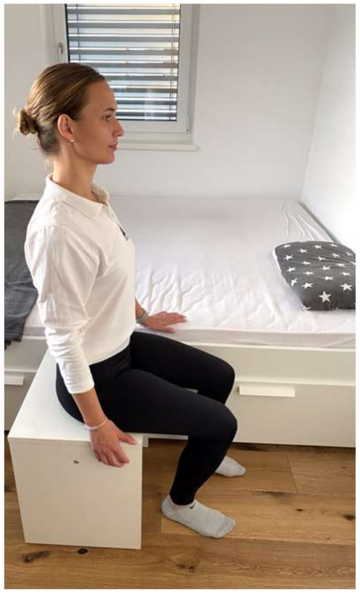

6 C

Transfer bed – chair – bed

- If necessary, support can be provided by aids or the therapist.
- Variations: Wheelchair - bed; wheelchair - WC; etc.

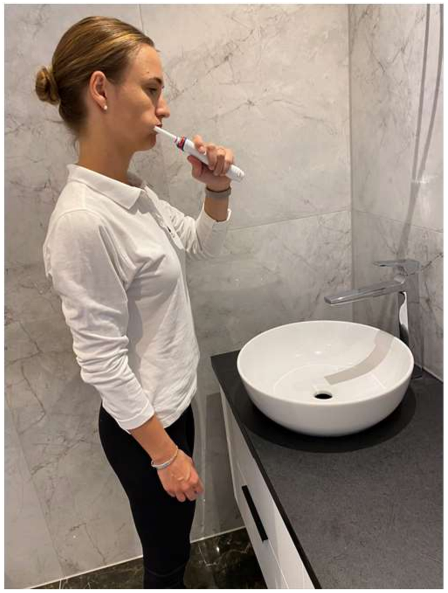

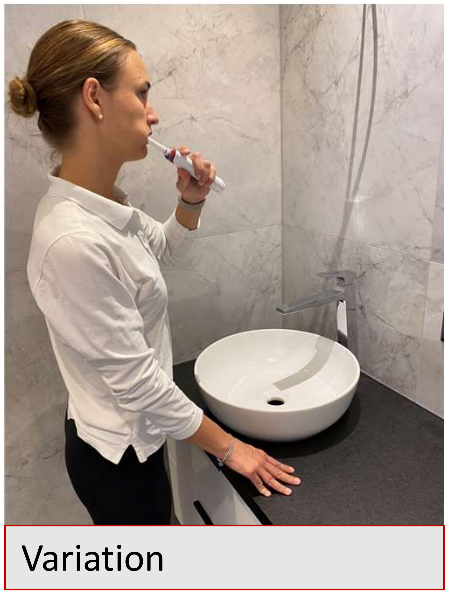

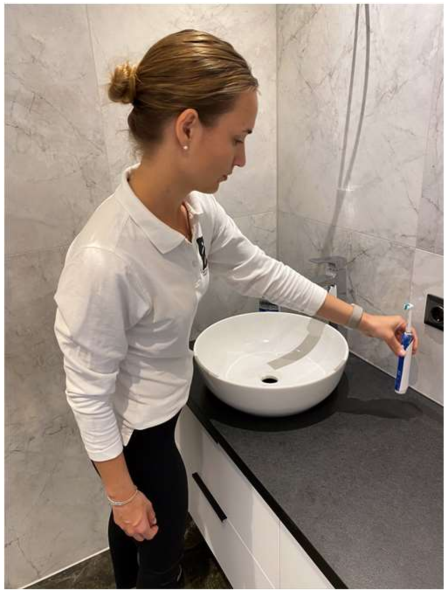

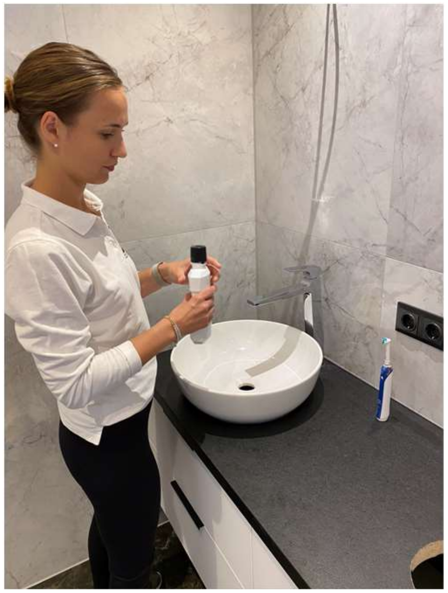

7 A

Sitting or standing: brushing your teeth

- Variation: washing face or other morning hygiene routine activities, with or without aids (e.g., support grab bar), arms can be used for support

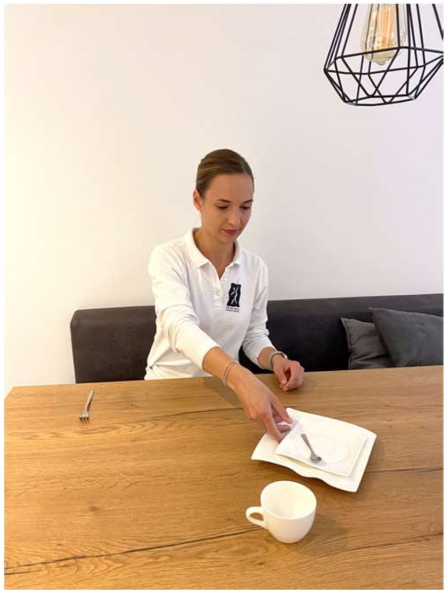

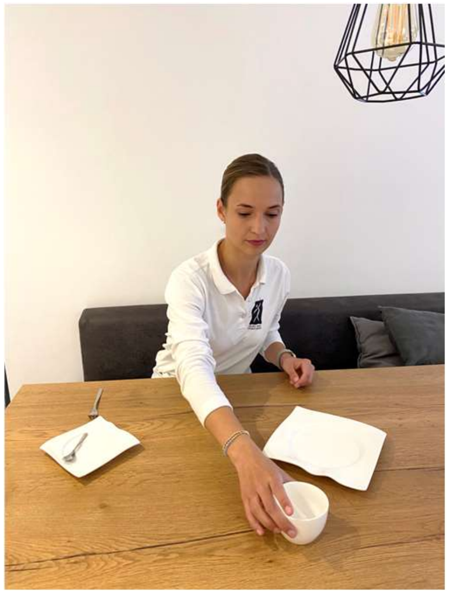

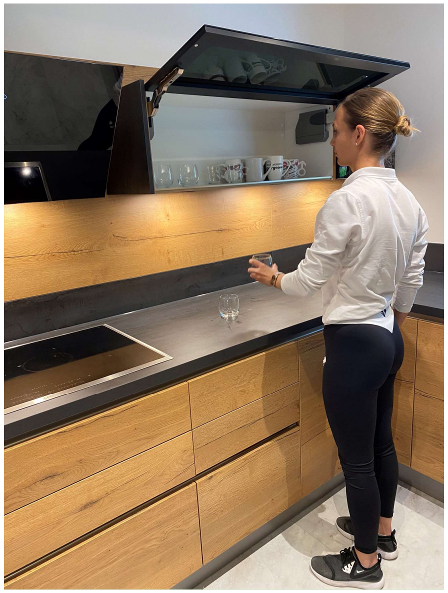

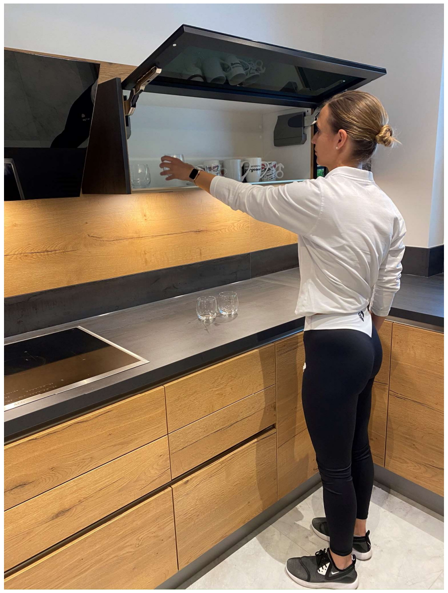

7 B

Sitting or standing: move objects on the table (e.g., cup)

- Variation: placing objects on the shelf or other activities that are carried out in the kitchen, e.g., making coffee/tea, buttering a loaf of bread, etc.

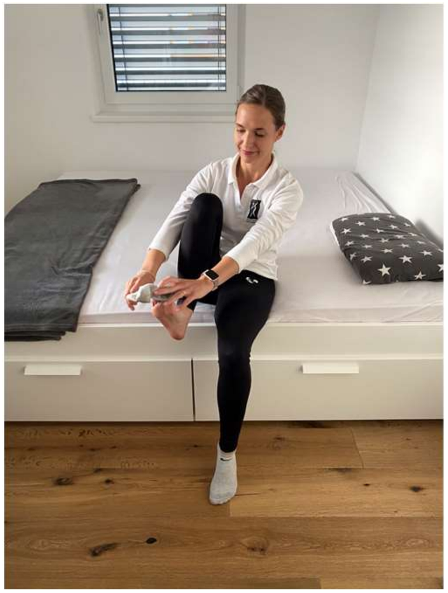
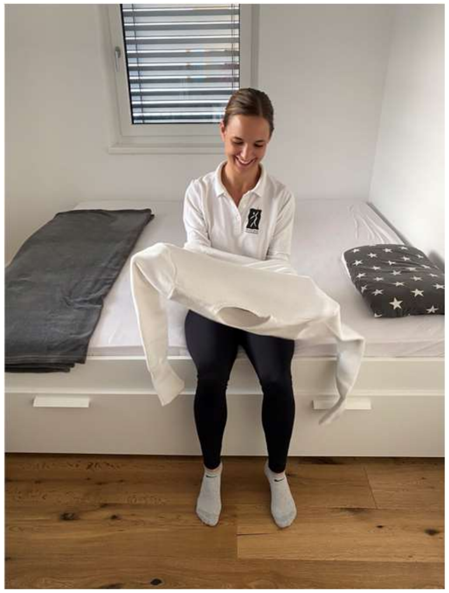
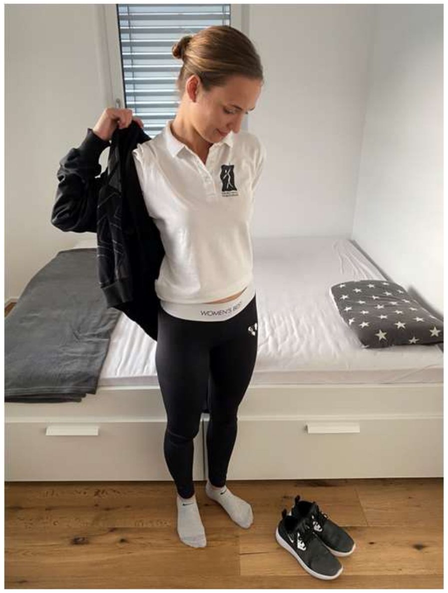
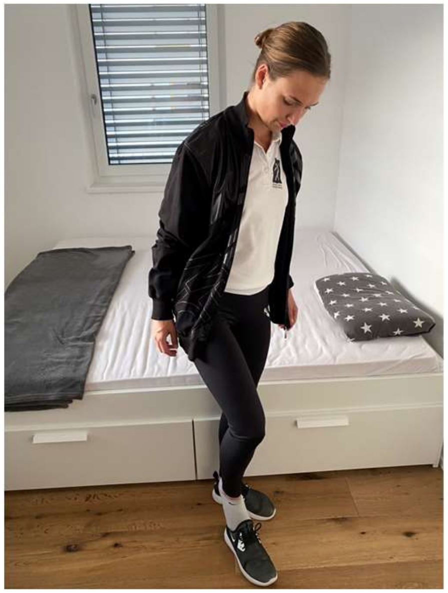

7 C

Sitting or standing: getting dressed

- Possibilities: top, pants, shoes, jackets, etc.

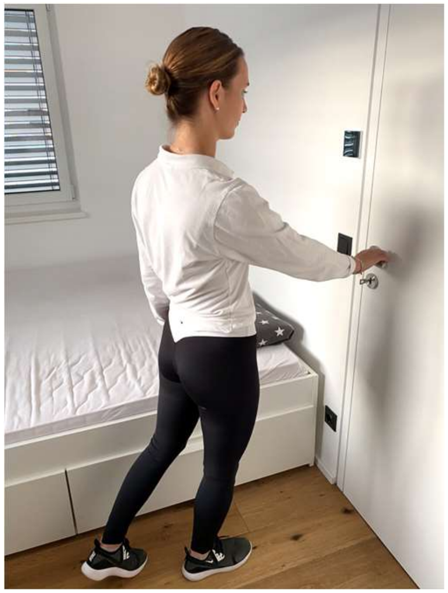 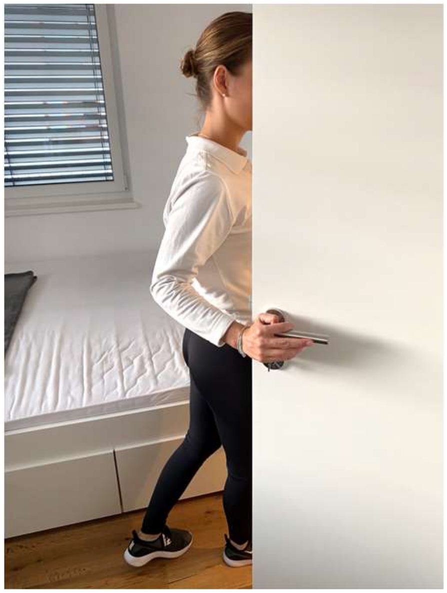 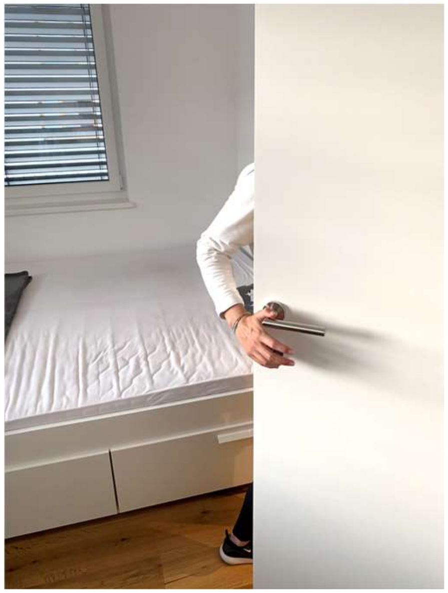

8 A

Open and close doors

- If necessary, aids can be used, assistance can be given. Compensation mechanisms are allowed.

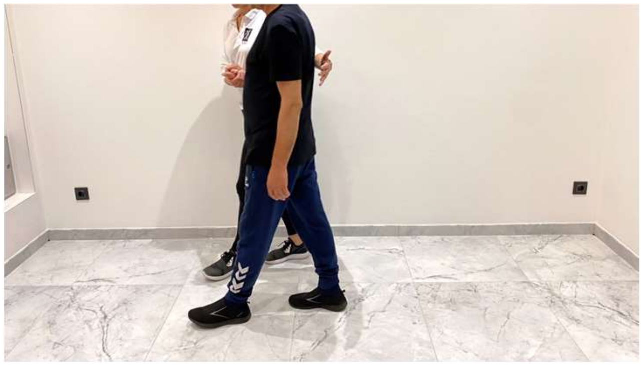

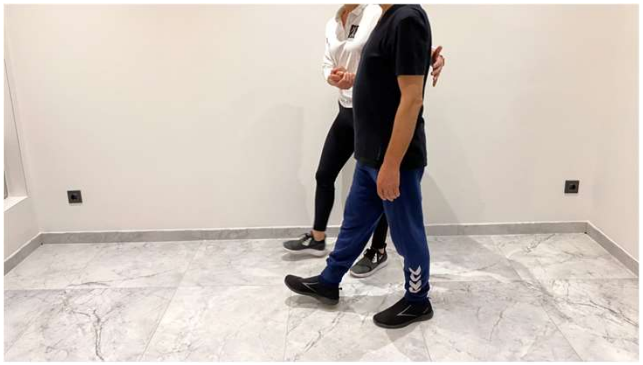

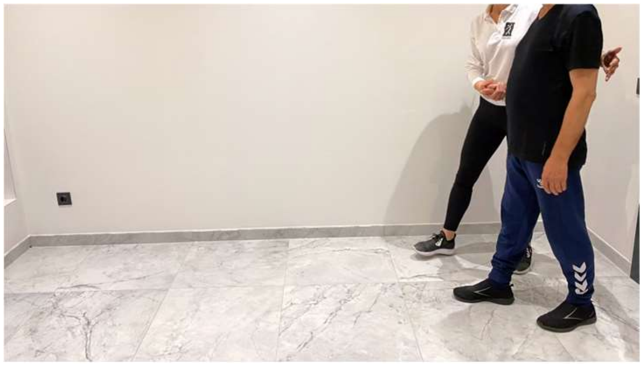

8
B

Walking from A to B with/without aids

- If necessary, assistance can be given. Compensation mechanisms are allowed.

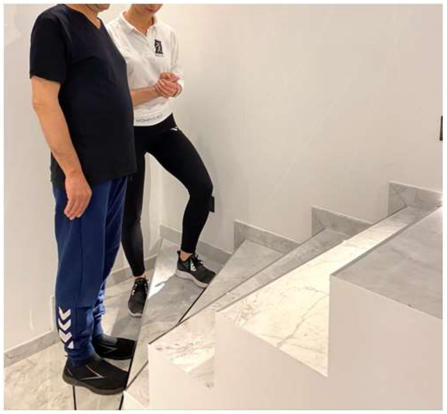
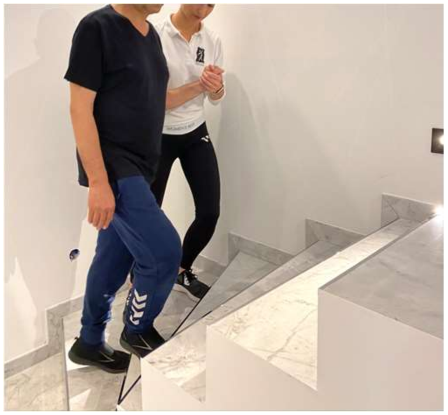
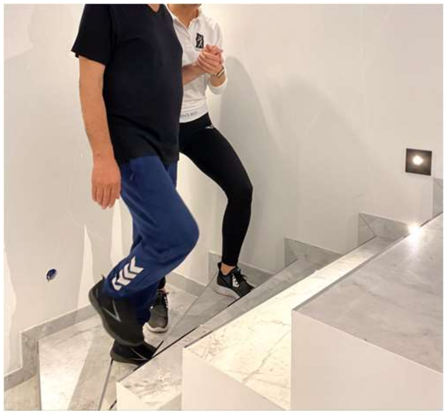
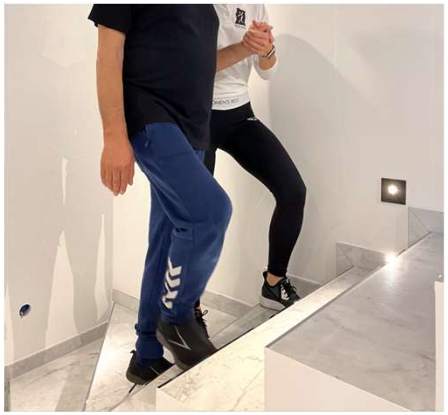

8
C

Walking the stairs with/without railings or aids

- Use of railing and aid (e.g., walking stick), if necessary.

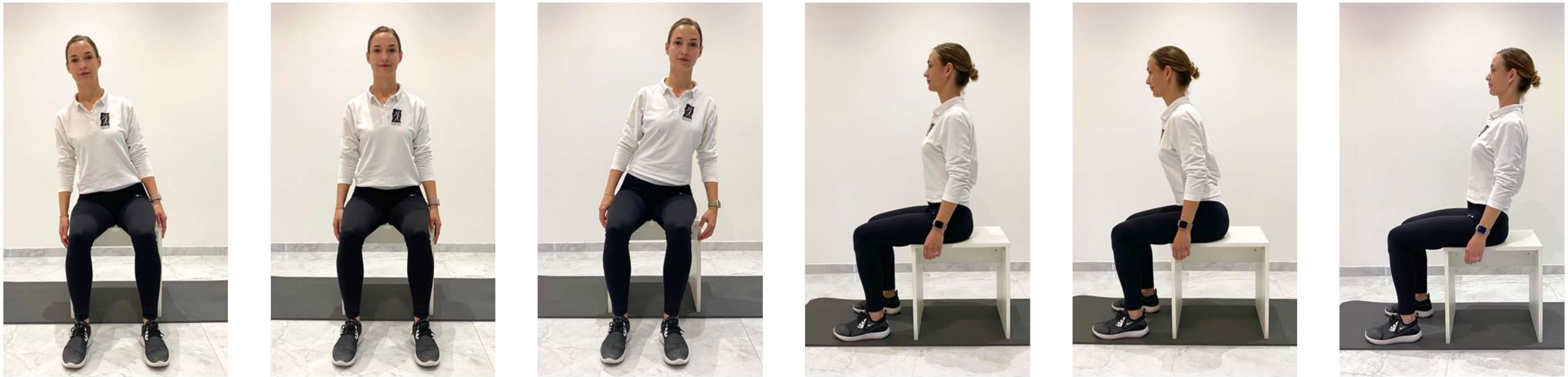

9 A

Weight shifting in different directions while seated

- To the left/right, front/back, in circles.
- If necessary, support can be provided by the therapist. Arms can be used for support.
- Variation: sitting on a balance pad or stability ball

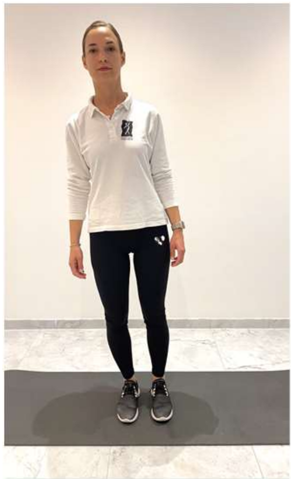

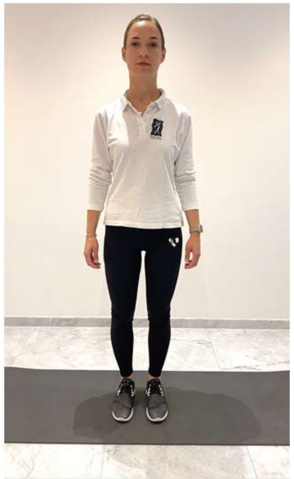

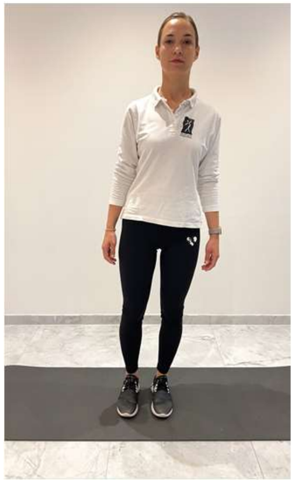

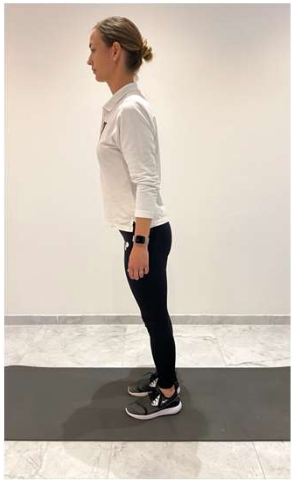

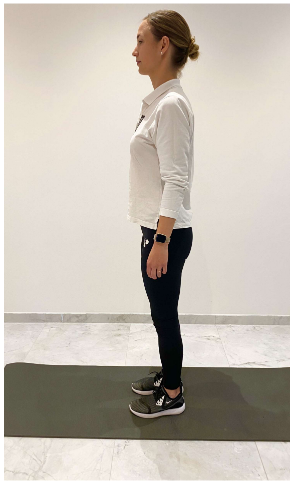

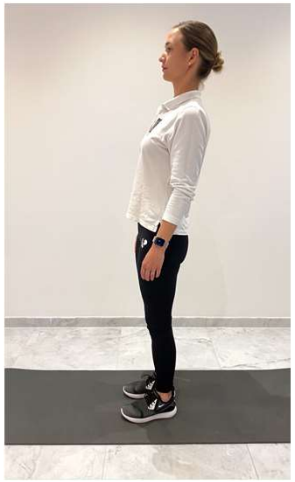

9 B

Shifting weight in different directions while standing

- To the left/right, front/back, in circles.
- If necessary, support can be provided by the therapist. Arms can be used for support (e.g., holding on to a table, placed in front).
- Variation: standing on a balance pad

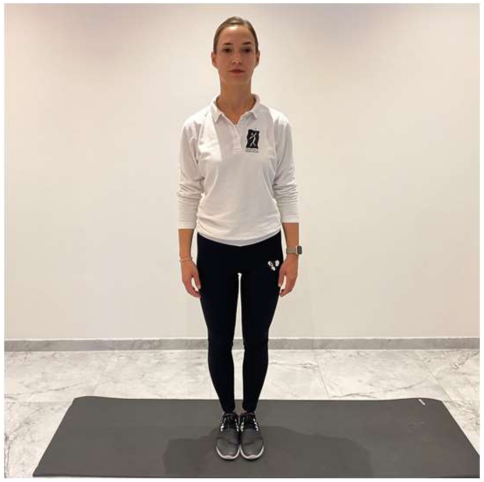

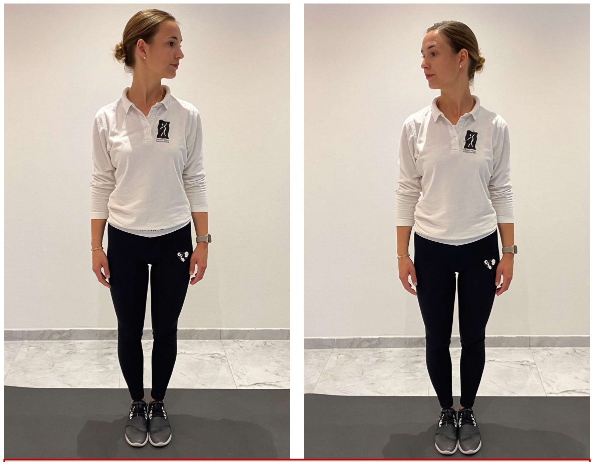

Variation 1

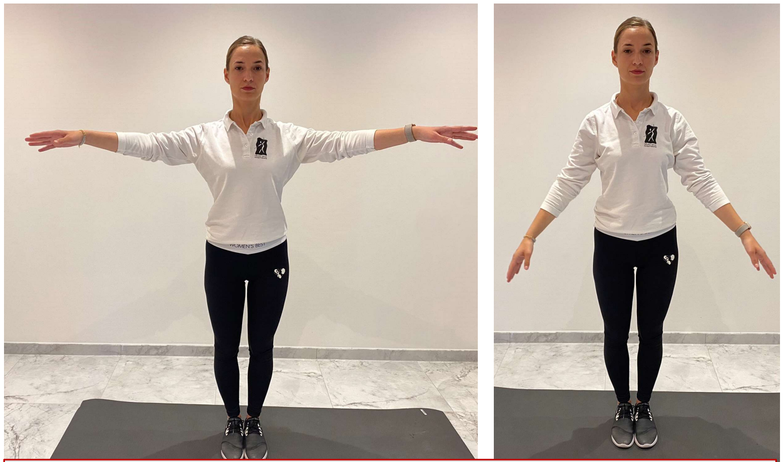

Variation 2

10 A

Stand with your feet together and keep your balance: hold the position for 15 seconds

- Variation: hold the position for longer (e.g., 60 seconds), add arm or head movements, catch a ball or similar

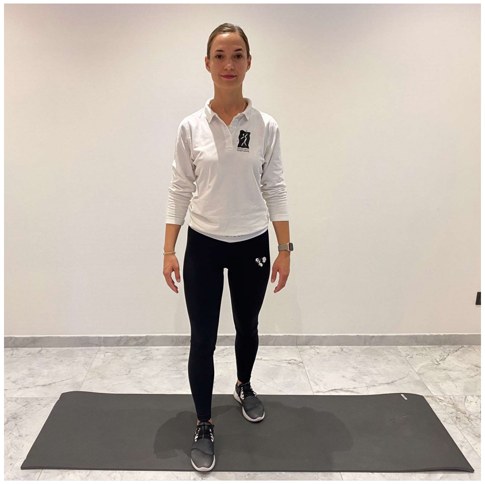

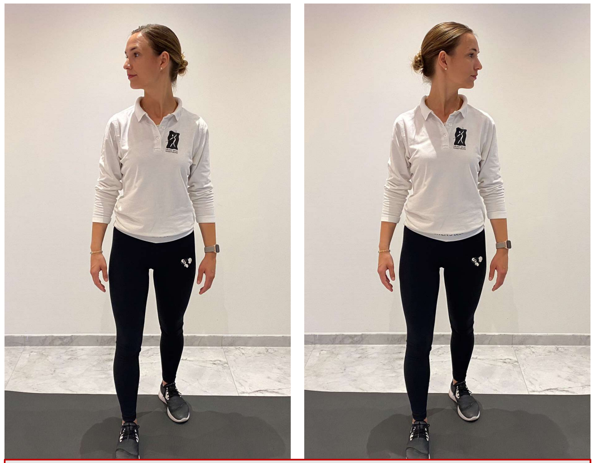

Variation 1

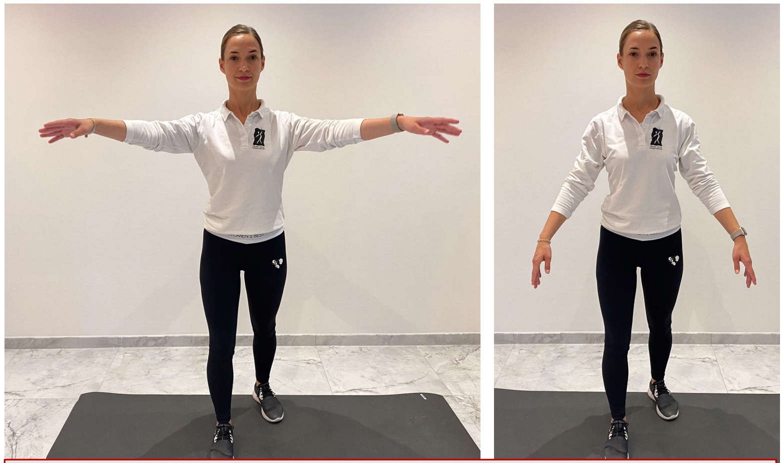

Variation 2

10 B

Standing with one foot in front of the other and keep your balance: hold position for 15 seconds

- Variation: hold the position for longer (e.g., 60 seconds), add arm or head movements, catch a ball or similar, vary step length/width (e.g., tandem stance)

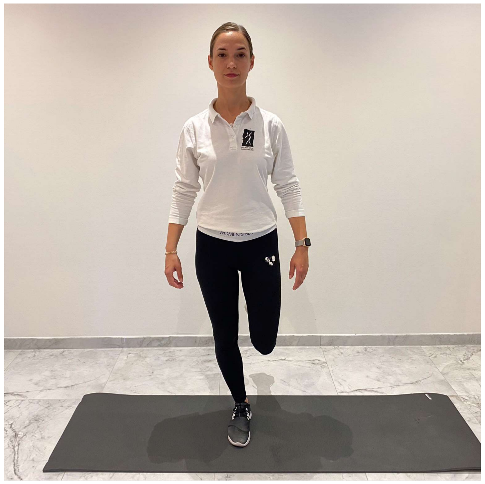

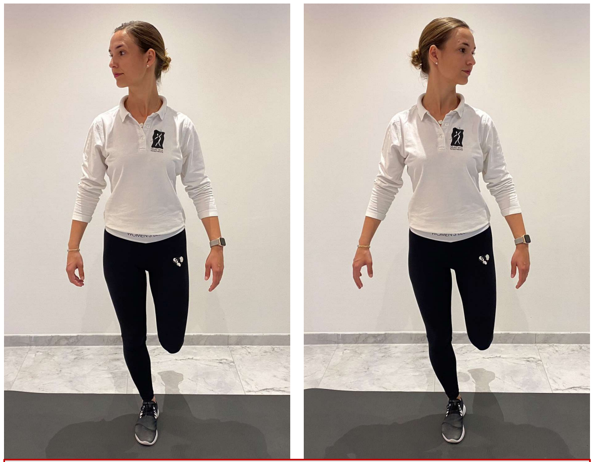

Variation 1

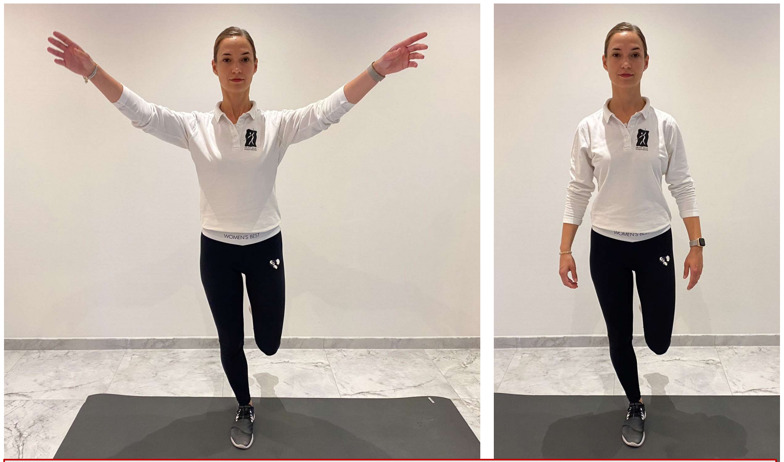

Variation 2

10 C

Stand on one leg and keep your balance: hold the position for 15 seconds

- Variation: hold the position for longer (e.g., 60 seconds), add arm or head movements, catch a ball or similar

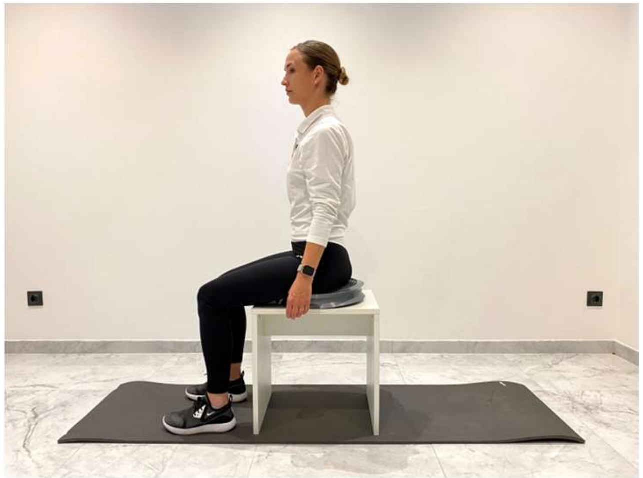

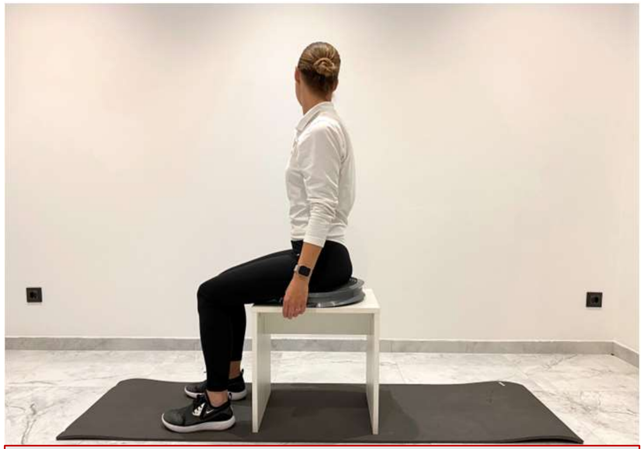

Variation 1

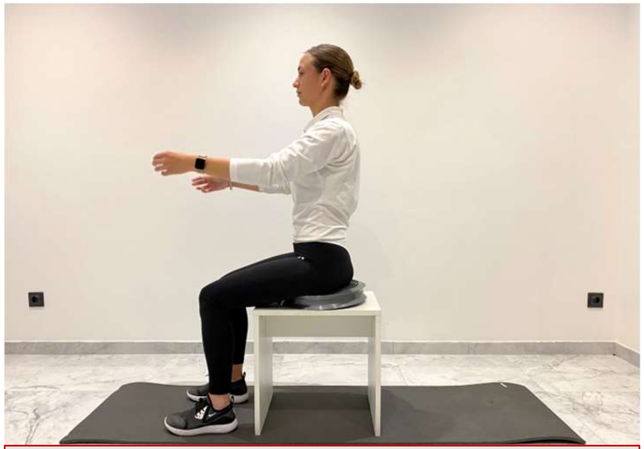

Variation 2

11 A

Sitting on a balance pad:
hold the position for 15 seconds

- Variation: hold the position for longer (e.g., 60 seconds), add arm or head movements, catch a ball or similar

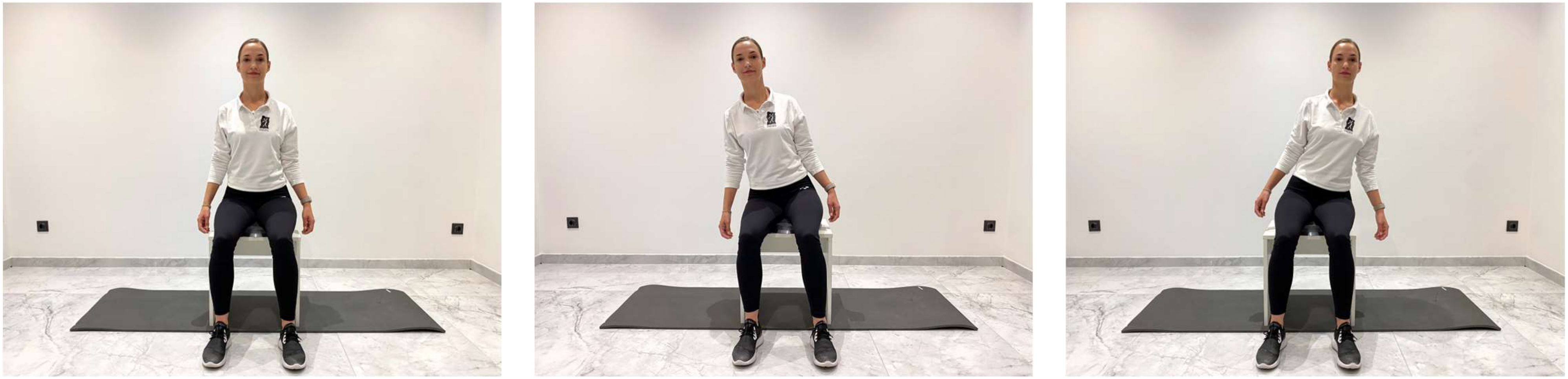

11 B

Sitting on a balance pad: lean to the side and try to hold the position

- Variation: add arm or head movements, use a stability ball for sitting

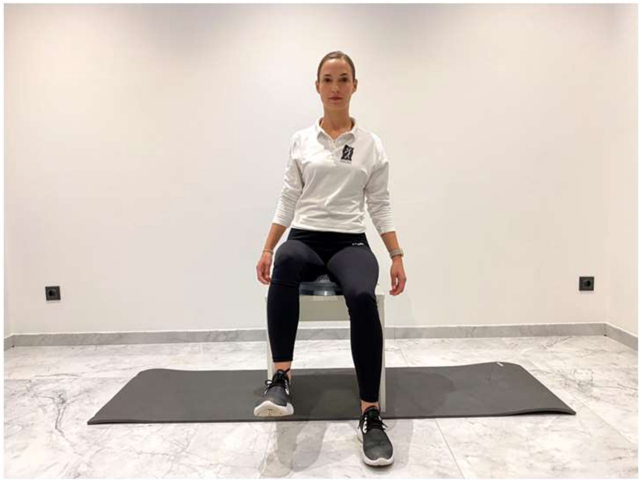

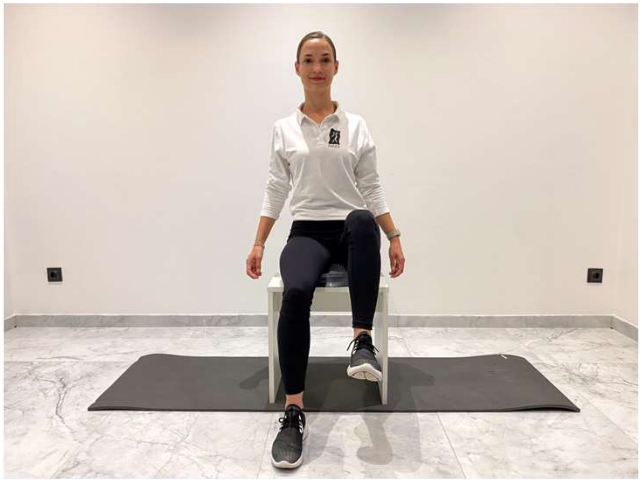

11
C

Sitting on a balance pad: lift your foot off the ground

- Variation: sit on a stability ball instead

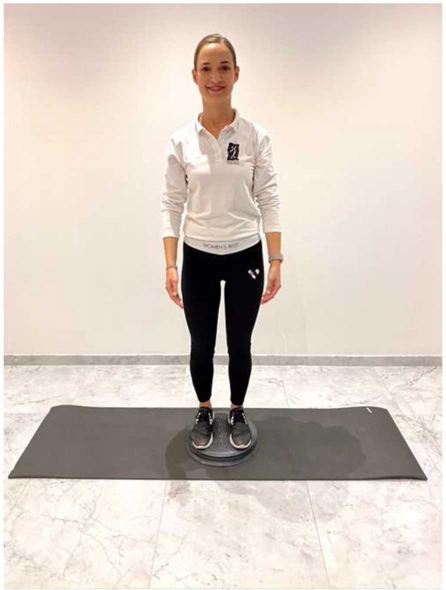

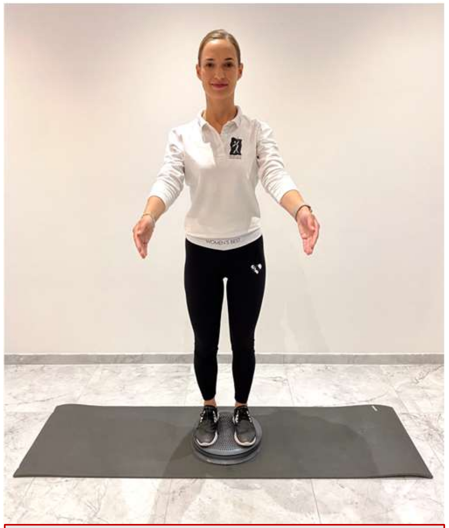

Variation 1

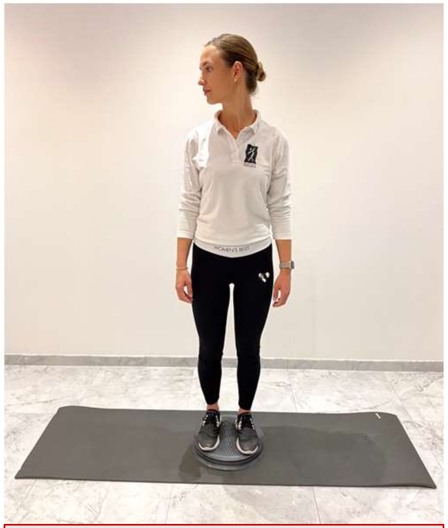

Variation 2

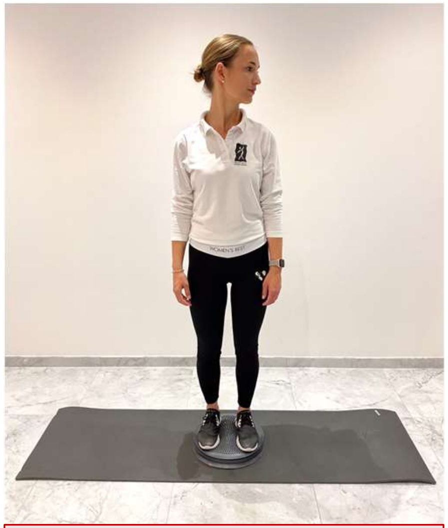

Variation 2

12 A

Standing on a balance pad: hold the position for 15 seconds

- Variation: hold the position for longer (e.g., 60 seconds), add arm or head movements

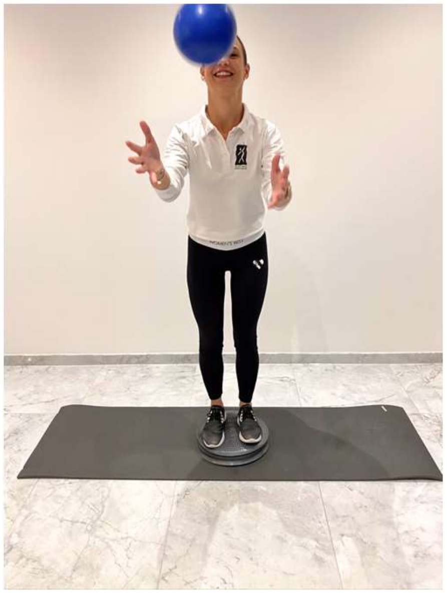

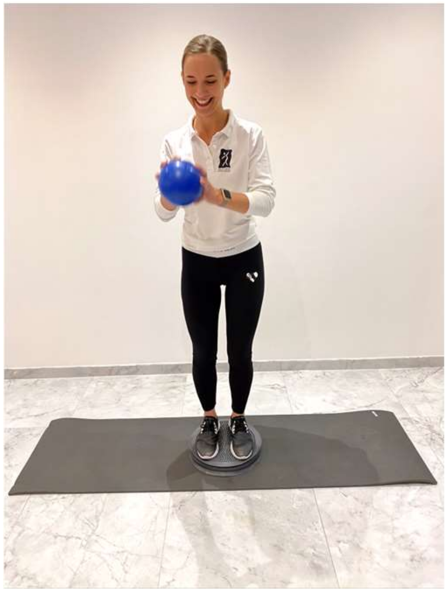

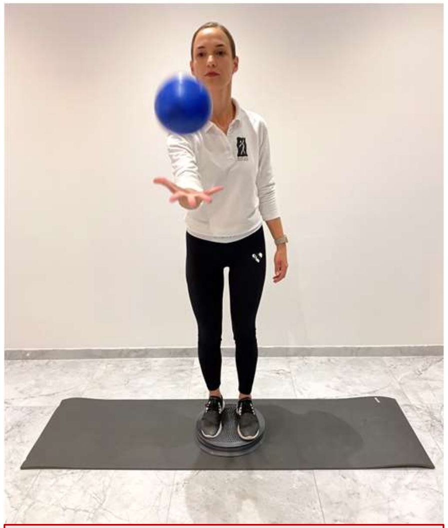

Variation 1

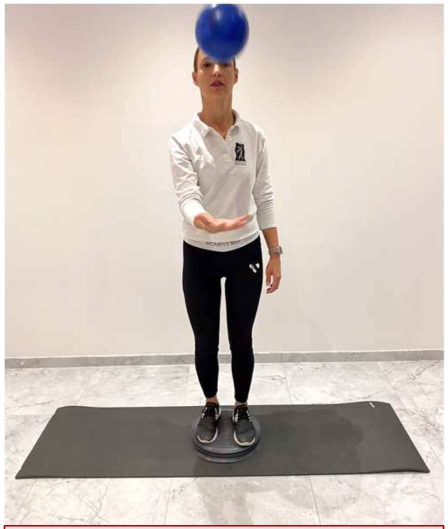

Variation 1

12 B

Standing on a balance pad: catching a ball

- Variation: vary objects (e.g., balloon, medicine ball, tennis ball, etc.) to make the exercise easier or more difficult, catch with one hand

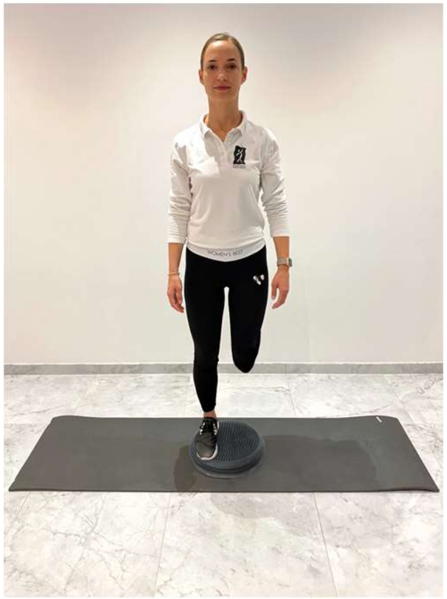

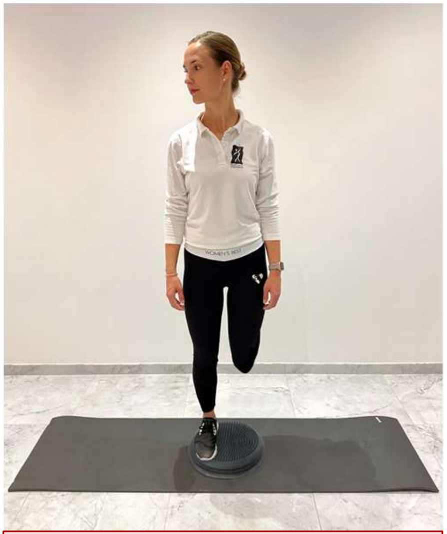

Variation 1

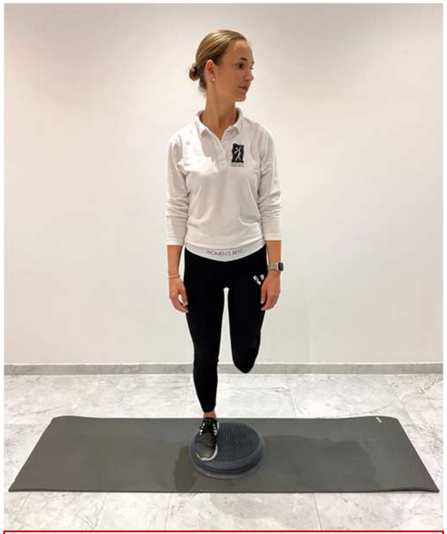

Variation 1

12 C

Single leg stance on a balance pad: hold the position for 15 seconds

- Variation: hold the position for longer (e.g., 60 seconds), add arm/leg or head movements

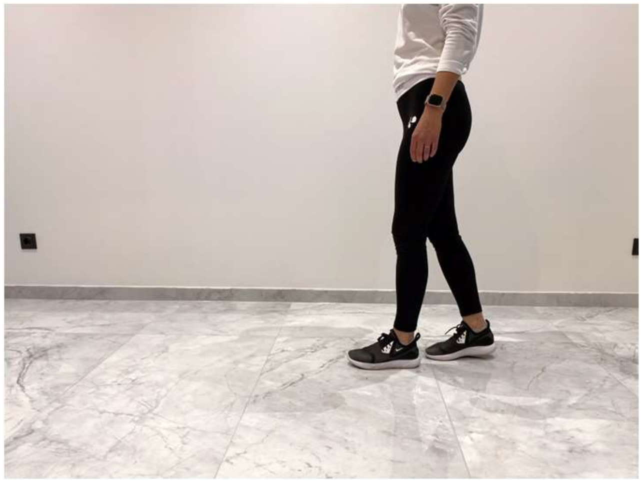

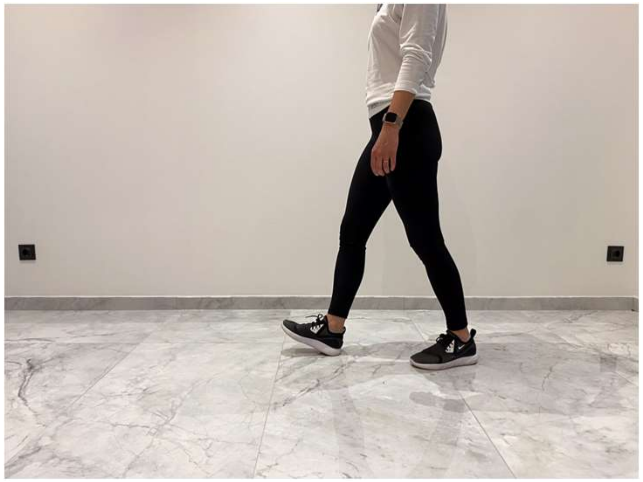

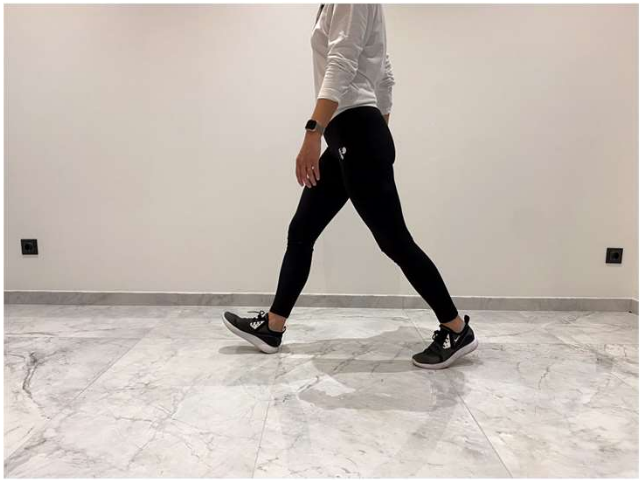

13 A

Walking: variation of stride length

- Variation: small or large steps

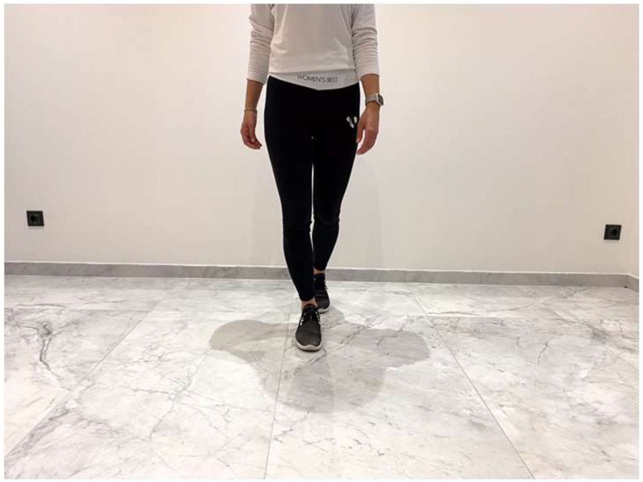

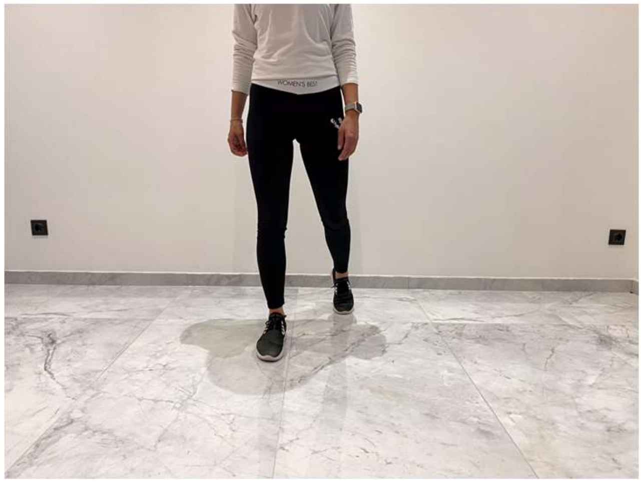

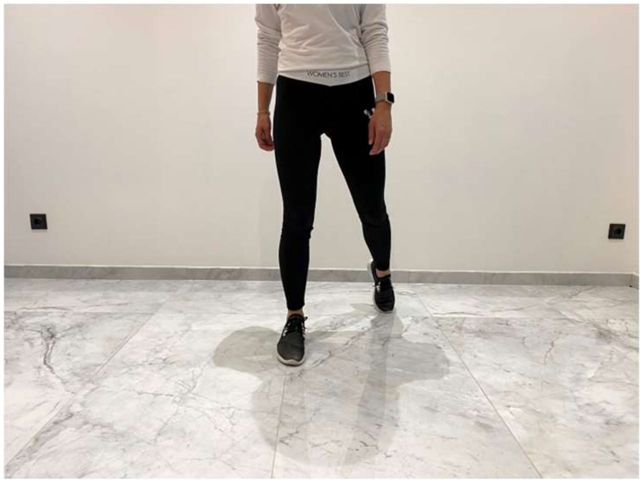

13 B

Walking: variation of the stride width

- Variation: narrow or wide steps

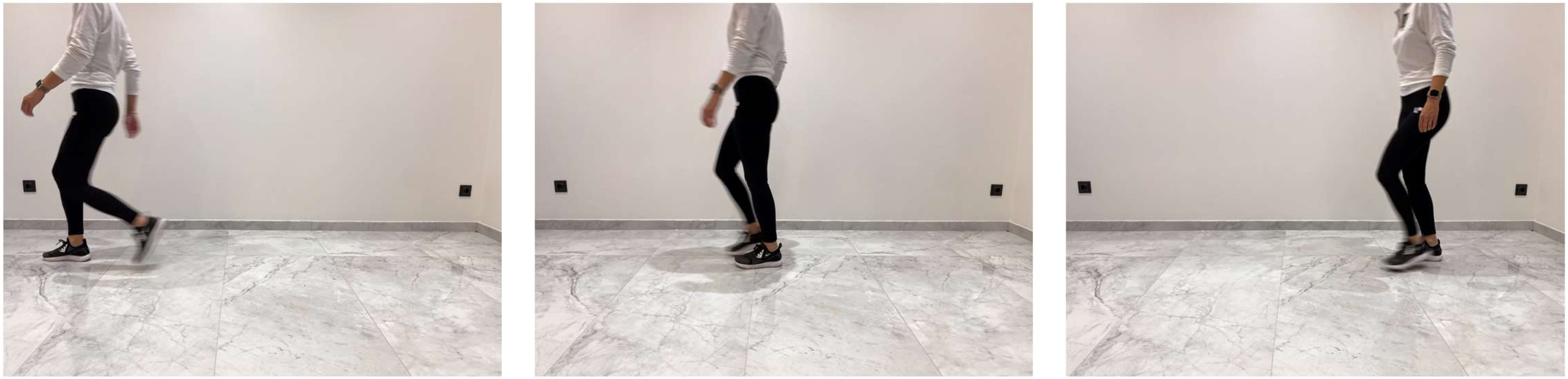

13 C

Walking: variation of gait speed

- Variation: slow or fast

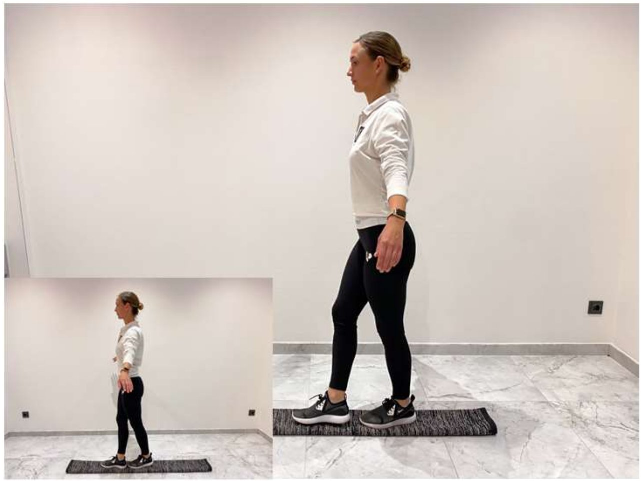
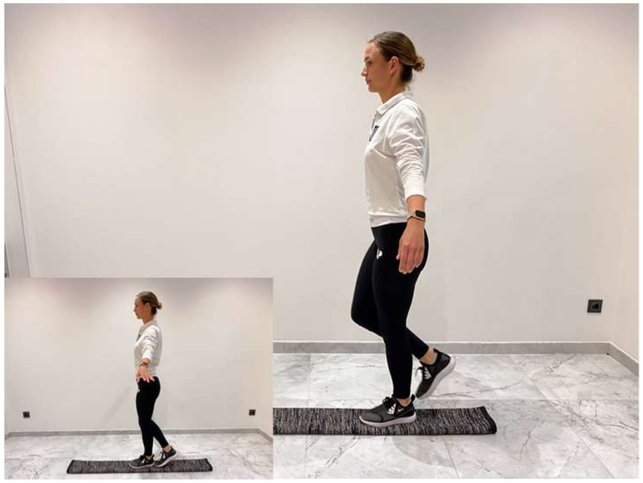
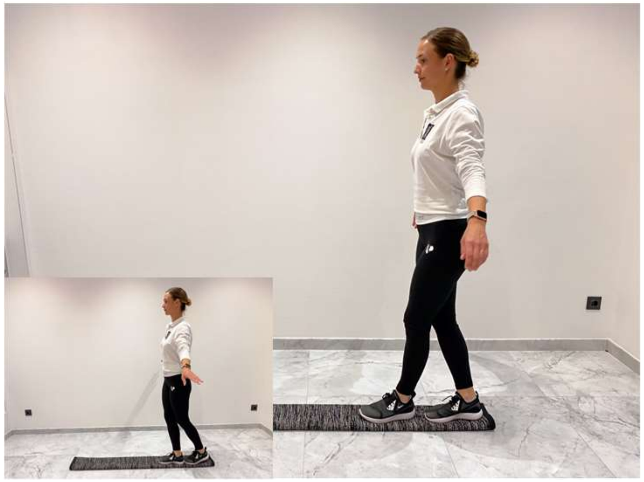

14 A

Tandem walking

- With/without support of the therapist or support of the arms (e.g., arms stretched out to the sides, holding on to a wall, etc.).

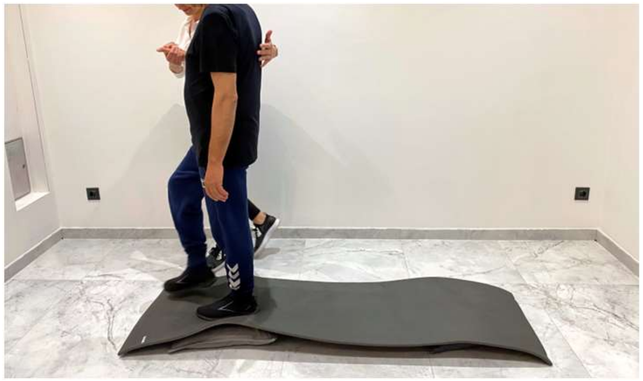

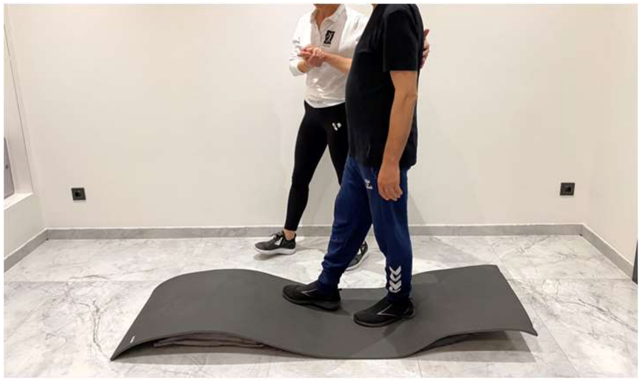

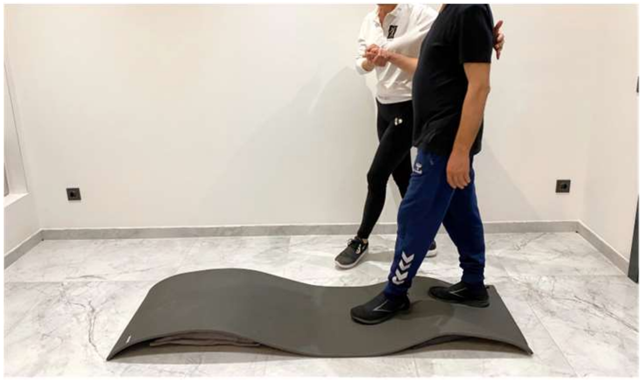

14 B

Walking on uneven ground

- If necessary, create uneven surface yourself by placing pillows under an exercise mat.
- With/without aids or assistance.

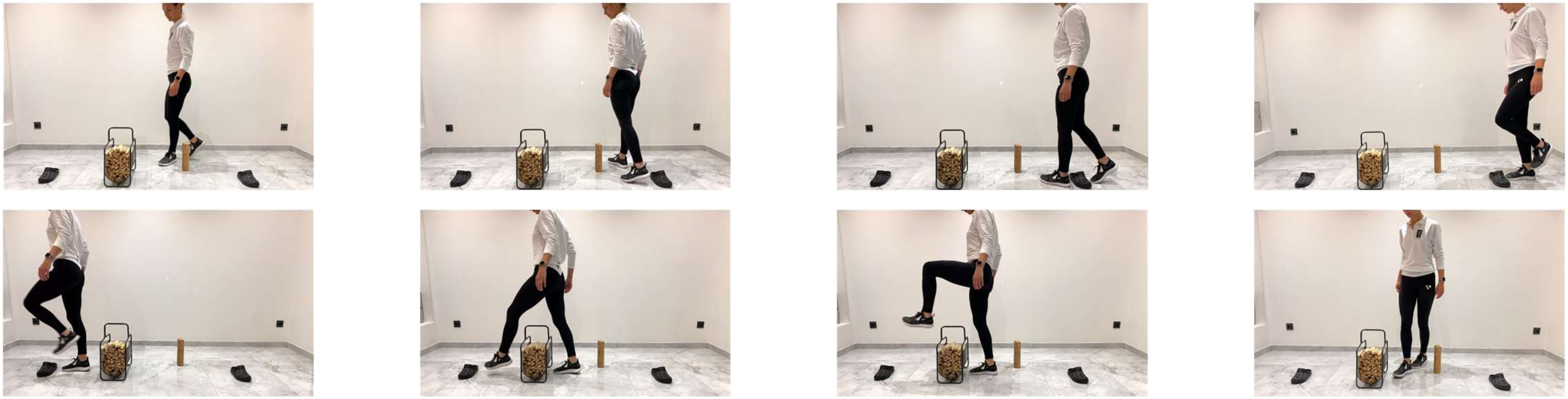

14 C

Obstacle course

- Obstacle avoidance (e.g., shoe boxes, etc.) and/or slalom walking (hats, shoes, etc.).
- With/without aids or assistance.

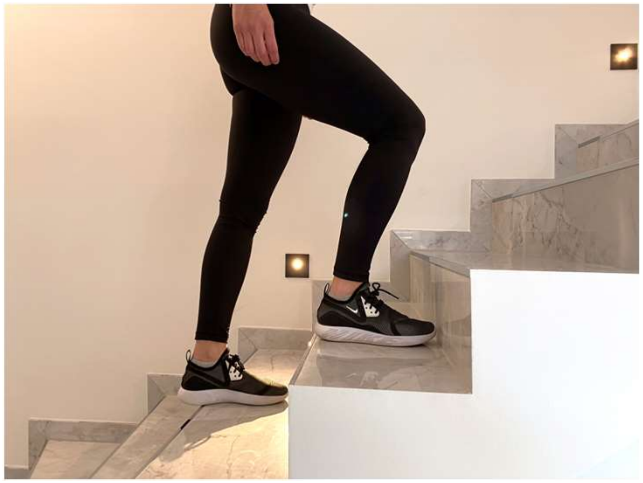

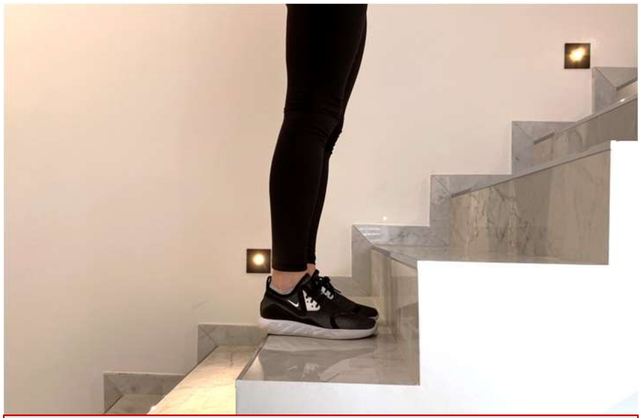

Place foot aside

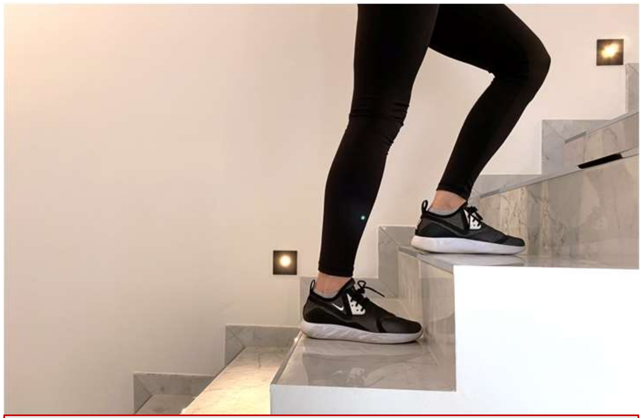

Alternating step

15 A

Stair climbing in different variations

- Step up with the right/left foot and place the other foot aside.
- Make alternating steps.
- Alternating long steps (e.g., over 2 steps, etc.).

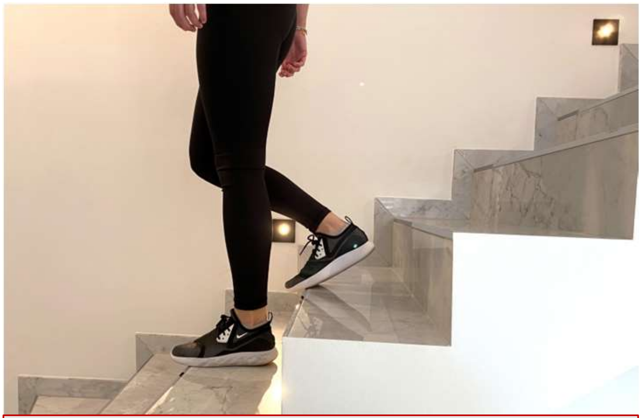

Alternating step

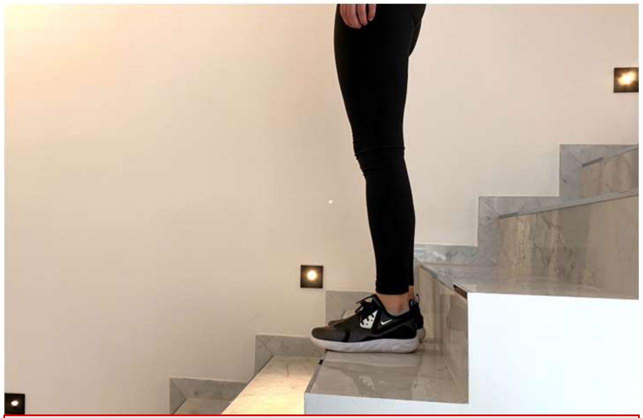

Place foot aside

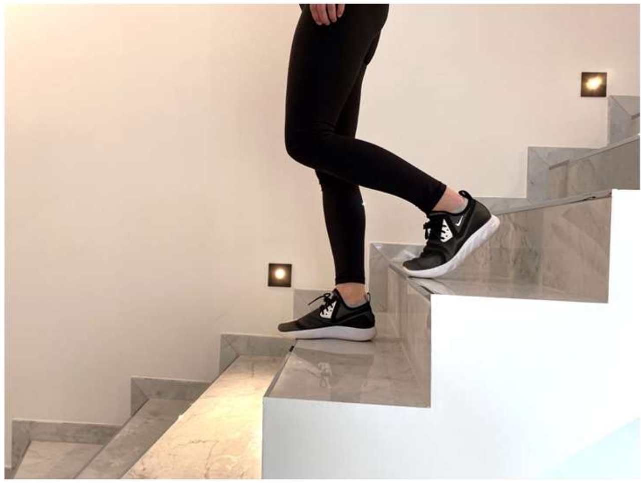

15 B

Descending stairs in different variations

- Step up with the right/left foot and place the other foot aside.
- Make alternating steps.
- Alternating long steps (e.g., over 2 steps, etc.).

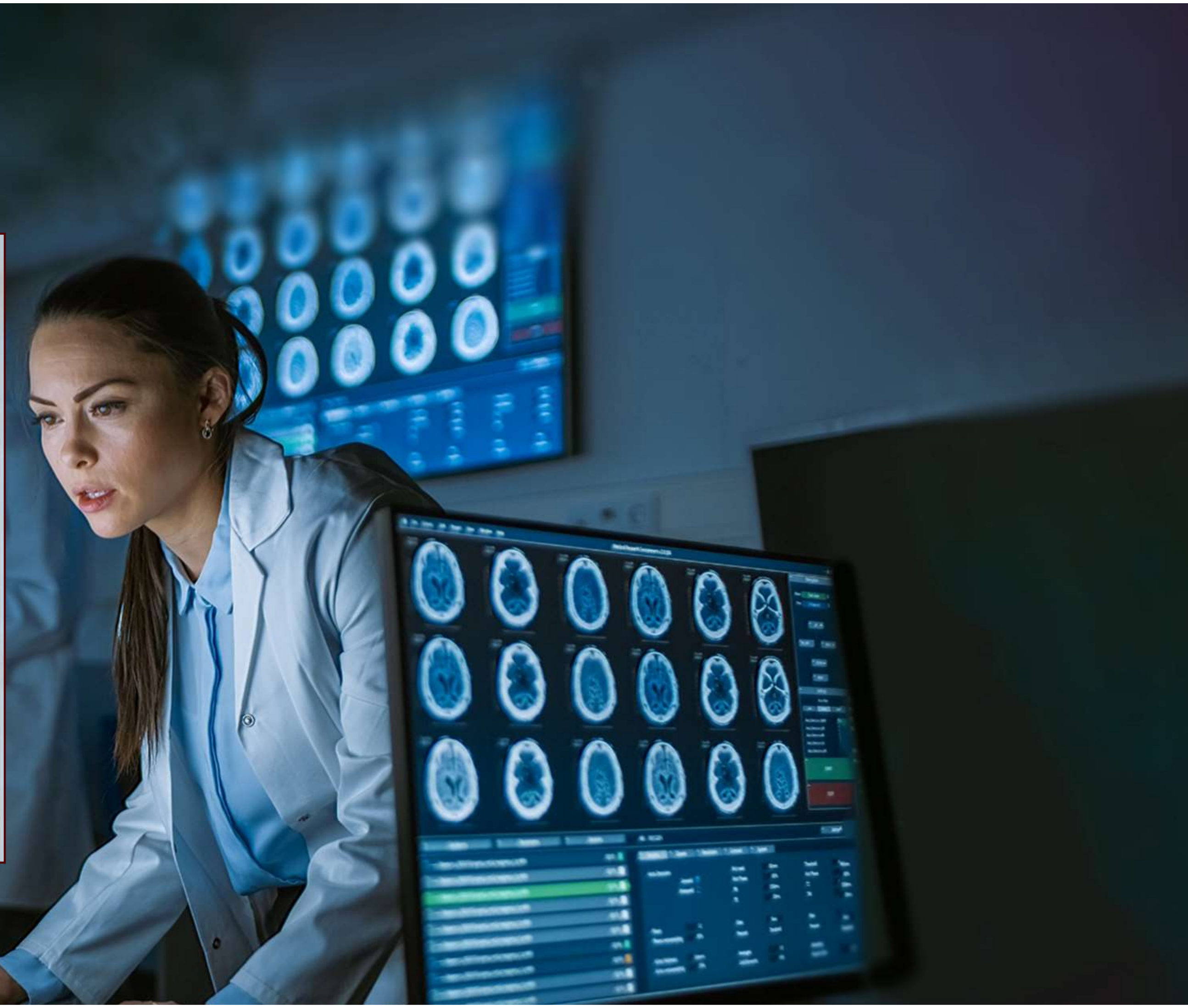

VASCage

The COMET-Centre VASCage is funded within the
COMET Programme - Competence Centres for Excellent Technologies
by

- Austrian Ministry for Climate Action, Environment, Energy, Mobility, Innovation and Technology
- Austrian Ministry of Labour and Economy

and the federal states

- Tyrol
- Salzburg
- Vienna

The COMET Programme is conducted by the Austrian Research Promotion Agency (FFG).

Federal Ministry
Republic of Austria
Labour and Economy

wirtschafts agentur wien

Tirol STANDORTAGENTUR TIROL

FFG Promoting Innovation.

LAND SALZBURG

Federal Ministry
Republic of Austria
Climate Action, Environment, Energy, Mobility, Innovation and Technology
