# Supplementary material for: Recovery of balance and walking in people with ataxia after acute cerebral stroke: study protocol for a prospective, monocentric, single-blinded, randomized controlled trial
Source: Front Stroke. 2024 Aug 5;3:1388891. doi: 10.3389/fstro.2024.1388891 (PMC12802608; doi:10.3389/fstro.2024.1388891)
Supplement: Supplementary file 6 [file Data_Sheet_6.PDF]

VASCage

GLAAS II
Standardübungen Physiotherapie für Ataxie[1]

Patricia Meier

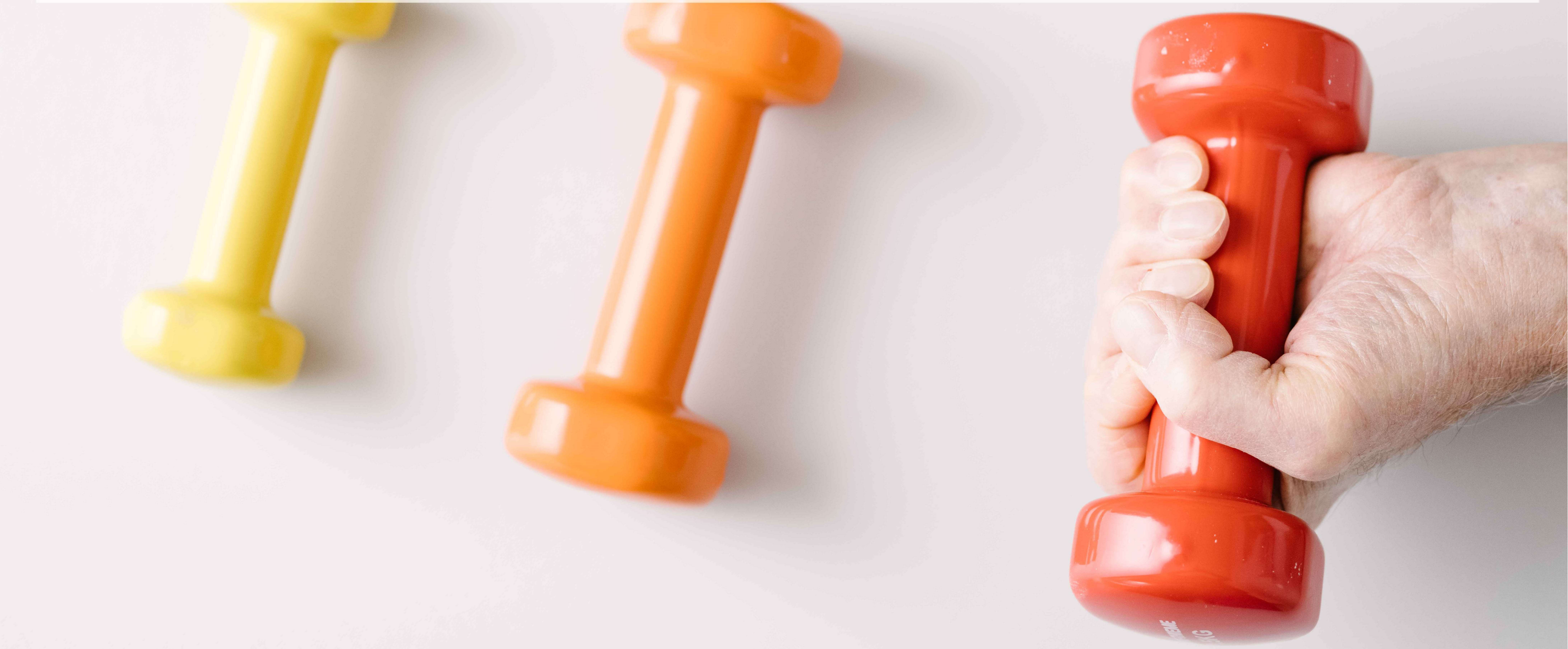

©Rene Böhmer

[1] Übungsprogramm erstellt auf Basis der "National Stroke Guidelines" der USA und der UK und reevaluiert durch Fragebögen in den teilnehmenden Studienzentren in Tirol.

Zentrale Aspekte und Prinzipien

- Die Ziele und die Übungen in der Therapie sollen alltagsrelevant sein.
- Die Übungen fokussieren auf ganze Handlungsabläufe und komplexe Bewegungen.
- Die Übungen sollen trotzdem angemessen oft wiederholt werden.
- Übungen sollen an die Leistungsgrenze des Patienten* angepasst sein, gegebenenfalls kann der Patient aber durch den Therapeuten oder ein Hilfsmittel unterstützt werden.
- Übungen, die sich nicht auf das Training von Alltagsfunktionen beziehen, fokussieren auf das statische Gleichgewicht und die Kräftigung des Rumpfes.
- 20 Therapieeinheiten (5 pro Woche) zu je 45min. & 60 Einheiten eigenständiges Training (5 pro Woche) zu je 15min.

*In diesem Übungsprogramm wird generell – aus Gründen der einfacheren Lesbarkeit – die männliche Form verwendet. Selbstverständlich sind jedoch männliche und weibliche Personen gleichermaßen gemeint.

VASC••age

Übungskategorien

- **Rumpfstabilitätstraining:**
  Übungen zur Kräftigung oder segmentalen Stabilisation des Rumpfes
- **„Activities of daily living“ (ADL) Training:**
  Training von alltäglichen Aktivitäten für Mobilität und Selbstversorgung, gegebenenfalls mit Hilfsmittel
- **Gleichgewichtstraining:**
  Gewichtsverlagerung und/oder Veränderung der Unterstützungsfläche statisches Gleichgewicht steht im Vordergrund
- **Gangtraining:**
  Training des Gehens mit Hilfsperson, Hilfsmittel oder eigenständig, einschließlich Stiegen steigen und unebenem Gelände

Steigerung und Variationen (1)

- Rumpfstabilitätstraining:
  Beginn mit Übungen der segmentalen Stabilisation des Rumpfes im Liegen, dann:
  - Steigerung der Ausgangsposition
  - Einbeziehung der Extremitäten (z.B. Becken heben in RL & 1 Bein angehoben)
  - steigern Sie nur, wenn der Pat. im Rumpf stabil bleiben kann!
- „Activities of daily living" Training:
  - im Liegen, Sitzen, Stehen, Gehen - gegebenenfalls mit Hilfsmittel
  - Einbezug einer oder mehrerer Extremitäten
  - Von einfachen zu komplexen Handlungen

Steigerung und Variationen (2)

- Gleichgewichtstraining:
  - Übungen auf der Liege/ am Bett, im Sitzen, im Stehen
  - mit instabilen Unterlagen
  - durch Verringerung der Unterstützungsfläche
  - statisches Gleichgewicht steht im Vordergrund!
- Gangtraining:
  - mit Hilfsperson, Hilfsmittel, eigenständig
  - mit Variation der Schrittlänge, Gehgeschwindigkeit, Spurbreite
  - Gangtraining im Parkour, auf Stiegen, im unebenen Gelände

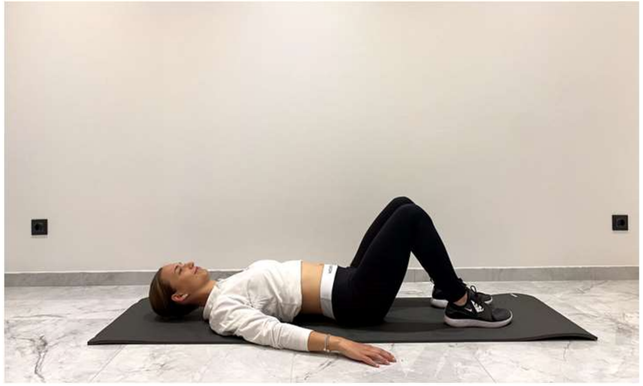
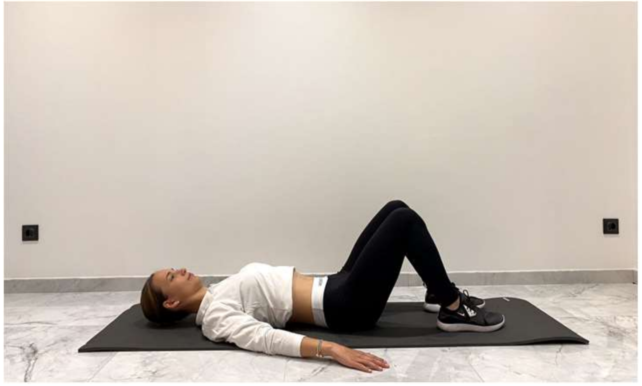
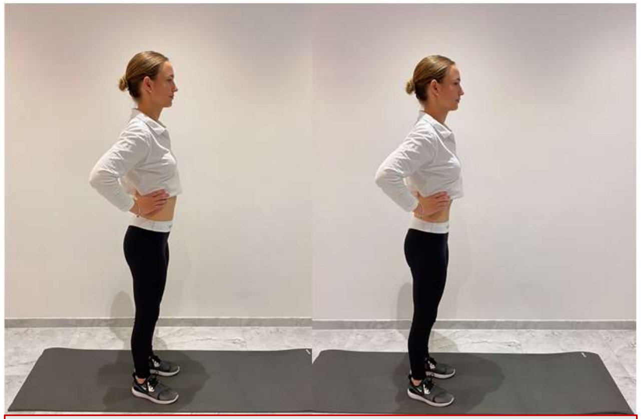

Variation

1 A

Bauchnabel einziehen

- Gegebenenfalls Kontrolle der Aktivität mittels „Pressure-Biofeedback-Unit"
- In Rückenlage, Seitenlage, Sitzen, Stehen: dabei eine oder mehrere Extremitäten bewegen
- Im Gehen

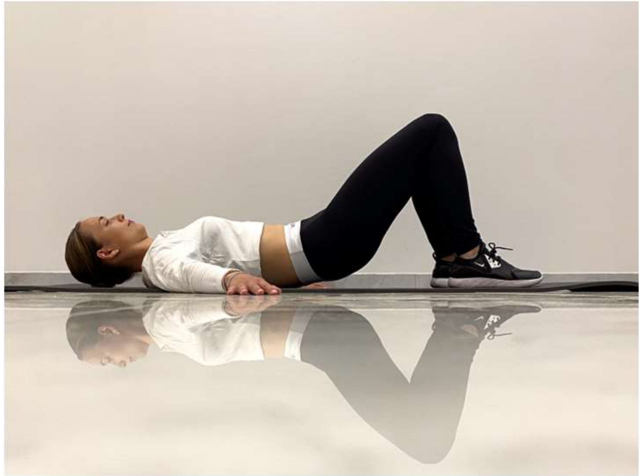

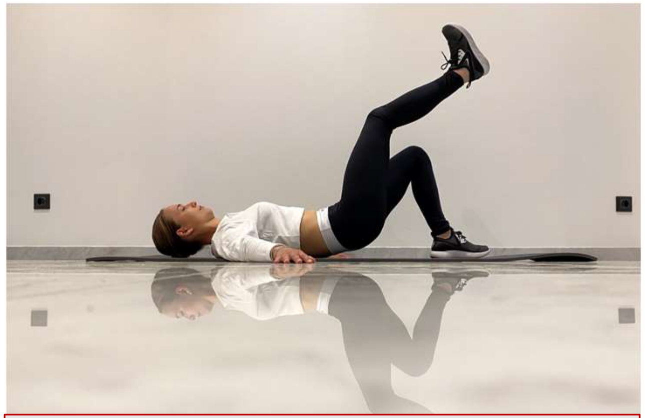

Variation 1

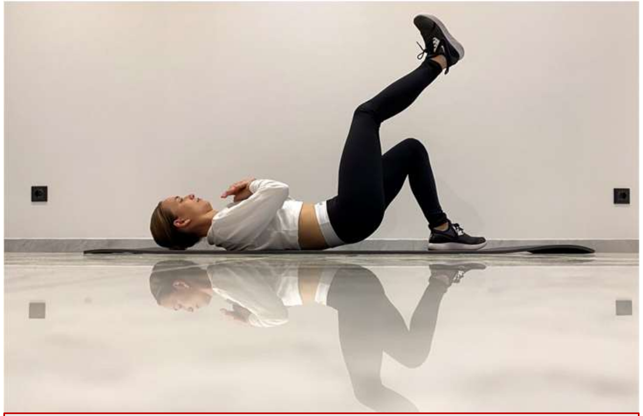

Variation 2

1
B

Rückenlage, Beine aufgestellt:
Bauchnabel anziehen & Gesäß leicht anheben

- Bei der Ausführung ist auf einen stabilen Rumpf zu achten
- Position zuerst kurz, dann immer länger halten probieren
- Variation: linkes/rechtes Bein abgehoben, ohne aufstützen der Arme, etc.

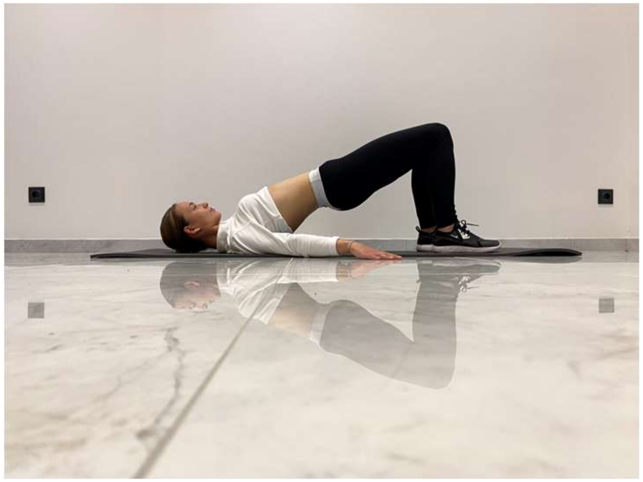

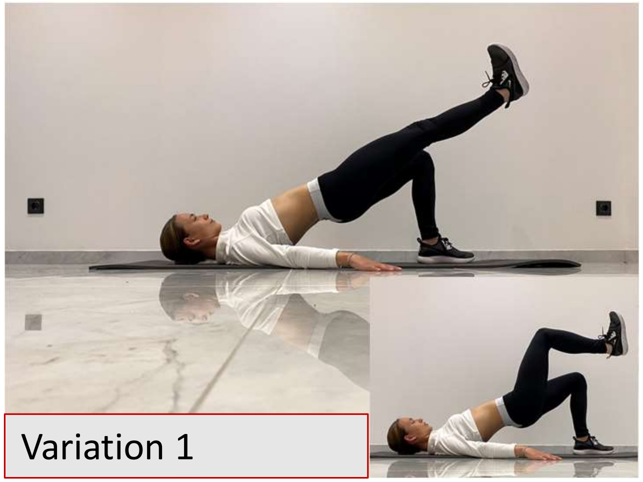

Variation 1

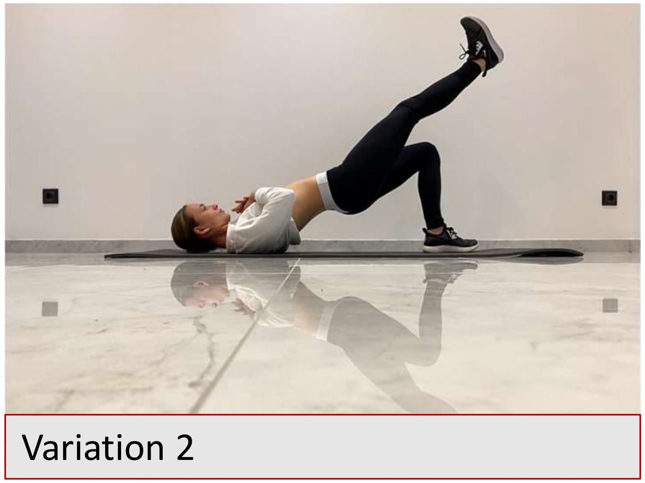

Variation 2

1 C

Rückenlage, Beine aufgestellt: Bauchnabel anziehen & Gesäß so weit wie möglich anheben (Bridging)

- Position halten und auf stabilen Rumpf achten
- Variation: linkes/rechtes Bein abheben, ohne aufstützen der Arme, etc.

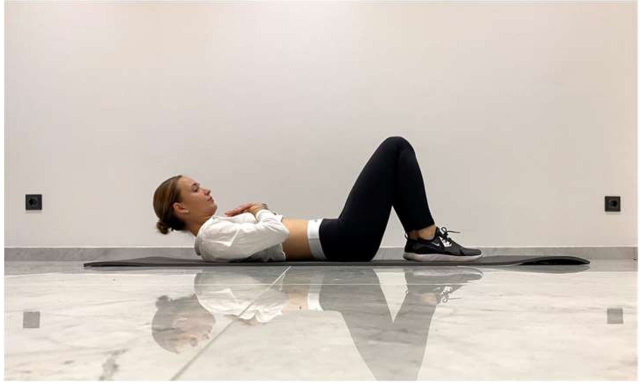

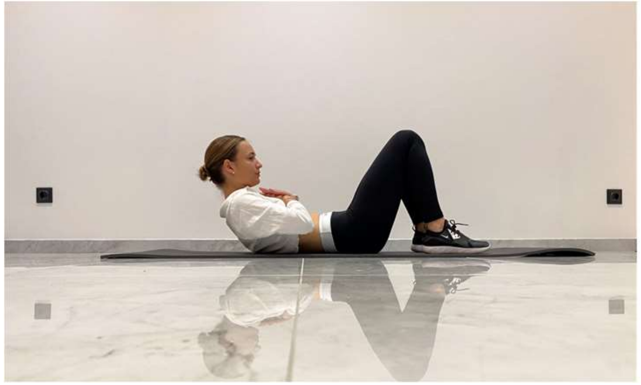

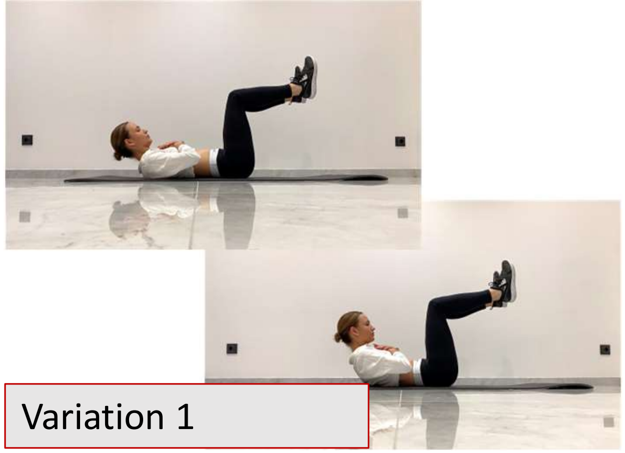

2
A

Rückenlage, Füße angezogen: Sit-ups gerade

- Variation: Beine dabei vom Boden abgehoben

Variationen 2-5

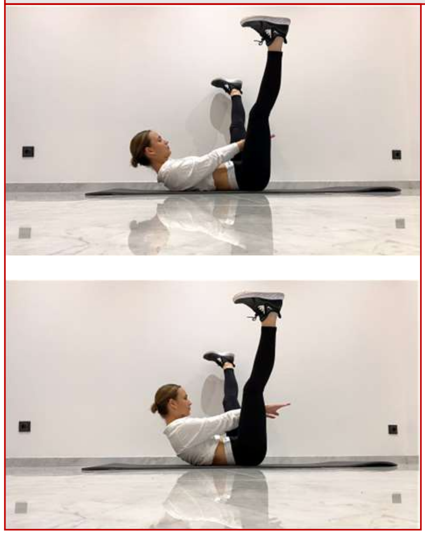
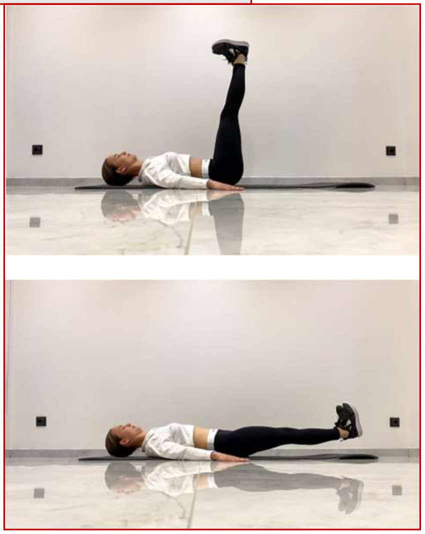
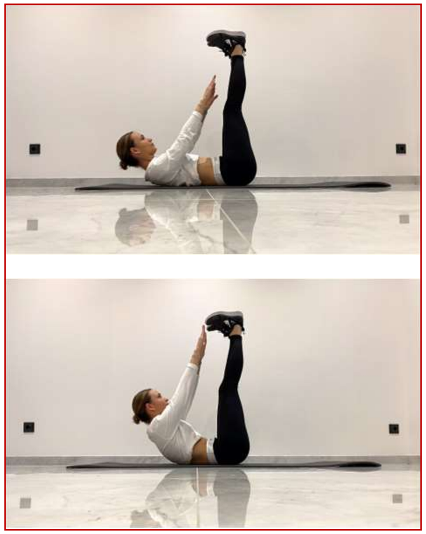
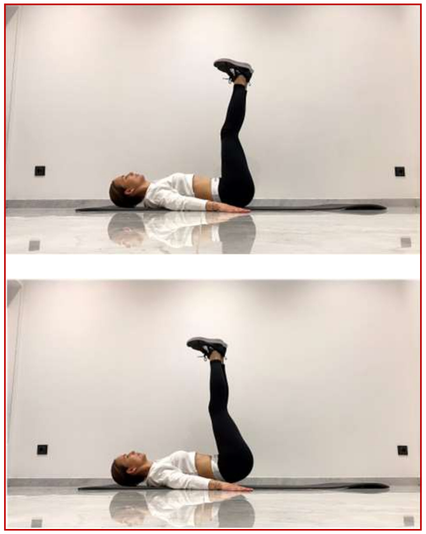

2
A

- Weitere Variationen:

Beine breit zur Decke ausgestreckt und zwischen den Füßen durchlangen
Beine zur Decke ausgestreckt und von oben nach unten bewegen (für Fortgeschrittene – und nur soweit es geht!)
Beine zur Decke ausgestreckt und Zehenspitzen berühren
Beine zur Decke ausgestreckt und das Gesäß anheben.

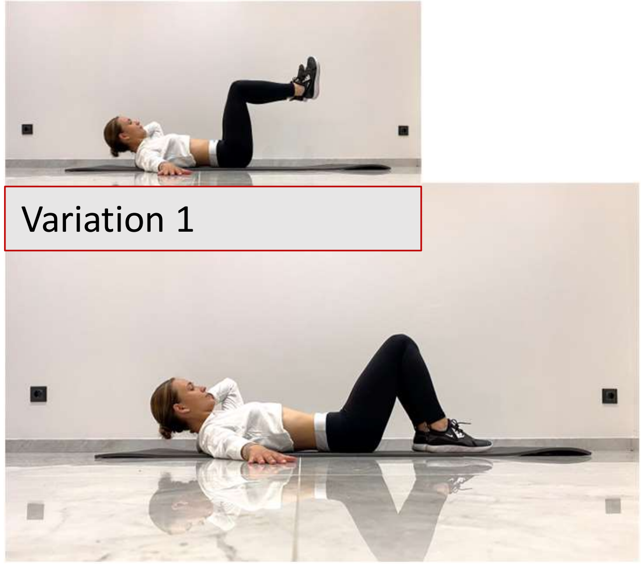

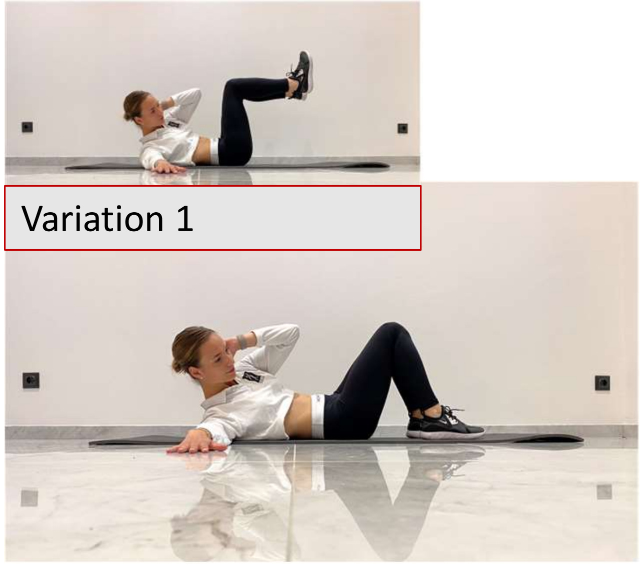

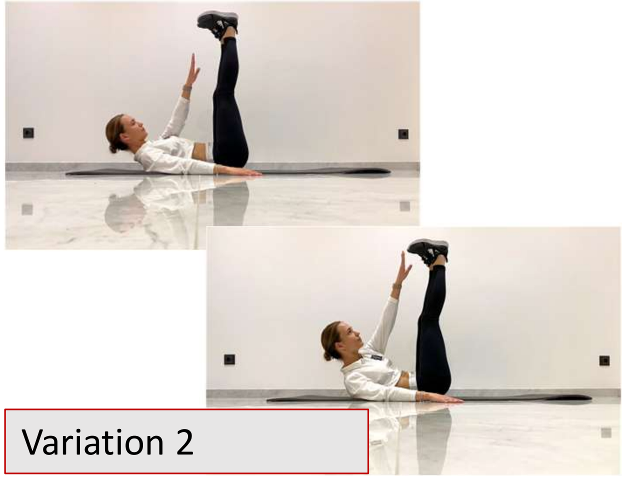

2 B

Rückenlage: Sit-ups schräg

Der rechte Ellbogen berührt das linke Knie, der linke Ellbogen das rechte Knie.

- Variation: Beine sind dabei ausgestreckt, Beine zeigen zur Decke und Zehenspitzen berühren

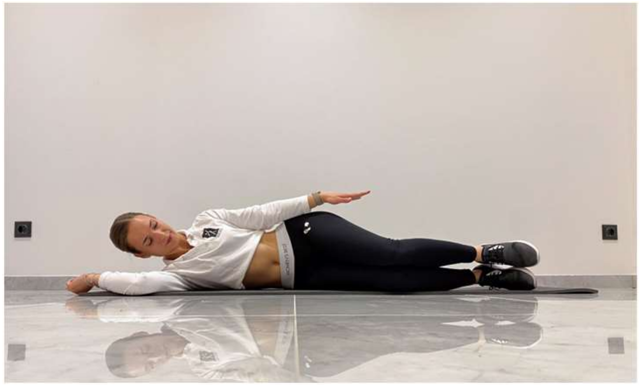

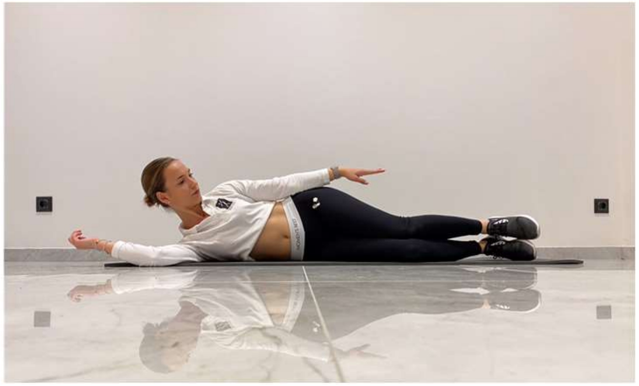

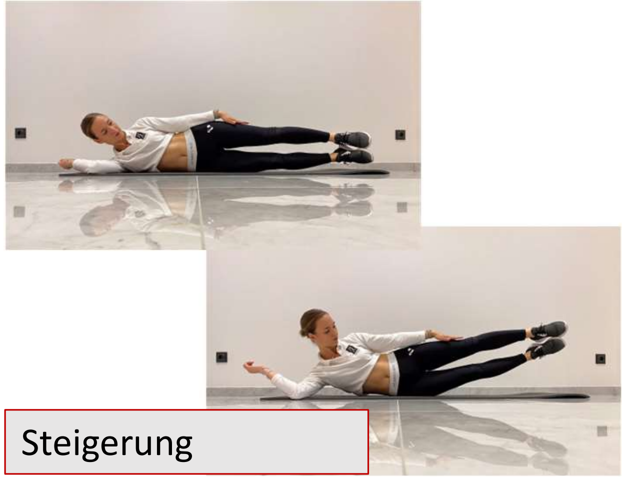

Steigerung

2 C

Seitenlage: Sit-ups seitlich

- Steigerung: auch die Beine können zugleich mit angehoben werden

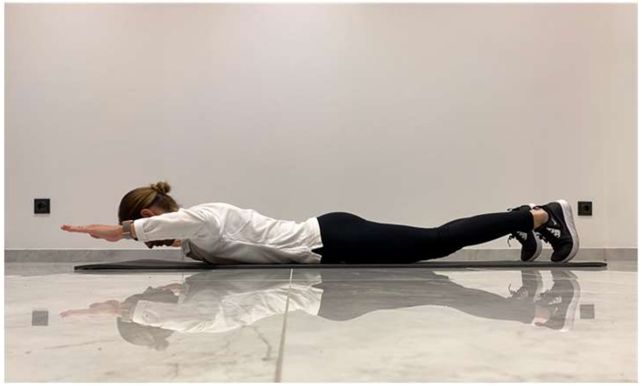

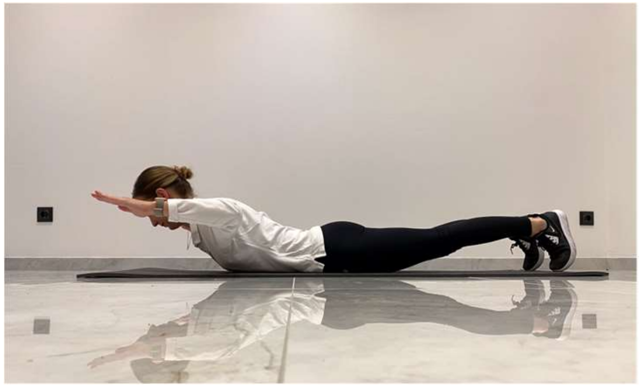

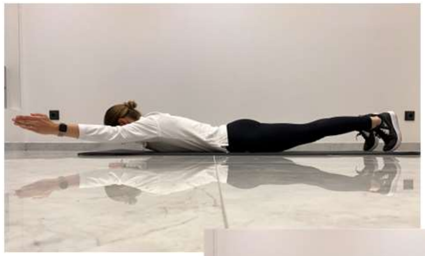

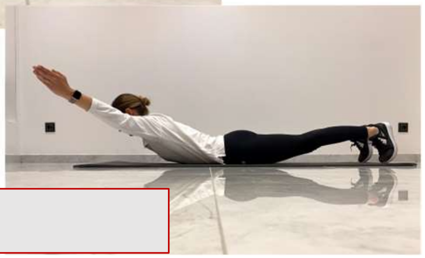

Steigerung

3 A

Bauchlage: Oberkörper anheben

- Variation: Position halten, verschiedene Positionen der Arme (T, W, I, V)
- Steigerung: die Arme sind dabei über dem Kopf nach vorne ausgestreckt (I oder V nach oben/vorne)

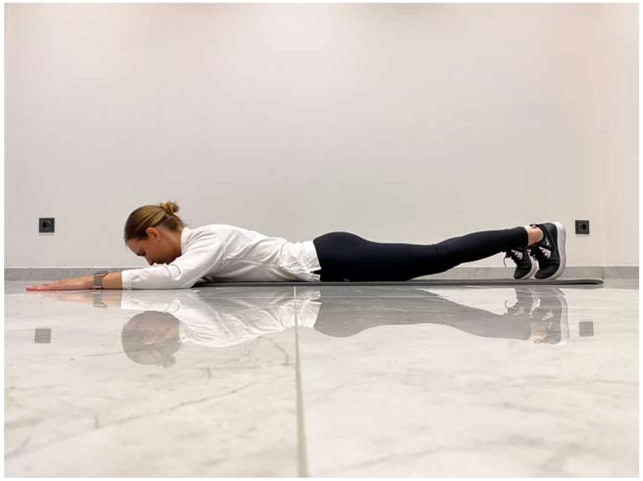
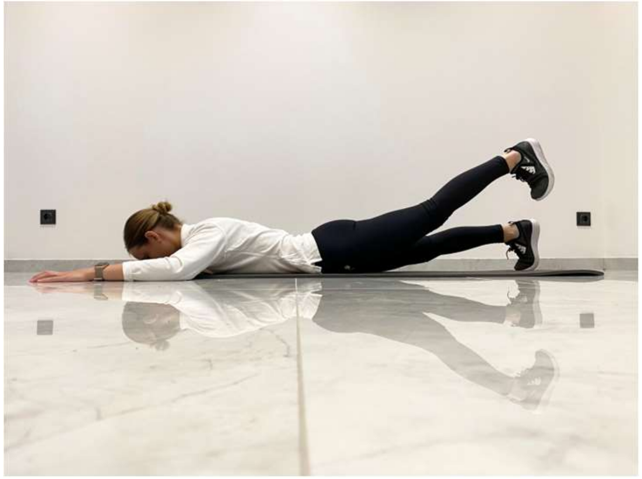
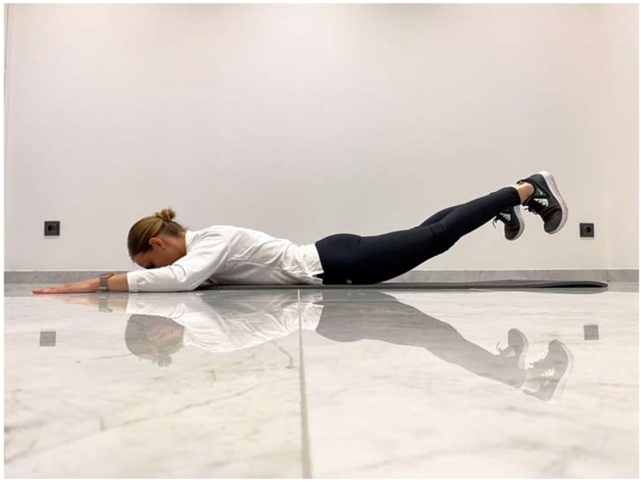

3 B

Bauchlage: ein oder beide Beine anheben

- Das Bein soll so weit wie möglich angehoben werden (am besten mit gestrecktem Knie), wobei das Becken stabil am Boden bleiben soll

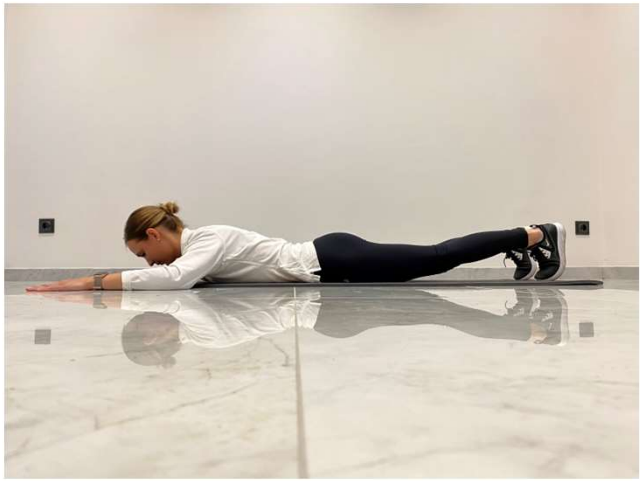

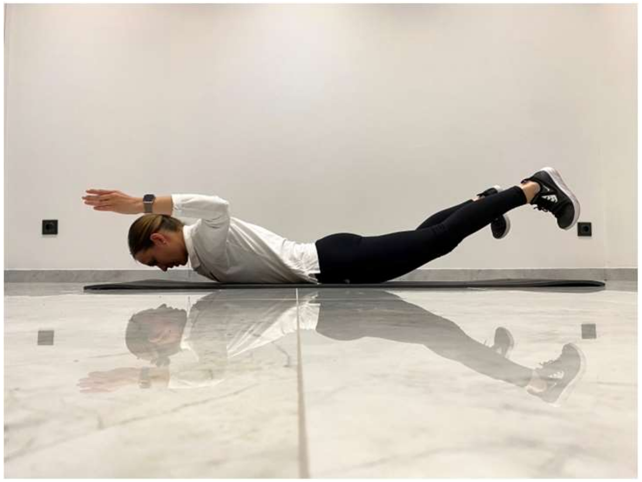

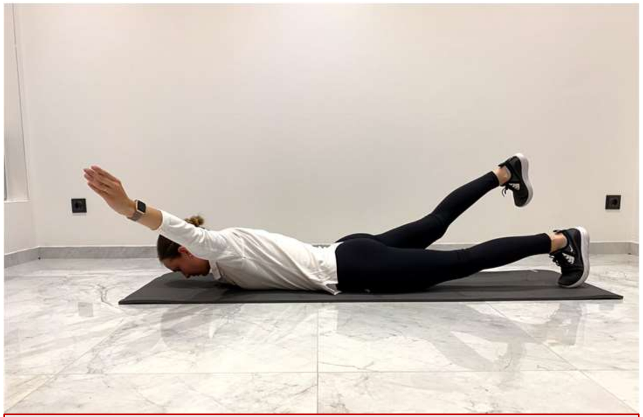

Variation

3
C

Bauchlage: Arme und Beine anheben

- Das Becken bleibt auch bei dieser Übung immer stabil am Boden
- Variation: gegengleich (linker Arm und rechtes Bein), abwechselnd (erst Arme, dann Beine)
- Steigerung: zusätzlich zu den Armen und Beinen auch den ganzen Oberkörper abheben

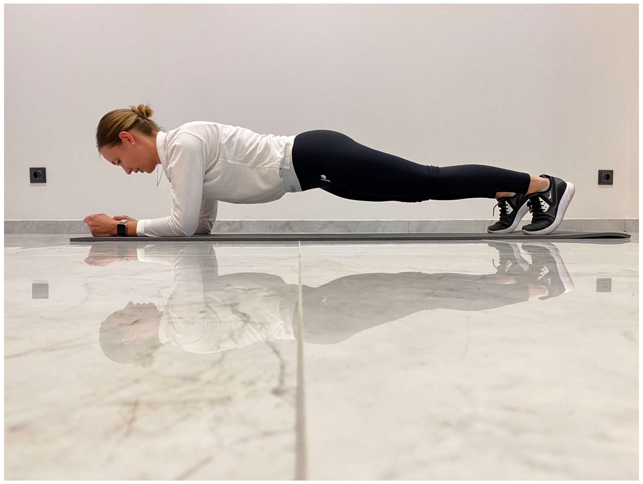

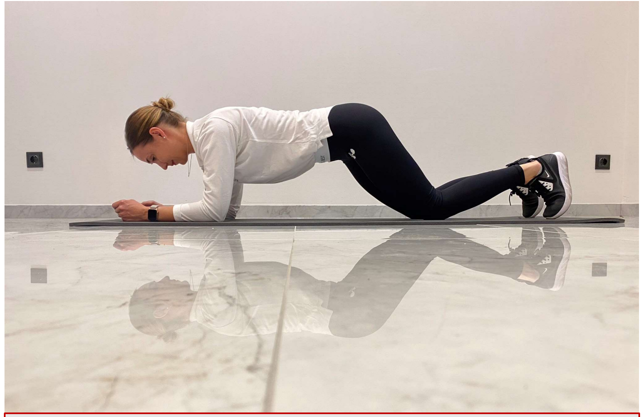

Variation 1

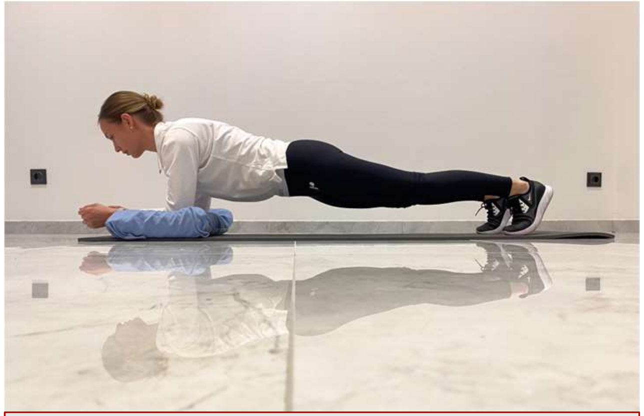

Variation 2

4 A

Plank mit Unterarmstütz: Position für 15 Sekunden halten

- Mögliche Variation: leichter: Knie können in der Unterarmstütz-Position am Boden abgelegt sein; schwerer: Unterarmstütz-Position mit instabiler Unterlage unter den Unterarmen und/oder Füßen

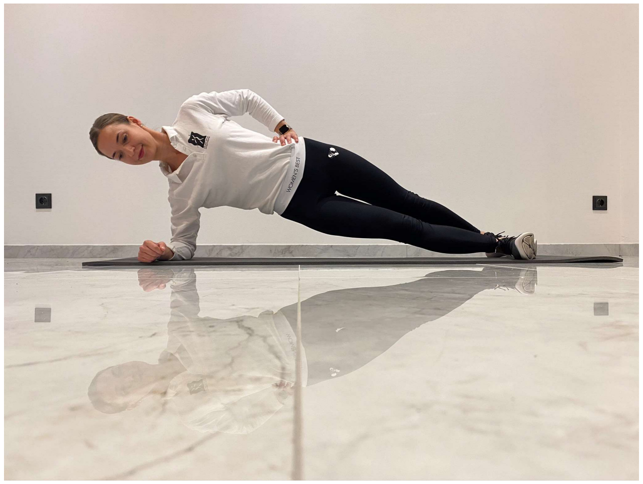

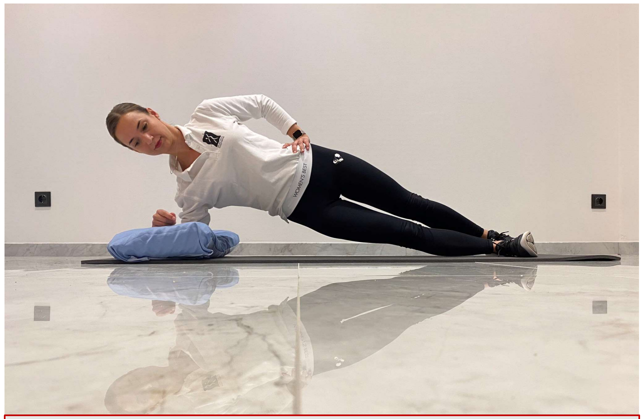

Variation 1

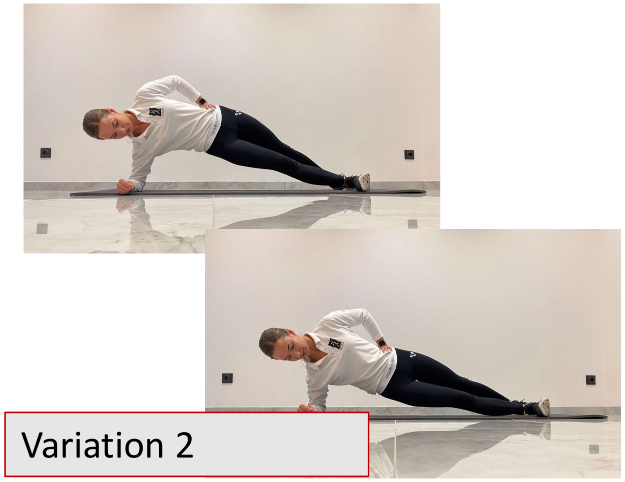

Variation 2

4
B

Seitliche Plank mit Unterarmstütz: Position für 15 Sekunden halten

- Mögliche Variation: leichtes Heben und Senken des Beckens, instabile Unterlage unter dem stützenden Arm oder Fuß

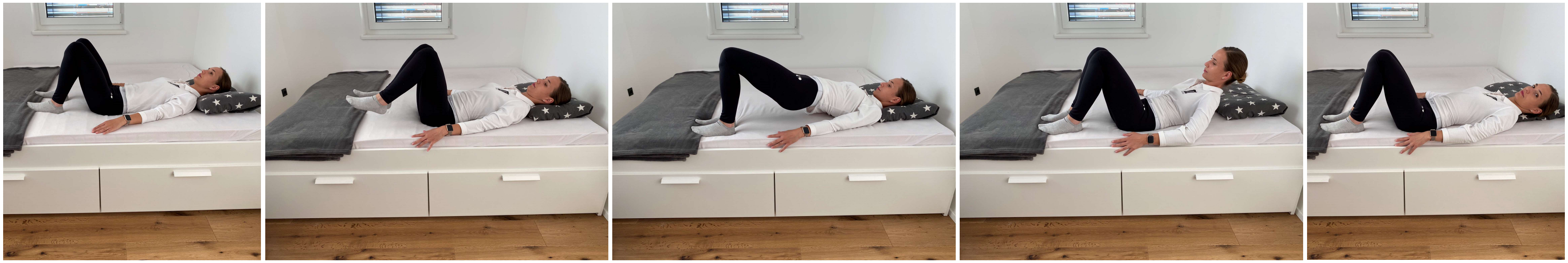

5 A

Liegend im Bett: an den linken/rechten Bettrand rutschen

- Falls notwendig, kann Unterstützung durch Hilfsmittel oder den Therapeuten gegeben werden

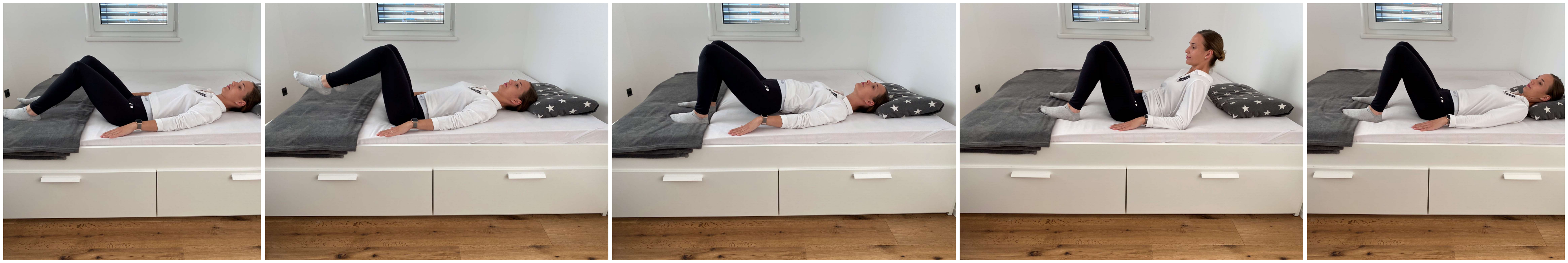

5
B

Liegend im Bett: an den oberen/unteren Bettrand rutschen

- Falls notwendig, kann Unterstützung durch Hilfsmittel oder den Therapeuten gegeben werden

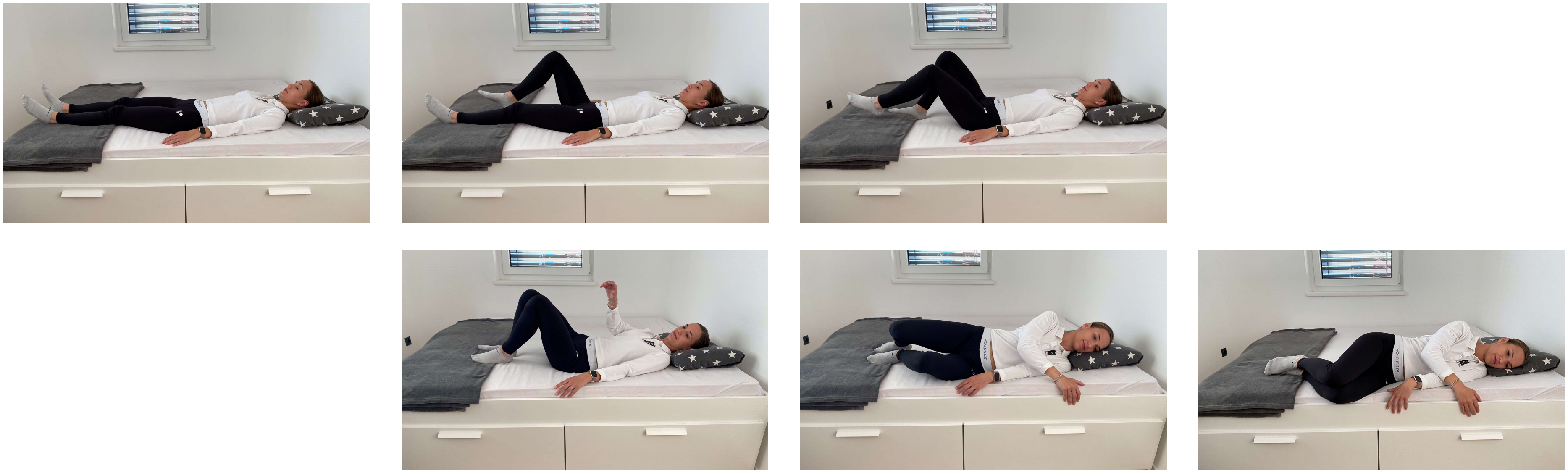

5 C

Liegend im Bett: auf die linke und rechte Seite drehen

- Falls notwendig, kann Unterstützung durch Hilfsmittel oder den Therapeuten gegeben werden

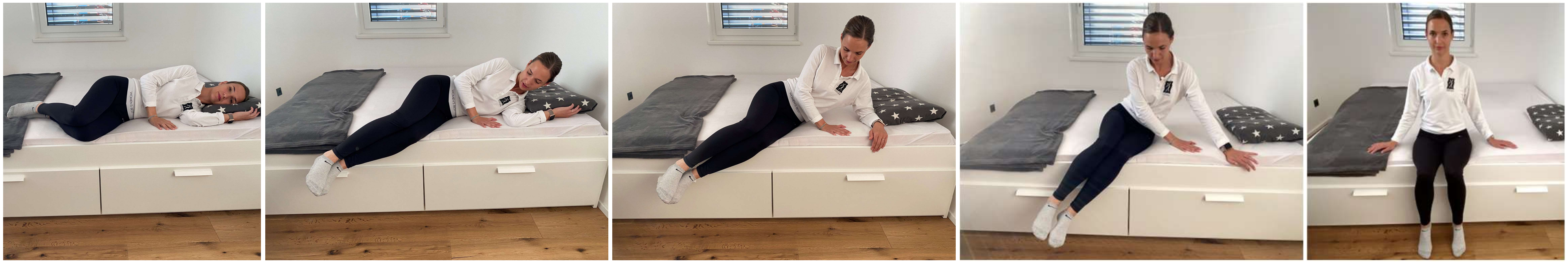

6 A

Aus dem Liegen aufsetzen & wieder hinlegen

- Falls notwendig, kann Unterstützung durch Hilfsmittel (z.B. Galgen) oder den Therapeuten (Hand zum Aufziehen) gegeben werden
- Variation: aus der Rückenlage oder über den Langsitz aufsetzen und dann die Füße aus dem Bett, von der Bauchlage aufstehen

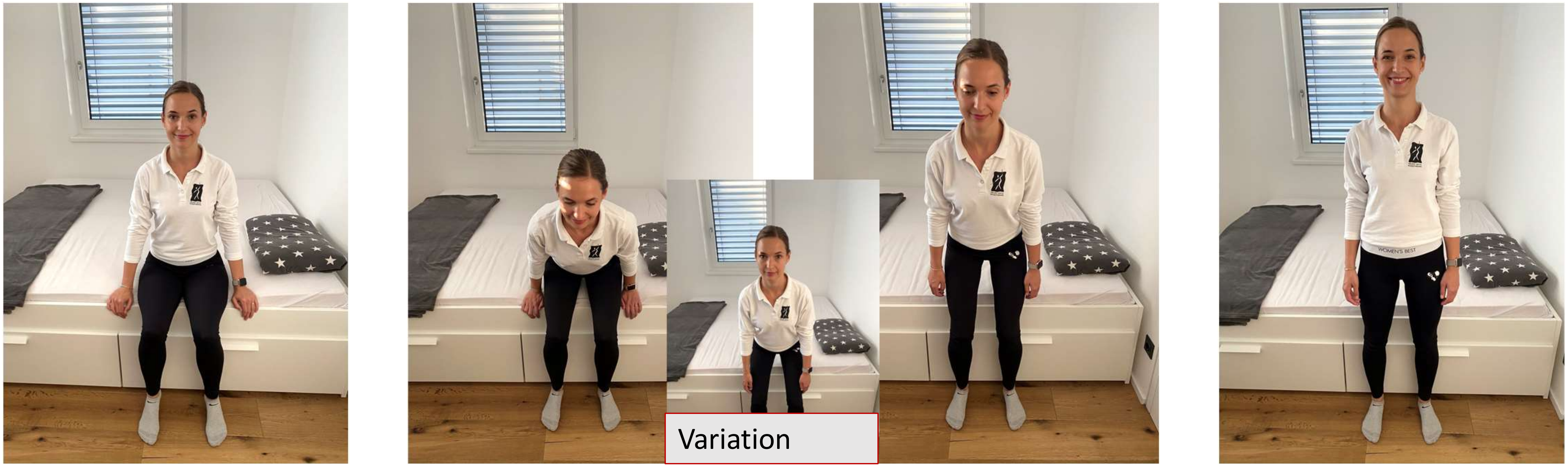

6 B

Aufstehen/hinsetzen

- Falls notwendig, kann Unterstützung durch Hilfsmittel oder den Therapeuten gegeben werden
- Variation: versch. Ausganspositionen (niedriger/erhöhter Sitz), versch. Fuß- bzw. Standbreite, ohne Einsatz der Hände

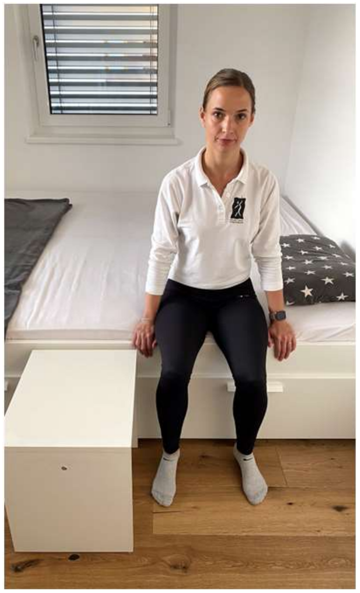
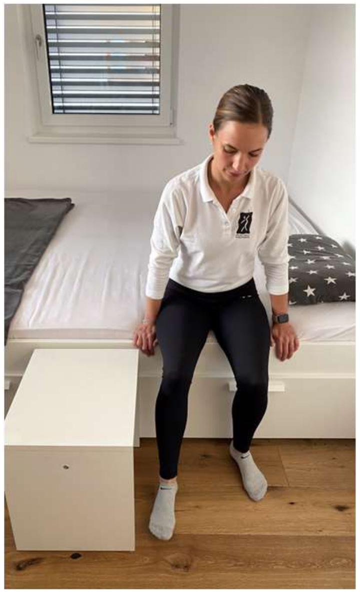
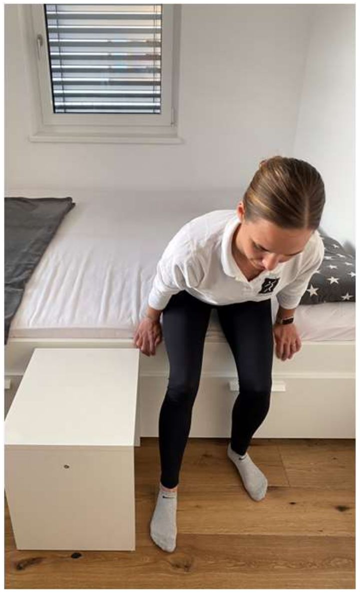
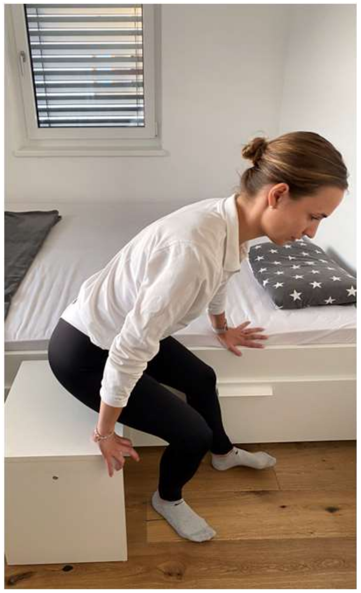
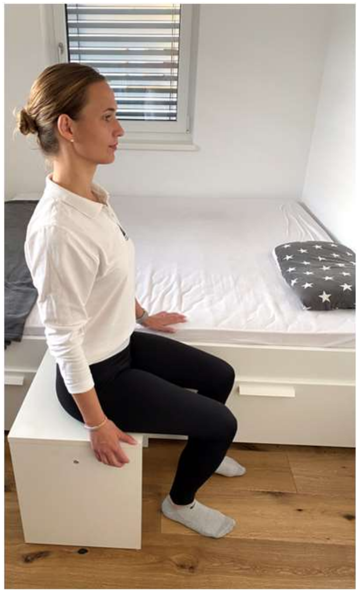

6 C

Transfer Bett – Stuhl – Bett

- Falls notwendig, kann Unterstützung durch Hilfsmittel oder den Therapeuten gegeben werden
- Variationen: Rollstuhl – Bett; Rollstuhl – WC; etc.

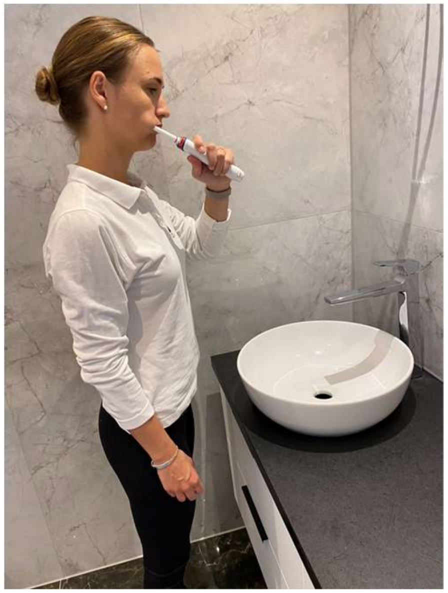

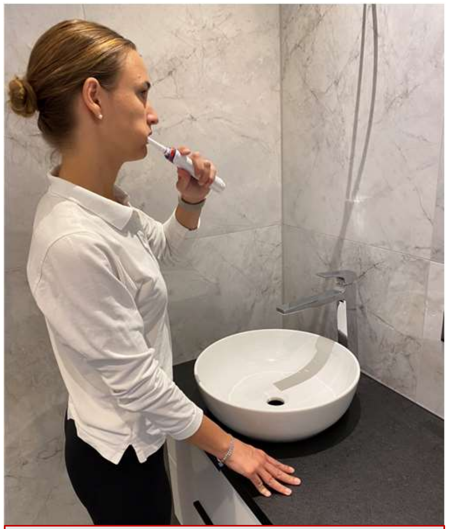

Variation

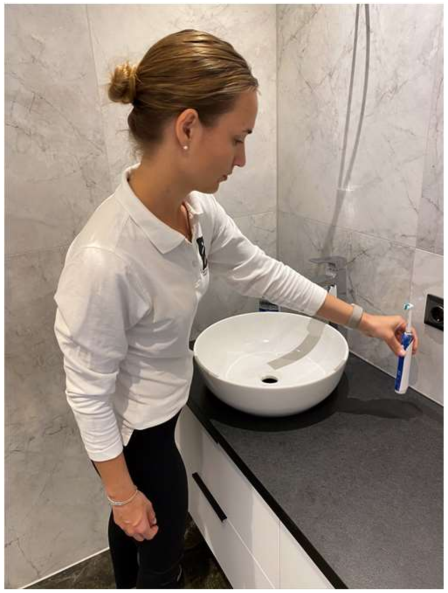

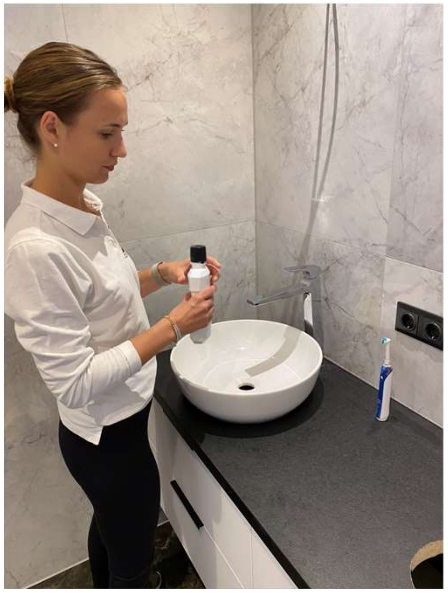

7 A

Im Sitzen oder Stehen: Zähneputzen

- Variation: Gesicht waschen oder andere Tätigkeiten der morgendlichen/ abendlichen Körperpflege, mit/ohne Festhalten

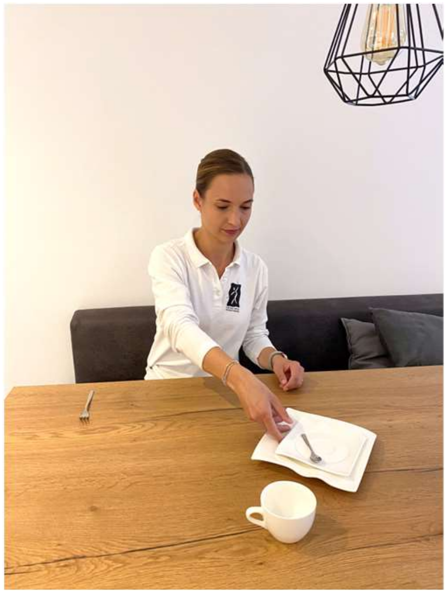

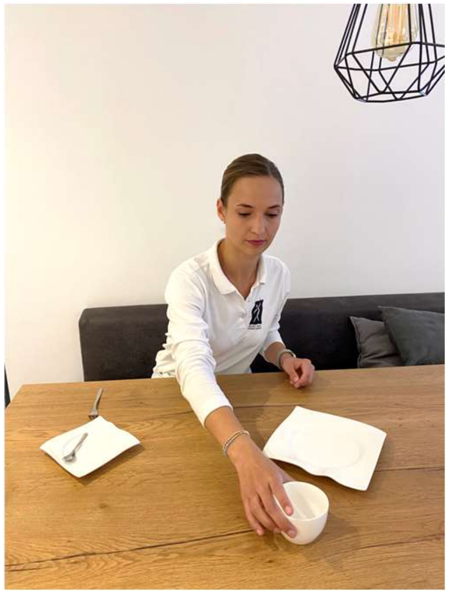

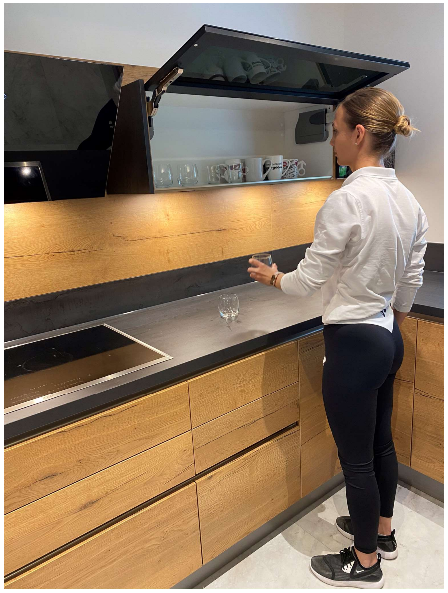

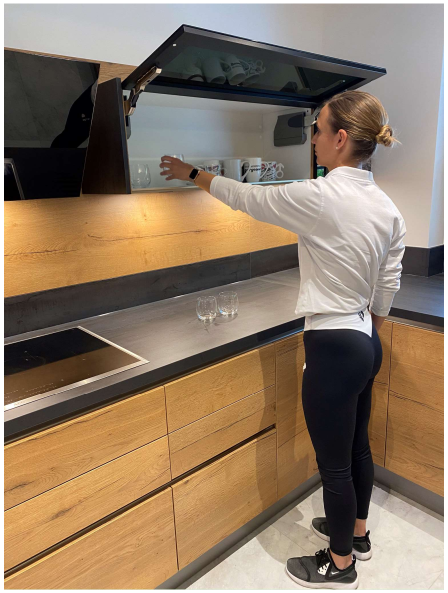

7 B

Im Sitzen oder Stehen: Gegenstände auf dem Tisch verstellen (z.B. Hütchen stapeln)

- Variation: Gegenstände ins Regal stellen oder andere Tätigkeiten, die in der Küche verrichtet werden, z.B. Kaffee/Tee kochen, ein Brot schmieren, etc.

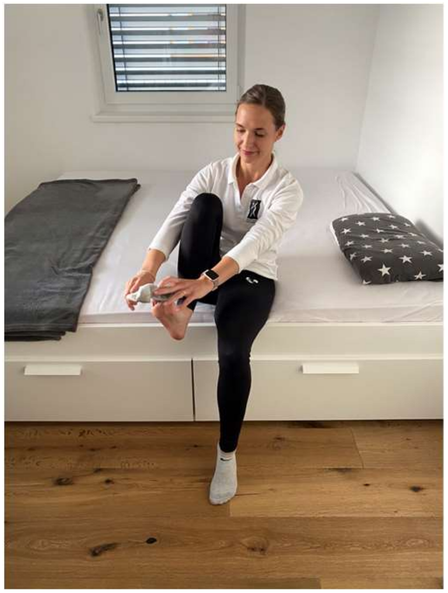
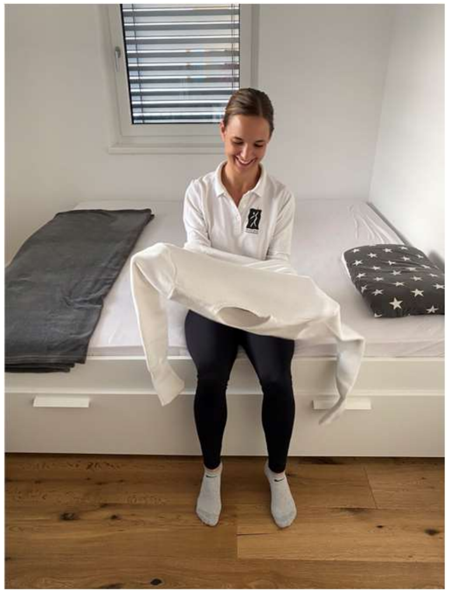
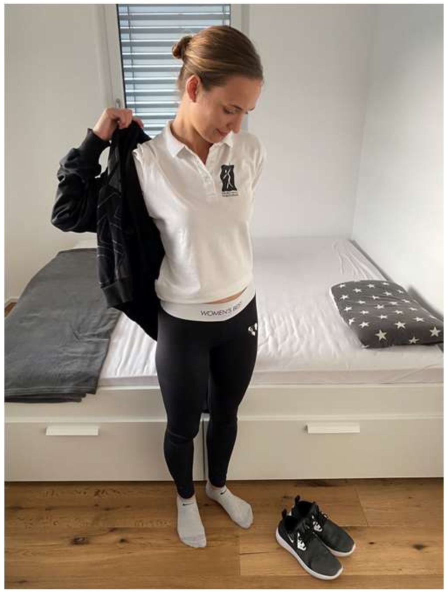
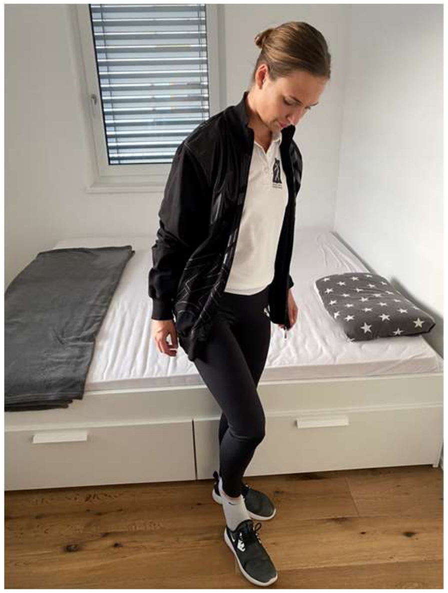

7 C

Im Sitzen oder Stehen: um- oder anziehen

- Möglichkeiten: Oberteil, Hose, Schuhe, Jacken, etc.

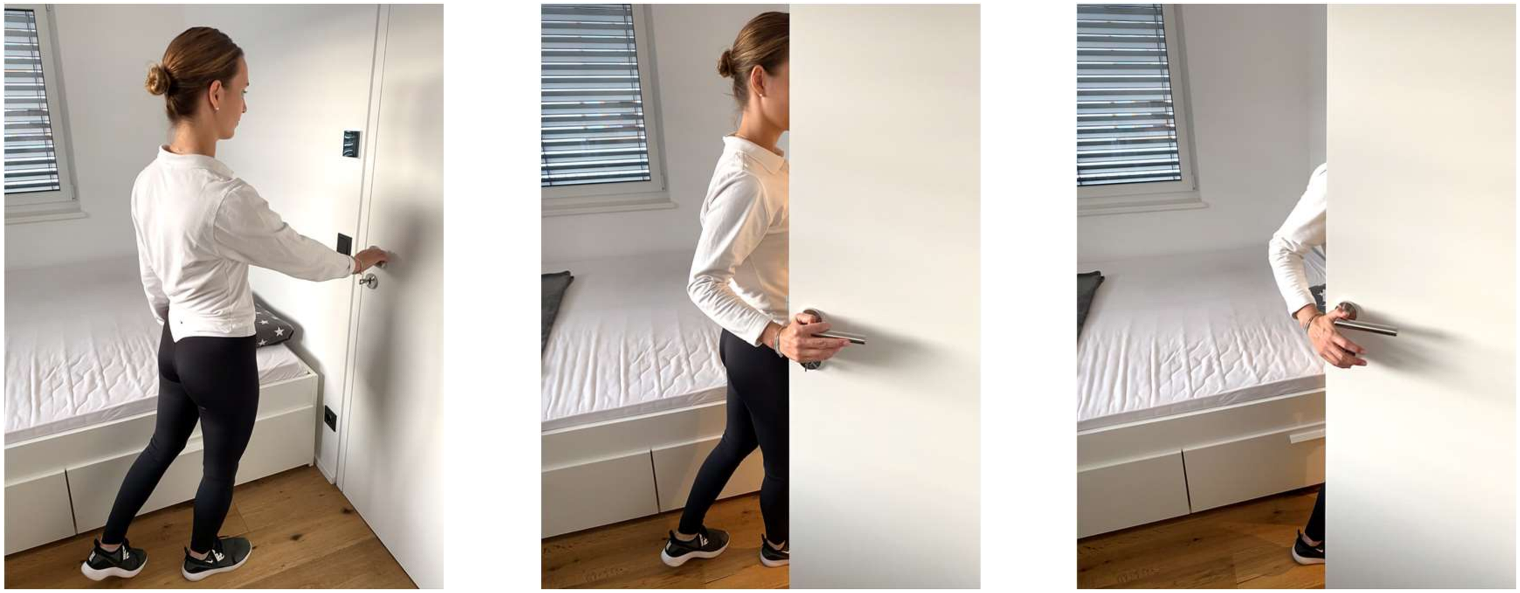

8A

Im Gehen/Stehen Türen auf und zu machen

- Die Handlung kann gegebenenfalls auch mit Hilfsmittel und Kompensationsmechanismen durchgeführt werden.

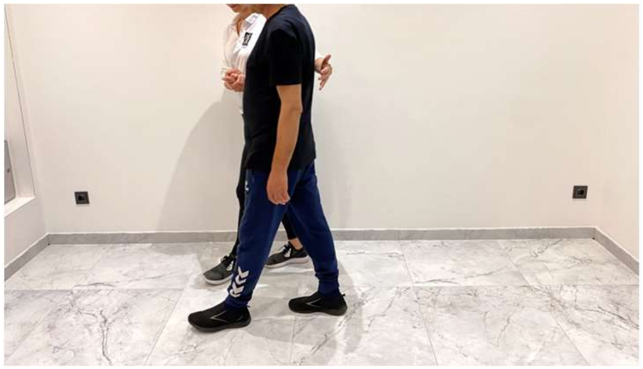

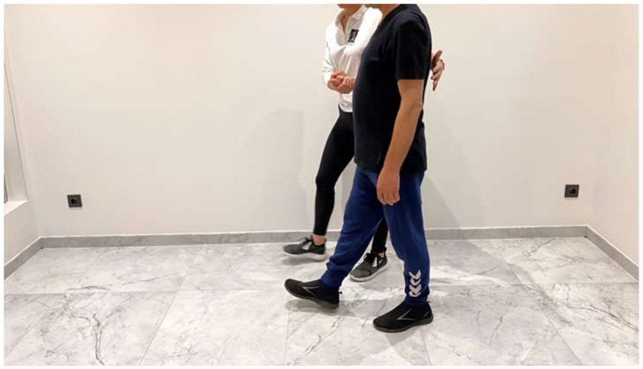

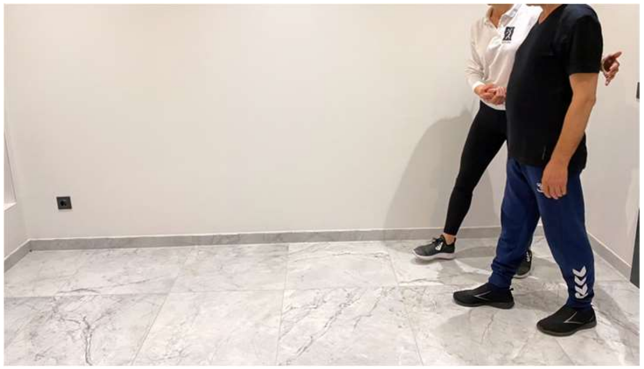

8
B

Mit/ohne Hilfsmittel
von A nach B gehen

- Die Aktivität darf gegebenenfalls auch mit Hilfsperson und Kompensationsmechanismen durchgeführt werden.

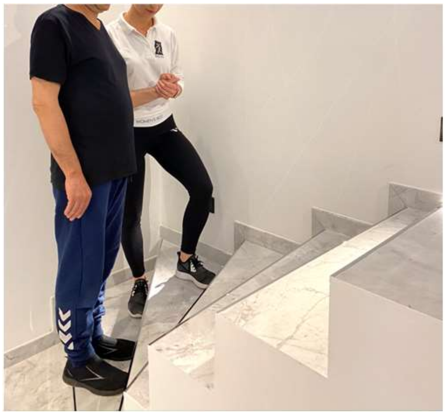
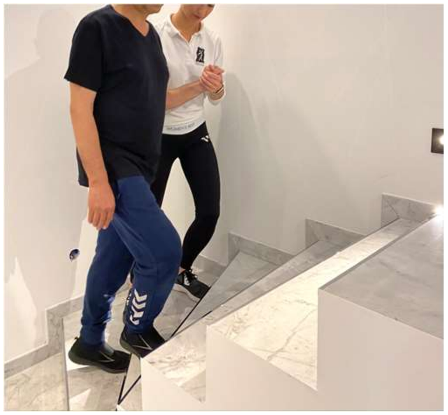
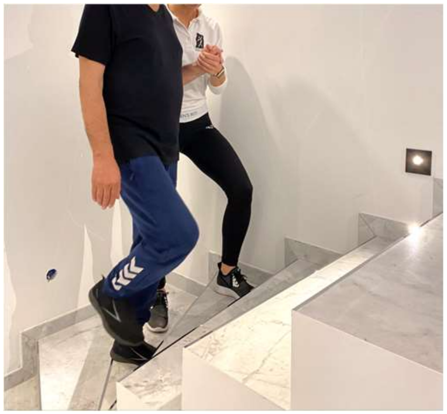
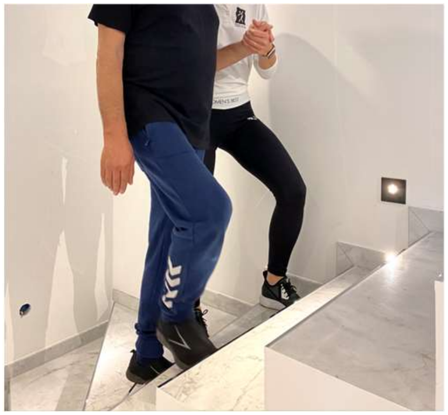

8
C

Mit/ohne Geländer bzw. Hilfsmittel die Stiegen gehen

- Es kann auch das Geländer plus ein Hilfsmittel (z.B. Gehstock) verwendet werden.

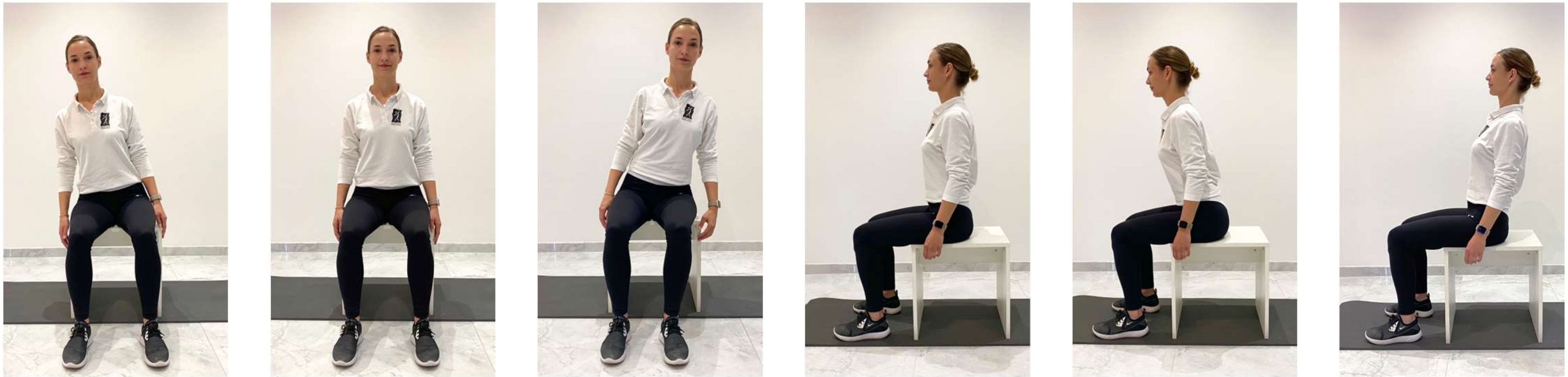

9
A

Gewichtsverlagerung im Sitzen in versch. Bewegungsrichtungen

- Nach links/rechts, vorne/hinten, kreisend nach links/rechts
- Falls notwendig, kann Unterstützung durch den Therapeuten oder durch Festhalten gegeben werden
- Variation: auf labiler Unterlage oder am Pezziball

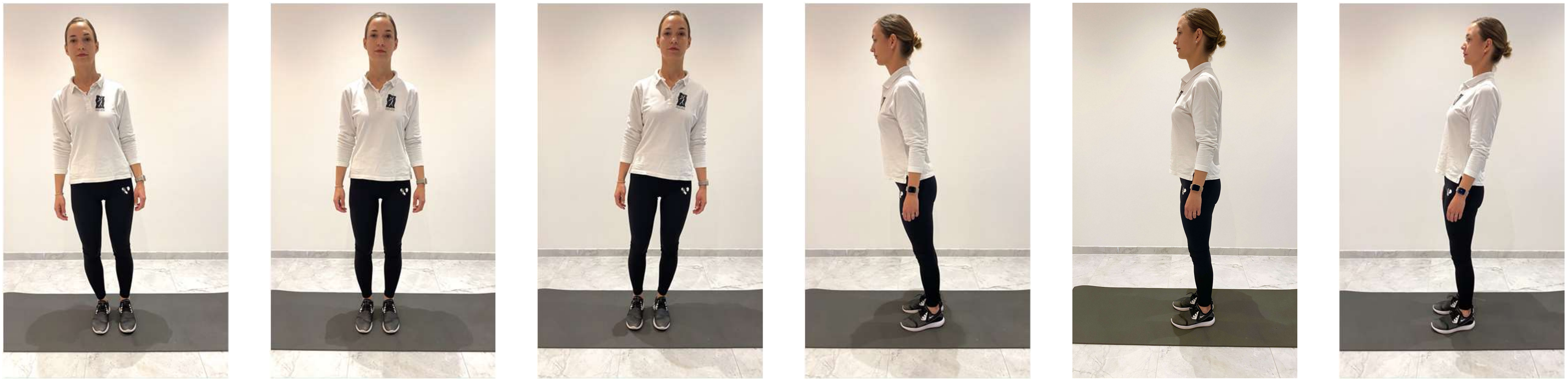

9
B

Gewichtsverlagerung im Stehen in versch. Bewegungsrichtungen

- Nach links/rechts, vorne/hinten, kreisend nach links/rechts
- Falls notwendig, kann Unterstützung durch den Therapeuten oder durch Festhalten gegeben werden
- Variation: auf labiler Unterlage

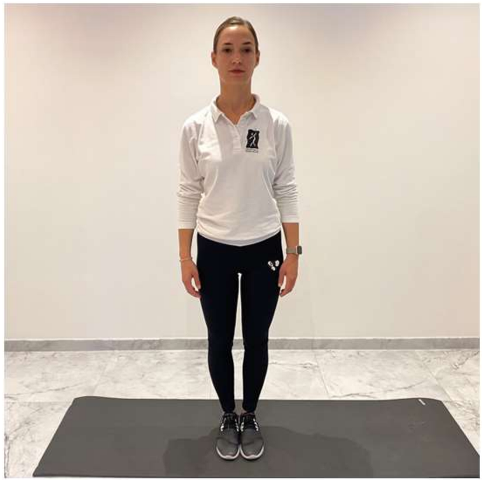

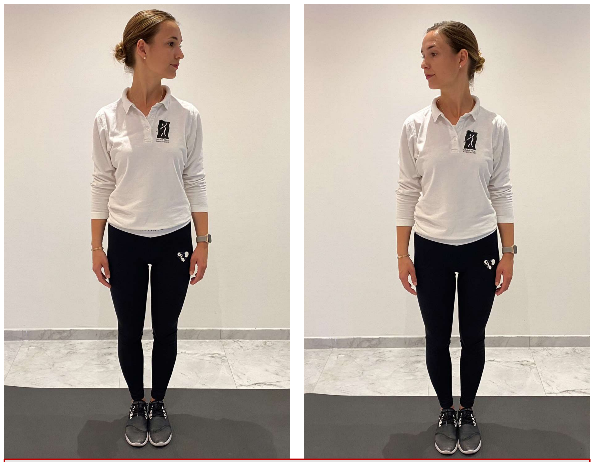

Steigerung 1

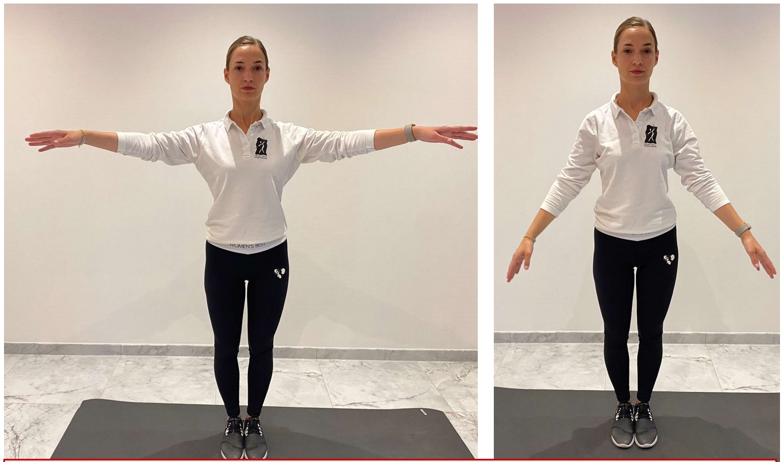

Steigerung 2

10 A

Mit geschlossenen Füßen stehen und das Gleichgewicht halten: Position für 15 Sekunden halten

- Steigerung: Position länger halten (z.B. 60 Sekunden), Arm- oder Kopfbewegungen machen, einen Ball o.ä. fangen

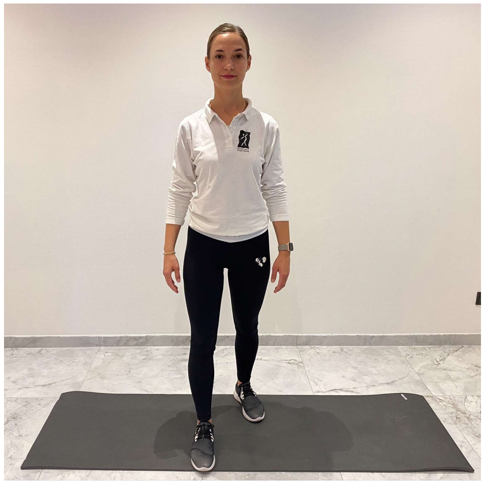

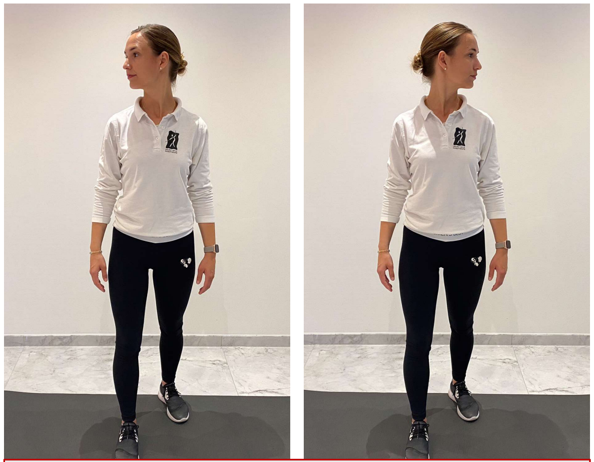

Steigerung 1

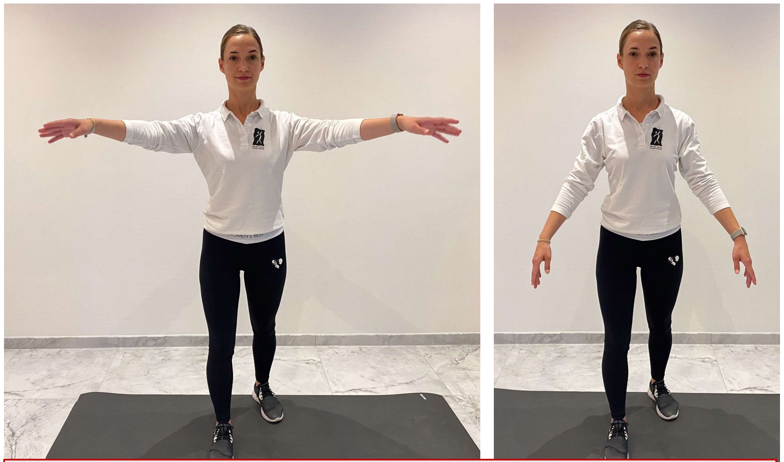

Steigerung 2

10 B

In Schrittstellung stehen und das Gleichgewicht halten: Position für 15 Sekunden halten

- Steigerung: Position länger halten (z.B. 60 Sekunden), Arm- oder Kopfbewegungen machen, einen Ball o.ä. fangen, Stehen im Tandem-Stand

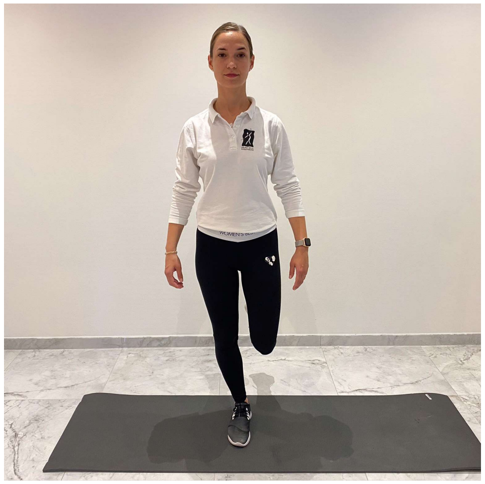

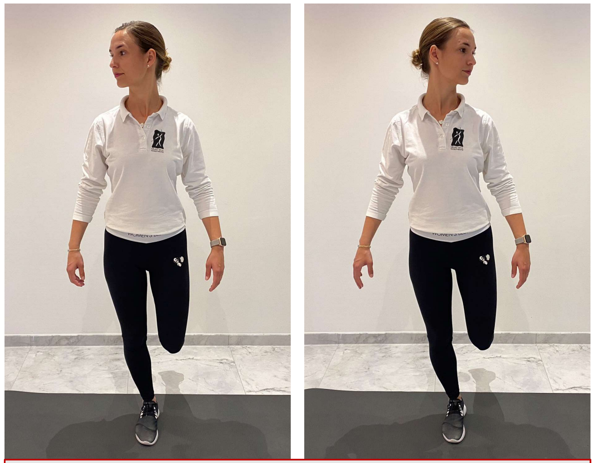

Steigerung 1

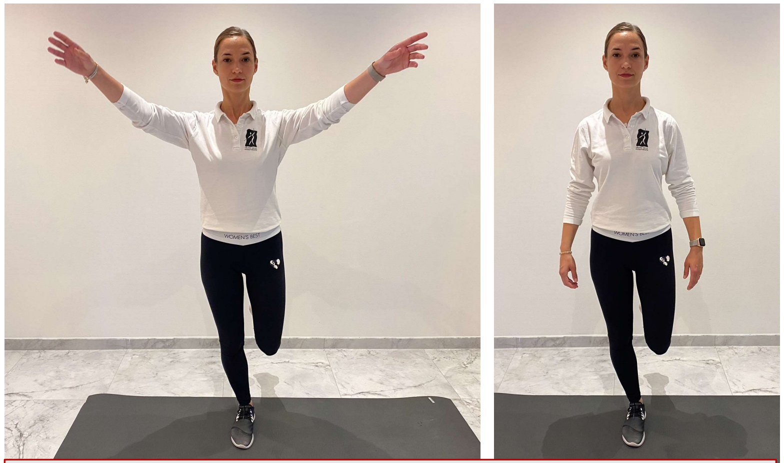

Steigerung 2

10 C

Im Einbeinstand stehen und das Gleichgewicht halten: Position für 15 Sekunden halten

- Steigerung: Position länger halten (z.B. 60 Sekunden), Arm- oder Kopfbewegungen machen, einen Ball o.ä. fangen

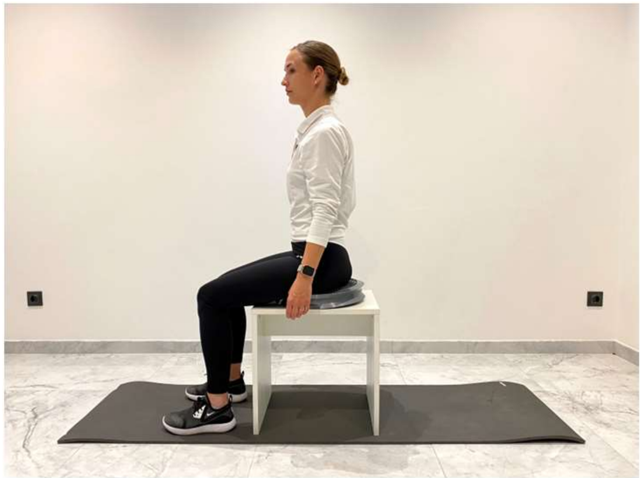

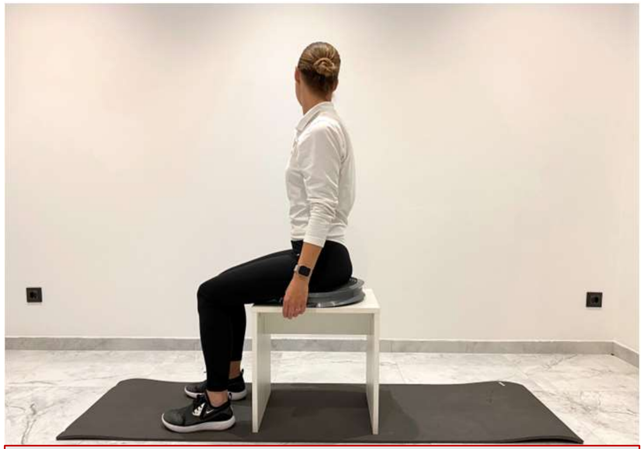

Steigerung 1

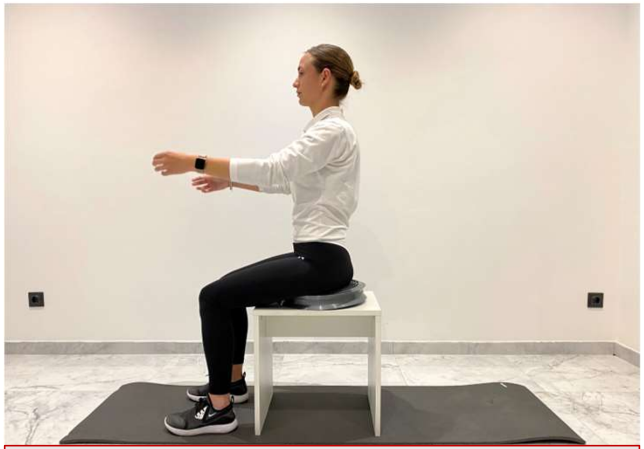

Steigerung 2

11 A

Im Sitzen mit labiler Unterlage: Position für 15 Sekunden halten

- Steigerung: Position länger halten (z.B. 60 Sekunden), Arm- oder Kopfbewegungen machen, einen Ball o.ä. fangen

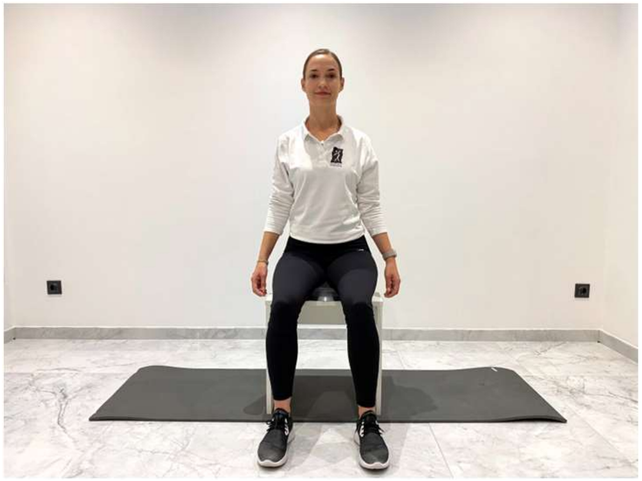
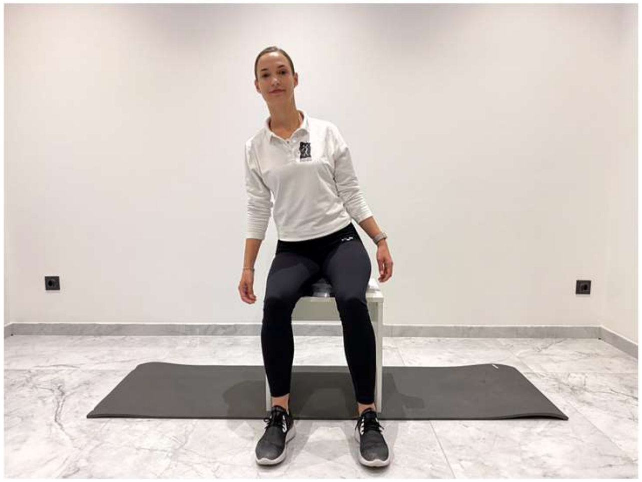
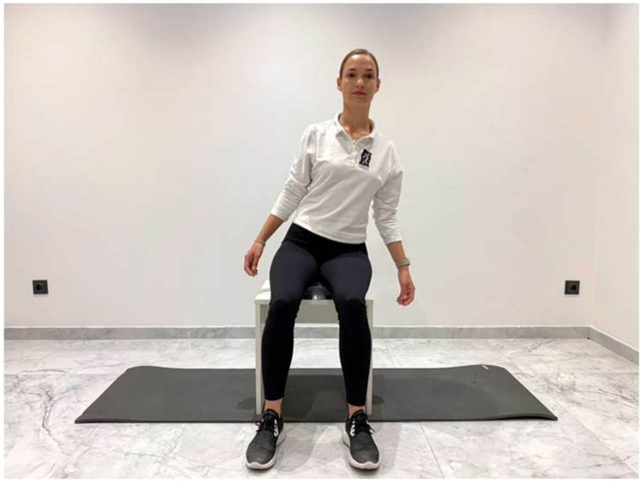

11 B

Im Sitzen mit labiler Unterlage: zur Seite neigen und probieren die Position zu halten

- Variation: Arm- oder Kopfbewegungen machen, Pezziball anstatt labiler Unterlage

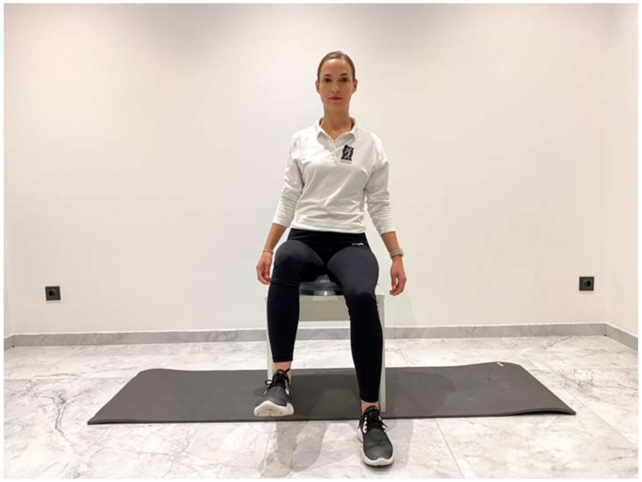

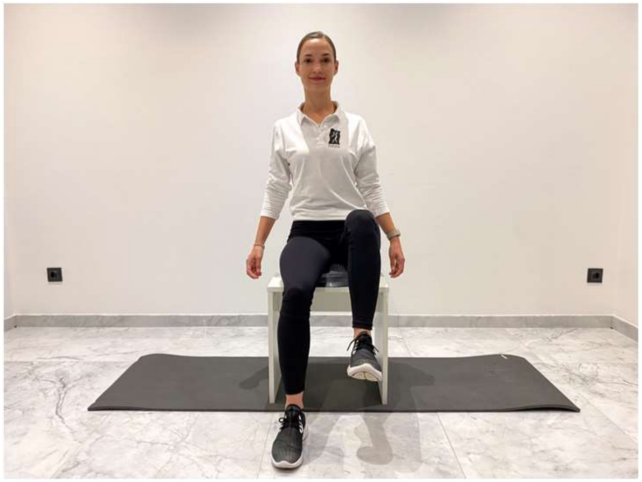

11 C

Im Sitzen mit labiler Unterlage: probieren den Fuß abzuheben

- Variation: Pezziball anstatt labiler Unterlage

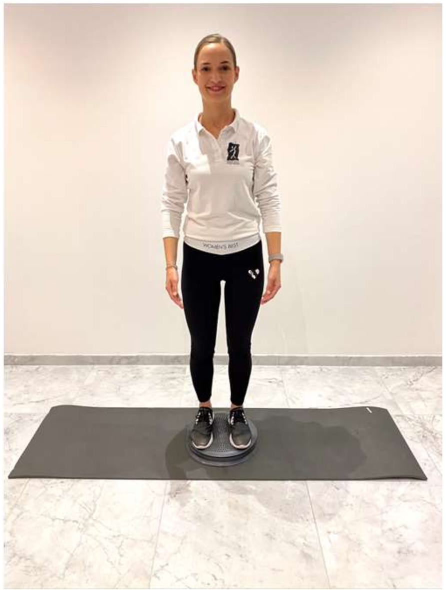

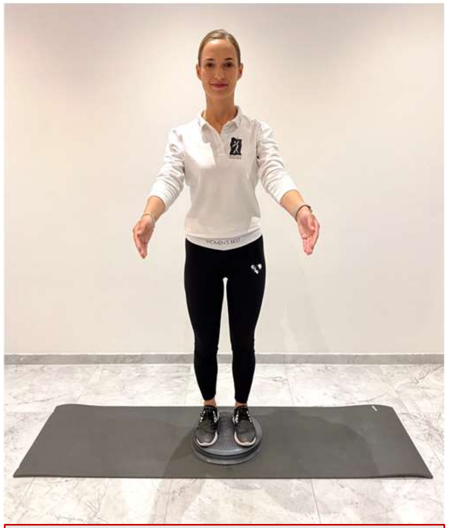

Steigerung 1

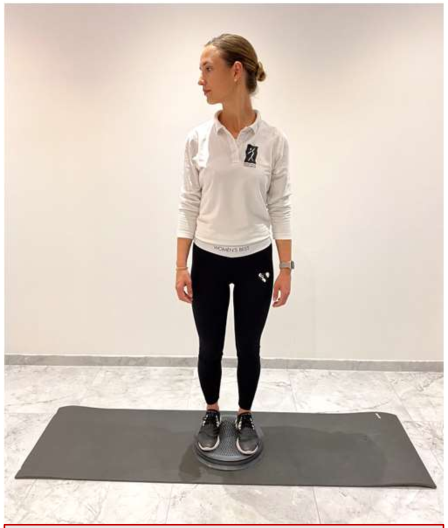

Steigerung 2

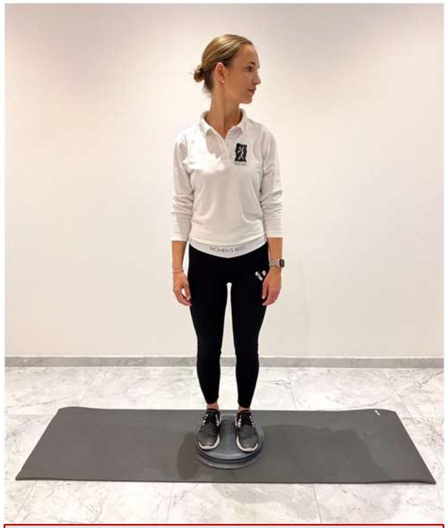

Steigerung 2

12 A

Im Stehen mit labiler Unterlage: Position für 15 Sekunden halten

- Steigerung: Position länger halten (z.B. 60 Sekunden), Arm- oder Kopfbewegungen machen

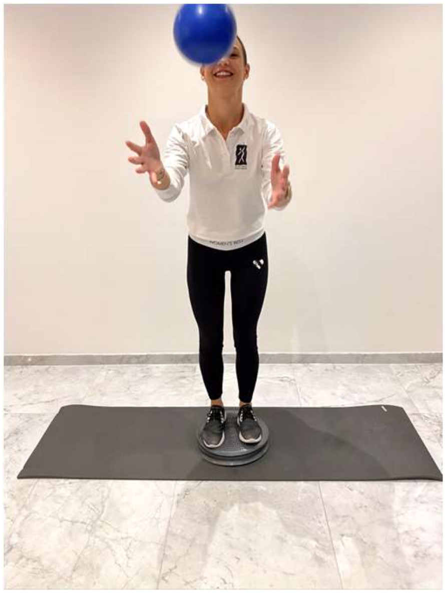

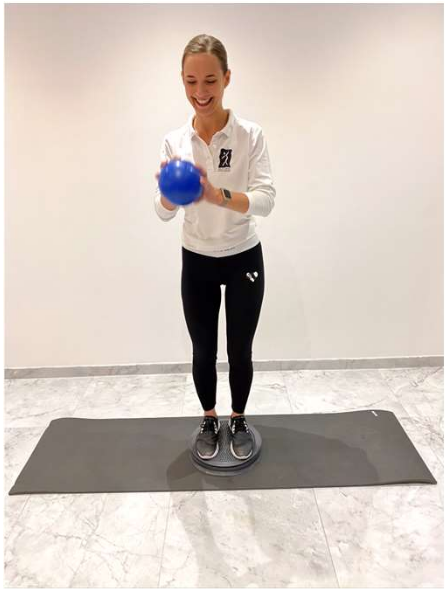

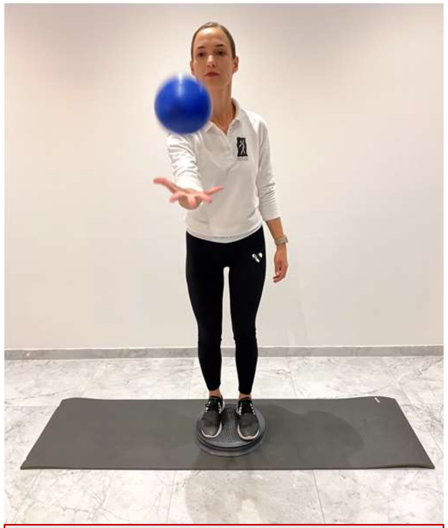

Variation 1

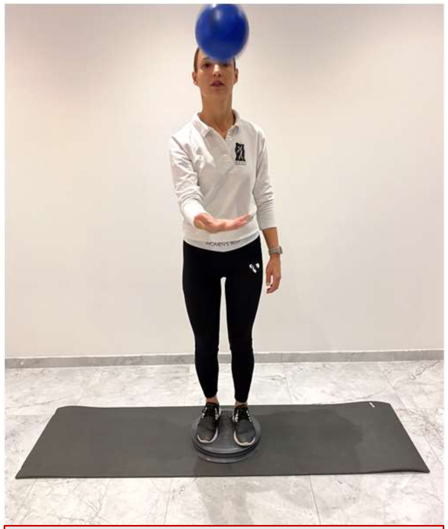

Variation 1

12 B

Im Stehen mit labiler Unterlage: einen Ball fangen

- Variation: die Gegenstände können verändert werden (z.B. Luftballon, Medizinball, kleiner Ball, etc.), um die Übung einfacher oder schwerer zu gestalten; mit nur einer Hand fangen

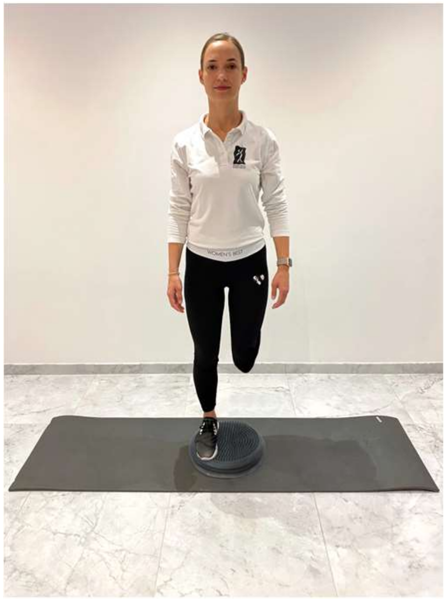

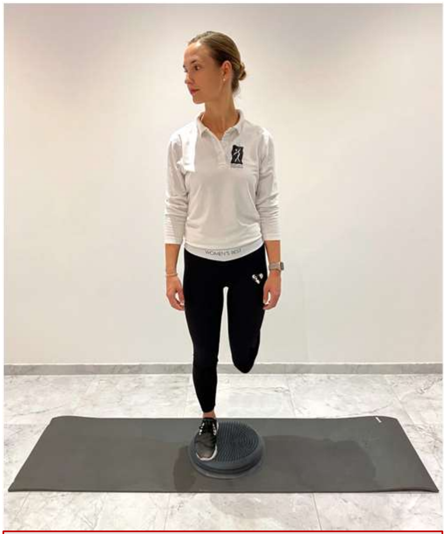

Steigerung 1

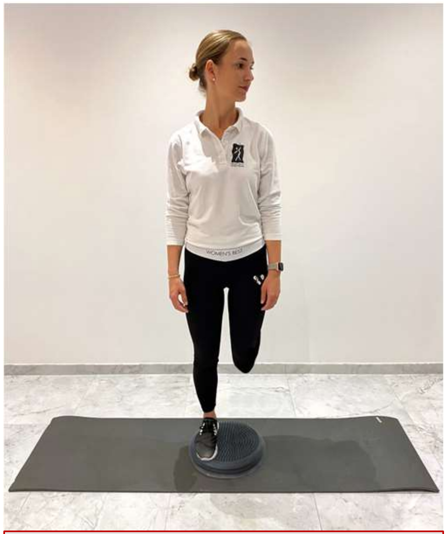

Steigerung 1

12 C

Einbeinstand auf labiler Unterlage: Position für 15 Sekunden halten

- Steigerung: Position länger halten (z.B. 60 Sekunden), Arm-, Bein- oder Kopfbewegungen machen

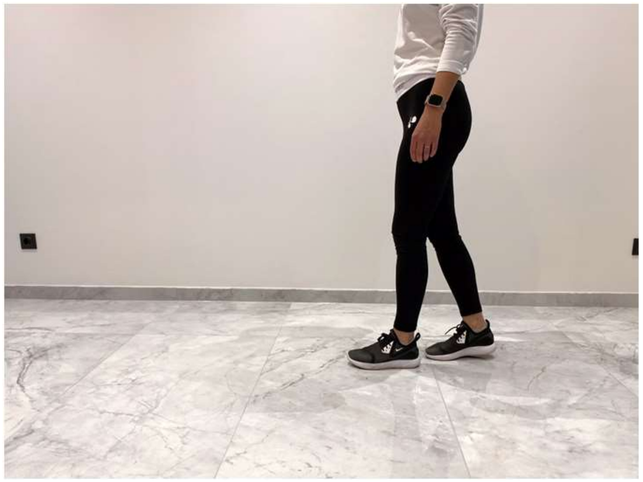

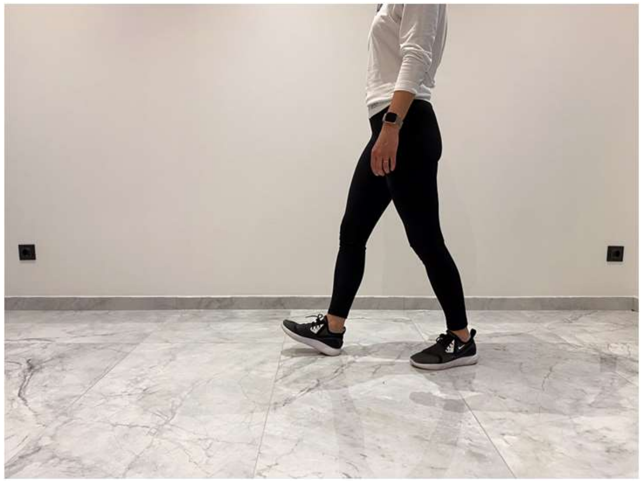

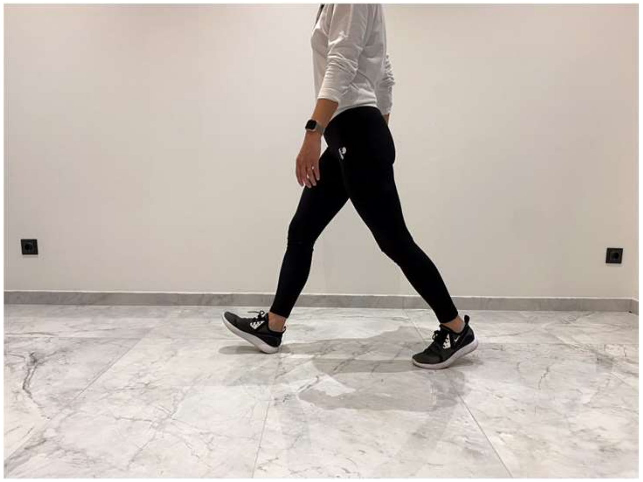

13
A

Gehen: Variation der Schrittlänge

- Möglichkeiten: Gehen mit kleinen oder großen Schritten

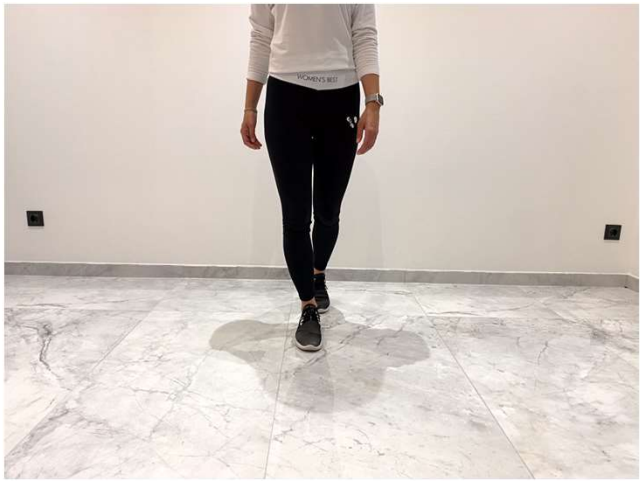

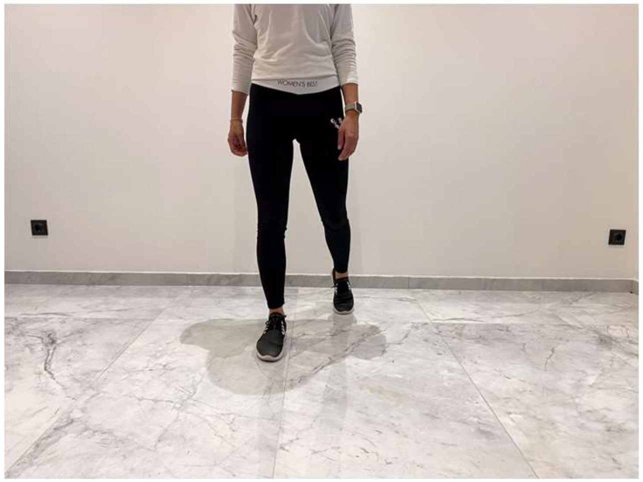

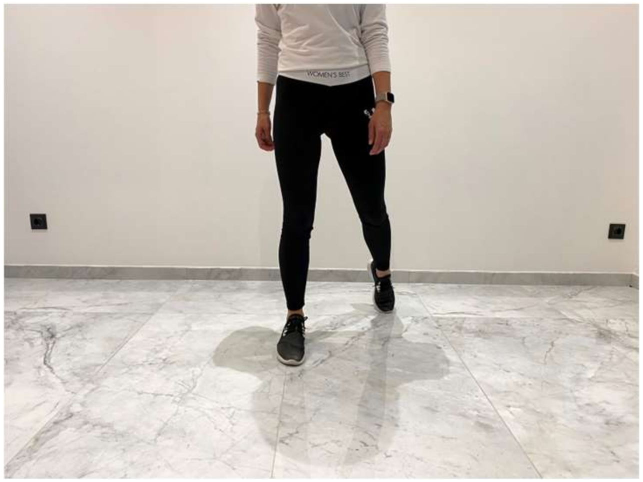

13 B

Gehen: Variation der Spurbreite

- Möglichkeiten: Gehen mit schmalen oder breiten Schritten

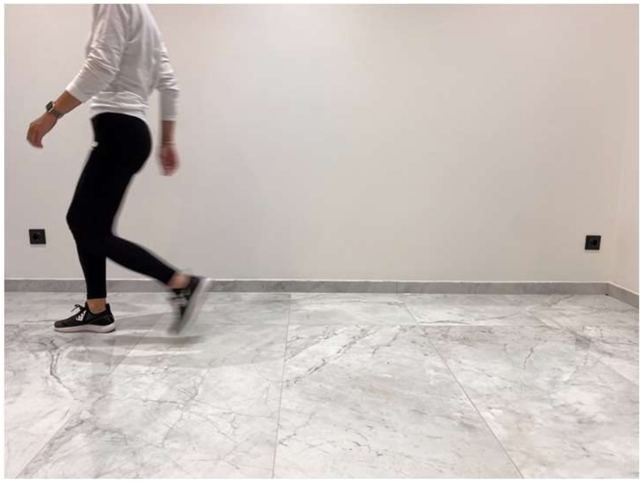
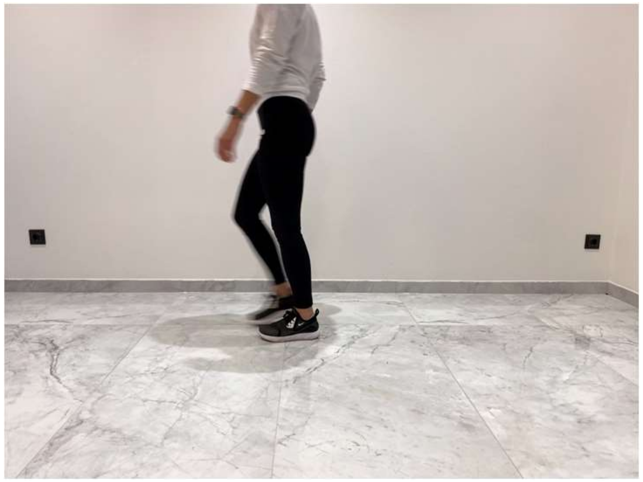
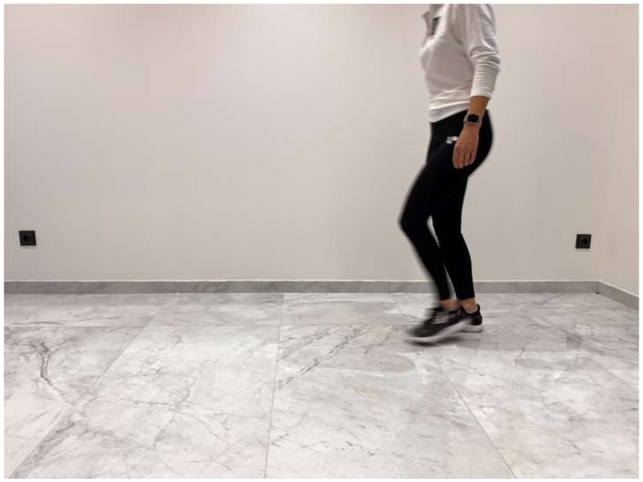

13 C

Gehen: Variation der Geschwindigkeit

- Möglichkeiten: Gehen mit langsamen oder schnellen Schritten

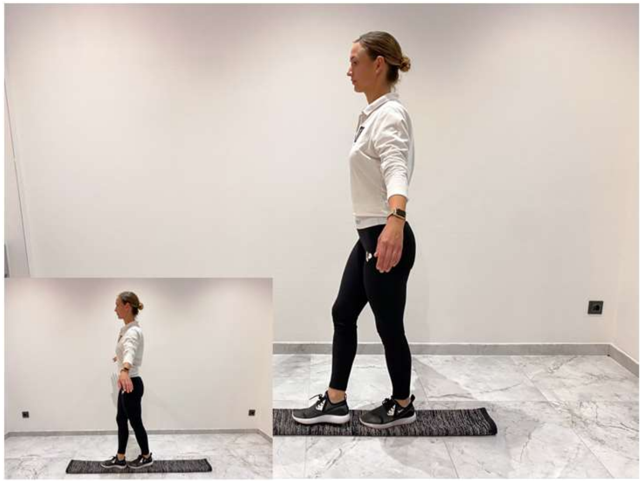

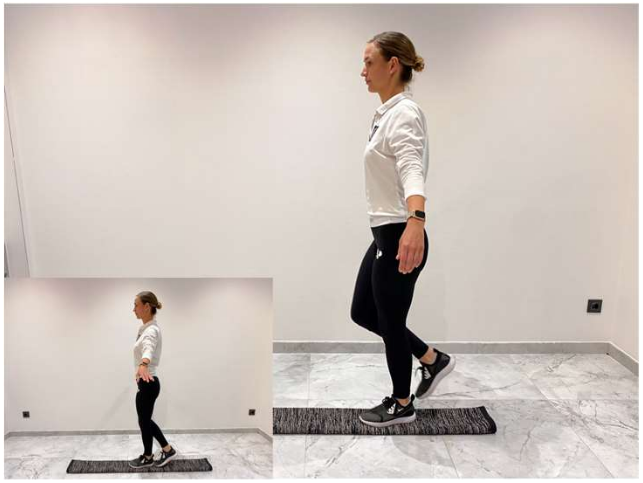

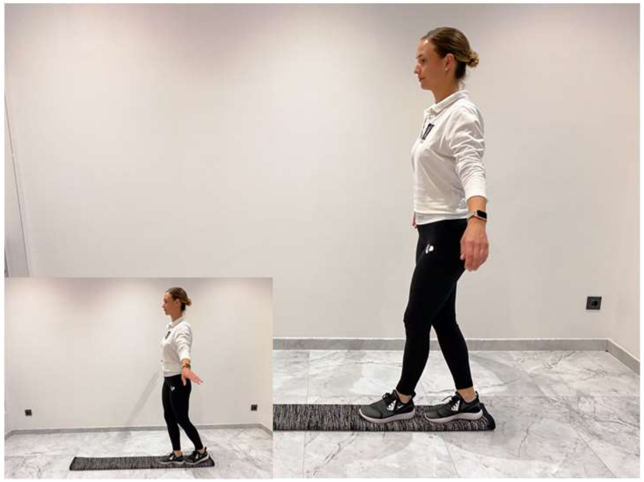

14 A

Auf einer Linie balancieren

- Mit/ohne Unterstützung des Therapeuten bzw. Unterstützung der Hände (Arme seitlich ausgestreckt zum Ausbalancieren)

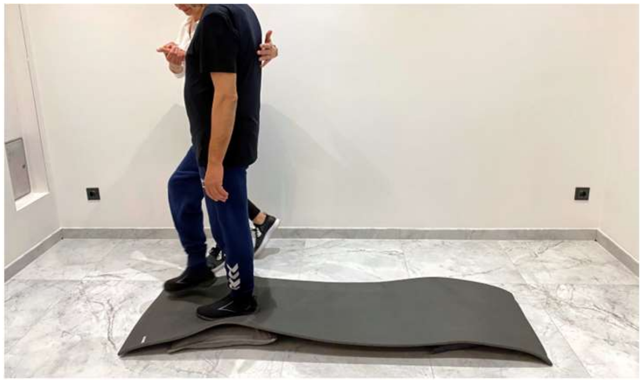
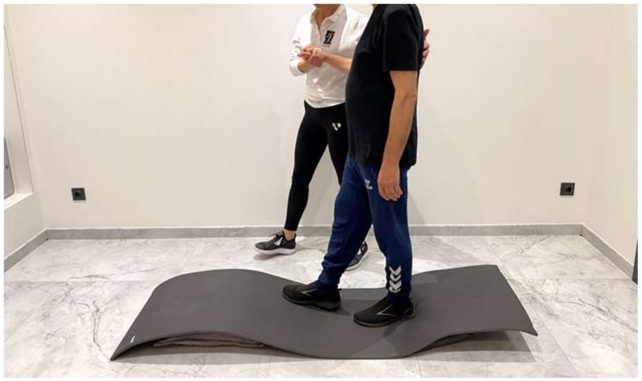
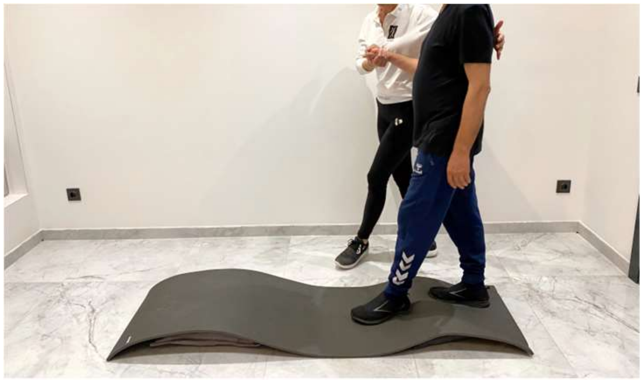

14 B

Auf unebenem Untergrund gehen

- Wenn kein unebener Boden zur Verfügung steht, können auch Gegenstände unter einer Gymnastikmatte platziert werden oder Kissen am Boden ausgelegt werden
- Gegebenenfalls mit Hilfsmittel oder Hilfsperson

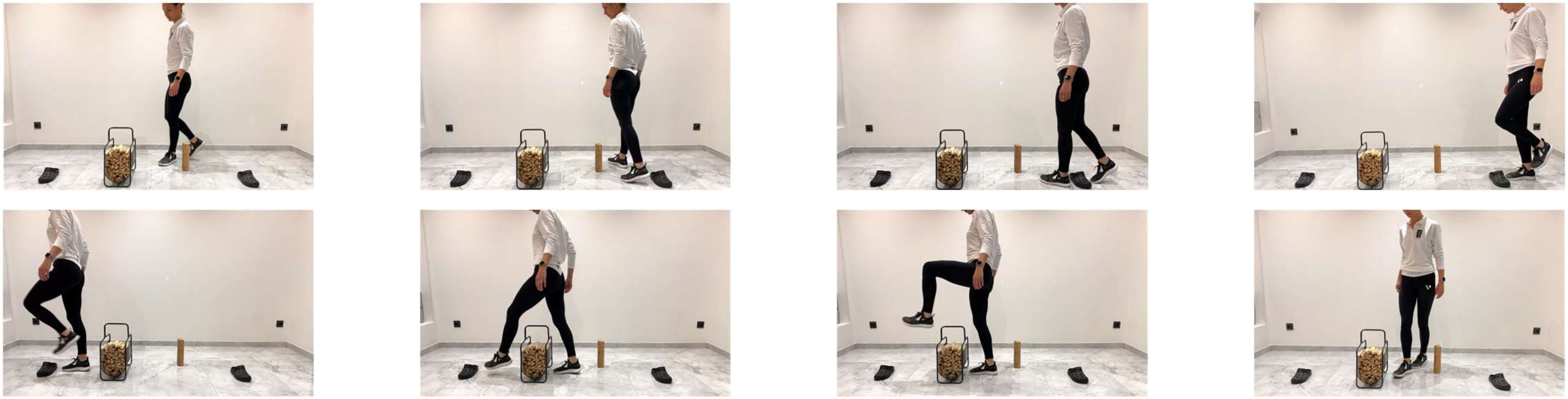

14
C

Parkour: verschiedene Gegenstände

- Dazu können Hindernisse zum Übersteigen (z.B. Stufen, Schuhkartons, etc.) oder zum Herumgehen (Hütchen, Schuhe, etc.) auf einem langen Gang platziert werden
- Gegebenenfalls mit Hilfsmittel oder Hilfsperson

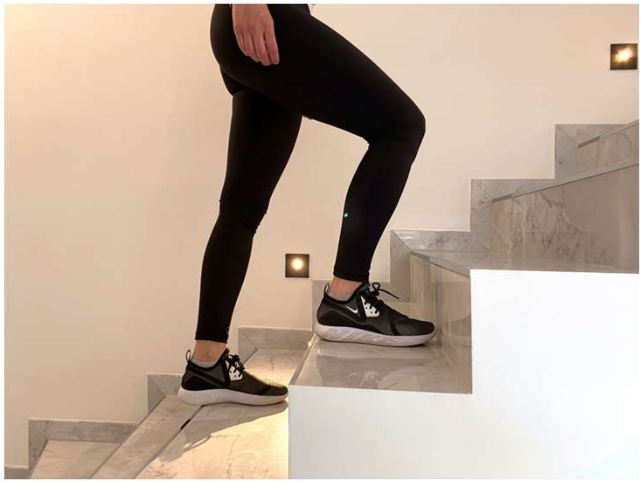

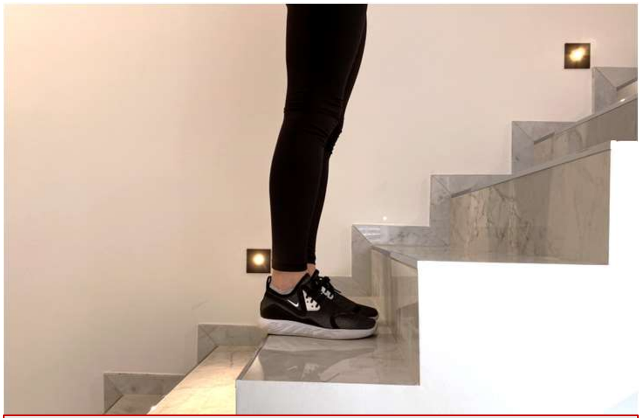

Beistellschritt

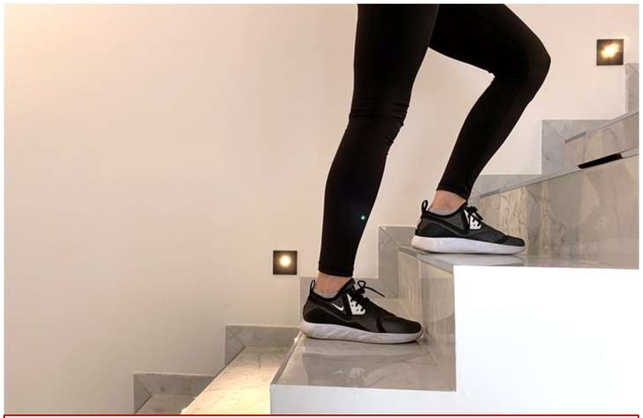

Alternierend

15 A

Stiegen steigen aufwärts in verschiedenen Variationen

- Mit dem rechten/linken Fuß nachsteigen (Beistellschritt)
- Alternierend
- Alternierend über 2 Stufen, etc.

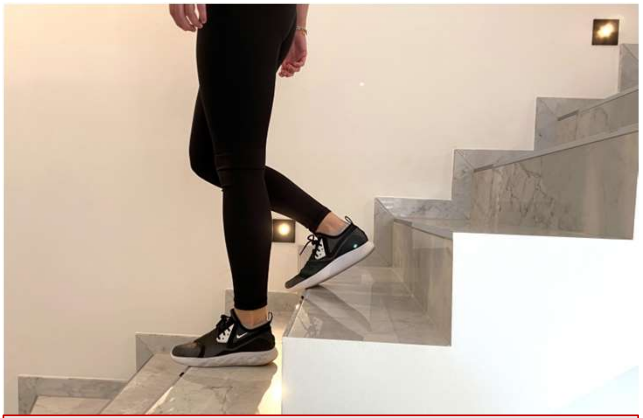

Alternierend

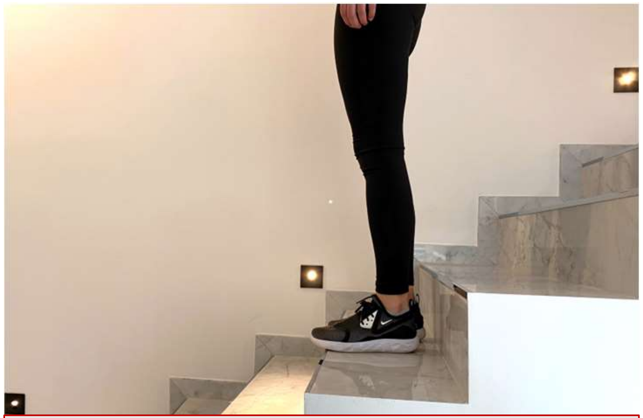

Beistellschritt

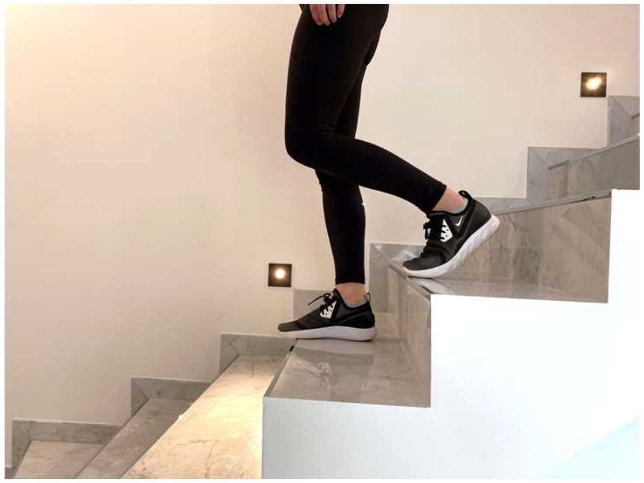

15 B

Stiegen steigen abwärts in verschiedenen Variationen

- Mit dem rechten/linken Fuß nachsteigen (Beistellschritt)
- Alternierend
- Alternierend über 2 Stufen, etc.

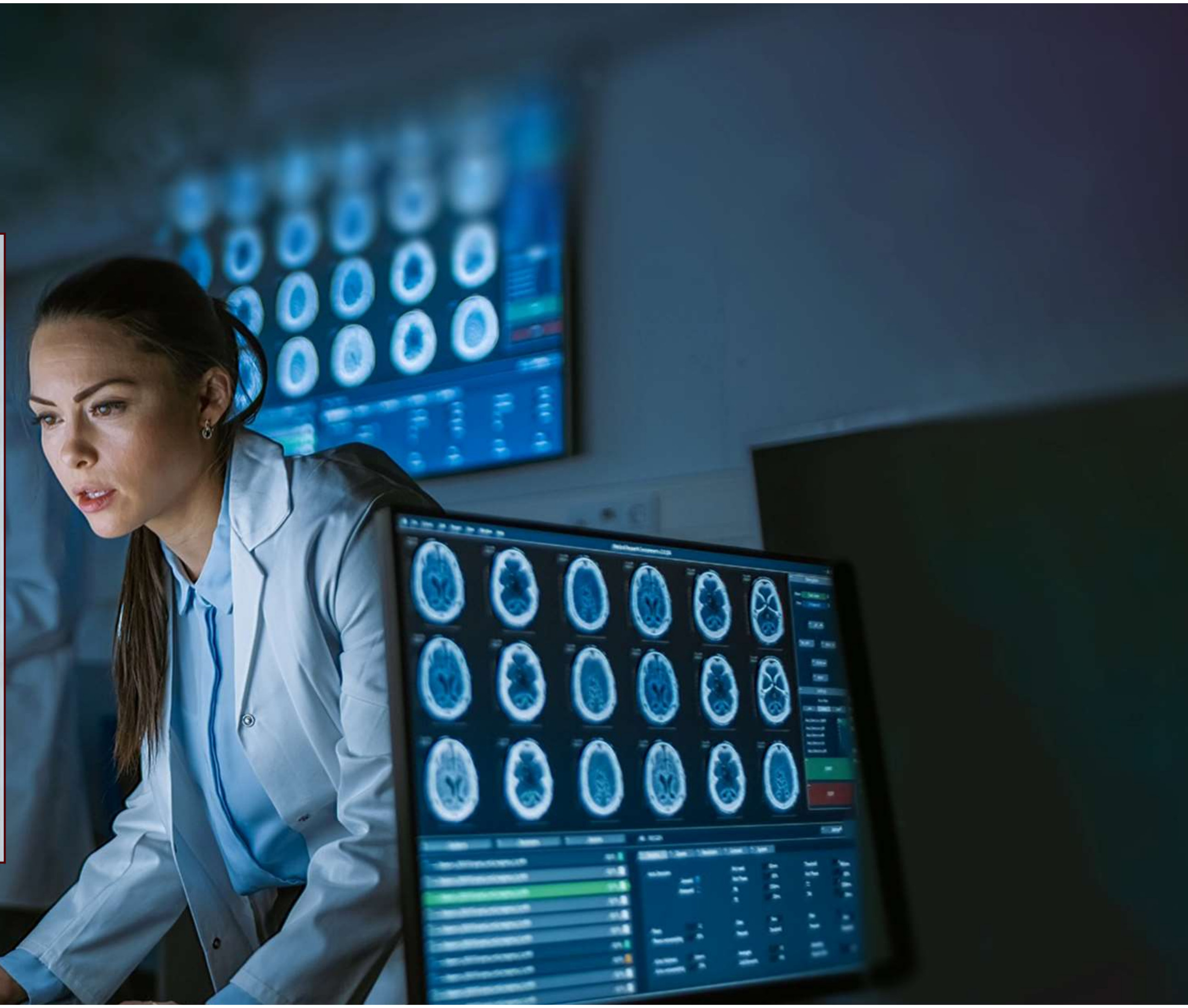

VASCage

The COMET-Centre VASCage is funded within the
COMET Programme - Competence Centres for Excellent Technologies
by

- Austrian Ministry for Climate Action, Environment, Energy, Mobility, Innovation and Technology
- Austrian Ministry of Labour and Economy

and the federal states

- Tyrol
- Salzburg
- Vienna

The COMET Programme is conducted by the Austrian Research Promotion Agency (FFG).

Federal Ministry
Republic of Austria
Labour and Economy

wirtschafts agentur wien

Tirol STANDORTAGENTUR TIROL

FFG Promoting Innovation.

LAND SALZBURG

Federal Ministry
Republic of Austria
Climate Action, Environment, Energy, Mobility, Innovation and Technology
